# Spatial datasets to define operational boundaries within four marine basins delineated by distance from shore, depth, EEZ and protected status, accompanied by maps and statistical summary tables

**DOI:** 10.1016/j.dib.2018.11.118

**Published:** 2018-11-28

**Authors:** Jeff Jenness, S.W.K. van den Burg, José Aguilar-Manjarrez, Melanie Torrie

**Affiliations:** a Jenness Enterprises, Flagstaff, AZ, USA; b Wageningen Economic Research, PO Box 29703, 2502 LS The Hague, the Netherlands; c Food and Agriculture Organization of the United Nations, Rome, Italy

## Abstract

This data article provides several datasets delineating, illustrating and summarizing marine regions within exclusive economic zones (EEZs) of 69 coastal states within the Atlantic, Baltic/North Sea, Mediterranean and Caribbean marine basins. Datasets include two GIS feature classes stored in a Version 10.0 file geodatabase: (1) A polygon feature class delineating operational boundaries within EEZs based on sovereign authority, distance bands from shore, depth ranges, and whether the region lies within a recognized protected area, and (2) A polyline feature class of shorelines for each coastal state. Both feature classes are fully described in the metadata. Datasets also include Excel files summarizing each marine basin and coastal state, summarized by area and proportion of all combinations of distance, depth and protected status. Datasets also include maps illustrating and describing all combinations of distance, depth and protection status, plus shoreline length, for each coastal state and for each marine basin. Datasets also include Visual Basic for Applications (VBA) code files used to generate all datasets, summary tables and maps. These datasets support analysis described in “Assessment of the geographical potential for co-use of marine space, based on operational boundaries for Blue Growth sectors” (van den Burg et al., 2018), and allow readers to extend the regional assessments described in that article down to individual country-level assessments.

**Specifications table**

**Table:** 

Subject area	*Economics and marine governance strategies*
More specific subject area	*Multiple use of offshore regions by marine industries*
Type of data	*GIS feature classes, Excel summary tables, map images and custom VBA functions*
How data were acquired	*Initial bathymetry, EEZ, state boundary and protected area datasets were downloaded from publicly-available sources, then were processed using various standard and custom GIS functions to produce these final products. All VBA functions were written by the corresponding author*.
Data format	*Processed and analyzed*.
Experimental factors	*All downloaded spatial geometric objects were examined for topological errors and repaired when necessary, then projected into a common spatial coordinate system and datum, and intersected with each other to produce a single dataset with comprehensive coverage of EEZs categorized into all combinations of depth range, distance band, protected status and sovereign state*.
Experimental features	*Data features include spatial datasets suitable for analysis and mapping in GIS, summary statistical tables and maps*.
Data source location	*Exclusive economic zones of 69 countries within the Atlantic, Baltic/North Sea, Mediterranean and Caribbean marine basins*.
Data accessibility	*These data are supplied with this data article*.
Related research article	*van den Burg, S.W.K., Aguilar-Manjarrez, J., Jenness, J., Torres, M., 2018. Assessment of the geographical potential for co-use of marine space, based on operational boundaries for Blue Growth sectors, Mar. Policy. In Press.*[1]

**Value of the data**

• These data provide an easy and convenient way to calculate how much area in an EEZ meet multiple criteria for distance-from-shore, depth range and protected status. Furthermore, the GIS feature classes allow you to quickly identify the exact boundaries of these regions.
• Based on these multiple criteria, it is straightforward to identify which regions support various marine industries, and which regions can support multiple industries simultaneously. These data would serve as a useful reference and analytical tool for marine resource managers.
• These data provide accurate area values because they are calculated directly from latitude/longitude coordinates using spherical geometry. Accuracy is not degraded by projection distortion.
• The GIS feature classes can be loaded directly into standard GIS systems, where they can be intersected and analyzed with other GIS data to enhance research or management strategies that may be influenced by ocean depth, distance from shore or protected status.

## 1 Data

These data provide resource managers with three tools to analyze and map regions within EEZs that lie within specific distance ranges from shore, within multiple depth ranges, and which lay within designated marine protected areas. These data are directly useful for identifying how much area is available for particular marine industries, and identifying where those areas are:

1. Two GIS feature classes are provided, which can be reviewed and analyzed in many GIS software packages. One feature class (“MARIBE_Operational_Boundaries”) is a polygon dataset delineating all regions within EEZs based on depth range, distance from shore, protected status, sovereign authority and MARIBE region, and also includes accurate area values for each polygon. The second feature class (“GAUL_Shorelines_Maribe_Countries”) is a polyline dataset delineating the shoreline of each country, and includes accurate length values for each shoreline. Both feature classes are stored in an Esri file geodatabase (v. 10.0).
2. Four Excel files summarize areas and proportions of each MARIBE region that lie within each combination of depth, distance from shore and protection status. Seventy-two additional Excel files provide similar statistics for each country and for a few areas that share a joint regime. Three countries (France, Netherlands and United Kingdom) are sovereign over EEZs in the Caribbean as well as EEZs in either the Atlantic, Baltic/North Sea and/or Mediterranean, and in these cases the Caribbean portions are summarized separately.
3. Four regional-level maps and 72 country-level maps illustrate the spatial distribution of depth ranges, distance bands and protected areas within each respective EEZ region.

Discussions and examples of how these data can be used are provided in van den burg et al. [1].

## 2 Experimental design, materials and methods

Version 9 of the World Exclusive Economic Zone (EEZ) shapefile [2] was downloaded from http://www.marineregions.org. MARIBE maritime regions were defined as the combined offshore regions of all countries with coastlines along the Caribbean, European Atlantic, Baltic/North Sea, and Mediterranean/Black Sea basins. Bathymetric data were downloaded from the General Bathymetric Chart of the Oceans (GEBCO) [4], [5], available at http://www.gebco.net/data_and_products/gridded_bathymetry_data/gebco_30_second_grid/. The Global Administrative Unit Layers shapefile (GAUL), published by the Food and Agriculture Organization of the United Nations [3], was downloaded from http://www.fao.org/geonetwork/srv/en/metadata.show?id=12691. The World Database of Protected Areas (WDPA) [6] was downloaded in file geodatabase format from the Protected Planet website at www.protectedplanet.net.

MARIBE maritime regions were first delineated into jurisdictional boundaries based on the EEZ polygons. Within each EEZ, standard buffering functions were used to identify regions extending out at 5, 12, 16, 24 and 200 NM from land areas extracted from the. A few small slivers of area were both within an EEZ, yet>200 NM from shore, and a separate classification was assigned to these areas.

These same regions were then delineated into depth ranges using GEBCO bathymetric data. These areas were classified into bands with depth ranges of 0 to −10 m, −10 m to −50 m, −50 m to −100 m, −100 m to −150 m, −150 m to −200 m, and <−200 m.

Finally, all areas were classified as either protected or not protected based on the WDPA delineations.

All jurisdictional boundaries, depth ranges and protected areas were intersected to produce a single polygon feature class that identified all areas within the EEZ of each MARIBE country by jurisdiction, depth range and protected status. Areas of each polygon were calculated using spherical trigonometric methods on Latitude/Longitude coordinates as described in Ref. [7]. The custom VBA function that performs this calculation is named “SphericalPolygonArea2”, and is available in the module “MyGeometricOperations.bas”.

Coastline length data were derived from the GAUL Administrative Unit polygons. This polyline dataset of marine shorelines was derived by (1) creating an “Ocean” polygon data set by clipping out the GAUL polygons from a general background polygon covering the entire earth, and then (2) deleting all small polygons from the “Ocean” data set which represented lakes, inland seas or internal holes in the GAUL data set, and then (3) creating a Coastline polyline data set by intersecting the GAUL polygons with the Ocean polygons. This last polyline data set is the linear intersection of all coastal countries with the oceans and therefore represents the coastline of all countries that face the ocean. Polyline lengths were calculated using Vincenty׳s equations for geodesic length over the ellipsoidal model of the earth [7], [8]. This shorelines layer provides the shoreline length per country. Cumulative shorelines per MARIBE basin were calculated by adding up all portions of shoreline that faced the basin.

After the final datasets were created, custom VBA code was applied to sum up the respective areas to produce summary tables describing the cumulative area in each unique combination of jurisdictional boundary, depth range and protected status. First the VBA function “AnalysisByCountry_PrepData” (in the module “ByCountryAnalysis.bas” was run to prepare the datasets for final analysis, and then the VBA function “AnalysisByCountry_Modified_for_Basin_Dec_29_2017” (in the module “ByCountryAnalysis.bas”) was used to summarize and map each MARIBE region. These regional analyses are presented in van den Burg et al. [1]. The VBA function “AnalysisByCountry” (in the module “ByCountryAnalysis.bas”) was used to analyze and map each country individually, and these datasets are included in this Data in Brief article.

A great deal of custom VBA code was required at all steps of this analysis, from matching up the EEZ sovereignty IDs to the GAUL Administrative IDs, to geometric processing of all datasets and eventually to summarizing the data in Excel format and generating maps. There were unexplained peculiarities in the source datasets that defeated the standard ArcGIS geoprocessing functions, such that intersection and union operations produced overlapping polygons in some cases, and empty holes in others. The datasets were also very large, causing ArcMap to run out of memory when the standard geoprocessing tools were used. Such issues required the development, extensive testing and validation of custom geometric processes. All code used in this project is included in the ArcMap MXD file “Operational_Boundaries_VBA_Code.mxd”, and presented in standalone modules in the folder “VBA Modules”.

## References

[1] van den Burg S.W.K., Aguilar-Manjarrez J., Jenness J., Torrie M. (2018). Assessment of the geographical potential for co-use of marine space, based on operational boundaries for Blue Growth sectors. Mar. Policy.

[2] S. Claus, N. De Hauwere, B. Vanhoorne, F. Souza Dias, P. Oset García, F. Hernandez, J. Mees, Exclusive Economic Zones Boundaries (EEZ), Flanders Mar. Inst. MarineRegions.org. [Online]. Belgium, 2016. 〈http://www.marineregions.org〉. (Accessed 18 October 2018).

[3] FAO, Global Administrative Unit Layers (GAUL), GeoNetwork. [Online]. Rome, 2015. 〈http://www.fao.org/geonetwork/srv/en/metadata.show?id=12691〉. (Accessed 18 October 2018).

[4] P. Weatherall, K. Marks, M. Jakobsson, T. Schmitt, S. Tani, J.E. Arndt, M. Rovere, D. Chayes, V. Ferrini, R. Wigley, GEBCO_2014 Grid, version 20150318, General Bathymetric Chart of the Oceans (2015). [Online]. United Kingdom, 2015. 〈http://www.gebco.net/data_and_products/gridded_bathymetry_data/gebco_30_second_grid/〉. (Accessed 18 October 2018).

[5] Weatherall P., Marks K., Jakobsson M., Schmitt T., Tani S., Arndt J.E., Rovere M., Chayes D., Ferrini V., Wigley R. (2015). A new digital bathymetric model of the world׳s oceans. Earth Space Sci..

[6] UNEP-WCMC & IUCN, Protected Planet: ProtectedPlanet WDPA Dataset; The World Database on Protected Areas (WDPA)/The Global Database on Protected Areas Management Effectiveness (GD-PAME). [Online]. United Kingdom, 2016. 〈www.protectedplanet.net〉. (Accessed 18 October 2018).

[7] J. Jenness, Tools for Graphics and Shapes, Jenness Enterprises, 2011. 〈http://www.jennessent.com/arcgis/shapes_graphics.htm〉. (Accessed 18 October 2018).

[8] Vincenty T. (1975). Direct and inverse solutions of geodesics on the ellipsoid with application of nested equations. Surv. Rev..

