# Supplementary material for: Spatial datasets to define operational boundaries within four marine basins delineated by distance from shore, depth, EEZ and protected status, accompanied by maps and statistical summary tables
Source: Data Brief. 2018 Nov 28;21:2638–42. doi: 10.1016/j.dib.2018.11.118 (PMC6290244; doi:10.1016/j.dib.2018.11.118)
Supplement: Supplementary file 1 — Supplementary material [file mmc1.pdf]

Conflicts of Interest Statement

Manuscript title: Manuscript No.: DIB-D-18-02949

Operational boundaries within four marine basins delineated by distance from shore, depth, EEZ and protected status, accompanied by maps and statistical summary tables

The authors whose names are listed immediately below certify that they have NO affiliations with or involvement in any organization or entity with any financial interest (such as honoraria; educational grants; participation in speakers' bureaus; membership, employment, consultancies, stock ownership, or other equity interest; and expert testimony or patent-licensing arrangements), or non-financial interest (such as personal or professional relationships, affiliations, knowledge or beliefs) in the subject matter or materials discussed in this manuscript.

Author names:

Jeff Jenness

Sander van den Burg

José Aguilar-Manjarrez

Melanie Torrie

The authors whose names are listed immediately below report the following details of affiliation or involvement in an organization or entity with a financial or non-financial interest in the subject matter or materials discussed in this manuscript. Please specify the nature of the conflict on a separate sheet of paper if the space below is inadequate.

Author names:

This statement is signed by all the authors to indicate agreement that the above information is true and correct (*a photocopy of this form may be used if there are more than 10 authors*):

Author's name (typed)	Author's signature	Date
Jeff Jenness		November 3, 2018
Sander van den Burg		November 5, 2018
José Aguilar-Manjarrez	José Aguilar-Manjarrez	November 5, 2018
Melanie Torrie		Nov. 7, 2018
