# Supplementary material for: Spatial datasets to define operational boundaries within four marine basins delineated by distance from shore, depth, EEZ and protected status, accompanied by maps and statistical summary tables
Source: Data Brief. 2018 Nov 28;21:2638–42. doi: 10.1016/j.dib.2018.11.118 (PMC6290244; doi:10.1016/j.dib.2018.11.118)
Supplement: Supplementary file 2 — Supplementary material [file mmc2.zip › Operational_Boundaries_Data/Maps/Country_Level_Maps.pdf]

Supplemental Maps

Albania: .... *Mediterranean* ....p. 3
Algeria: .... *Mediterranean* ....p. 4
Antigua and Barbuda: .... *Caribbean* ....p. 5
Bahamas: .... *Caribbean* ....p. 6
Barbados: .... *Caribbean* ....p. 7
Belgium: .... *North Sea* ....p. 8
Belize: .... *Caribbean* ....p. 9
Bosnia and Herzegovina: .... *Mediterranean* ....p. 10
Bulgaria: .... *Black Sea* ....p. 11
Colombia + Joint Regime: .... *Caribbean* ....p. 12
Colombia: .... *Caribbean* ....p. 13
Costa Rica: .... *Caribbean* ....p. 14
Croatia: .... *Mediterranean* ....p. 15
Cuba: .... *Caribbean* ....p. 16
Cyprus: .... *Mediterranean* ....p. 17
Denmark: .... *Baltic* ....p. 18
Dominica: .... *Caribbean* ....p. 19
Dominican Republic: .... *Caribbean* ....p. 20
Egypt: .... *Mediterranean* ....p. 21
Estonia: .... *Baltic* ....p. 22
Finland: .... *Baltic* ....p. 23
France: .... *Atlantic* and *Mediterranean* ....p. 24
France: .... *Caribbean* ....p. 25
Georgia: .... *Black Sea* ....p. 26
Germany: .... *North Sea* and *Baltic* ....p. 27
Greece: .... *Mediterranean* ....p. 28
Grenada: .... *Caribbean* ....p. 29
Guatemala: .... *Caribbean* ....p. 30
Guyana: .... *Caribbean* ....p. 31
Haiti: .... *Caribbean* ....p. 32
Honduras: .... *Caribbean* ....p. 33
Ireland: .... *Atlantic* ....p. 34
Israel: .... *Mediterranean* ....p. 35
Italy: .... *Mediterranean* ....p. 36
Jamaica + Joint Regime: .... *Caribbean* ....p. 37
Jamaica: .... *Caribbean* ....p. 38
Joint Regime: .... *Caribbean* ....p. 39
Latvia: .... *Baltic* ....p. 40
Lebanon: .... *Mediterranean* ....p. 41
Libya: .... *Mediterranean* ....p. 42
Lithuania: .... *Baltic* ....p. 43
Malta: .... *Mediterranean* ....p. 44
Mexico: .... *Gulf of Mexico* and *Caribbean* ....p. 45
Monaco: .... *Mediterranean* ....p. 46
Montenegro: .... *Mediterranean* ....p. 47
Morocco: .... *Mediterranean* ....p. 48

Netherlands: .... *North Sea* ....p. 49
Netherlands: .... *Caribbean* ....p. 50
Nicaragua: .... *Caribbean* ....p. 51
Norway: .... *North Sea* and *Norwegian Sea* ....p. 52
Palestine: .... *Mediterranean* ....p. 53
Panama: .... *Caribbean* ....p. 54
Poland: .... *Baltic* ....p. 55
Portugal: .... *Atlantic* ....p. 56
Romania: .... *Black Sea* ....p. 57
Russia: .... *Baltic* and *Black Sea* ....p. 58
Saint Kitts and Nevis: .... *Caribbean* ....p. 59
Saint Lucia: .... *Caribbean* ....p. 60
Saint Vincent and the Grenadines: .... *Caribbean* ....p. 61
Slovenia: .... *Mediterranean* ....p. 62
Spain: .... *Atlantic* and *Mediterranean* ....p. 63
Suriname: .... *Caribbean* ....p. 64
Sweden: .... *Baltic* ....p. 65
Syria: .... *Mediterranean* ....p. 66
Trinidad and Tobago: .... *Caribbean* ....p. 67
Tunisia: .... *Mediterranean* ....p. 68
Turkey: .... *Mediterranean* and *Black Sea* ....p. 69
Ukraine: .... *Black Sea* ....p. 70
United Kingdom: .... *Atlantic*, *North Sea* and *Mediterranean* ....p. 71
United Kingdom: .... *Caribbean* ....p. 72
United States: .... *Gulf of Mexico* and *Caribbean* ....p. 73
Venezuela: .... *Caribbean* ....p. 74

Food and Agriculture Organization of the United Nations

Albania: Mediterranean Region

©FAO, 2018

Kilometers
0 125

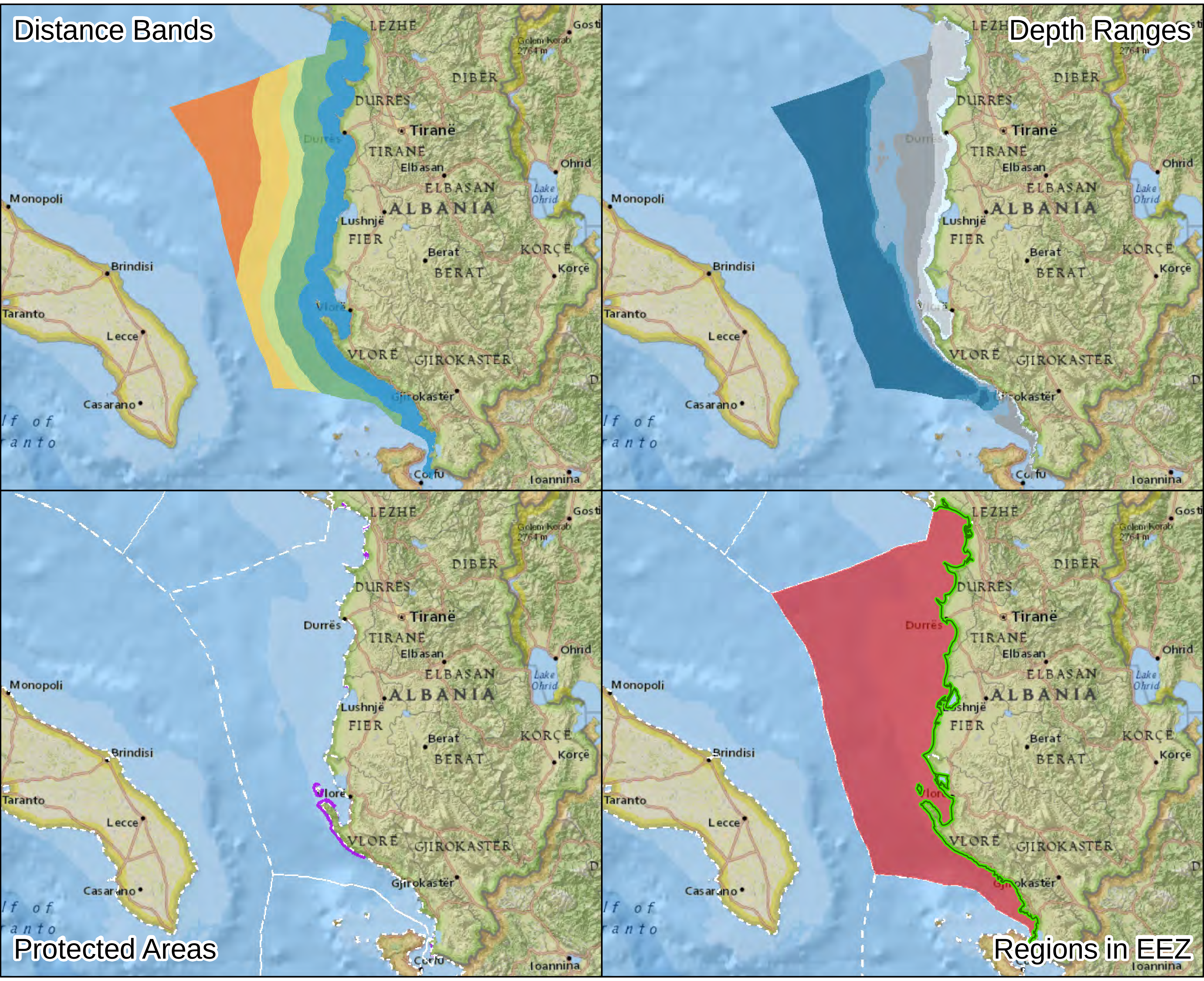

Distance Bands

- 0 to 5 NM
- 5 to 12 NM
- 12 to 16 NM
- 16 to 24 NM
- 24 to 200 NM

Depth Bands

- > -10m
- -10m to -50m
- -50m to -100m
- -100m to -150m
- -150m to -200m
- < -200m

Protected

- Protected Areas

Region

- Mediterranean

Coastline Length

579 Km

All Areas in Albania EEZ: Cell Values = Area in Km^2 [% Depth Band (Row), % Distance Band (Column), % EEZ]

	0 to 5 NM	5 to 12 NM	12 to 16 NM	16 to 24 NM	24 to 200 NM	> 200 NM	Total
> -10m	527 [100%, 17%, 4%]	0 [0%, 0%, 0%]	0 [0%, 0%, 0%]	0 [0%, 0%, 0%]	0 [0%, 0%, 0%]	0 [0%, 0%, 0%]	527 [4% of Total]
-10m to -50m	1,254 [92%, 40%, 10%]	116 [8%, 4%, 1%]	0 [0%, 0%, 0%]	0 [0%, 0%, 0%]	0 [0%, 0%, 0%]	0 [0%, 0%, 0%]	1,370 [11% of Total]
-50m to -100m	625 [33%, 20%, 5%]	1,069 [57%, 37%, 9%]	137 [7%, 10%, 1%]	47 [3%, 2%, 0%]	0 [0%, 0%, 0%]	0 [0%, 0%, 0%]	1,878 [16% of Total]
-100m to -150m	190 [10%, 6%, 2%]	511 [27%, 18%, 4%]	632 [33%, 45%, 5%]	571 [30%, 25%, 5%]	0 [0%, 0%, 0%]	0 [0%, 0%, 0%]	1,905 [16% of Total]
-150m to -200m	161 [29%, 5%, 1%]	131 [23%, 5%, 1%]	16 [3%, 1%, 0%]	250 [45%, 11%, 2%]	0 [0%, 0%, 0%]	0 [0%, 0%, 0%]	558 [5% of Total]
<-200m	348 [6%, 11%, 3%]	1,065 [18%, 37%, 9%]	619 [11%, 44%, 5%]	1,411 [24%, 62%, 12%]	2,427 [41%, 100%, 20%]	0 [0%, 0%, 0%]	5,871 [48% of Total]
Total	3,105 [26% of Total]	2,891 [24% of Total]	1,405 [12% of Total]	2,279 [19% of Total]	2,427 [20% of Total]	0.000 [0% of Total]	12,108 Km^2

Areas in Albania EEZ Excluding Protected Areas: Cell Values = Area in Km^2 [% Depth Band (Row), % Distance Band (Column), % EEZ]

131 [1%] Km^2 Protected	0 to 5 NM	5 to 12 NM	12 to 16 NM	16 to 24 NM	24 to 200 NM	> 200 NM	Total
> -10m	465 [100%, 16%, 4%]	0 [0%, 0%, 0%]	0 [0%, 0%, 0%]	0 [0%, 0%, 0%]	0 [0%, 0%, 0%]	0 [0%, 0%, 0%]	465 [4% of Total]
-10m to -50m	1,218 [91%, 41%, 10%]	116 [9%, 4%, 1%]	0 [0%, 0%, 0%]	0 [0%, 0%, 0%]	0 [0%, 0%, 0%]	0 [0%, 0%, 0%]	1,334 [11% of Total]
-50m to -100m	597 [32%, 20%, 5%]	1,069 [58%, 37%, 9%]	137 [7%, 10%, 1%]	47 [3%, 2%, 0%]	0 [0%, 0%, 0%]	0 [0%, 0%, 0%]	1,850 [15% of Total]
-100m to -150m	185 [10%, 6%, 2%]	511 [27%, 18%, 4%]	632 [33%, 45%, 5%]	571 [30%, 25%, 5%]	0 [0%, 0%, 0%]	0 [0%, 0%, 0%]	1,899 [16% of Total]
-150m to -200m	160 [29%, 5%, 1%]	131 [23%, 5%, 1%]	16 [3%, 1%, 0%]	250 [45%, 11%, 2%]	0 [0%, 0%, 0%]	0 [0%, 0%, 0%]	557 [5% of Total]
<-200m	348 [6%, 12%, 3%]	1,065 [18%, 37%, 9%]	619 [11%, 44%, 5%]	1,411 [24%, 62%, 12%]	2,427 [41%, 100%, 20%]	0 [0%, 0%, 0%]	5,871 [49% of Total]
Total	2,974 [25% of Total]	2,891 [24% of Total]	1,405 [12% of Total]	2,279 [19% of Total]	2,427 [20% of Total]	0.000 [0% of Total]	11,977 Km^2

The designations employed and the presentation of material in the map do not imply the expression of any opinion whatsoever on the part of FAO concerning the legal or constitutional status of any country, territory or sea area, or concerning the delimitation of frontiers.

Background reference map from National Geographic. Content may not reflect National Geographic's current map policy. Sources: National Geographic, Esri, DeLorme, HERE, UNEP-WCMC, USGS, NASA, ESA, METI, NRCAN, GEBCO, NOAA, increment P Corp.

Projection: Azimuthal Equidistant
Datum: WGS 1984
False Easting: 0.0000
False Northing: 0.0000
Central Meridian: 19.1712
Latitude Of Origin: 40.7617

N W E S

Food and Agriculture Organization of the United Nations

Algeria: Mediterranean Region

©FAO, 2018

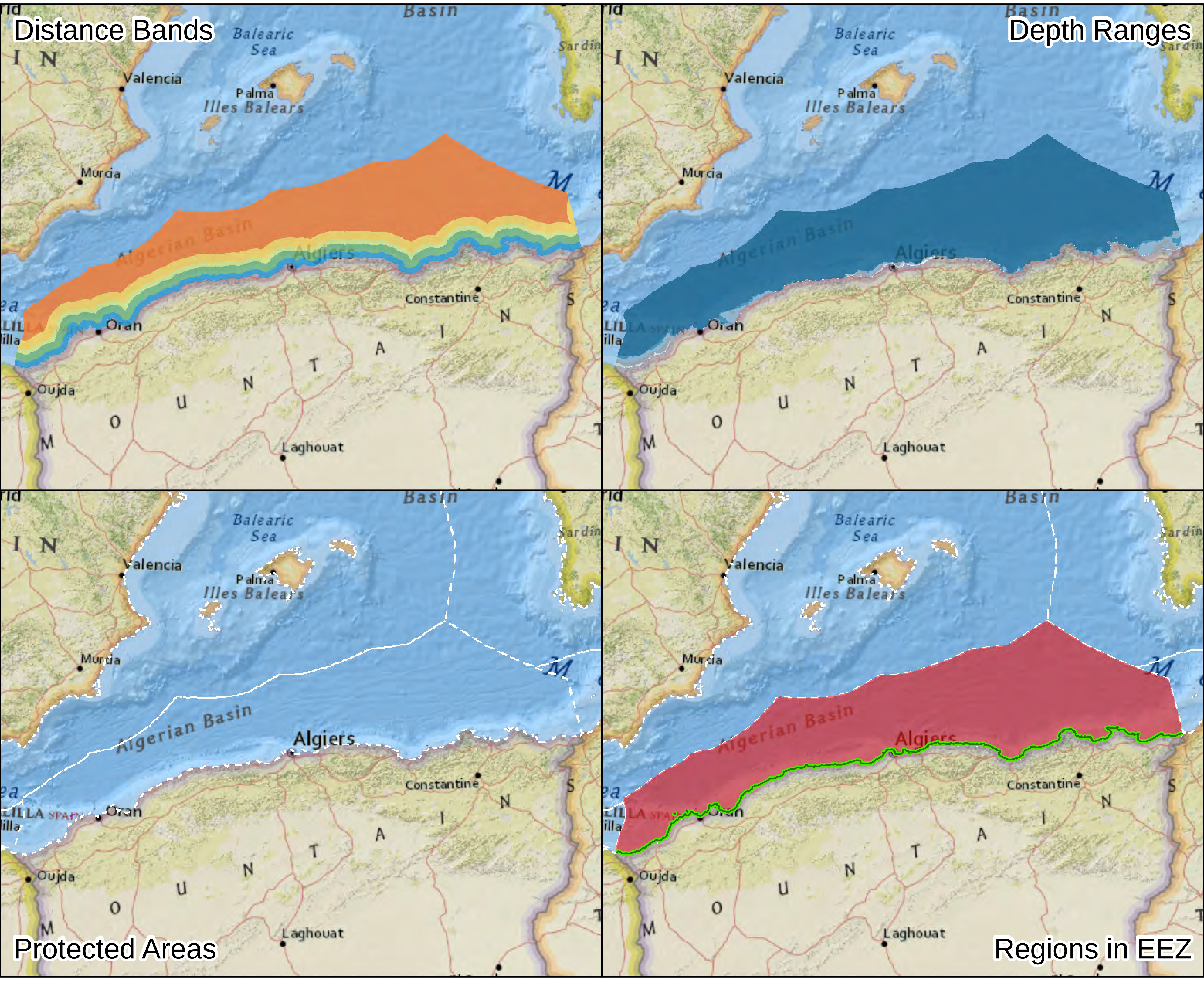

Distance Bands

- 0 to 5 NM
- 5 to 12 NM
- 12 to 16 NM
- 16 to 24 NM
- 24 to 200 NM

Depth Bands

- > -10m
- -10m to -50m
- -50m to -100m
- -100m to -150m
- -150m to -200m
- < -200m

Protected

- Protected Areas

Coastline Length

1,287 Km

Region

- Mediterranean

All Areas in Algeria EEZ: Cell Values = Area in Km^2 [% Depth Band (Row), % Distance Band (Column), % EEZ]

	0 to 5 NM	5 to 12 NM	12 to 16 NM	16 to 24 NM	24 to 200 NM	> 200 NM	Total
> -10m	199 [100%, 2%, 0%]	0 [0%, 0%, 0%]	0 [0%, 0%, 0%]	0 [0%, 0%, 0%]	0 [0%, 0%, 0%]	0 [0%, 0%, 0%]	199 [0% of Total]
-10m to -50m	524 [100%, 5%, 0%]	0 [0%, 0%, 0%]	0 [0%, 0%, 0%]	0 [0%, 0%, 0%]	0 [0%, 0%, 0%]	0 [0%, 0%, 0%]	524 [0% of Total]
-50m to -100m	1,445 [91%, 13%, 1%]	112 [7%, 1%, 0%]	36 [2%, 0%, 0%]	0.702 [0%, 0%, 0%]	0 [0%, 0%, 0%]	0 [0%, 0%, 0%]	1,593 [1% of Total]
-100m to -150m	1,596 [57%, 14%, 1%]	1,128 [41%, 8%, 1%]	47 [2%, 1%, 0%]	4 [0%, 0%, 0%]	0 [0%, 0%, 0%]	0 [0%, 0%, 0%]	2,776 [2% of Total]
-150m to -200m	922 [59%, 8%, 1%]	594 [38%, 4%, 0%]	27 [2%, 0%, 0%]	8 [1%, 0%, 0%]	0 [0%, 0%, 0%]	0 [0%, 0%, 0%]	1,551 [1% of Total]
<-200m	6,756 [5%, 59%, 5%]	12,944 [10%, 88%, 10%]	7,973 [6%, 99%, 6%]	16,005 [13%, 100%, 12%]	80,248 [65%, 100%, 61%]	0 [0%, 0%, 0%]	123,927 [95% of Total]
Total	11,441 [9% of Total]	14,779 [11% of Total]	8,082 [6% of Total]	16,018 [12% of Total]	80,248 [61% of Total]	0.000 [0% of Total]	130,569 Km^2

Areas in Algeria EEZ Excluding Protected Areas: Cell Values = Area in Km^2 [% Depth Band (Row), % Distance Band (Column), % EEZ]

29 [0%] Km^2 Protected	0 to 5 NM	5 to 12 NM	12 to 16 NM	16 to 24 NM	24 to 200 NM	> 200 NM	Total
> -10m	199 [100%, 2%, 0%]	0 [0%, 0%, 0%]	0 [0%, 0%, 0%]	0 [0%, 0%, 0%]	0 [0%, 0%, 0%]	0 [0%, 0%, 0%]	199 [0% of Total]
-10m to -50m	524 [100%, 5%, 0%]	0 [0%, 0%, 0%]	0 [0%, 0%, 0%]	0 [0%, 0%, 0%]	0 [0%, 0%, 0%]	0 [0%, 0%, 0%]	524 [0% of Total]
-50m to -100m	1,445 [91%, 13%, 1%]	112 [7%, 1%, 0%]	36 [2%, 0%, 0%]	0.702 [0%, 0%, 0%]	0 [0%, 0%, 0%]	0 [0%, 0%, 0%]	1,593 [1% of Total]
-100m to -150m	1,596 [57%, 14%, 1%]	1,128 [41%, 8%, 1%]	47 [2%, 1%, 0%]	4 [0%, 0%, 0%]	0 [0%, 0%, 0%]	0 [0%, 0%, 0%]	2,776 [2% of Total]
-150m to -200m	922 [59%, 8%, 1%]	594 [38%, 4%, 0%]	27 [2%, 0%, 0%]	8 [1%, 0%, 0%]	0 [0%, 0%, 0%]	0 [0%, 0%, 0%]	1,551 [1% of Total]
<-200m	6,728 [5%, 59%, 5%]	12,944 [10%, 88%, 10%]	7,973 [6%, 99%, 6%]	16,005 [13%, 100%, 12%]	80,248 [65%, 100%, 61%]	0 [0%, 0%, 0%]	123,898 [95% of Total]
Total	11,413 [9% of Total]	14,779 [11% of Total]	8,082 [6% of Total]	16,018 [12% of Total]	80,248 [61% of Total]	0.000 [0% of Total]	130,540 Km^2

The designations employed and the presentation of material in the map do not imply the expression of any opinion whatsoever on the part of FAO concerning the legal or constitutional status of any country, territory or sea area, or concerning the delimitation of frontiers.

Background reference map from National Geographic. Content may not reflect National Geographic's current map policy. Sources: National Geographic, Esri, DeLorme, HERE, UNEP-WCMC, USGS, NASA, ESA, METI, NRCAN, GEBCO, NOAA, increment P Corp.

Projection: Azimuthal Equidistant
Datum: WGS 1984
False Easting: 0.0000
False Northing: 0.0000
Central Meridian: 3.2175
Latitude Of Origin: 36.9388

Food and Agriculture Organization of the United Nations

Antigua and Barbuda: Caribbean Region

©FAO, 2018

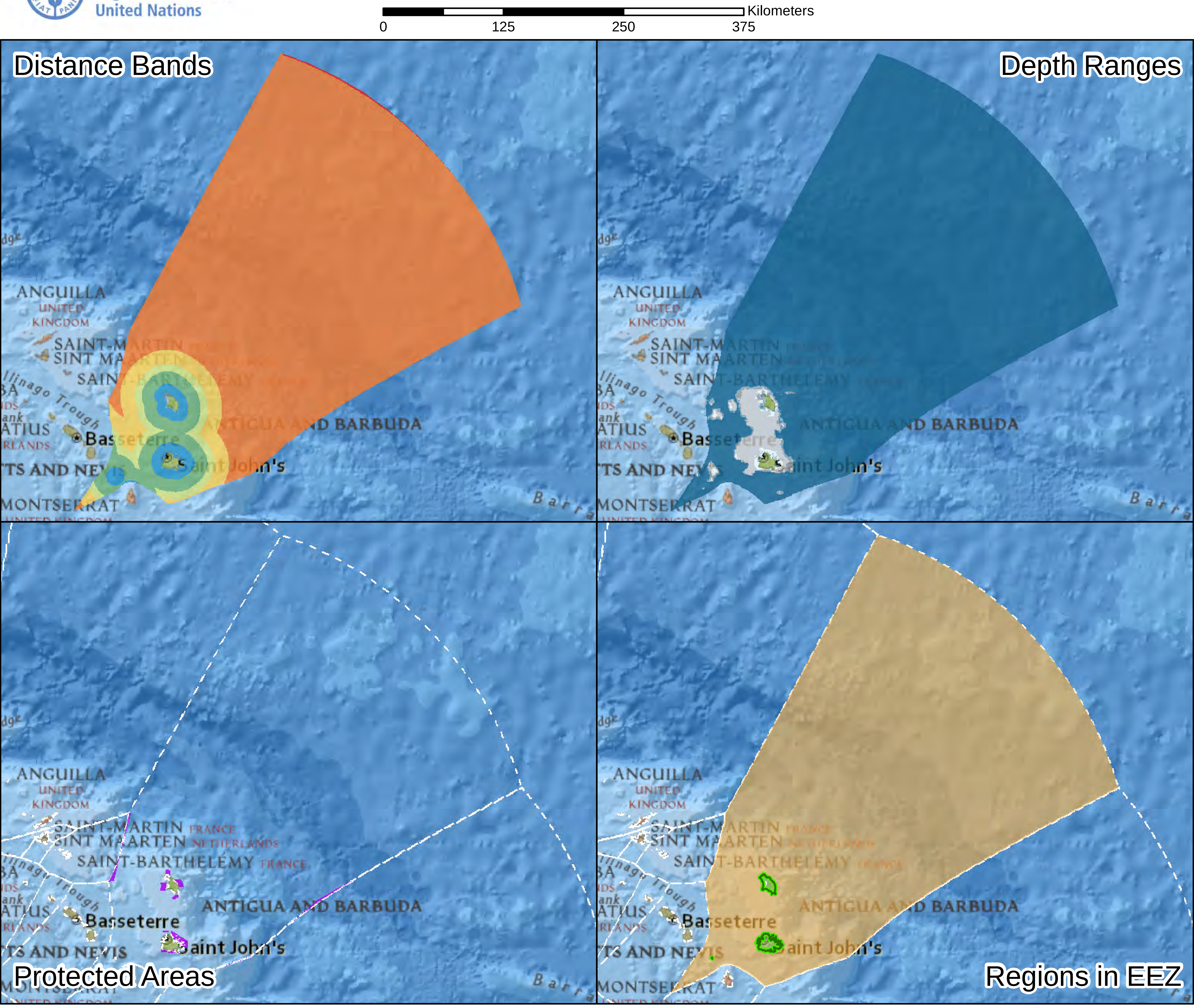

Distance Bands

- 0 to 5 NM
- 5 to 12 NM
- 12 to 16 NM
- 16 to 24 NM
- 24 to 200 NM
- > 200 NM

Depth Bands

- > -10m
- -10m to -50m
- -50m to -100m
- -100m to -150m
- -150m to -200m
- < -200m

Protected

- Protected Areas

Coastline Length

- 310 Km

Region

- Caribbean

All Areas in Antigua and Barbuda EEZ: Cell Values = Area in Km^2 [% Depth Band (Row), % Distance Band (Column), % EEZ]

	0 to 5 NM	5 to 12 NM	12 to 16 NM	16 to 24 NM	24 to 200 NM	> 200 NM	Total
> -10m	334 [87%, 15%, 0%]	11 [3%, 0%, 0%]	5 [1%, 0%, 0%]	25 [7%, 1%, 0%]	8 [2%, 0%, 0%]	0 [0%, 0%, 0%]	382 [0% of Total]
-10m to -50m	931 [36%, 42%, 1%]	1,455 [56%, 27%, 1%]	109 [4%, 4%, 0%]	54 [2%, 1%, 0%]	27 [1%, 0%, 0%]	0 [0%, 0%, 0%]	2,576 [2% of Total]
-50m to -100m	203 [41%, 9%, 0%]	171 [34%, 3%, 0%]	71 [14%, 2%, 0%]	31 [6%, 1%, 0%]	22 [4%, 0%, 0%]	0 [0%, 0%, 0%]	496 [0% of Total]
-100m to -150m	88 [30%, 4%, 0%]	101 [34%, 2%, 0%]	44 [15%, 2%, 0%]	33 [11%, 1%, 0%]	27 [9%, 0%, 0%]	0 [0%, 0%, 0%]	294 [0% of Total]
-150m to -200m	68 [28%, 3%, 0%]	83 [34%, 2%, 0%]	47 [19%, 2%, 0%]	28 [11%, 1%, 0%]	22 [9%, 0%, 0%]	0 [0%, 0%, 0%]	247 [0% of Total]
<-200m	607 [1%, 27%, 1%]	3,525 [3%, 66%, 3%]	2,651 [2%, 91%, 2%]	4,815 [4%, 97%, 4%]	96,039 [89%, 100%, 86%]	250 [0%, 100%, 0%]	107,886 [96% of Total]
Total	2,231 [2% of Total]	5,345 [5% of Total]	2,926 [3% of Total]	4,985 [4% of Total]	96,145 [86% of Total]	250 [0% of Total]	111,882 Km^2

Areas in Antigua and Barbuda EEZ Excluding Protected Areas: Cell Values = Area in Km^2 [% Depth Band (Row), % Distance Band (Column), % EEZ]

482 [0%] Km^2 Protected	0 to 5 NM	5 to 12 NM	12 to 16 NM	16 to 24 NM	24 to 200 NM	> 200 NM	Total
> -10m	191 [80%, 10%, 0%]	11 [5%, 0%, 0%]	5 [2%, 0%, 0%]	25 [10%, 1%, 0%]	8 [3%, 0%, 0%]	0 [0%, 0%, 0%]	240 [0% of Total]
-10m to -50m	841 [34%, 43%, 1%]	1,455 [59%, 27%, 1%]	109 [4%, 4%, 0%]	54 [2%, 1%, 0%]	27 [1%, 0%, 0%]	0 [0%, 0%, 0%]	2,486 [2% of Total]
-50m to -100m	184 [38%, 9%, 0%]	171 [36%, 3%, 0%]	71 [15%, 2%, 0%]	31 [6%, 1%, 0%]	22 [5%, 0%, 0%]	0 [0%, 0%, 0%]	478 [0% of Total]
-100m to -150m	80 [28%, 4%, 0%]	101 [36%, 2%, 0%]	44 [16%, 2%, 0%]	33 [12%, 1%, 0%]	25 [9%, 0%, 0%]	0 [0%, 0%, 0%]	284 [0% of Total]
-150m to -200m	63 [26%, 3%, 0%]	83 [35%, 2%, 0%]	47 [19%, 2%, 0%]	28 [12%, 1%, 0%]	20 [8%, 0%, 0%]	0 [0%, 0%, 0%]	239 [0% of Total]
<-200m	588 [1%, 30%, 1%]	3,525 [3%, 66%, 3%]	2,651 [2%, 91%, 2%]	4,805 [4%, 97%, 4%]	95,855 [89%, 100%, 86%]	250 [0%, 100%, 0%]	107,673 [97% of Total]
Total	1,947 [2% of Total]	5,345 [5% of Total]	2,926 [3% of Total]	4,975 [4% of Total]	95,956 [86% of Total]	250 [0% of Total]	111,400 Km^2

The designations employed and the presentation of material in the map do not imply the expression of any opinion whatsoever on the part of FAO concerning the legal or constitutional status of any country, territory or sea area, or concerning the delimitation of frontiers.

Background reference map from National Geographic. Content may not reflect National Geographic's current map policy. Sources: National Geographic, Esri, DeLorme, HERE, UNEP-WCMC, USGS, NASA, ESA, METI, NRCAN, GEBCO, NOAA, increment P Corp.

Projection: Azimuthal Equidistant
Datum: WGS 1984
False Easting: 0.0000
False Northing: 0.0000
Central Meridian: -60.5596
Latitude Of Origin: 18.7671

Food and Agriculture Organization of the United Nations

Bahamas: Caribbean Region

©FAO, 2018

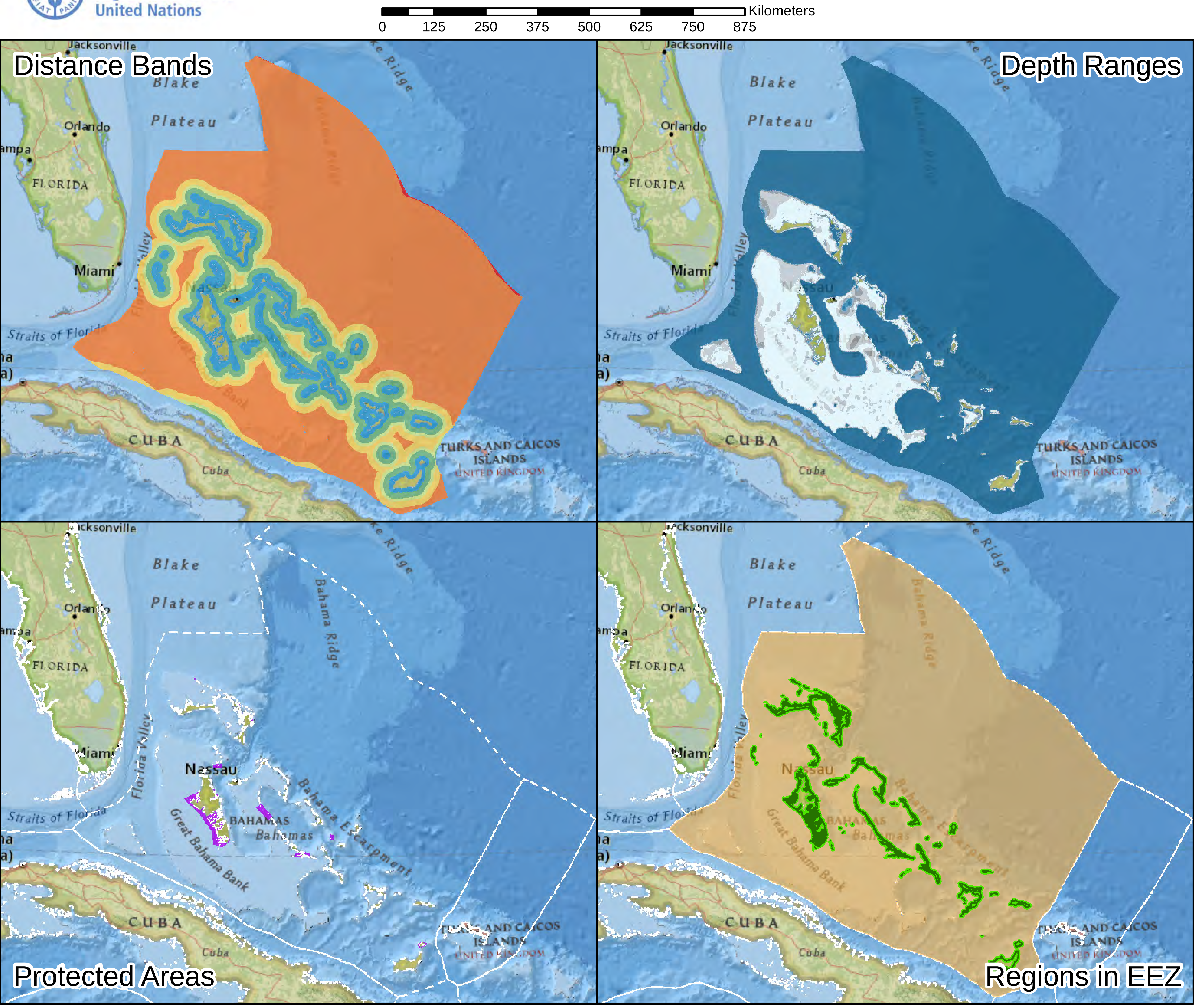

Distance Bands

- 0 to 5 NM
- 5 to 12 NM
- 12 to 16 NM
- 16 to 24 NM
- 24 to 200 NM
- > 200 NM

Depth Bands

- > -10m
- -10m to -50m
- -50m to -100m
- -100m to -150m
- -150m to -200m
- < -200m

Protected

- Protected Areas

Coastline Length

- 16,831 Km

Region

- Caribbean

All Areas in Bahamas EEZ: Cell Values = Area in Km^2 [% Depth Band (Row), % Distance Band (Column), % EEZ]

	0 to 5 NM	5 to 12 NM	12 to 16 NM	16 to 24 NM	24 to 200 NM	> 200 NM	Total
> -10m	20,255 [25%, 37%, 3%]	13,051 [16%, 18%, 2%]	5,003 [6%, 14%, 1%]	11,479 [14%, 18%, 2%]	31,621 [39%, 8%, 5%]	0 [0%, 0%, 0%]	81,409 [13% of Total]
-10m to -50m	7,961 [29%, 14%, 1%]	4,735 [18%, 7%, 1%]	1,856 [7%, 5%, 0%]	2,875 [11%, 5%, 0%]	9,605 [36%, 2%, 2%]	0 [0%, 0%, 0%]	27,034 [4% of Total]
-50m to -100m	3,887 [52%, 7%, 1%]	1,246 [17%, 2%, 0%]	337 [4%, 1%, 0%]	566 [8%, 1%, 0%]	1,468 [20%, 0%, 0%]	0 [0%, 0%, 0%]	7,505 [1% of Total]
-100m to -150m	2,435 [58%, 4%, 0%]	585 [14%, 1%, 0%]	196 [5%, 1%, 0%]	263 [6%, 0%, 0%]	744 [18%, 0%, 0%]	0 [0%, 0%, 0%]	4,223 [1% of Total]
-150m to -200m	1,807 [57%, 3%, 0%]	469 [15%, 1%, 0%]	146 [5%, 0%, 0%]	162 [5%, 0%, 0%]	566 [18%, 0%, 0%]	0 [0%, 0%, 0%]	3,150 [1% of Total]
<-200m	18,746 [4%, 34%, 3%]	50,779 [10%, 72%, 8%]	28,050 [6%, 79%, 5%]	46,988 [9%, 75%, 8%]	350,667 [71%, 89%, 57%]	1,267 [0%, 100%, 0%]	496,497 [80% of Total]
Total	55,091 [9% of Total]	70,866 [11% of Total]	35,588 [6% of Total]	62,334 [10% of Total]	394,671 [64% of Total]	1,267 [0% of Total]	619,817 Km^2

Areas in Bahamas EEZ Excluding Protected Areas: Cell Values = Area in Km^2 [% Depth Band (Row), % Distance Band (Column), % EEZ]

3,357 [1%] Km^2 Protected	0 to 5 NM	5 to 12 NM	12 to 16 NM	16 to 24 NM	24 to 200 NM	> 200 NM	Total
> -10m	18,610 [23%, 36%, 3%]	13,026 [16%, 18%, 2%]	5,003 [6%, 14%, 1%]	11,479 [14%, 18%, 2%]	31,621 [40%, 8%, 5%]	0 [0%, 0%, 0%]	79,740 [13% of Total]
-10m to -50m	7,279 [28%, 14%, 1%]	4,735 [18%, 7%, 1%]	1,856 [7%, 5%, 0%]	2,875 [11%, 5%, 0%]	9,605 [36%, 2%, 2%]	0 [0%, 0%, 0%]	26,352 [4% of Total]
-50m to -100m	3,517 [49%, 7%, 1%]	1,246 [17%, 2%, 0%]	337 [5%, 1%, 0%]	566 [8%, 1%, 0%]	1,468 [21%, 0%, 0%]	0 [0%, 0%, 0%]	7,134 [1% of Total]
-100m to -150m	2,229 [55%, 4%, 0%]	585 [15%, 1%, 0%]	196 [5%, 1%, 0%]	263 [7%, 0%, 0%]	744 [19%, 0%, 0%]	0 [0%, 0%, 0%]	4,016 [1% of Total]
-150m to -200m	1,667 [55%, 3%, 0%]	469 [16%, 1%, 0%]	146 [5%, 0%, 0%]	162 [5%, 0%, 0%]	566 [19%, 0%, 0%]	0 [0%, 0%, 0%]	3,010 [0% of Total]
<-200m	18,457 [4%, 36%, 3%]	50,779 [10%, 72%, 8%]	28,050 [6%, 79%, 5%]	46,988 [9%, 75%, 8%]	350,667 [71%, 89%, 57%]	1,267 [0%, 100%, 0%]	496,207 [80% of Total]
Total	51,759 [8% of Total]	70,841 [11% of Total]	35,588 [6% of Total]	62,334 [10% of Total]	394,671 [64% of Total]	1,267 [0% of Total]	616,460 Km^2

The designations employed and the presentation of material in the map do not imply the expression of any opinion whatsoever on the part of FAO concerning the legal or constitutional status of any country, territory or sea area, or concerning the delimitation of frontiers.

Background reference map from National Geographic. Content may not reflect National Geographic's current map policy. Sources: National Geographic, Esri, DeLorme, HERE, UNEP-WCMC, USGS, NASA, ESA, METI, NRCAN, GEBCO, NOAA, increment P Corp.

Projection: Azimuthal Equidistant
Datum: WGS 1984
False Easting: 0.0000
False Northing: 0.0000
Central Meridian: -75.8710
Latitude Of Origin: 25.3730

Food and Agriculture Organization of the United Nations

Barbados: Caribbean Region

©FAO, 2018

Kilometers: 0 125 250 375 500

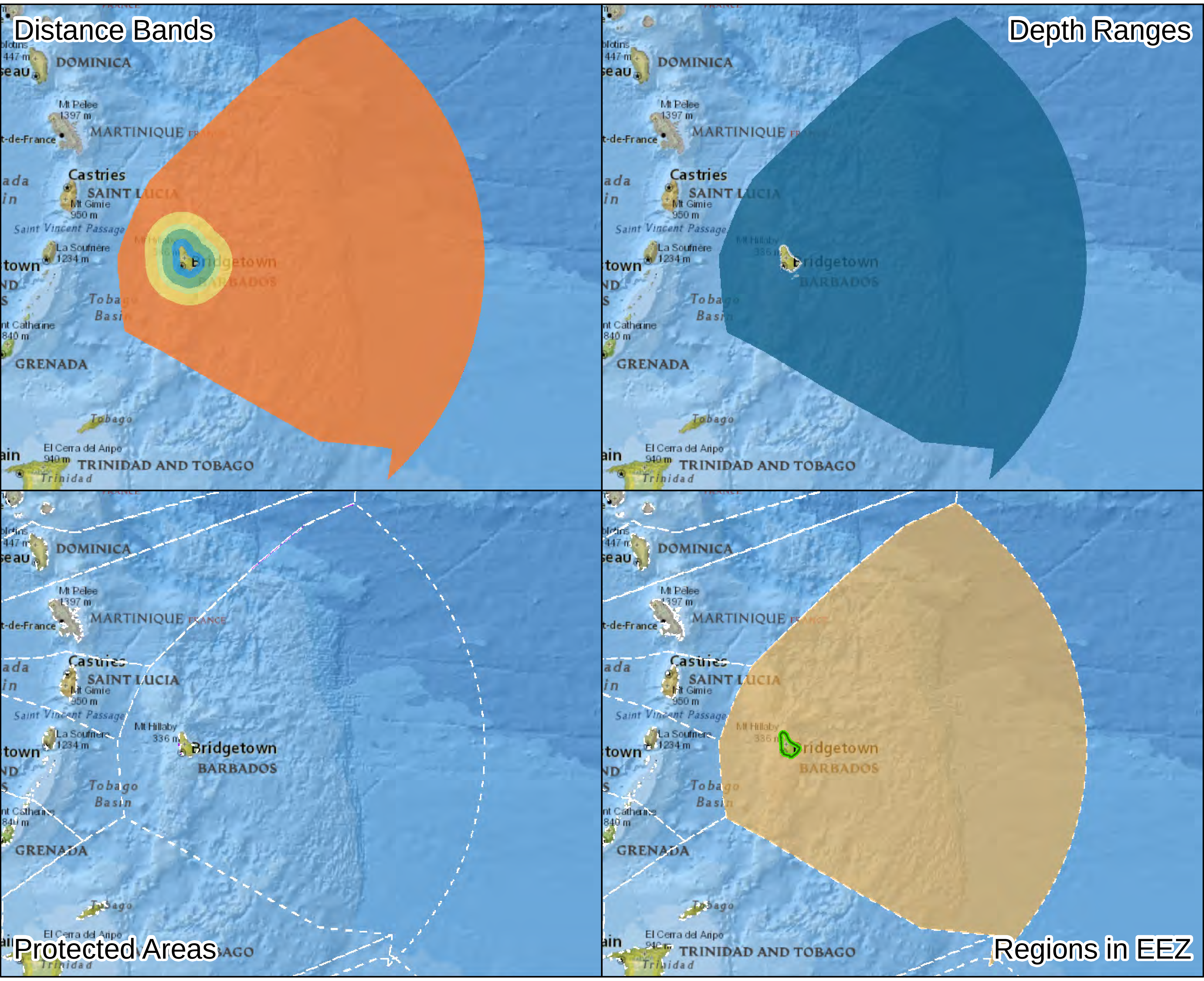

Distance Bands		Depth Bands		Protected	Region
0 to 5 NM	16 to 24 NM	> -10m	-100m to -150m	Protected Areas	Caribbean
5 to 12 NM	24 to 200 NM	-10m to -50m	-150m to -200m		
12 to 16 NM	> 200 NM	-50m to -100m	< -200m		

Coastline Length: 111 Km

All Areas in Barbados EEZ: Cell Values = Area in Km^2 [% Depth Band (Row), % Distance Band (Column), % EEZ]

	0 to 5 NM	5 to 12 NM	12 to 16 NM	16 to 24 NM	24 to 200 NM	> 200 NM	Total
> -10m	64 [100%, 6%, 0%]	0 [0%, 0%, 0%]	0 [0%, 0%, 0%]	0 [0%, 0%, 0%]	0 [0%, 0%, 0%]	0 [0%, 0%, 0%]	64 [0% of Total]
-10m to -50m	74 [100%, 7%, 0%]	0 [0%, 0%, 0%]	0 [0%, 0%, 0%]	0 [0%, 0%, 0%]	0 [0%, 0%, 0%]	0 [0%, 0%, 0%]	74 [0% of Total]
-50m to -100m	50 [96%, 5%, 0%]	2 [4%, 0%, 0%]	0 [0%, 0%, 0%]	0 [0%, 0%, 0%]	0 [0%, 0%, 0%]	0 [0%, 0%, 0%]	53 [0% of Total]
-100m to -150m	59 [89%, 5%, 0%]	7 [11%, 0%, 0%]	0 [0%, 0%, 0%]	0 [0%, 0%, 0%]	0 [0%, 0%, 0%]	0 [0%, 0%, 0%]	66 [0% of Total]
-150m to -200m	62 [84%, 6%, 0%]	12 [16%, 0%, 0%]	0 [0%, 0%, 0%]	0 [0%, 0%, 0%]	0 [0%, 0%, 0%]	0 [0%, 0%, 0%]	74 [0% of Total]
<-200m	804 [0%, 72%, 0%]	2,429 [1%, 99%, 1%]	1,873 [1%, 100%, 1%]	4,782 [3%, 100%, 3%]	175,521 [95%, 100%, 94%]	7 [0%, 100%, 0%]	185,416 [100% of Total]
Total	1,114 [1% of Total]	2,450 [1% of Total]	1,873 [1% of Total]	4,782 [3% of Total]	175,521 [94% of Total]	7 [0% of Total]	185,746 Km^2

Areas in Barbados EEZ Excluding Protected Areas: Cell Values = Area in Km^2 [% Depth Band (Row), % Distance Band (Column), % EEZ]

84 [0%] Km^2 Protected	0 to 5 NM	5 to 12 NM	12 to 16 NM	16 to 24 NM	24 to 200 NM	> 200 NM	Total
> -10m	62 [100%, 6%, 0%]	0 [0%, 0%, 0%]	0 [0%, 0%, 0%]	0 [0%, 0%, 0%]	0 [0%, 0%, 0%]	0 [0%, 0%, 0%]	62 [0% of Total]
-10m to -50m	69 [100%, 6%, 0%]	0 [0%, 0%, 0%]	0 [0%, 0%, 0%]	0 [0%, 0%, 0%]	0 [0%, 0%, 0%]	0 [0%, 0%, 0%]	69 [0% of Total]
-50m to -100m	50 [95%, 5%, 0%]	2 [5%, 0%, 0%]	0 [0%, 0%, 0%]	0 [0%, 0%, 0%]	0 [0%, 0%, 0%]	0 [0%, 0%, 0%]	52 [0% of Total]
-100m to -150m	56 [89%, 5%, 0%]	7 [11%, 0%, 0%]	0 [0%, 0%, 0%]	0 [0%, 0%, 0%]	0 [0%, 0%, 0%]	0 [0%, 0%, 0%]	63 [0% of Total]
-150m to -200m	60 [84%, 5%, 0%]	12 [16%, 0%, 0%]	0 [0%, 0%, 0%]	0 [0%, 0%, 0%]	0 [0%, 0%, 0%]	0 [0%, 0%, 0%]	72 [0% of Total]
<-200m	803 [0%, 73%, 0%]	2,429 [1%, 99%, 1%]	1,873 [1%, 100%, 1%]	4,782 [3%, 100%, 3%]	175,450 [95%, 100%, 94%]	7 [0%, 100%, 0%]	185,344 [100% of Total]
Total	1,101 [1% of Total]	2,450 [1% of Total]	1,873 [1% of Total]	4,782 [3% of Total]	175,450 [94% of Total]	7 [0% of Total]	185,662 Km^2

The designations employed and the presentation of material in the map do not imply the expression of any opinion whatsoever on the part of FAO concerning the legal or constitutional status of any country, territory or sea area, or concerning the delimitation of frontiers.

Background reference map from National Geographic. Content may not reflect National Geographic's current map policy. Sources: National Geographic, Esri, DeLorme, HERE, UNEP-WCMC, USGS, NASA, ESA, METI, NRCAN, GEBCO, NOAA, increment P Corp.

Projection: Azimuthal Equidistant
Datum: WGS 1984
False Easting: 0.0000
False Northing: 0.0000
Central Meridian: -58.1916
Latitude Of Origin: 13.3118

N W E S

Belgium: North Sea Region

Food and Agriculture Organization of the United Nations

©FAO, 2018

Kilometers
0 70

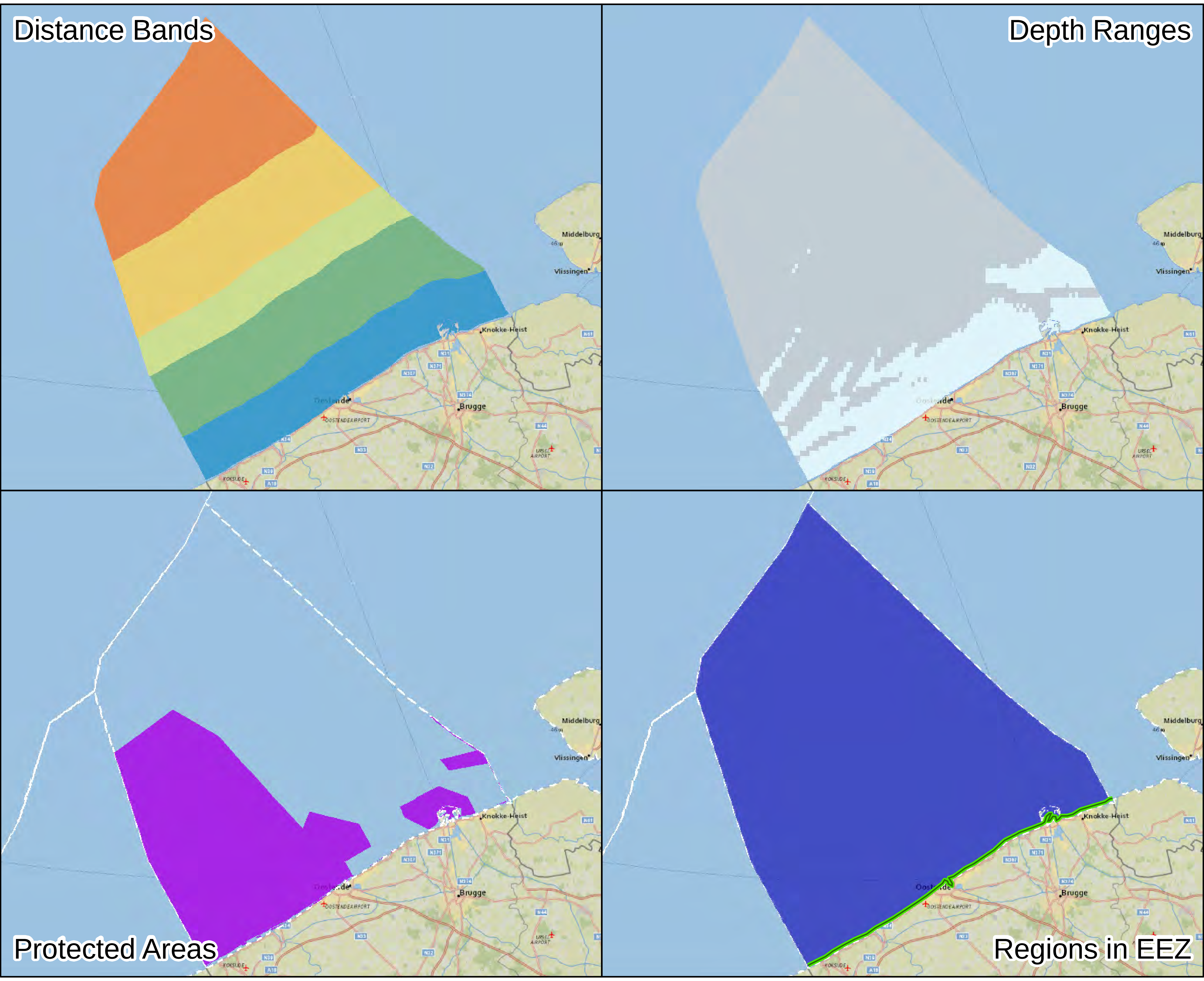

Distance Bands
- 0 to 5 NM
- 5 to 12 NM
- 12 to 16 NM
- 16 to 24 NM
- 24 to 200 NM

Depth Bands
- > -10m
- -10m to -50m

Protected
- Protected Areas

Coastline Length
- 78 Km

Region
- Baltic

All Areas in Belgium EEZ: Cell Values = Area in Km^2 [% Depth Band (Row), % Distance Band (Column), % EEZ]

	0 to 5 NM	5 to 12 NM	12 to 16 NM	16 to 24 NM	24 to 200 NM	> 200 NM	Total
> -10m	519 [75%, 85%, 15%]	166 [24%, 21%, 5%]	5 [1%, 1%, 0%]	2 [0%, 0%, 0%]	0 [0%, 0%, 0%]	0 [0%, 0%, 0%]	691 [20% of Total]
-10m to -50m	91 [3%, 15%, 3%]	637 [23%, 79%, 18%]	411 [15%, 99%, 12%]	745 [27%, 100%, 22%]	889 [32%, 100%, 26%]	0 [0%, 0%, 0%]	2,773 [80% of Total]
-50m to -100m	0 [0%, 0%, 0%]	0 [0%, 0%, 0%]	0 [0%, 0%, 0%]	0 [0%, 0%, 0%]	0 [0%, 0%, 0%]	0 [0%, 0%, 0%]	0.000 [0% of Total]
-100m to -150m	0 [0%, 0%, 0%]	0 [0%, 0%, 0%]	0 [0%, 0%, 0%]	0 [0%, 0%, 0%]	0 [0%, 0%, 0%]	0 [0%, 0%, 0%]	0.000 [0% of Total]
-150m to -200m	0 [0%, 0%, 0%]	0 [0%, 0%, 0%]	0 [0%, 0%, 0%]	0 [0%, 0%, 0%]	0 [0%, 0%, 0%]	0 [0%, 0%, 0%]	0.000 [0% of Total]
<-200m	0 [0%, 0%, 0%]	0 [0%, 0%, 0%]	0 [0%, 0%, 0%]	0 [0%, 0%, 0%]	0 [0%, 0%, 0%]	0 [0%, 0%, 0%]	0.000 [0% of Total]
Total	610 [18% of Total]	803 [23% of Total]	415 [12% of Total]	747 [22% of Total]	889 [26% of Total]	0.000 [0% of Total]	3,464 Km^2

Areas in Belgium EEZ Excluding Protected Areas: Cell Values = Area in Km^2 [% Depth Band (Row), % Distance Band (Column), % EEZ]

1,258 [36%] Km^2 Protected	0 to 5 NM	5 to 12 NM	12 to 16 NM	16 to 24 NM	24 to 200 NM	> 200 NM	Total
> -10m	177 [74%, 84%, 8%]	63 [26%, 16%, 3%]	0 [0%, 0%, 0%]	0 [0%, 0%, 0%]	0 [0%, 0%, 0%]	0 [0%, 0%, 0%]	241 [11% of Total]
-10m to -50m	35 [2%, 16%, 2%]	342 [17%, 84%, 16%]	234 [12%, 100%, 11%]	469 [24%, 100%, 21%]	886 [45%, 100%, 40%]	0 [0%, 0%, 0%]	1,965 [89% of Total]
-50m to -100m	0 [0%, 0%, 0%]	0 [0%, 0%, 0%]	0 [0%, 0%, 0%]	0 [0%, 0%, 0%]	0 [0%, 0%, 0%]	0 [0%, 0%, 0%]	0.000 [0% of Total]
-100m to -150m	0 [0%, 0%, 0%]	0 [0%, 0%, 0%]	0 [0%, 0%, 0%]	0 [0%, 0%, 0%]	0 [0%, 0%, 0%]	0 [0%, 0%, 0%]	0.000 [0% of Total]
-150m to -200m	0 [0%, 0%, 0%]	0 [0%, 0%, 0%]	0 [0%, 0%, 0%]	0 [0%, 0%, 0%]	0 [0%, 0%, 0%]	0 [0%, 0%, 0%]	0.000 [0% of Total]
<-200m	0 [0%, 0%, 0%]	0 [0%, 0%, 0%]	0 [0%, 0%, 0%]	0 [0%, 0%, 0%]	0 [0%, 0%, 0%]	0 [0%, 0%, 0%]	0.000 [0% of Total]
Total	212 [10% of Total]	405 [18% of Total]	234 [11% of Total]	469 [21% of Total]	886 [40% of Total]	0.000 [0% of Total]	2,206 Km^2

The designations employed and the presentation of material in the map do not imply the expression of any opinion whatsoever on the part of FAO concerning the legal or constitutional status of any country, territory or sea area, or concerning the delimitation of frontiers.

Background reference map from National Geographic. Content may not reflect National Geographic's current map policy. Sources: National Geographic, Esri, DeLorme, HERE, UNEP-WCMC, USGS, NASA, ESA, METI, NRCAN, GEBCO, NOAA, increment P Corp.

Projection: Azimuthal Equidistant
Datum: WGS 1984
False Easting: 0.0000
False Northing: 0.0000
Central Meridian: 2.8012
Latitude Of Origin: 51.4841

Belize: Caribbean Region

©FAO, 2018

Food and Agriculture Organization of the United Nations

Kilometers
0 125

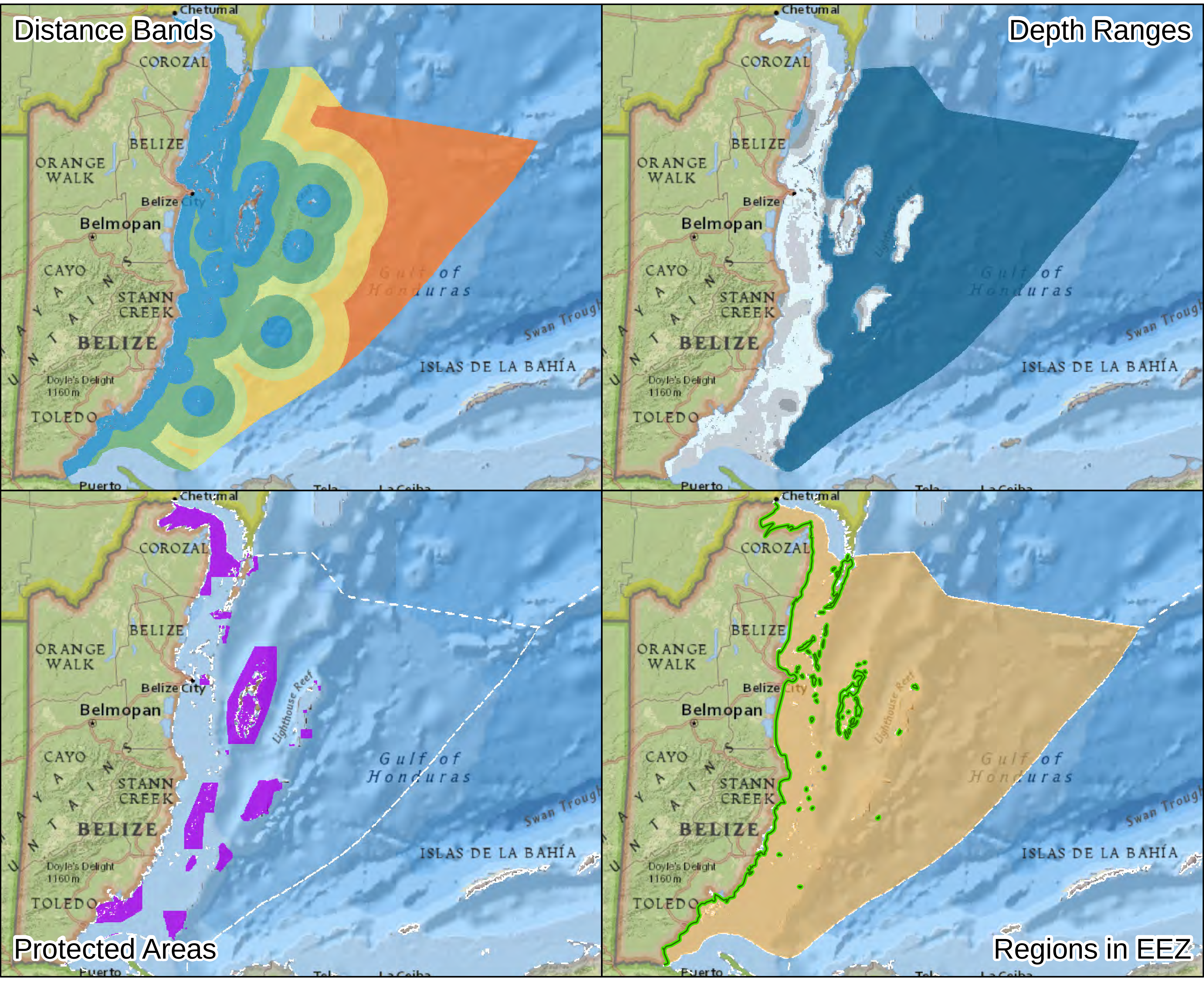

Distance Bands

- 0 to 5 NM
- 5 to 12 NM
- 12 to 16 NM
- 16 to 24 NM
- 24 to 200 NM

Depth Bands

- > -10m
- -10m to -50m
- -50m to -100m
- -100m to -150m
- -150m to -200m
- < -200m

Protected

- Protected Areas

Region

- Caribbean

Coastline Length

977 Km

All Areas in Belize EEZ: Cell Values = Area in Km^2 [% Depth Band (Row), % Distance Band (Column), % EEZ]

	0 to 5 NM	5 to 12 NM	12 to 16 NM	16 to 24 NM	24 to 200 NM	> 200 NM	Total
> -10m	3,929 [79%, 42%, 12%]	907 [18%, 11%, 3%]	130 [3%, 4%, 0%]	0 [0%, 0%, 0%]	0 [0%, 0%, 0%]	0 [0%, 0%, 0%]	4,966 [15% of Total]
-10m to -50m	2,192 [63%, 24%, 6%]	1,096 [32%, 14%, 3%]	162 [5%, 5%, 0%]	3 [0%, 0%, 0%]	0 [0%, 0%, 0%]	0 [0%, 0%, 0%]	3,453 [10% of Total]
-50m to -100m	550 [74%, 6%, 2%]	131 [18%, 2%, 0%]	56 [8%, 2%, 0%]	9 [1%, 0%, 0%]	0 [0%, 0%, 0%]	0 [0%, 0%, 0%]	746 [2% of Total]
-100m to -150m	321 [73%, 3%, 1%]	80 [18%, 1%, 0%]	34 [8%, 1%, 0%]	2 [0%, 0%, 0%]	0 [0%, 0%, 0%]	0 [0%, 0%, 0%]	437 [1% of Total]
-150m to -200m	258 [69%, 3%, 1%]	78 [21%, 1%, 0%]	38 [10%, 1%, 0%]	1 [0%, 0%, 0%]	0 [0%, 0%, 0%]	0 [0%, 0%, 0%]	375 [1% of Total]
<-200m	2,019 [8%, 22%, 6%]	5,817 [24%, 72%, 17%]	3,175 [13%, 88%, 9%]	4,434 [18%, 100%, 13%]	8,522 [36%, 100%, 25%]	0 [0%, 0%, 0%]	23,966 [71% of Total]
Total	9,269 [27% of Total]	8,109 [24% of Total]	3,595 [11% of Total]	4,449 [13% of Total]	8,522 [25% of Total]	0.000 [0% of Total]	33,944 Km^2

Areas in Belize EEZ Excluding Protected Areas: Cell Values = Area in Km^2 [% Depth Band (Row), % Distance Band (Column), % EEZ]

3,662 [11%] Km^2 Protected	0 to 5 NM	5 to 12 NM	12 to 16 NM	16 to 24 NM	24 to 200 NM	> 200 NM	Total
> -10m	2,426 [79%, 38%, 8%]	591 [19%, 8%, 2%]	56 [2%, 2%, 0%]	0 [0%, 0%, 0%]	0 [0%, 0%, 0%]	0 [0%, 0%, 0%]	3,073 [10% of Total]
-10m to -50m	1,548 [59%, 24%, 5%]	1,004 [38%, 13%, 3%]	90 [3%, 3%, 0%]	0.294 [0%, 0%, 0%]	0 [0%, 0%, 0%]	0 [0%, 0%, 0%]	2,642 [9% of Total]
-50m to -100m	396 [76%, 6%, 1%]	88 [17%, 1%, 0%]	31 [6%, 1%, 0%]	3 [1%, 0%, 0%]	0 [0%, 0%, 0%]	0 [0%, 0%, 0%]	519 [2% of Total]
-100m to -150m	231 [75%, 4%, 1%]	49 [16%, 1%, 0%]	27 [9%, 1%, 0%]	2 [1%, 0%, 0%]	0 [0%, 0%, 0%]	0 [0%, 0%, 0%]	309 [1% of Total]
-150m to -200m	182 [69%, 3%, 1%]	49 [19%, 1%, 0%]	31 [12%, 1%, 0%]	1 [0%, 0%, 0%]	0 [0%, 0%, 0%]	0 [0%, 0%, 0%]	263 [1% of Total]
<-200m	1,680 [7%, 26%, 6%]	5,707 [24%, 76%, 19%]	3,135 [13%, 93%, 10%]	4,434 [19%, 100%, 15%]	8,522 [36%, 100%, 28%]	0 [0%, 0%, 0%]	23,477 [78% of Total]
Total	6,463 [21% of Total]	7,488 [25% of Total]	3,370 [11% of Total]	4,440 [15% of Total]	8,522 [28% of Total]	0.000 [0% of Total]	30,282 Km^2

The designations employed and the presentation of material in the map do not imply the expression of any opinion whatsoever on the part of FAO concerning the legal or constitutional status of any country, territory or sea area, or concerning the delimitation of frontiers.

Background reference map from National Geographic. Content may not reflect National Geographic's current map policy. Sources: National Geographic, Esri, DeLorme, HERE, UNEP-WCMC, USGS, NASA, ESA, METI, NRCAN, GEBCO, NOAA, increment P Corp.

Projection: Azimuthal Equidistant
Datum: WGS 1984
False Easting: 0.0000
False Northing: 0.0000
Central Meridian: -87.5555
Latitude Of Origin: 17.1856

N W E S

Bosnia and Herzegovina: Mediterranean Region

Food and Agriculture Organization of the United Nations

©FAO, 2018

Kilometers
0 5.5

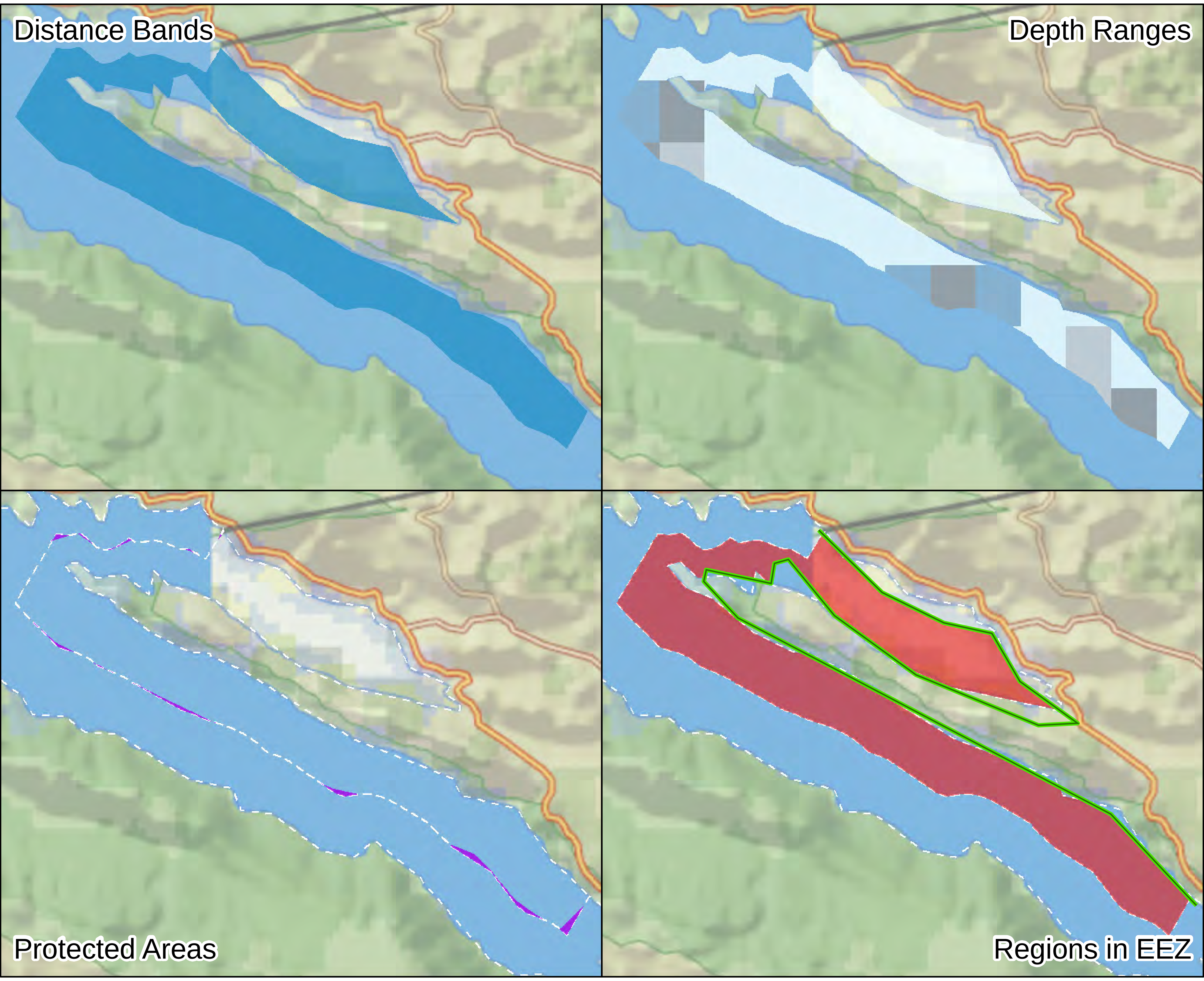

Distance Bands
0 to 5 NM

Depth Bands
> -10m
-10m to -50m
-50m to -100m
-100m to -150m

Protected
Protected Areas

Coastline Length
21 Km

Region
Mediterranean

All Areas in Bosnia and Herzegovina EEZ: Cell Values = Area in Km^2 [% Depth Band (Row), % Distance Band (Column), % EEZ]

	0 to 5 NM	5 to 12 NM	12 to 16 NM	16 to 24 NM	24 to 200 NM	> 200 NM	Total
> -10m	8 [100%, 71%, 71%]	0 [0%, 0%, 0%]	0 [0%, 0%, 0%]	0 [0%, 0%, 0%]	0 [0%, 0%, 0%]	0 [0%, 0%, 0%]	8 [71% of Total]
-10m to -50m	0.877 [100%, 7%, 7%]	0 [0%, 0%, 0%]	0 [0%, 0%, 0%]	0 [0%, 0%, 0%]	0 [0%, 0%, 0%]	0 [0%, 0%, 0%]	0.877 [7% of Total]
-50m to -100m	1 [100%, 12%, 12%]	0 [0%, 0%, 0%]	0 [0%, 0%, 0%]	0 [0%, 0%, 0%]	0 [0%, 0%, 0%]	0 [0%, 0%, 0%]	1 [12% of Total]
-100m to -150m	1 [100%, 10%, 10%]	0 [0%, 0%, 0%]	0 [0%, 0%, 0%]	0 [0%, 0%, 0%]	0 [0%, 0%, 0%]	0 [0%, 0%, 0%]	1 [10% of Total]
-150m to -200m	0 [0%, 0%, 0%]	0 [0%, 0%, 0%]	0 [0%, 0%, 0%]	0 [0%, 0%, 0%]	0 [0%, 0%, 0%]	0 [0%, 0%, 0%]	0.000 [0% of Total]
<-200m	0 [0%, 0%, 0%]	0 [0%, 0%, 0%]	0 [0%, 0%, 0%]	0 [0%, 0%, 0%]	0 [0%, 0%, 0%]	0 [0%, 0%, 0%]	0.000 [0% of Total]
Total	12 [100% of Total]	0.000 [0% of Total]	0.000 [0% of Total]	0.000 [0% of Total]	0.000 [0% of Total]	0.000 [0% of Total]	12 Km^2

Areas in Bosnia and Herzegovina EEZ Excluding Protected Areas: Cell Values = Area in Km^2 [% Depth Band (Row), % Distance Band (Column), % EEZ]

0 [2%] Km^2 Protected	0 to 5 NM	5 to 12 NM	12 to 16 NM	16 to 24 NM	24 to 200 NM	> 200 NM	Total
> -10m	8 [100%, 72%, 72%]	0 [0%, 0%, 0%]	0 [0%, 0%, 0%]	0 [0%, 0%, 0%]	0 [0%, 0%, 0%]	0 [0%, 0%, 0%]	8 [72% of Total]
-10m to -50m	0.830 [100%, 7%, 7%]	0 [0%, 0%, 0%]	0 [0%, 0%, 0%]	0 [0%, 0%, 0%]	0 [0%, 0%, 0%]	0 [0%, 0%, 0%]	0.830 [7% of Total]
-50m to -100m	1 [100%, 11%, 11%]	0 [0%, 0%, 0%]	0 [0%, 0%, 0%]	0 [0%, 0%, 0%]	0 [0%, 0%, 0%]	0 [0%, 0%, 0%]	1 [11% of Total]
-100m to -150m	1 [100%, 10%, 10%]	0 [0%, 0%, 0%]	0 [0%, 0%, 0%]	0 [0%, 0%, 0%]	0 [0%, 0%, 0%]	0 [0%, 0%, 0%]	1 [10% of Total]
-150m to -200m	0 [0%, 0%, 0%]	0 [0%, 0%, 0%]	0 [0%, 0%, 0%]	0 [0%, 0%, 0%]	0 [0%, 0%, 0%]	0 [0%, 0%, 0%]	0.000 [0% of Total]
<-200m	0 [0%, 0%, 0%]	0 [0%, 0%, 0%]	0 [0%, 0%, 0%]	0 [0%, 0%, 0%]	0 [0%, 0%, 0%]	0 [0%, 0%, 0%]	0.000 [0% of Total]
Total	12 [100% of Total]	0.000 [0% of Total]	0.000 [0% of Total]	0.000 [0% of Total]	0.000 [0% of Total]	0.000 [0% of Total]	12 Km^2

The designations employed and the presentation of material in the map do not imply the expression of any opinion whatsoever on the part of FAO concerning the legal or constitutional status of any country, territory or sea area, or concerning the delimitation of frontiers.

Background reference map from National Geographic. Content may not reflect National Geographic's current map policy. Sources: National Geographic, Esri, DeLorme, HERE, UNEP-WCMC, USGS, NASA, ESA, METI, NRCAN, GEBCO, NOAA, increment P Corp.

Projection: Azimuthal Equidistant
Datum: WGS 1984
False Easting: 0.0000
False Northing: 0.0000
Central Meridian: 17.5949
Latitude Of Origin: 42.9107

N W E S

Bulgaria: Black Sea Region

Food and Agriculture Organization of the United Nations

©FAO, 2018

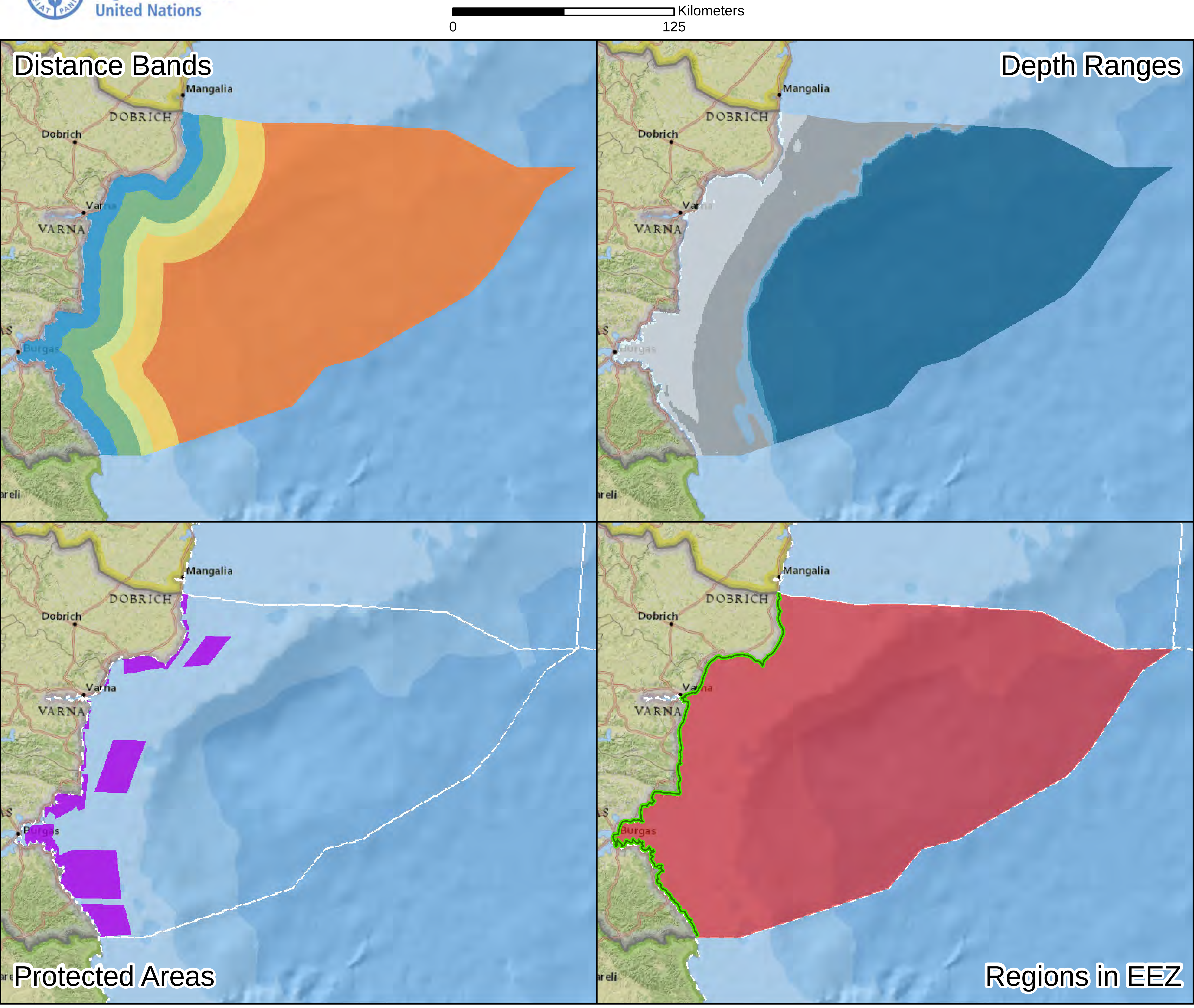

Distance Bands
- 0 to 5 NM
- 5 to 12 NM
- 12 to 16 NM
- 16 to 24 NM
- 24 to 200 NM

Depth Bands
- > -10m
- -10m to -50m
- -50m to -100m
- -100m to -150m
- -150m to -200m
- < -200m

Protected
- Protected Areas

Coastline Length
- 360 Km

Region
- Mediterranean

All Areas in Bulgaria EEZ: Cell Values = Area in Km^2 [% Depth Band (Row), % Distance Band (Column), % EEZ]

	0 to 5 NM	5 to 12 NM	12 to 16 NM	16 to 24 NM	24 to 200 NM	> 200 NM	Total
> -10m	213 [100%, 8%, 1%]	0 [0%, 0%, 0%]	0 [0%, 0%, 0%]	0 [0%, 0%, 0%]	0 [0%, 0%, 0%]	0 [0%, 0%, 0%]	213 [1% of Total]
-10m to -50m	2,084 [53%, 77%, 6%]	1,657 [42%, 50%, 5%]	208 [5%, 12%, 1%]	6 [0%, 0%, 0%]	0 [0%, 0%, 0%]	0 [0%, 0%, 0%]	3,955 [11% of Total]
-50m to -100m	395 [6%, 15%, 1%]	1,654 [27%, 50%, 5%]	1,515 [25%, 85%, 4%]	2,067 [34%, 61%, 6%]	484 [8%, 2%, 1%]	0 [0%, 0%, 0%]	6,116 [18% of Total]
-100m to -150m	0 [0%, 0%, 0%]	0 [0%, 0%, 0%]	52 [5%, 3%, 0%]	657 [67%, 20%, 2%]	268 [27%, 1%, 1%]	0 [0%, 0%, 0%]	976 [3% of Total]
-150m to -200m	0 [0%, 0%, 0%]	0 [0%, 0%, 0%]	0 [0%, 0%, 0%]	119 [32%, 4%, 0%]	249 [68%, 1%, 1%]	0 [0%, 0%, 0%]	368 [1% of Total]
<-200m	0 [0%, 0%, 0%]	0 [0%, 0%, 0%]	0 [0%, 0%, 0%]	513 [2%, 15%, 1%]	22,384 [98%, 96%, 65%]	0 [0%, 0%, 0%]	22,897 [66% of Total]
Total	2,693 [8% of Total]	3,311 [10% of Total]	1,775 [5% of Total]	3,363 [10% of Total]	23,385 [68% of Total]	0.000 [0% of Total]	34,526 Km^2

Areas in Bulgaria EEZ Excluding Protected Areas: Cell Values = Area in Km^2 [% Depth Band (Row), % Distance Band (Column), % EEZ]

2,693 [8%] Km^2 Protected	0 to 5 NM	5 to 12 NM	12 to 16 NM	16 to 24 NM	24 to 200 NM	> 200 NM	Total
> -10m	73 [100%, 5%, 0%]	0 [0%, 0%, 0%]	0 [0%, 0%, 0%]	0 [0%, 0%, 0%]	0 [0%, 0%, 0%]	0 [0%, 0%, 0%]	73 [0% of Total]
-10m to -50m	1,190 [46%, 83%, 4%]	1,202 [46%, 54%, 4%]	189 [7%, 13%, 1%]	6 [0%, 0%, 0%]	0 [0%, 0%, 0%]	0 [0%, 0%, 0%]	2,588 [8% of Total]
-50m to -100m	179 [4%, 12%, 1%]	1,006 [20%, 46%, 3%]	1,226 [25%, 84%, 4%]	2,036 [41%, 61%, 6%]	484 [10%, 2%, 2%]	0 [0%, 0%, 0%]	4,931 [15% of Total]
-100m to -150m	0 [0%, 0%, 0%]	0 [0%, 0%, 0%]	52 [5%, 4%, 0%]	657 [67%, 20%, 2%]	268 [27%, 1%, 1%]	0 [0%, 0%, 0%]	976 [3% of Total]
-150m to -200m	0 [0%, 0%, 0%]	0 [0%, 0%, 0%]	0 [0%, 0%, 0%]	119 [32%, 4%, 0%]	249 [68%, 1%, 1%]	0 [0%, 0%, 0%]	368 [1% of Total]
<-200m	0 [0%, 0%, 0%]	0 [0%, 0%, 0%]	0 [0%, 0%, 0%]	513 [2%, 15%, 2%]	22,384 [98%, 96%, 70%]	0 [0%, 0%, 0%]	22,897 [72% of Total]
Total	1,442 [5% of Total]	2,209 [7% of Total]	1,467 [5% of Total]	3,331 [10% of Total]	23,385 [73% of Total]	0.000 [0% of Total]	31,834 Km^2

The designations employed and the presentation of material in the map do not imply the expression of any opinion whatsoever on the part of FAO concerning the legal or constitutional status of any country, territory or sea area, or concerning the delimitation of frontiers.

Background reference map from National Geographic. Content may not reflect National Geographic's current map policy. Sources: National Geographic, Esri, DeLorme, HERE, UNEP-WCMC, USGS, NASA, ESA, METI, NRCAN, GEBCO, NOAA, increment P Corp.

Projection: Azimuthal Equidistant
Datum: WGS 1984
False Easting: 0.0000
False Northing: 0.0000
Central Meridian: 29.3930
Latitude Of Origin: 42.8578

Colombia + Joint Regime: Caribbean Region

©FAO, 2018

Food and Agriculture Organization of the United Nations

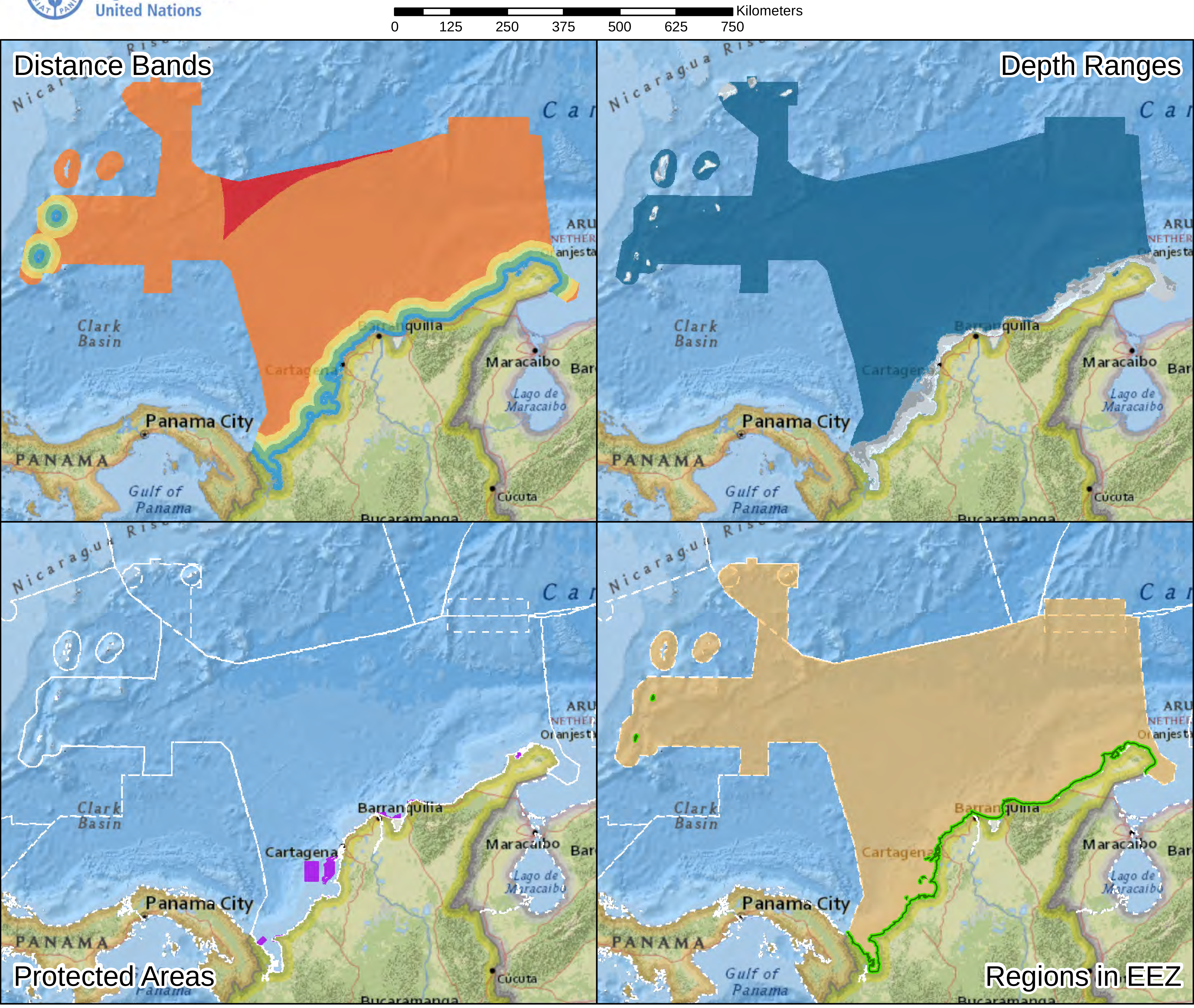

Distance Bands

- 0 to 5 NM
- 5 to 12 NM
- 12 to 16 NM
- 16 to 24 NM
- 24 to 200 NM
- > 200 NM

Depth Bands

- > -10m
- -10m to -50m
- -50m to -100m
- -100m to -150m
- -150m to -200m
- < -200m

Protected

- Protected Areas

Region

- Caribbean

Coastline Length

1,696 Km

All Areas in Colombia + Joint Regime EEZ: Cell Values = Area in Km^2 [% Depth Band (Row), % Distance Band (Column), % EEZ]

	0 to 5 NM	5 to 12 NM	12 to 16 NM	16 to 24 NM	24 to 200 NM	> 200 NM	Total
> -10m	3,604 [72%, 26%, 1%]	318 [6%, 2%, 0%]	34 [1%, 0%, 0%]	102 [2%, 0%, 0%]	937 [19%, 0%, 0%]	0 [0%, 0%, 0%]	4,995 [1% of Total]
-10m to -50m	6,754 [45%, 48%, 2%]	5,337 [36%, 29%, 1%]	715 [5%, 7%, 0%]	682 [5%, 3%, 0%]	1,492 [10%, 0%, 0%]	0 [0%, 0%, 0%]	14,981 [4% of Total]
-50m to -100m	1,828 [20%, 13%, 0%]	3,927 [43%, 22%, 1%]	1,846 [20%, 18%, 0%]	901 [10%, 4%, 0%]	707 [8%, 0%, 0%]	0 [0%, 0%, 0%]	9,209 [2% of Total]
-100m to -150m	419 [16%, 3%, 0%]	823 [32%, 5%, 0%]	538 [21%, 5%, 0%]	410 [16%, 2%, 0%]	381 [15%, 0%, 0%]	0 [0%, 0%, 0%]	2,571 [1% of Total]
-150m to -200m	302 [16%, 2%, 0%]	621 [33%, 3%, 0%]	202 [11%, 2%, 0%]	380 [20%, 2%, 0%]	389 [21%, 0%, 0%]	0 [0%, 0%, 0%]	1,894 [0% of Total]
<-200m	1,122 [0%, 8%, 0%]	7,223 [2%, 40%, 2%]	6,938 [2%, 68%, 2%]	18,577 [5%, 88%, 4%]	340,832 [88%, 99%, 81%]	14,320 [4%, 100%, 3%]	389,013 [92% of Total]
Total	14,027 [3% of Total]	18,249 [4% of Total]	10,274 [2% of Total]	21,053 [5% of Total]	344,738 [82% of Total]	14,320 [3% of Total]	422,662 Km^2

Areas in Colombia + Joint Regime EEZ Excluding Protected Areas: Cell Values = Area in Km^2 [% Depth Band (Row), % Distance Band (Column), % EEZ]

3,404 [1%] Km^2 Protected	0 to 5 NM	5 to 12 NM	12 to 16 NM	16 to 24 NM	24 to 200 NM	> 200 NM	Total
> -10m	3,344 [71%, 26%, 1%]	305 [6%, 2%, 0%]	34 [1%, 0%, 0%]	102 [2%, 1%, 0%]	937 [20%, 0%, 0%]	0 [0%, 0%, 0%]	4,722 [1% of Total]
-10m to -50m	6,196 [43%, 49%, 1%]	5,258 [37%, 30%, 1%]	715 [5%, 7%, 0%]	679 [5%, 3%, 0%]	1,492 [10%, 0%, 0%]	0 [0%, 0%, 0%]	14,341 [3% of Total]
-50m to -100m	1,487 [19%, 12%, 0%]	3,246 [41%, 19%, 1%]	1,699 [21%, 17%, 0%]	826 [10%, 4%, 0%]	707 [9%, 0%, 0%]	0 [0%, 0%, 0%]	7,965 [2% of Total]
-100m to -150m	367 [17%, 3%, 0%]	737 [33%, 4%, 0%]	396 [18%, 4%, 0%]	322 [15%, 2%, 0%]	381 [17%, 0%, 0%]	0 [0%, 0%, 0%]	2,203 [1% of Total]
-150m to -200m	278 [16%, 2%, 0%]	621 [35%, 4%, 0%]	194 [11%, 2%, 0%]	311 [17%, 2%, 0%]	389 [22%, 0%, 0%]	0 [0%, 0%, 0%]	1,794 [0% of Total]
<-200m	1,051 [0%, 8%, 0%]	7,223 [2%, 42%, 2%]	6,925 [2%, 70%, 2%]	18,094 [5%, 89%, 4%]	340,619 [88%, 99%, 81%]	14,320 [4%, 100%, 3%]	388,233 [93% of Total]
Total	12,724 [3% of Total]	17,391 [4% of Total]	9,963 [2% of Total]	20,335 [5% of Total]	344,525 [82% of Total]	14,320 [3% of Total]	419,258 Km^2

The designations employed and the presentation of material in the map do not imply the expression of any opinion whatsoever on the part of FAO concerning the legal or constitutional status of any country, territory or sea area, or concerning the delimitation of frontiers.

Background reference map from National Geographic. Content may not reflect National Geographic's current map policy. Sources: National Geographic, Esri, DeLorme, HERE, UNEP-WCMC, USGS, NASA, ESA, METI, NRCAN, GEBCO, NOAA, increment P Corp.

Projection: Azimuthal Equidistant
Datum: WGS 1984
False Easting: 0.0000
False Northing: 0.0000
Central Meridian: -76.4112
Latitude Of Origin: 12.0351

Colombia: Caribbean Region

©FAO, 2018

Food and Agriculture Organization of the United Nations

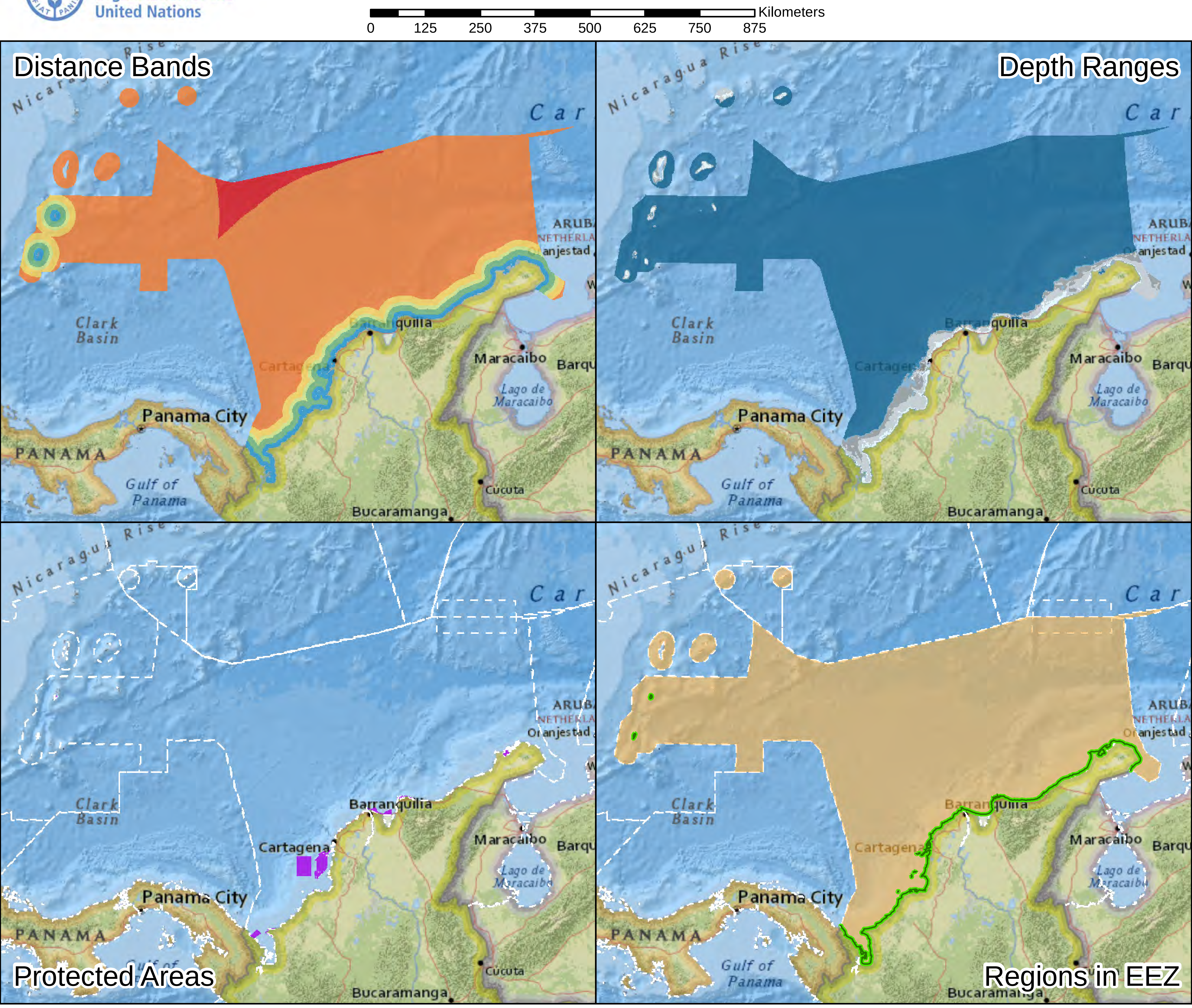

Distance Bands

- 0 to 5 NM
- 5 to 12 NM
- 12 to 16 NM
- 16 to 24 NM
- 24 to 200 NM
- > 200 NM

Depth Bands

- > -10m
- -10m to -50m
- -50m to -100m
- -100m to -150m
- -150m to -200m
- < -200m

Protected

- Protected Areas

Coastline Length

- 1,696 Km

Region

- Caribbean

All Areas in Colombia EEZ: Cell Values = Area in Km^2 [% Depth Band (Row), % Distance Band (Column), % EEZ]

	0 to 5 NM	5 to 12 NM	12 to 16 NM	16 to 24 NM	24 to 200 NM	> 200 NM	Total
> -10m	3,604 [72%, 26%, 1%]	318 [6%, 2%, 0%]	34 [1%, 0%, 0%]	102 [2%, 0%, 0%]	936 [19%, 0%, 0%]	0 [0%, 0%, 0%]	4,995 [1% of Total]
-10m to -50m	6,754 [46%, 48%, 2%]	5,337 [36%, 29%, 1%]	715 [5%, 7%, 0%]	682 [5%, 3%, 0%]	1,319 [9%, 0%, 0%]	0 [0%, 0%, 0%]	14,808 [4% of Total]
-50m to -100m	1,828 [20%, 13%, 0%]	3,927 [44%, 22%, 1%]	1,846 [21%, 18%, 0%]	901 [10%, 4%, 0%]	497 [6%, 0%, 0%]	0 [0%, 0%, 0%]	8,999 [2% of Total]
-100m to -150m	419 [17%, 3%, 0%]	823 [33%, 5%, 0%]	538 [22%, 5%, 0%]	410 [16%, 2%, 0%]	312 [12%, 0%, 0%]	0 [0%, 0%, 0%]	2,502 [1% of Total]
-150m to -200m	302 [17%, 2%, 0%]	621 [34%, 3%, 0%]	202 [11%, 2%, 0%]	380 [21%, 2%, 0%]	315 [17%, 0%, 0%]	0 [0%, 0%, 0%]	1,820 [0% of Total]
<-200m	1,122 [0%, 8%, 0%]	7,223 [2%, 40%, 2%]	6,938 [2%, 68%, 2%]	18,577 [5%, 88%, 5%]	320,407 [87%, 99%, 80%]	14,320 [4%, 100%, 4%]	368,588 [92% of Total]
Total	14,027 [3% of Total]	18,249 [5% of Total]	10,274 [3% of Total]	21,053 [5% of Total]	323,787 [81% of Total]	14,320 [4% of Total]	401,711 Km^2

Areas in Colombia EEZ Excluding Protected Areas: Cell Values = Area in Km^2 [% Depth Band (Row), % Distance Band (Column), % EEZ]

3,404 [1%] Km^2 Protected	0 to 5 NM	5 to 12 NM	12 to 16 NM	16 to 24 NM	24 to 200 NM	> 200 NM	Total
> -10m	3,344 [71%, 26%, 1%]	305 [6%, 2%, 0%]	34 [1%, 0%, 0%]	102 [2%, 1%, 0%]	936 [20%, 0%, 0%]	0 [0%, 0%, 0%]	4,722 [1% of Total]
-10m to -50m	6,196 [44%, 49%, 2%]	5,258 [37%, 30%, 1%]	715 [5%, 7%, 0%]	679 [5%, 3%, 0%]	1,319 [9%, 0%, 0%]	0 [0%, 0%, 0%]	14,168 [4% of Total]
-50m to -100m	1,487 [19%, 12%, 0%]	3,246 [42%, 19%, 1%]	1,699 [22%, 17%, 0%]	826 [11%, 4%, 0%]	497 [6%, 0%, 0%]	0 [0%, 0%, 0%]	7,755 [2% of Total]
-100m to -150m	367 [17%, 3%, 0%]	737 [35%, 4%, 0%]	396 [19%, 4%, 0%]	322 [15%, 2%, 0%]	312 [15%, 0%, 0%]	0 [0%, 0%, 0%]	2,134 [1% of Total]
-150m to -200m	278 [16%, 2%, 0%]	621 [36%, 4%, 0%]	194 [11%, 2%, 0%]	311 [18%, 2%, 0%]	315 [18%, 0%, 0%]	0 [0%, 0%, 0%]	1,720 [0% of Total]
<-200m	1,051 [0%, 8%, 0%]	7,223 [2%, 42%, 2%]	6,925 [2%, 70%, 2%]	18,094 [5%, 89%, 5%]	320,193 [87%, 99%, 80%]	14,320 [4%, 100%, 4%]	367,807 [92% of Total]
Total	12,724 [3% of Total]	17,391 [4% of Total]	9,963 [3% of Total]	20,335 [5% of Total]	323,573 [81% of Total]	14,320 [4% of Total]	398,307 Km^2

The designations employed and the presentation of material in the map do not imply the expression of any opinion whatsoever on the part of FAO concerning the legal or constitutional status of any country, territory or sea area, or concerning the delimitation of frontiers.

Background reference map from National Geographic. Content may not reflect National Geographic's current map policy. Sources: National Geographic, Esri, DeLorme, HERE, UNEP-WCMC, USGS, NASA, ESA, METI, NRCAN, GEBCO, NOAA, increment P Corp.

Projection: Azimuthal Equidistant
Datum: WGS 1984
False Easting: 0.0000
False Northing: 0.0000
Central Meridian: -76.2709
Latitude Of Origin: 11.9758

Food and Agriculture Organization of the United Nations

Costa Rica: Caribbean Region

©FAO, 2018

Kilometers
0 125

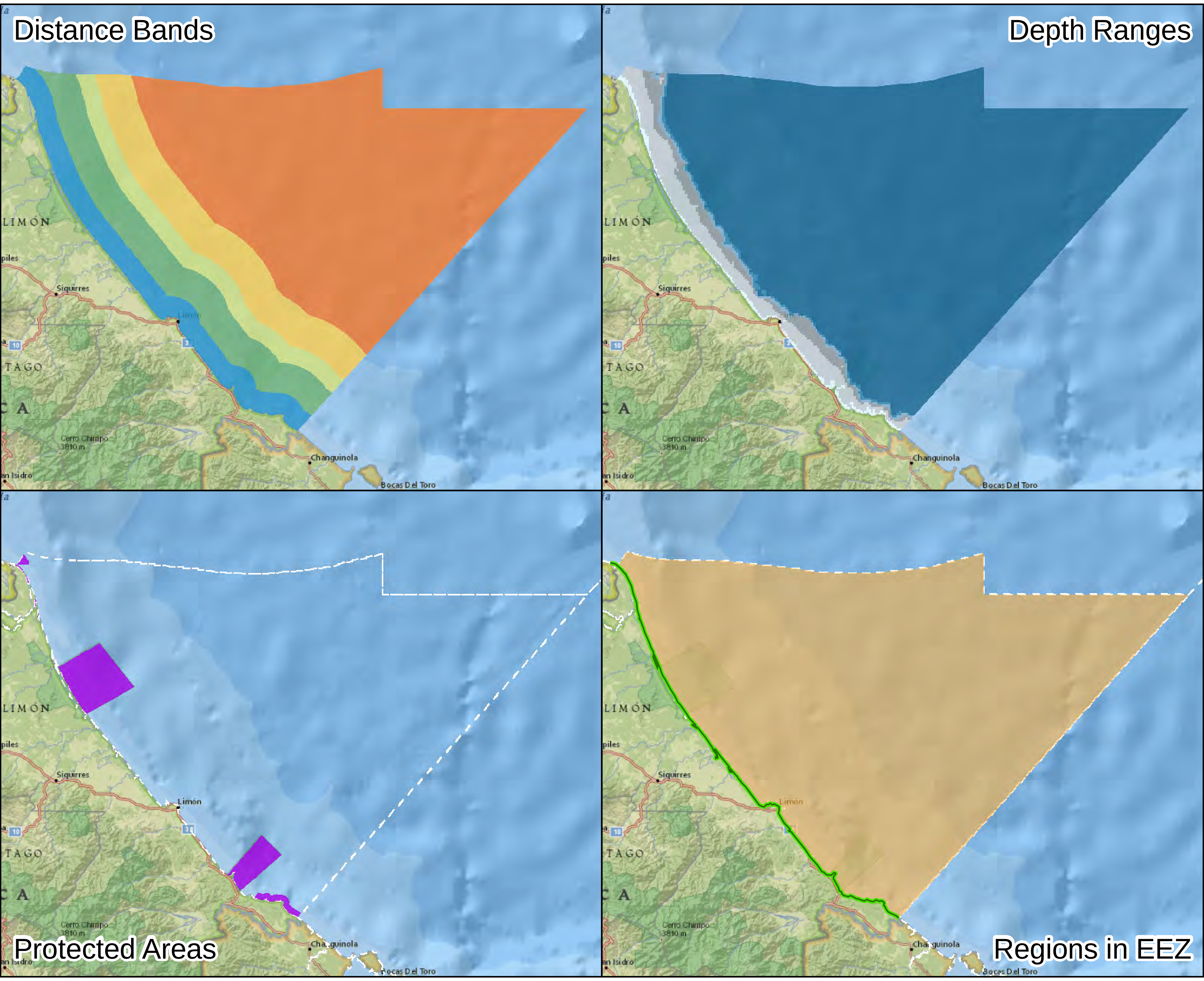

Distance Bands

- 0 to 5 NM
- 5 to 12 NM
- 12 to 16 NM
- 16 to 24 NM
- 24 to 200 NM

Depth Bands

- > -10m
- -10m to -50m
- -50m to -100m
- -100m to -150m
- -150m to -200m
- < -200m

Protected

- Protected Areas

Coastline Length

244 Km

Region

- Caribbean

All Areas in Costa Rica EEZ: Cell Values = Area in Km^2 [% Depth Band (Row), % Distance Band (Column), % EEZ]

	0 to 5 NM	5 to 12 NM	12 to 16 NM	16 to 24 NM	24 to 200 NM	> 200 NM	Total
> -10m	197 [100%, 11%, 1%]	0 [0%, 0%, 0%]	0 [0%, 0%, 0%]	0 [0%, 0%, 0%]	0 [0%, 0%, 0%]	0 [0%, 0%, 0%]	197 [1% of Total]
-10m to -50m	1,060 [99%, 58%, 5%]	12 [1%, 0%, 0%]	0 [0%, 0%, 0%]	0 [0%, 0%, 0%]	0 [0%, 0%, 0%]	0 [0%, 0%, 0%]	1,072 [5% of Total]
-50m to -100m	305 [55%, 17%, 2%]	245 [45%, 10%, 1%]	0 [0%, 0%, 0%]	0 [0%, 0%, 0%]	0 [0%, 0%, 0%]	0 [0%, 0%, 0%]	550 [3% of Total]
-100m to -150m	81 [40%, 4%, 0%]	124 [60%, 5%, 1%]	0 [0%, 0%, 0%]	0 [0%, 0%, 0%]	0 [0%, 0%, 0%]	0 [0%, 0%, 0%]	204 [1% of Total]
-150m to -200m	58 [34%, 3%, 0%]	112 [66%, 4%, 1%]	0 [0%, 0%, 0%]	0 [0%, 0%, 0%]	0 [0%, 0%, 0%]	0 [0%, 0%, 0%]	170 [1% of Total]
<-200m	122 [1%, 7%, 1%]	1,992 [11%, 80%, 10%]	1,331 [7%, 100%, 7%]	2,506 [14%, 100%, 12%]	12,117 [67%, 100%, 60%]	0 [0%, 0%, 0%]	18,069 [89% of Total]
Total	1,824 [9% of Total]	2,484 [12% of Total]	1,331 [7% of Total]	2,506 [12% of Total]	12,117 [60% of Total]	0.000 [0% of Total]	20,262 Km^2

Areas in Costa Rica EEZ Excluding Protected Areas: Cell Values = Area in Km^2 [% Depth Band (Row), % Distance Band (Column), % EEZ]

841 [4%] Km^2 Protected	0 to 5 NM	5 to 12 NM	12 to 16 NM	16 to 24 NM	24 to 200 NM	> 200 NM	Total
> -10m	137 [100%, 10%, 1%]	0 [0%, 0%, 0%]	0 [0%, 0%, 0%]	0 [0%, 0%, 0%]	0 [0%, 0%, 0%]	0 [0%, 0%, 0%]	137 [1% of Total]
-10m to -50m	812 [99%, 57%, 4%]	11 [1%, 1%, 0%]	0 [0%, 0%, 0%]	0 [0%, 0%, 0%]	0 [0%, 0%, 0%]	0 [0%, 0%, 0%]	823 [4% of Total]
-50m to -100m	248 [56%, 17%, 1%]	193 [44%, 9%, 1%]	0 [0%, 0%, 0%]	0 [0%, 0%, 0%]	0 [0%, 0%, 0%]	0 [0%, 0%, 0%]	441 [2% of Total]
-100m to -150m	73 [42%, 5%, 0%]	102 [58%, 5%, 1%]	0 [0%, 0%, 0%]	0 [0%, 0%, 0%]	0 [0%, 0%, 0%]	0 [0%, 0%, 0%]	175 [1% of Total]
-150m to -200m	51 [36%, 4%, 0%]	90 [64%, 4%, 0%]	0 [0%, 0%, 0%]	0 [0%, 0%, 0%]	0 [0%, 0%, 0%]	0 [0%, 0%, 0%]	140 [1% of Total]
<-200m	113 [1%, 8%, 1%]	1,653 [9%, 81%, 9%]	1,315 [7%, 100%, 7%]	2,506 [14%, 100%, 13%]	12,117 [68%, 100%, 62%]	0 [0%, 0%, 0%]	17,704 [91% of Total]
Total	1,434 [7% of Total]	2,049 [11% of Total]	1,315 [7% of Total]	2,506 [13% of Total]	12,117 [62% of Total]	0.000 [0% of Total]	19,421 Km^2

The designations employed and the presentation of material in the map do not imply the expression of any opinion whatsoever on the part of FAO concerning the legal or constitutional status of any country, territory or sea area, or concerning the delimitation of frontiers.

Background reference map from National Geographic. Content may not reflect National Geographic's current map policy. Sources: National Geographic, Esri, DeLorme, HERE, UNEP-WCMC, USGS, NASA, ESA, METI, NRCAN, GEBCO, NOAA, increment P Corp.

Projection: Azimuthal Equidistant
Datum: WGS 1984
False Easting: 0.0000
False Northing: 0.0000
Central Meridian: -82.5509
Latitude Of Origin: 10.2776

Croatia: Mediterranean Region

Food and Agriculture Organization of the United Nations

©FAO, 2018

Kilometers
0 125 250

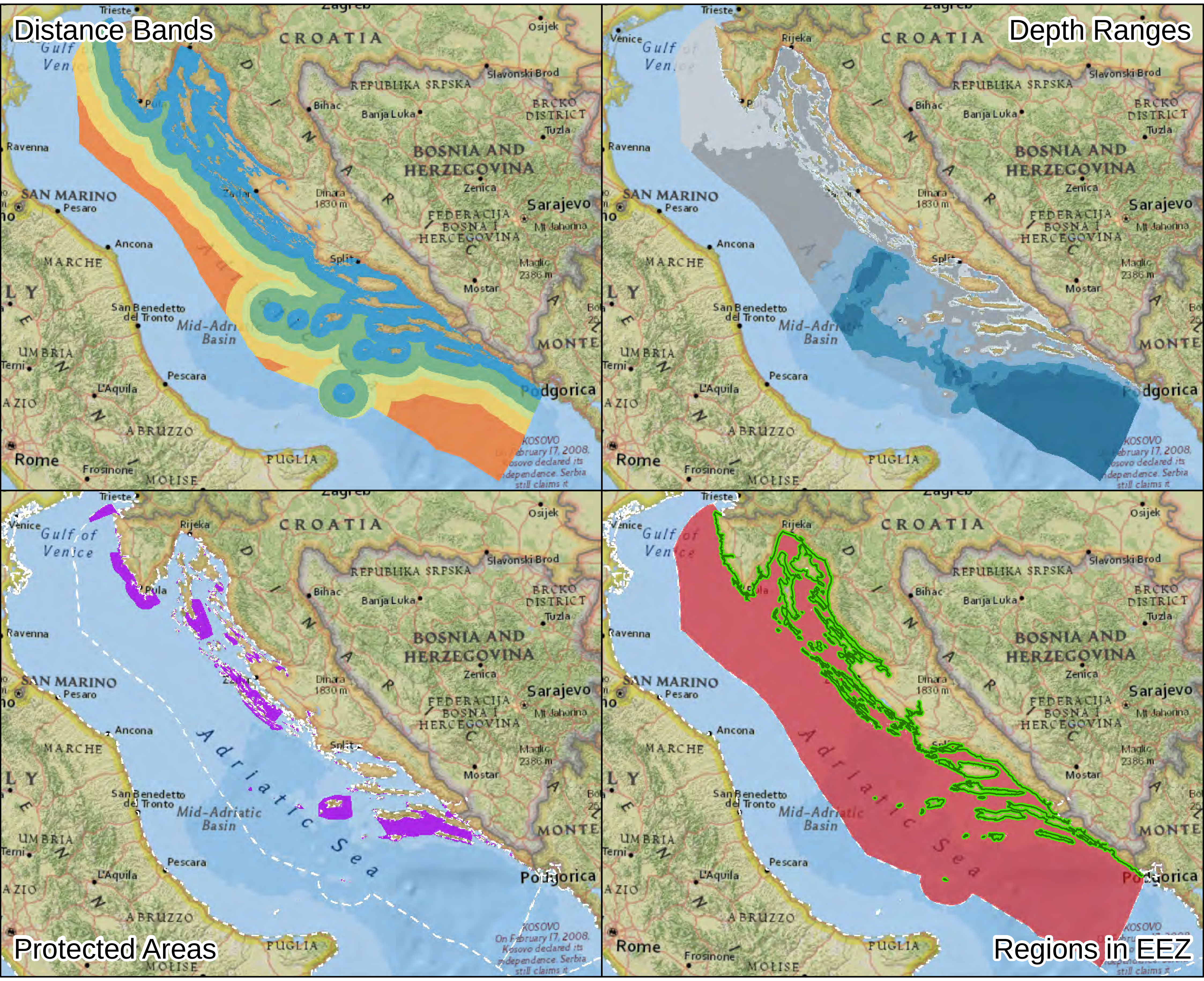

Distance Bands

- 0 to 5 NM
- 5 to 12 NM
- 12 to 16 NM
- 16 to 24 NM
- 24 to 200 NM

Depth Bands

- > -10m
- -10m to -50m
- -50m to -100m
- -100m to -150m
- -150m to -200m
- < -200m

Protected

- Protected Areas

Coastline Length

3,981 Km

Region

- Mediterranean

All Areas in Croatia EEZ: Cell Values = Area in Km² [% Depth Band (Row), % Distance Band (Column), % EEZ]

	0 to 5 NM	5 to 12 NM	12 to 16 NM	16 to 24 NM	24 to 200 NM	> 200 NM	Total
> -10m	2,050 [100%, 12%, 4%]	0 [0%, 0%, 0%]	0 [0%, 0%, 0%]	0 [0%, 0%, 0%]	0 [0%, 0%, 0%]	0 [0%, 0%, 0%]	2,050 [4% of Total]
-10m to -50m	5,097 [49%, 29%, 9%]	2,019 [19%, 16%, 4%]	986 [9%, 17%, 2%]	1,643 [16%, 18%, 3%]	666 [6%, 7%, 1%]	0 [0%, 0%, 0%]	10,411 [19% of Total]
-50m to -100m	7,251 [47%, 41%, 13%]	2,121 [14%, 17%, 4%]	941 [6%, 16%, 2%]	2,096 [14%, 23%, 4%]	2,909 [19%, 29%, 5%]	0 [0%, 0%, 0%]	15,318 [28% of Total]
-100m to -150m	2,658 [28%, 15%, 5%]	3,494 [37%, 28%, 6%]	739 [8%, 13%, 1%]	1,577 [17%, 17%, 3%]	922 [10%, 9%, 2%]	0 [0%, 0%, 0%]	9,390 [17% of Total]
-150m to -200m	500 [8%, 3%, 1%]	3,208 [50%, 26%, 6%]	1,634 [26%, 28%, 3%]	1,064 [17%, 12%, 2%]	0 [0%, 0%, 0%]	0 [0%, 0%, 0%]	6,405 [12% of Total]
<-200m	26 [0%, 0%, 0%]	1,599 [14%, 13%, 3%]	1,537 [13%, 26%, 3%]	2,846 [25%, 31%, 5%]	5,386 [47%, 55%, 10%]	0 [0%, 0%, 0%]	11,394 [21% of Total]
Total	17,581 [32% of Total]	12,441 [23% of Total]	5,838 [11% of Total]	9,227 [17% of Total]	9,883 [18% of Total]	0.000 [0% of Total]	54,969 Km²

Areas in Croatia EEZ Excluding Protected Areas: Cell Values = Area in Km² [% Depth Band (Row), % Distance Band (Column), % EEZ]

4,847 [9%] Km² Protected	0 to 5 NM	5 to 12 NM	12 to 16 NM	16 to 24 NM	24 to 200 NM	> 200 NM	Total
> -10m	1,052 [100%, 8%, 2%]	0 [0%, 0%, 0%]	0 [0%, 0%, 0%]	0 [0%, 0%, 0%]	0 [0%, 0%, 0%]	0 [0%, 0%, 0%]	1,052 [2% of Total]
-10m to -50m	3,405 [40%, 26%, 7%]	1,893 [22%, 16%, 4%]	986 [11%, 17%, 2%]	1,643 [19%, 18%, 3%]	666 [8%, 7%, 1%]	0 [0%, 0%, 0%]	8,594 [17% of Total]
-50m to -100m	5,694 [42%, 43%, 11%]	1,962 [14%, 16%, 4%]	941 [7%, 16%, 2%]	2,096 [15%, 23%, 4%]	2,909 [21%, 29%, 6%]	0 [0%, 0%, 0%]	13,602 [27% of Total]
-100m to -150m	2,432 [27%, 19%, 5%]	3,446 [38%, 29%, 7%]	739 [8%, 13%, 1%]	1,577 [17%, 17%, 3%]	922 [10%, 9%, 2%]	0 [0%, 0%, 0%]	9,116 [18% of Total]
-150m to -200m	493 [8%, 4%, 1%]	3,183 [50%, 26%, 6%]	1,627 [26%, 28%, 3%]	1,064 [17%, 12%, 2%]	0 [0%, 0%, 0%]	0 [0%, 0%, 0%]	6,366 [13% of Total]
<-200m	26 [0%, 0%, 0%]	1,598 [14%, 13%, 3%]	1,536 [13%, 26%, 3%]	2,846 [25%, 31%, 6%]	5,386 [47%, 55%, 11%]	0 [0%, 0%, 0%]	11,392 [23% of Total]
Total	13,102 [26% of Total]	12,082 [24% of Total]	5,829 [12% of Total]	9,227 [18% of Total]	9,883 [20% of Total]	0.000 [0% of Total]	50,122 Km²

The designations employed and the presentation of material in the map do not imply the expression of any opinion whatsoever on the part of FAO concerning the legal or constitutional status of any country, territory or sea area, or concerning the delimitation of frontiers.

Background reference map from National Geographic. Content may not reflect National Geographic's current map policy. Sources: National Geographic, Esri, DeLorme, HERE, UNEP-WCMC, USGS, NASA, ESA, METI, NRCAN, GEBCO, NOAA, increment P Corp.

Projection: Azimuthal Equidistant
Datum: WGS 1984
False Easting: 0.0000
False Northing: 0.0000
Central Meridian: 15.7788
Latitude Of Origin: 43.6054

Cuba: Caribbean Region

©FAO, 2018

Food and Agriculture Organization of the United Nations

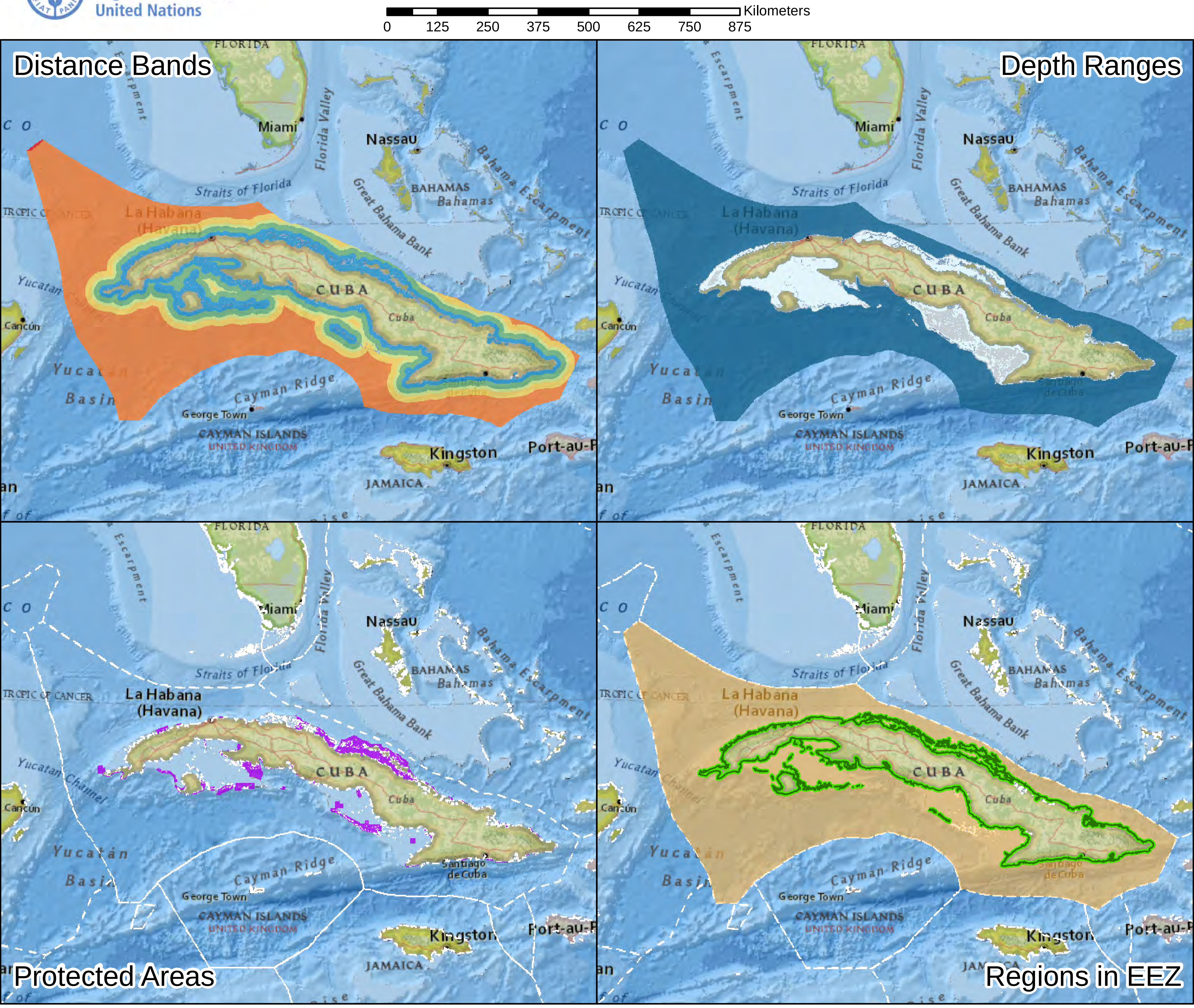

Distance Bands

- 0 to 5 NM
- 5 to 12 NM
- 12 to 16 NM
- 16 to 24 NM
- 24 to 200 NM
- > 200 NM

Depth Bands

- > -10m
- -10m to -50m
- -50m to -100m
- -100m to -150m
- -150m to -200m
- < -200m

Protected

- Protected Areas

Coastline Length

10,364 Km

Region

- Caribbean

All Areas in Cuba EEZ: Cell Values = Area in Km^2 [% Depth Band (Row), % Distance Band (Column), % EEZ]

	0 to 5 NM	5 to 12 NM	12 to 16 NM	16 to 24 NM	24 to 200 NM	> 200 NM	Total
> -10m	26,400 [69%, 52%, 7%]	8,820 [23%, 18%, 3%]	1,961 [5%, 8%, 1%]	1,194 [3%, 3%, 0%]	12 [0%, 0%, 0%]	0 [0%, 0%, 0%]	38,387 [11% of Total]
-10m to -50m	4,753 [35%, 9%, 1%]	5,840 [43%, 12%, 2%]	2,189 [16%, 9%, 1%]	831 [6%, 2%, 0%]	14 [0%, 0%, 0%]	0 [0%, 0%, 0%]	13,627 [4% of Total]
-50m to -100m	1,360 [81%, 3%, 0%]	160 [9%, 0%, 0%]	37 [2%, 0%, 0%]	117 [7%, 0%, 0%]	12 [1%, 0%, 0%]	0 [0%, 0%, 0%]	1,686 [0% of Total]
-100m to -150m	1,054 [81%, 2%, 0%]	118 [9%, 0%, 0%]	33 [3%, 0%, 0%]	87 [7%, 0%, 0%]	13 [1%, 0%, 0%]	0 [0%, 0%, 0%]	1,305 [0% of Total]
-150m to -200m	780 [76%, 2%, 0%]	126 [12%, 0%, 0%]	30 [3%, 0%, 0%]	85 [8%, 0%, 0%]	6 [1%, 0%, 0%]	0 [0%, 0%, 0%]	1,027 [0% of Total]
<-200m	15,943 [5%, 32%, 5%]	33,567 [11%, 69%, 10%]	19,005 [6%, 82%, 5%]	35,324 [12%, 94%, 10%]	192,027 [65%, 100%, 55%]	264 [0%, 100%, 0%]	296,130 [84% of Total]
Total	50,290 [14% of Total]	48,631 [14% of Total]	23,255 [7% of Total]	37,639 [11% of Total]	192,084 [55% of Total]	264 [0% of Total]	352,162 Km^2

Areas in Cuba EEZ Excluding Protected Areas: Cell Values = Area in Km^2 [% Depth Band (Row), % Distance Band (Column), % EEZ]

14,056 [4%] Km^2 Protected	0 to 5 NM	5 to 12 NM	12 to 16 NM	16 to 24 NM	24 to 200 NM	> 200 NM	Total
> -10m	18,721 [64%, 48%, 6%]	8,254 [28%, 18%, 2%]	1,585 [5%, 7%, 0%]	854 [3%, 2%, 0%]	8 [0%, 0%, 0%]	0 [0%, 0%, 0%]	29,423 [9% of Total]
-10m to -50m	3,273 [28%, 8%, 1%]	5,582 [48%, 12%, 2%]	2,064 [18%, 9%, 1%]	792 [7%, 2%, 0%]	12 [0%, 0%, 0%]	0 [0%, 0%, 0%]	11,723 [3% of Total]
-50m to -100m	621 [71%, 2%, 0%]	105 [12%, 0%, 0%]	32 [4%, 0%, 0%]	105 [12%, 0%, 0%]	11 [1%, 0%, 0%]	0 [0%, 0%, 0%]	875 [0% of Total]
-100m to -150m	559 [74%, 1%, 0%]	76 [10%, 0%, 0%]	28 [4%, 0%, 0%]	80 [11%, 0%, 0%]	10 [1%, 0%, 0%]	0 [0%, 0%, 0%]	753 [0% of Total]
-150m to -200m	513 [72%, 1%, 0%]	88 [12%, 0%, 0%]	29 [4%, 0%, 0%]	79 [11%, 0%, 0%]	6 [1%, 0%, 0%]	0 [0%, 0%, 0%]	715 [0% of Total]
<-200m	14,973 [5%, 39%, 4%]	33,037 [11%, 70%, 10%]	19,002 [6%, 84%, 6%]	35,319 [12%, 95%, 10%]	192,022 [65%, 100%, 57%]	264 [0%, 100%, 0%]	294,617 [87% of Total]
Total	38,659 [11% of Total]	47,142 [14% of Total]	22,742 [7% of Total]	37,229 [11% of Total]	192,070 [57% of Total]	264 [0% of Total]	338,106 Km^2

The designations employed and the presentation of material in the map do not imply the expression of any opinion whatsoever on the part of FAO concerning the legal or constitutional status of any country, territory or sea area, or concerning the delimitation of frontiers.

Background reference map from National Geographic. Content may not reflect National Geographic's current map policy. Sources: National Geographic, Esri, DeLorme, HERE, UNEP-WCMC, USGS, NASA, ESA, METI, NRCAN, GEBCO, NOAA, increment P Corp.

Projection: Azimuthal Equidistant
Datum: WGS 1984
False Easting: 0.0000
False Northing: 0.0000
Central Meridian: -80.2610
Latitude Of Origin: 22.0284

Cyprus: Mediterranean Region

Food and Agriculture Organization of the United Nations

©FAO, 2018

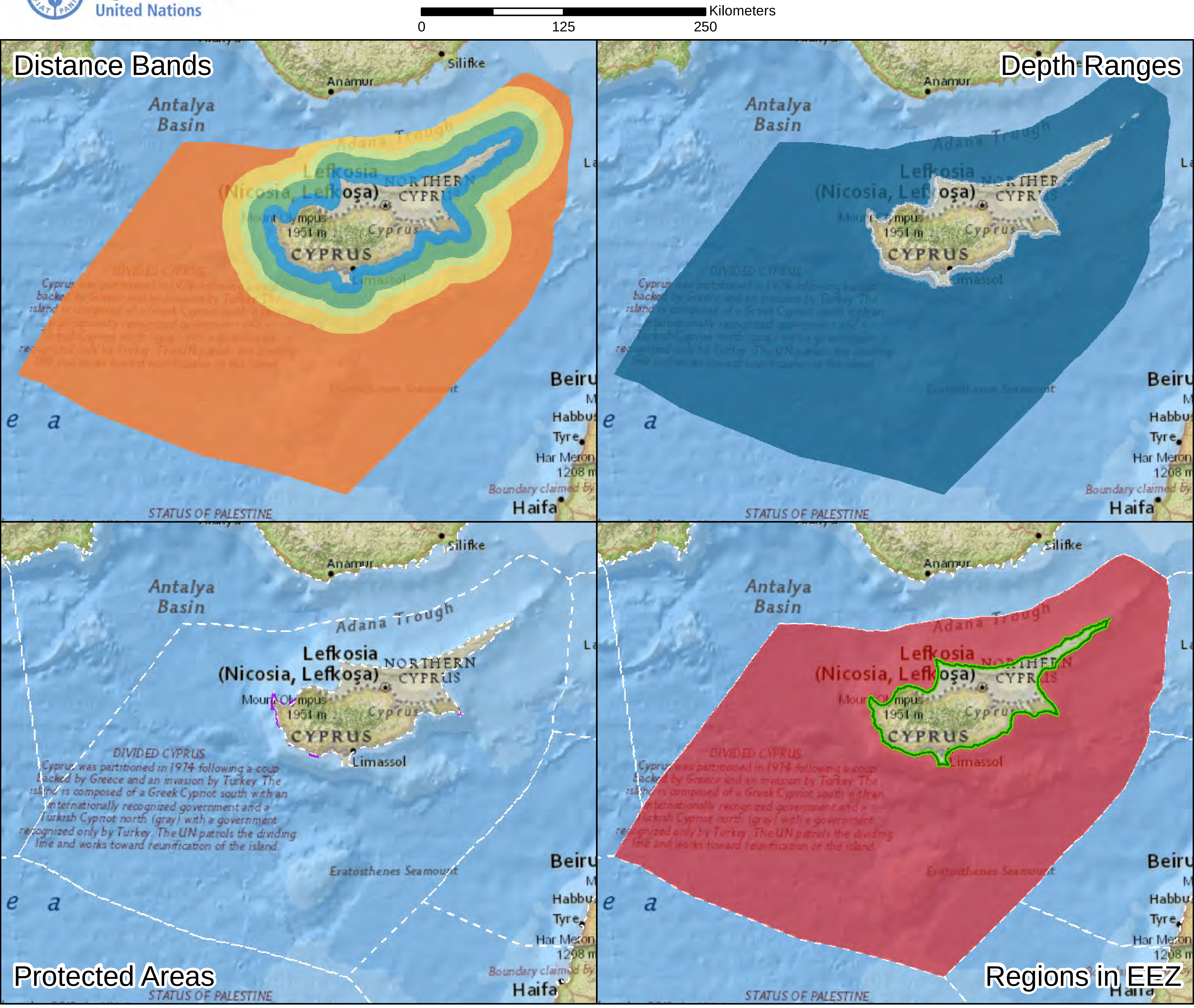

Distance Bands

- 0 to 5 NM
- 5 to 12 NM
- 12 to 16 NM
- 16 to 24 NM
- 24 to 200 NM

Depth Bands

- > -10m
- -10m to -50m
- -50m to -100m
- -100m to -150m
- -150m to -200m
- < -200m

Protected

- Protected Areas

Coastline Length

857 Km

Region

- Mediterranean

All Areas in Cyprus EEZ: Cell Values = Area in Km^2 [% Depth Band (Row), % Distance Band (Column), % EEZ]

	0 to 5 NM	5 to 12 NM	12 to 16 NM	16 to 24 NM	24 to 200 NM	> 200 NM	Total
> -10m	314 [100%, 5%, 0%]	0 [0%, 0%, 0%]	0 [0%, 0%, 0%]	0 [0%, 0%, 0%]	0 [0%, 0%, 0%]	0 [0%, 0%, 0%]	314 [0% of Total]
-10m to -50m	876 [100%, 14%, 1%]	0 [0%, 0%, 0%]	0 [0%, 0%, 0%]	0 [0%, 0%, 0%]	0 [0%, 0%, 0%]	0 [0%, 0%, 0%]	876 [1% of Total]
-50m to -100m	737 [100%, 12%, 1%]	2 [0%, 0%, 0%]	0 [0%, 0%, 0%]	0.696 [0%, 0%, 0%]	0 [0%, 0%, 0%]	0 [0%, 0%, 0%]	739 [1% of Total]
-100m to -150m	456 [95%, 7%, 0%]	11 [2%, 0%, 0%]	7 [2%, 0%, 0%]	5 [1%, 0%, 0%]	0 [0%, 0%, 0%]	0 [0%, 0%, 0%]	480 [0% of Total]
-150m to -200m	321 [87%, 5%, 0%]	25 [7%, 0%, 0%]	11 [3%, 0%, 0%]	12 [3%, 0%, 0%]	0 [0%, 0%, 0%]	0 [0%, 0%, 0%]	368 [0% of Total]
<-200m	3,503 [4%, 56%, 4%]	8,928 [9%, 100%, 9%]	5,385 [6%, 100%, 5%]	10,796 [11%, 100%, 11%]	67,074 [70%, 100%, 68%]	0 [0%, 0%, 0%]	95,685 [97% of Total]
Total	6,206 [6% of Total]	8,965 [9% of Total]	5,403 [5% of Total]	10,813 [11% of Total]	67,074 [68% of Total]	0.000 [0% of Total]	98,462 Km^2

Areas in Cyprus EEZ Excluding Protected Areas: Cell Values = Area in Km^2 [% Depth Band (Row), % Distance Band (Column), % EEZ]

127 [0%] Km^2 Protected	0 to 5 NM	5 to 12 NM	12 to 16 NM	16 to 24 NM	24 to 200 NM	> 200 NM	Total
> -10m	282 [100%, 5%, 0%]	0 [0%, 0%, 0%]	0 [0%, 0%, 0%]	0 [0%, 0%, 0%]	0 [0%, 0%, 0%]	0 [0%, 0%, 0%]	282 [0% of Total]
-10m to -50m	796 [100%, 13%, 1%]	0 [0%, 0%, 0%]	0 [0%, 0%, 0%]	0 [0%, 0%, 0%]	0 [0%, 0%, 0%]	0 [0%, 0%, 0%]	796 [1% of Total]
-50m to -100m	722 [100%, 12%, 1%]	2 [0%, 0%, 0%]	0 [0%, 0%, 0%]	0.696 [0%, 0%, 0%]	0 [0%, 0%, 0%]	0 [0%, 0%, 0%]	724 [1% of Total]
-100m to -150m	456 [95%, 7%, 0%]	11 [2%, 0%, 0%]	7 [2%, 0%, 0%]	5 [1%, 0%, 0%]	0 [0%, 0%, 0%]	0 [0%, 0%, 0%]	479 [0% of Total]
-150m to -200m	320 [87%, 5%, 0%]	25 [7%, 0%, 0%]	11 [3%, 0%, 0%]	12 [3%, 0%, 0%]	0 [0%, 0%, 0%]	0 [0%, 0%, 0%]	368 [0% of Total]
<-200m	3,503 [4%, 58%, 4%]	8,928 [9%, 100%, 9%]	5,385 [6%, 100%, 5%]	10,796 [11%, 100%, 11%]	67,074 [70%, 100%, 68%]	0 [0%, 0%, 0%]	95,685 [97% of Total]
Total	6,079 [6% of Total]	8,965 [9% of Total]	5,403 [5% of Total]	10,813 [11% of Total]	67,074 [68% of Total]	0.000 [0% of Total]	98,335 Km^2

The designations employed and the presentation of material in the map do not imply the expression of any opinion whatsoever on the part of FAO concerning the legal or constitutional status of any country, territory or sea area, or concerning the delimitation of frontiers.

Background reference map from National Geographic. Content may not reflect National Geographic's current map policy. Sources: National Geographic, Esri, DeLorme, HERE, UNEP-WCMC, USGS, NASA, ESA, METI, NRCAN, GEBCO, NOAA, increment P Corp.

Projection: Azimuthal Equidistant
Datum: WGS 1984
False Easting: 0.0000
False Northing: 0.0000
Central Meridian: 32.5204
Latitude Of Origin: 34.5540

Food and Agriculture Organization of the United Nations

Denmark: Baltic Region

©FAO, 2018

Kilometers
0 125 250 375 500

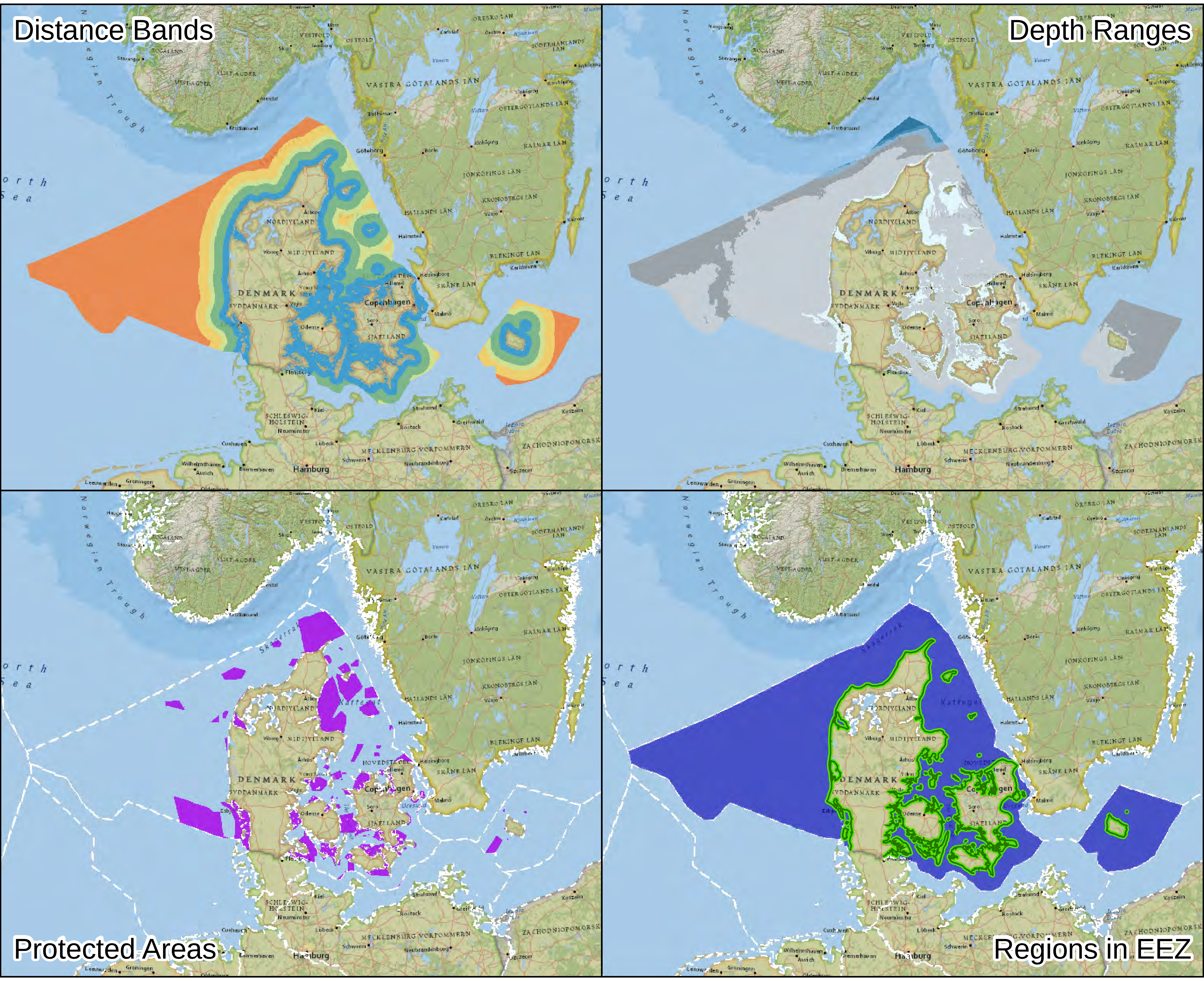

Distance Bands
- 0 to 5 NM
- 5 to 12 NM
- 12 to 16 NM
- 16 to 24 NM
- 24 to 200 NM

Depth Bands
- > -10m
- -10m to -50m
- -50m to -100m
- -100m to -150m
- -150m to -200m
- < -200m

Protected
- Protected Areas

Coastline Length
- 4,803 Km

Region
- Baltic

All Areas in Denmark EEZ: Cell Values = Area in Km^2 [% Depth Band (Row), % Distance Band (Column), % EEZ]

	0 to 5 NM	5 to 12 NM	12 to 16 NM	16 to 24 NM	24 to 200 NM	> 200 NM	Total
> -10m	9,483 [87%, 39%, 9%]	1,120 [10%, 6%, 1%]	196 [2%, 2%, 0%]	47 [0%, 0%, 0%]	0 [0%, 0%, 0%]	0 [0%, 0%, 0%]	10,846 [11% of Total]
-10m to -50m	14,280 [21%, 58%, 14%]	14,562 [22%, 78%, 14%]	6,160 [9%, 76%, 6%]	6,620 [10%, 62%, 6%]	25,004 [38%, 62%, 24%]	0 [0%, 0%, 0%]	66,628 [65% of Total]
-50m to -100m	801 [4%, 3%, 1%]	2,651 [12%, 14%, 3%]	1,586 [7%, 20%, 2%]	3,558 [16%, 33%, 3%]	13,571 [61%, 34%, 13%]	0 [0%, 0%, 0%]	22,167 [22% of Total]
-100m to -150m	21 [1%, 0%, 0%]	270 [19%, 1%, 0%]	89 [6%, 1%, 0%]	360 [25%, 3%, 0%]	703 [49%, 2%, 1%]	0 [0%, 0%, 0%]	1,444 [1% of Total]
-150m to -200m	0 [0%, 0%, 0%]	31 [5%, 0%, 0%]	85 [14%, 1%, 0%]	173 [29%, 2%, 0%]	303 [51%, 1%, 0%]	0 [0%, 0%, 0%]	592 [1% of Total]
<-200m	0 [0%, 0%, 0%]	0 [0%, 0%, 0%]	5 [1%, 0%, 0%]	0.696 [0%, 0%, 0%]	811 [99%, 2%, 1%]	0 [0%, 0%, 0%]	816 [1% of Total]
Total	24,586 [24% of Total]	18,635 [18% of Total]	8,121 [8% of Total]	10,760 [10% of Total]	40,392 [39% of Total]	0.000 [0% of Total]	102,493 Km^2

Areas in Denmark EEZ Excluding Protected Areas: Cell Values = Area in Km^2 [% Depth Band (Row), % Distance Band (Column), % EEZ]

17,712 [17%] Km^2 Protected	0 to 5 NM	5 to 12 NM	12 to 16 NM	16 to 24 NM	24 to 200 NM	> 200 NM	Total
> -10m	3,679 [91%, 23%, 4%]	224 [6%, 1%, 0%]	103 [3%, 2%, 0%]	42 [1%, 0%, 0%]	0 [0%, 0%, 0%]	0 [0%, 0%, 0%]	4,048 [5% of Total]
-10m to -50m	11,499 [20%, 73%, 14%]	12,828 [22%, 84%, 15%]	5,225 [9%, 79%, 6%]	5,185 [9%, 61%, 6%]	23,348 [40%, 60%, 28%]	0 [0%, 0%, 0%]	58,086 [69% of Total]
-50m to -100m	664 [3%, 4%, 1%]	2,190 [11%, 14%, 3%]	1,302 [6%, 20%, 2%]	3,134 [15%, 37%, 4%]	13,558 [65%, 35%, 16%]	0 [0%, 0%, 0%]	20,849 [25% of Total]
-100m to -150m	0.489 [0%, 0%, 0%]	2 [0%, 0%, 0%]	0.912 [0%, 0%, 0%]	98 [13%, 1%, 0%]	658 [87%, 2%, 1%]	0 [0%, 0%, 0%]	759 [1% of Total]
-150m to -200m	0 [0%, 0%, 0%]	0 [0%, 0%, 0%]	2 [1%, 0%, 0%]	9 [4%, 0%, 0%]	222 [96%, 1%, 0%]	0 [0%, 0%, 0%]	232 [0% of Total]
<-200m	0 [0%, 0%, 0%]	0 [0%, 0%, 0%]	1 [0%, 0%, 0%]	0.050 [0%, 0%, 0%]	806 [100%, 2%, 1%]	0 [0%, 0%, 0%]	807 [1% of Total]
Total	15,842 [19% of Total]	15,245 [18% of Total]	6,634 [8% of Total]	8,468 [10% of Total]	38,592 [46% of Total]	0.000 [0% of Total]	84,781 Km^2

The designations employed and the presentation of material in the map do not imply the expression of any opinion whatsoever on the part of FAO concerning the legal or constitutional status of any country, territory or sea area, or concerning the delimitation of frontiers.

Background reference map from National Geographic. Content may not reflect National Geographic's current map policy. Sources: National Geographic, Esri, DeLorme, HERE, UNEP-WCMC, USGS, NASA, ESA, METI, NRCAN, GEBCO, NOAA, increment P Corp.

Projection: Azimuthal Equidistant
Datum: WGS 1984
False Easting: 0.0000
False Northing: 0.0000
Central Meridian: 9.8787
Latitude Of Origin: 56.3131

Food and Agriculture Organization of the United Nations

Dominica: Caribbean Region

©FAO, 2018

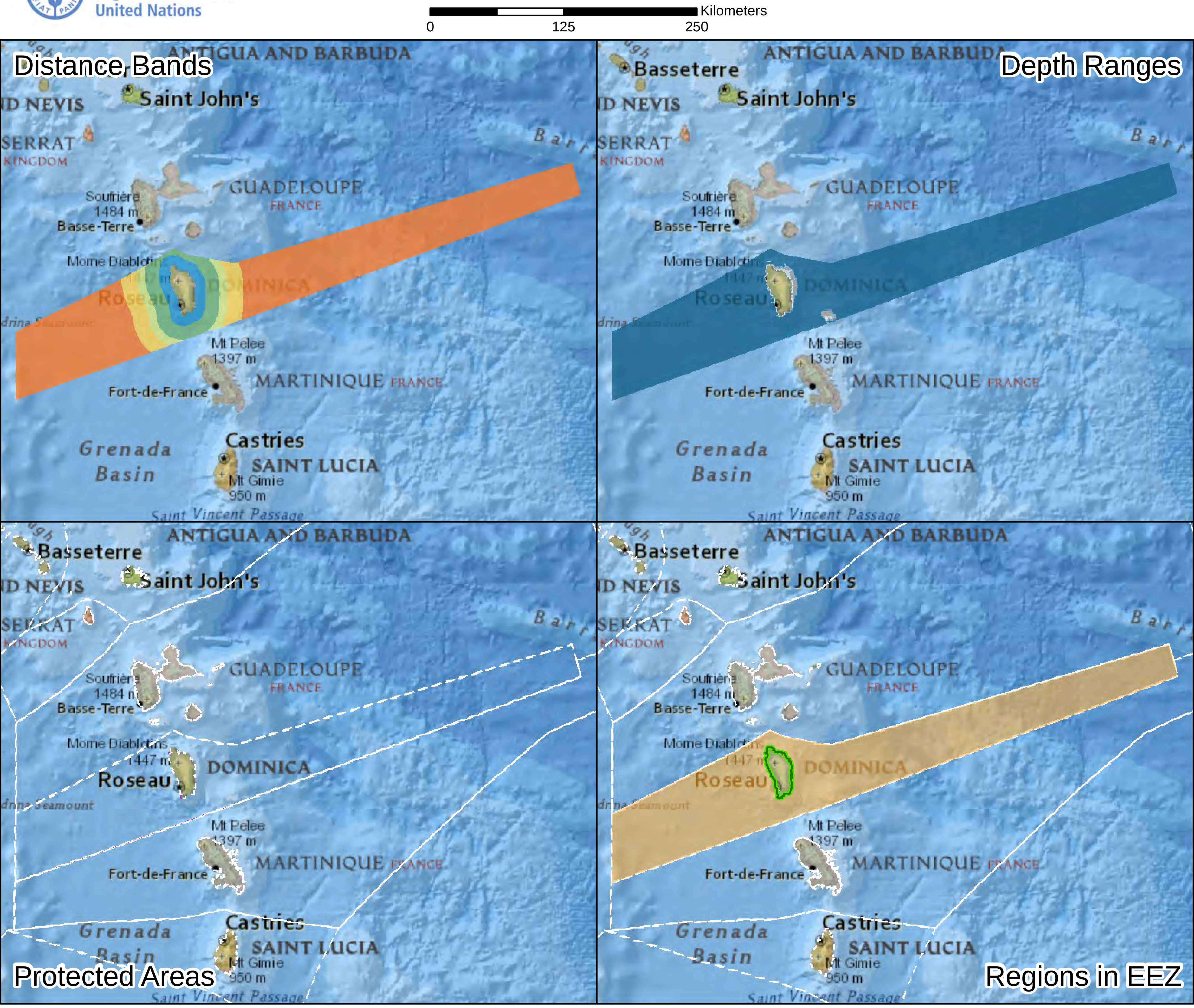

Distance Bands

- 0 to 5 NM
- 5 to 12 NM
- 12 to 16 NM
- 16 to 24 NM
- 24 to 200 NM

Depth Bands

- > -10m
- -10m to -50m
- -50m to -100m
- -100m to -150m
- -150m to -200m
- < -200m

Protected

- Protected Areas

Coastline Length

201 Km

Region

- Caribbean

All Areas in Dominica EEZ: Cell Values = Area in Km^2 [% Depth Band (Row), % Distance Band (Column), % EEZ]

	0 to 5 NM	5 to 12 NM	12 to 16 NM	16 to 24 NM	24 to 200 NM	> 200 NM	Total
> -10m	63 [93%, 4%, 0%]	0 [0%, 0%, 0%]	0 [0%, 0%, 0%]	5 [7%, 0%, 0%]	0 [0%, 0%, 0%]	0 [0%, 0%, 0%]	68 [0% of Total]
-10m to -50m	64 [78%, 4%, 0%]	0 [0%, 0%, 0%]	0 [0%, 0%, 0%]	18 [22%, 1%, 0%]	0 [0%, 0%, 0%]	0 [0%, 0%, 0%]	82 [0% of Total]
-50m to -100m	140 [71%, 10%, 0%]	0 [0%, 0%, 0%]	4 [2%, 0%, 0%]	53 [27%, 3%, 0%]	0 [0%, 0%, 0%]	0 [0%, 0%, 0%]	197 [1% of Total]
-100m to -150m	47 [68%, 3%, 0%]	0 [0%, 0%, 0%]	4 [5%, 0%, 0%]	18 [27%, 1%, 0%]	0 [0%, 0%, 0%]	0 [0%, 0%, 0%]	69 [0% of Total]
-150m to -200m	43 [71%, 3%, 0%]	2 [3%, 0%, 0%]	3 [4%, 0%, 0%]	13 [22%, 1%, 0%]	0 [0%, 0%, 0%]	0 [0%, 0%, 0%]	60 [0% of Total]
<-200m	1,095 [4%, 75%, 4%]	2,402 [9%, 100%, 8%]	1,121 [4%, 99%, 4%]	1,930 [7%, 95%, 7%]	21,567 [77%, 100%, 75%]	0 [0%, 0%, 0%]	28,116 [98% of Total]
Total	1,452 [5% of Total]	2,404 [8% of Total]	1,132 [4% of Total]	2,038 [7% of Total]	21,567 [75% of Total]	0.000 [0% of Total]	28,593 Km^2

Areas in Dominica EEZ Excluding Protected Areas: Cell Values = Area in Km^2 [% Depth Band (Row), % Distance Band (Column), % EEZ]

36 [0%] Km^2 Protected	0 to 5 NM	5 to 12 NM	12 to 16 NM	16 to 24 NM	24 to 200 NM	> 200 NM	Total
> -10m	60 [92%, 4%, 0%]	0 [0%, 0%, 0%]	0 [0%, 0%, 0%]	5 [8%, 0%, 0%]	0 [0%, 0%, 0%]	0 [0%, 0%, 0%]	65 [0% of Total]
-10m to -50m	63 [78%, 4%, 0%]	0 [0%, 0%, 0%]	0 [0%, 0%, 0%]	18 [22%, 1%, 0%]	0 [0%, 0%, 0%]	0 [0%, 0%, 0%]	81 [0% of Total]
-50m to -100m	138 [71%, 10%, 0%]	0 [0%, 0%, 0%]	4 [2%, 0%, 0%]	53 [27%, 3%, 0%]	0 [0%, 0%, 0%]	0 [0%, 0%, 0%]	195 [1% of Total]
-100m to -150m	46 [68%, 3%, 0%]	0 [0%, 0%, 0%]	4 [5%, 0%, 0%]	18 [27%, 1%, 0%]	0 [0%, 0%, 0%]	0 [0%, 0%, 0%]	68 [0% of Total]
-150m to -200m	42 [71%, 3%, 0%]	2 [3%, 0%, 0%]	3 [4%, 0%, 0%]	13 [22%, 1%, 0%]	0 [0%, 0%, 0%]	0 [0%, 0%, 0%]	59 [0% of Total]
<-200m	1,093 [4%, 76%, 4%]	2,400 [9%, 100%, 8%]	1,119 [4%, 99%, 4%]	1,928 [7%, 95%, 7%]	21,549 [77%, 100%, 75%]	0 [0%, 0%, 0%]	28,089 [98% of Total]
Total	1,441 [5% of Total]	2,402 [8% of Total]	1,130 [4% of Total]	2,036 [7% of Total]	21,549 [75% of Total]	0.000 [0% of Total]	28,557 Km^2

The designations employed and the presentation of material in the map do not imply the expression of any opinion whatsoever on the part of FAO concerning the legal or constitutional status of any country, territory or sea area, or concerning the delimitation of frontiers.

Background reference map from National Geographic. Content may not reflect National Geographic's current map policy. Sources: National Geographic, Esri, DeLorme, HERE, UNEP-WCMC, USGS, NASA, ESA, METI, NRCAN, GEBCO, NOAA, increment P Corp.

Projection: Azimuthal Equidistant
Datum: WGS 1984
False Easting: 0.0000
False Northing: 0.0000
Central Meridian: -60.3444
Latitude Of Origin: 15.4947

Food and Agriculture Organization of the United Nations

Dominican Republic: Caribbean Region

©FAO, 2018

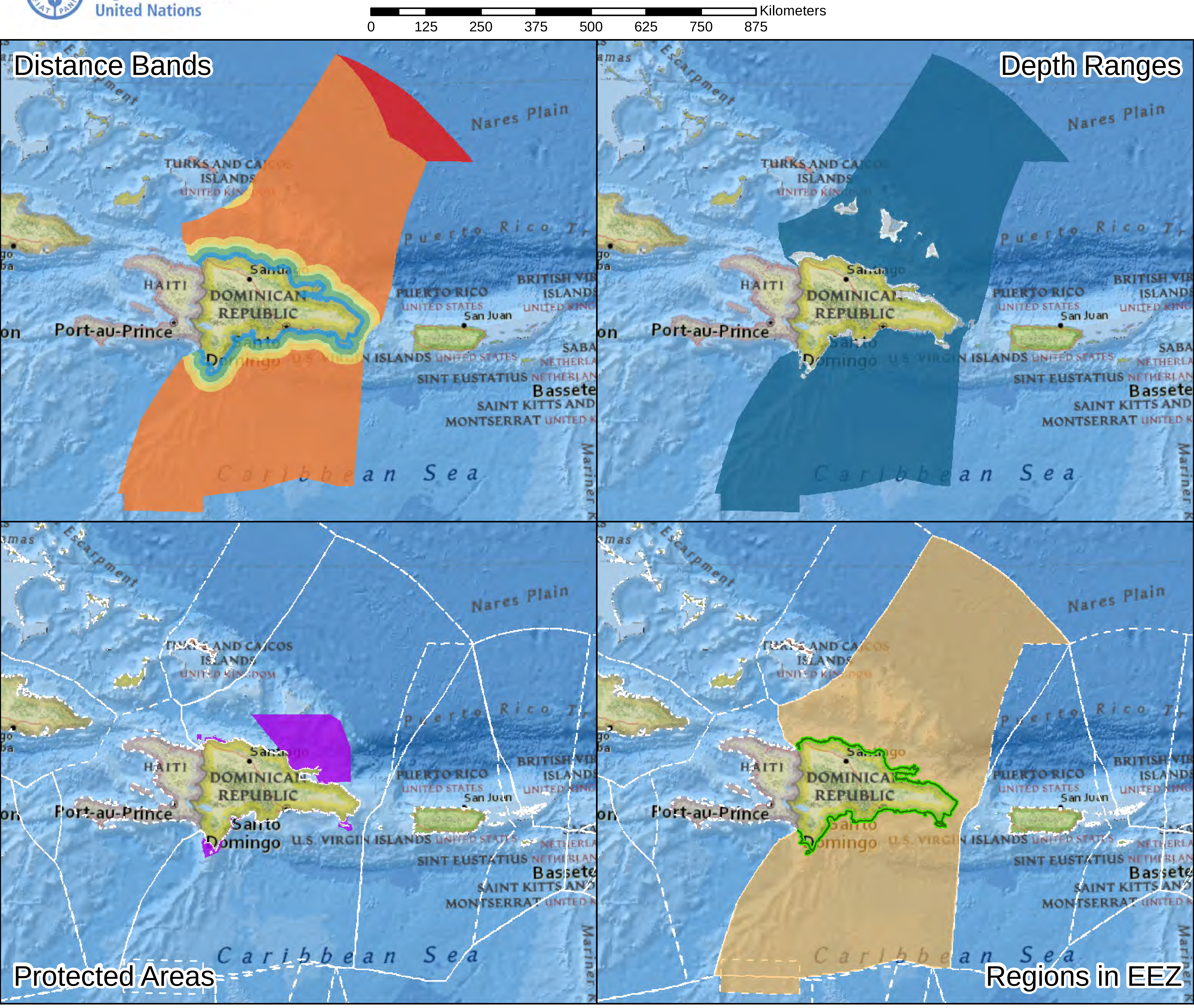

Distance Bands		Depth Bands		Protected	Region
0 to 5 NM	16 to 24 NM	> -10m	-100m to -150m	Protected Areas	Caribbean
5 to 12 NM	24 to 200 NM	-10m to -50m	-150m to -200m	**Coastline Length**	
12 to 16 NM	> 200 NM	-50m to -100m	< -200m	1,687 Km	

All Areas in Dominican Republic EEZ: Cell Values = Area in Km^2 [% Depth Band (Row), % Distance Band (Column), % EEZ]

	0 to 5 NM	5 to 12 NM	12 to 16 NM	16 to 24 NM	24 to 200 NM	> 200 NM	Total
> -10m	1,596 [57%, 14%, 0%]	24 [1%, 0%, 0%]	0 [0%, 0%, 0%]	0.614 [0%, 0%, 0%]	1,201 [43%, 0%, 0%]	0 [0%, 0%, 0%]	2,820 [1% of Total]
-10m to -50m	2,793 [55%, 24%, 1%]	403 [8%, 3%, 0%]	0 [0%, 0%, 0%]	2 [0%, 0%, 0%]	1,881 [37%, 1%, 1%]	0 [0%, 0%, 0%]	5,078 [1% of Total]
-50m to -100m	995 [44%, 9%, 0%]	256 [11%, 2%, 0%]	7 [0%, 0%, 0%]	102 [5%, 1%, 0%]	884 [39%, 0%, 0%]	0 [0%, 0%, 0%]	2,244 [1% of Total]
-100m to -150m	712 [45%, 6%, 0%]	206 [13%, 1%, 0%]	31 [2%, 0%, 0%]	42 [3%, 0%, 0%]	604 [38%, 0%, 0%]	0 [0%, 0%, 0%]	1,594 [0% of Total]
-150m to -200m	742 [56%, 6%, 0%]	272 [20%, 2%, 0%]	60 [5%, 1%, 0%]	35 [3%, 0%, 0%]	216 [16%, 0%, 0%]	0 [0%, 0%, 0%]	1,325 [0% of Total]
<-200m	4,805 [1%, 41%, 1%]	13,162 [4%, 92%, 4%]	8,176 [2%, 99%, 2%]	16,792 [5%, 99%, 5%]	280,066 [81%, 98%, 78%]	22,297 [6%, 100%, 6%]	345,299 [96% of Total]
Total	11,642 [3% of Total]	14,322 [4% of Total]	8,274 [2% of Total]	16,975 [5% of Total]	284,851 [79% of Total]	22,297 [6% of Total]	358,361 Km^2

Areas in Dominican Republic EEZ Excluding Protected Areas: Cell Values = Area in Km^2 [% Depth Band (Row), % Distance Band (Column), % EEZ]

24,445 [7%] Km^2 Protected	0 to 5 NM	5 to 12 NM	12 to 16 NM	16 to 24 NM	24 to 200 NM	> 200 NM	Total
> -10m	738 [56%, 9%, 0%]	20 [2%, 0%, 0%]	0 [0%, 0%, 0%]	0.614 [0%, 0%, 0%]	565 [43%, 0%, 0%]	0 [0%, 0%, 0%]	1,323 [0% of Total]
-10m to -50m	1,728 [51%, 22%, 1%]	248 [7%, 2%, 0%]	0 [0%, 0%, 0%]	2 [0%, 0%, 0%]	1,393 [41%, 1%, 0%]	0 [0%, 0%, 0%]	3,371 [1% of Total]
-50m to -100m	686 [39%, 9%, 0%]	169 [10%, 2%, 0%]	7 [0%, 0%, 0%]	102 [6%, 1%, 0%]	777 [45%, 0%, 0%]	0 [0%, 0%, 0%]	1,742 [1% of Total]
-100m to -150m	499 [40%, 6%, 0%]	152 [12%, 1%, 0%]	30 [2%, 0%, 0%]	42 [3%, 0%, 0%]	515 [42%, 0%, 0%]	0 [0%, 0%, 0%]	1,238 [0% of Total]
-150m to -200m	572 [56%, 7%, 0%]	203 [20%, 2%, 0%]	60 [6%, 1%, 0%]	35 [3%, 0%, 0%]	150 [15%, 0%, 0%]	0 [0%, 0%, 0%]	1,020 [0% of Total]
<-200m	3,684 [1%, 47%, 1%]	10,237 [3%, 93%, 3%]	6,396 [2%, 98%, 2%]	13,137 [4%, 99%, 4%]	269,471 [83%, 99%, 81%]	22,297 [7%, 100%, 7%]	325,222 [97% of Total]
Total	7,906 [2% of Total]	11,030 [3% of Total]	6,493 [2% of Total]	13,319 [4% of Total]	272,870 [82% of Total]	22,297 [7% of Total]	333,916 Km^2

The designations employed and the presentation of material in the map do not imply the expression of any opinion whatsoever on the part of FAO concerning the legal or constitutional status of any country, territory or sea area, or concerning the delimitation of frontiers.

Background reference map from National Geographic. Content may not reflect National Geographic's current map policy. Sources: National Geographic, Esri, DeLorme, HERE, UNEP-WCMC, USGS, NASA, ESA, METI, NRCAN, GEBCO, NOAA, increment P Corp.

Projection: Azimuthal Equidistant
Datum: WGS 1984
False Easting: 0.0000
False Northing: 0.0000
Central Meridian: -69.6483
Latitude Of Origin: 19.3822

Food and Agriculture Organization of the United Nations

Egypt: Mediterranean Region

©FAO, 2018

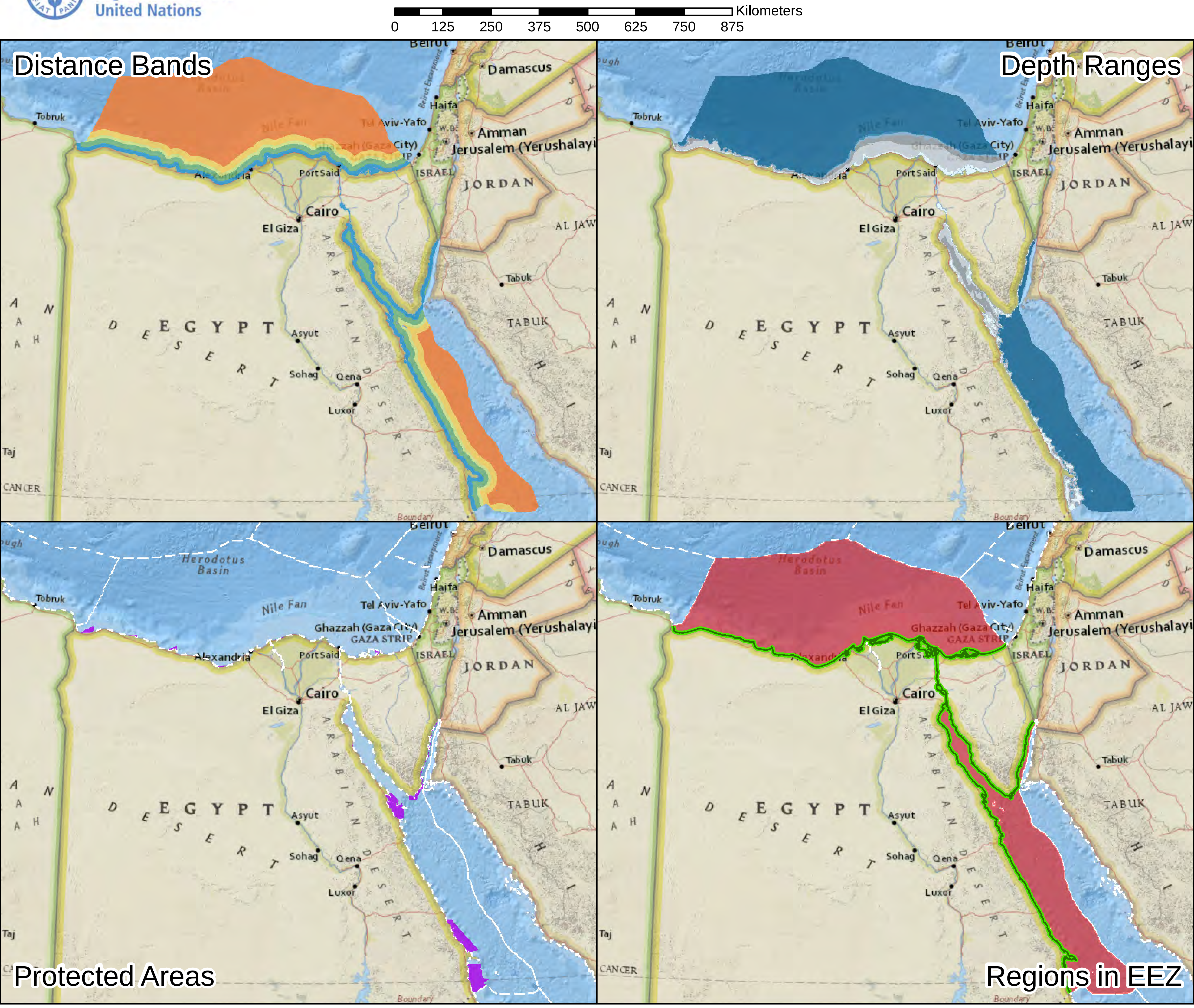

Distance Bands

- 0 to 5 NM
- 5 to 12 NM
- 12 to 16 NM
- 16 to 24 NM
- 24 to 200 NM

Depth Bands

- > -10m
- -10m to -50m
- -50m to -100m
- -100m to -150m
- -150m to -200m
- < -200m

Protected

- Protected Areas

Coastline Length

4,308 Km

Region

- Mediterranean

All Areas in Egypt EEZ: Cell Values = Area in Km^2 [% Depth Band (Row), % Distance Band (Column), % EEZ]

	0 to 5 NM	5 to 12 NM	12 to 16 NM	16 to 24 NM	24 to 200 NM	> 200 NM	Total
> -10m	6,317 [88%, 27%, 3%]	523 [7%, 2%, 0%]	181 [3%, 2%, 0%]	158 [2%, 1%, 0%]	7 [0%, 0%, 0%]	0 [0%, 0%, 0%]	7,186 [3% of Total]
-10m to -50m	9,709 [44%, 41%, 4%]	8,233 [38%, 34%, 3%]	2,172 [10%, 18%, 1%]	1,545 [7%, 7%, 1%]	222 [1%, 0%, 0%]	0 [0%, 0%, 0%]	21,881 [9% of Total]
-50m to -100m	3,059 [24%, 13%, 1%]	3,889 [30%, 16%, 2%]	1,504 [12%, 13%, 1%]	3,267 [25%, 14%, 1%]	1,292 [10%, 1%, 1%]	0 [0%, 0%, 0%]	13,011 [5% of Total]
-100m to -150m	688 [19%, 3%, 0%]	925 [26%, 4%, 0%]	226 [6%, 2%, 0%]	637 [18%, 3%, 0%]	1,132 [31%, 1%, 0%]	0 [0%, 0%, 0%]	3,608 [1% of Total]
-150m to -200m	504 [20%, 2%, 0%]	640 [25%, 3%, 0%]	229 [9%, 2%, 0%]	422 [16%, 2%, 0%]	769 [30%, 0%, 0%]	0 [0%, 0%, 0%]	2,564 [1% of Total]
<-200m	3,381 [2%, 14%, 1%]	10,129 [5%, 42%, 4%]	7,605 [4%, 64%, 3%]	16,797 [9%, 74%, 7%]	157,047 [81%, 98%, 65%]	0 [0%, 0%, 0%]	194,959 [80% of Total]
Total	23,659 [10% of Total]	24,340 [10% of Total]	11,916 [5% of Total]	22,826 [9% of Total]	160,468 [66% of Total]	0.000 [0% of Total]	243,209 Km^2

Areas in Egypt EEZ Excluding Protected Areas: Cell Values = Area in Km^2 [% Depth Band (Row), % Distance Band (Column), % EEZ]

7,248 [3%] Km^2 Protected	0 to 5 NM	5 to 12 NM	12 to 16 NM	16 to 24 NM	24 to 200 NM	> 200 NM	Total
> -10m	4,771 [97%, 24%, 2%]	94 [2%, 0%, 0%]	23 [0%, 0%, 0%]	12 [0%, 0%, 0%]	7 [0%, 0%, 0%]	0 [0%, 0%, 0%]	4,906 [2% of Total]
-10m to -50m	8,252 [42%, 42%, 3%]	7,482 [38%, 35%, 3%]	2,013 [10%, 18%, 1%]	1,485 [8%, 7%, 1%]	222 [1%, 0%, 0%]	0 [0%, 0%, 0%]	19,454 [8% of Total]
-50m to -100m	2,656 [22%, 13%, 1%]	3,361 [28%, 16%, 1%]	1,404 [12%, 12%, 1%]	3,245 [27%, 14%, 1%]	1,292 [11%, 1%, 1%]	0 [0%, 0%, 0%]	11,958 [5% of Total]
-100m to -150m	589 [19%, 3%, 0%]	563 [18%, 3%, 0%]	182 [6%, 2%, 0%]	630 [20%, 3%, 0%]	1,132 [37%, 1%, 0%]	0 [0%, 0%, 0%]	3,095 [1% of Total]
-150m to -200m	454 [20%, 2%, 0%]	434 [19%, 2%, 0%]	221 [10%, 2%, 0%]	418 [18%, 2%, 0%]	769 [33%, 0%, 0%]	0 [0%, 0%, 0%]	2,296 [1% of Total]
<-200m	3,163 [2%, 16%, 1%]	9,669 [5%, 45%, 4%]	7,585 [4%, 66%, 3%]	16,796 [9%, 74%, 7%]	157,040 [81%, 98%, 67%]	0 [0%, 0%, 0%]	194,251 [82% of Total]
Total	19,885 [8% of Total]	21,603 [9% of Total]	11,428 [5% of Total]	22,584 [10% of Total]	160,461 [68% of Total]	0.000 [0% of Total]	235,961 Km^2

The designations employed and the presentation of material in the map do not imply the expression of any opinion whatsoever on the part of FAO concerning the legal or constitutional status of any country, territory or sea area, or concerning the delimitation of frontiers.

Background reference map from National Geographic. Content may not reflect National Geographic's current map policy. Sources: National Geographic, Esri, DeLorme, HERE, UNEP-WCMC, USGS, NASA, ESA, METI, NRCAN, GEBCO, NOAA, increment P Corp.

Projection: Azimuthal Equidistant
Datum: WGS 1984
False Easting: 0.0000
False Northing: 0.0000
Central Meridian: 31.2013
Latitude Of Origin: 28.4570

Estonia: Baltic Region

©FAO, 2018

Food and Agriculture Organization of the United Nations

Kilometers
0 125 250

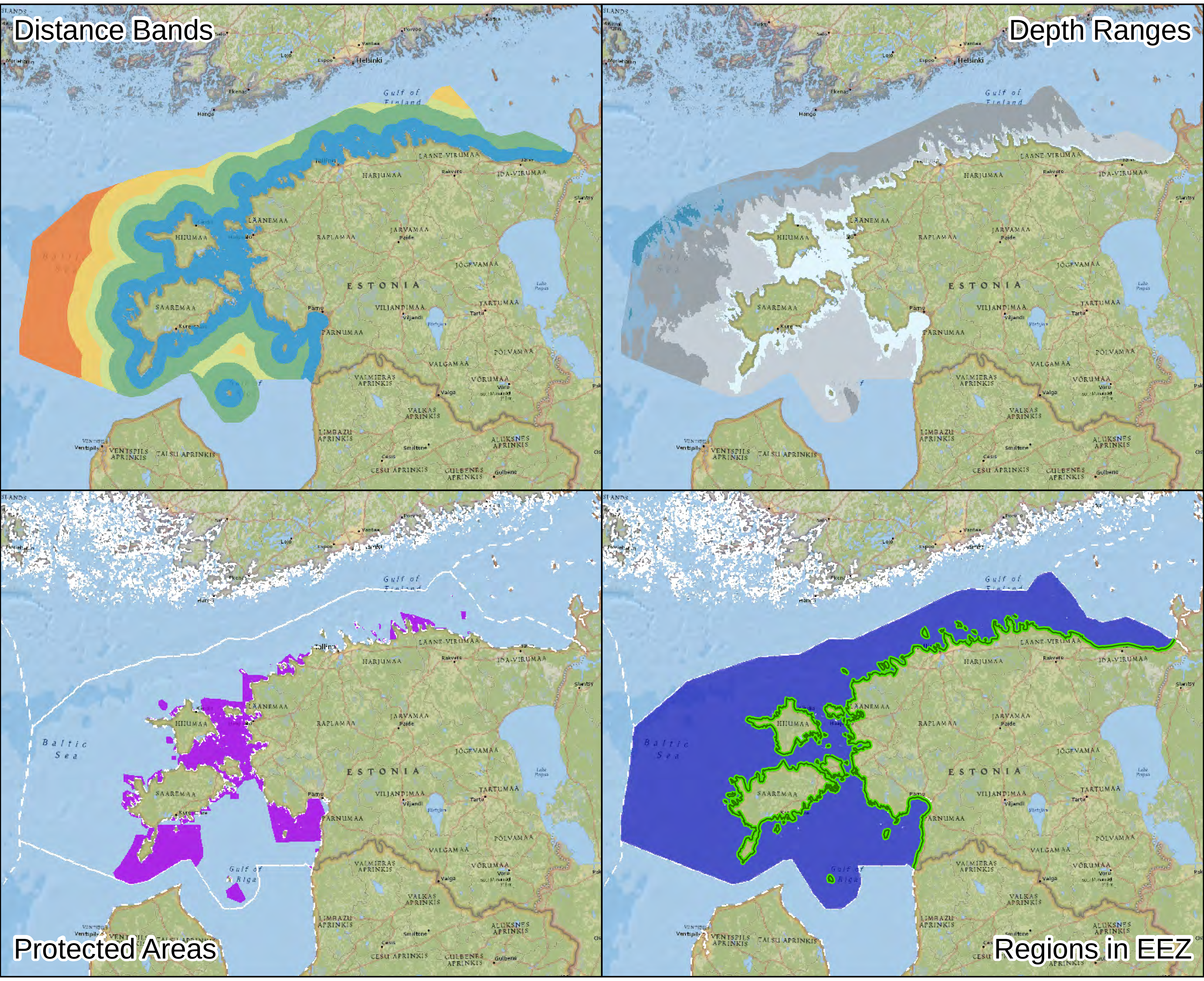

Distance Bands

- 0 to 5 NM
- 5 to 12 NM
- 12 to 16 NM
- 16 to 24 NM
- 24 to 200 NM

Depth Bands

- > -10m
- -10m to -50m
- -50m to -100m
- -100m to -150m
- -150m to -200m
- < -200m

Protected

- Protected Areas

Coastline Length

2,059 Km

Region

- Baltic

All Areas in Estonia EEZ: Cell Values = Area in Km^2 [% Depth Band (Row), % Distance Band (Column), % EEZ]

	0 to 5 NM	5 to 12 NM	12 to 16 NM	16 to 24 NM	24 to 200 NM	> 200 NM	Total
> -10m	5,172 [98%, 42%, 14%]	125 [2%, 1%, 0%]	1 [0%, 0%, 0%]	0 [0%, 0%, 0%]	0 [0%, 0%, 0%]	0 [0%, 0%, 0%]	5,298 [15% of Total]
-10m to -50m	5,627 [38%, 46%, 16%]	6,994 [47%, 62%, 19%]	1,472 [10%, 41%, 4%]	565 [4%, 16%, 2%]	190 [1%, 4%, 1%]	0 [0%, 0%, 0%]	14,848 [41% of Total]
-50m to -100m	1,456 [12%, 12%, 4%]	3,755 [32%, 34%, 10%]	1,627 [14%, 45%, 5%]	2,107 [18%, 58%, 6%]	2,742 [23%, 52%, 8%]	0 [0%, 0%, 0%]	11,687 [32% of Total]
-100m to -150m	40 [1%, 0%, 0%]	327 [9%, 3%, 1%]	508 [14%, 14%, 1%]	900 [25%, 25%, 3%]	1,769 [50%, 34%, 5%]	0 [0%, 0%, 0%]	3,544 [10% of Total]
-150m to -200m	0 [0%, 0%, 0%]	0 [0%, 0%, 0%]	13 [2%, 0%, 0%]	46 [8%, 1%, 0%]	546 [90%, 10%, 2%]	0 [0%, 0%, 0%]	605 [2% of Total]
<-200m	0 [0%, 0%, 0%]	0 [0%, 0%, 0%]	0 [0%, 0%, 0%]	0 [0%, 0%, 0%]	4 [100%, 0%, 0%]	0 [0%, 0%, 0%]	4 [0% of Total]
Total	12,294 [34% of Total]	11,202 [31% of Total]	3,621 [10% of Total]	3,618 [10% of Total]	5,251 [15% of Total]	0.000 [0% of Total]	35,986 Km^2

Areas in Estonia EEZ Excluding Protected Areas: Cell Values = Area in Km^2 [% Depth Band (Row), % Distance Band (Column), % EEZ]

6,533 [18%] Km^2 Protected	0 to 5 NM	5 to 12 NM	12 to 16 NM	16 to 24 NM	24 to 200 NM	> 200 NM	Total
> -10m	1,274 [99%, 18%, 4%]	11 [1%, 0%, 0%]	0.612 [0%, 0%, 0%]	0 [0%, 0%, 0%]	0 [0%, 0%, 0%]	0 [0%, 0%, 0%]	1,286 [4% of Total]
-10m to -50m	4,259 [34%, 61%, 14%]	5,951 [48%, 59%, 20%]	1,404 [11%, 40%, 5%]	565 [5%, 16%, 2%]	190 [2%, 4%, 1%]	0 [0%, 0%, 0%]	12,369 [42% of Total]
-50m to -100m	1,416 [12%, 20%, 5%]	3,754 [32%, 37%, 13%]	1,627 [14%, 46%, 6%]	2,107 [18%, 58%, 7%]	2,742 [24%, 52%, 9%]	0 [0%, 0%, 0%]	11,645 [40% of Total]
-100m to -150m	40 [1%, 1%, 0%]	327 [9%, 3%, 1%]	508 [14%, 14%, 2%]	900 [25%, 25%, 3%]	1,769 [50%, 34%, 6%]	0 [0%, 0%, 0%]	3,544 [12% of Total]
-150m to -200m	0 [0%, 0%, 0%]	0 [0%, 0%, 0%]	13 [2%, 0%, 0%]	46 [8%, 1%, 0%]	546 [90%, 10%, 2%]	0 [0%, 0%, 0%]	605 [2% of Total]
<-200m	0 [0%, 0%, 0%]	0 [0%, 0%, 0%]	0 [0%, 0%, 0%]	0 [0%, 0%, 0%]	4 [100%, 0%, 0%]	0 [0%, 0%, 0%]	4 [0% of Total]
Total	6,988 [24% of Total]	10,043 [34% of Total]	3,552 [12% of Total]	3,618 [12% of Total]	5,251 [18% of Total]	0.000 [0% of Total]	29,454 Km^2

The designations employed and the presentation of material in the map do not imply the expression of any opinion whatsoever on the part of FAO concerning the legal or constitutional status of any country, territory or sea area, or concerning the delimitation of frontiers.

Background reference map from National Geographic. Content may not reflect National Geographic's current map policy. Sources: National Geographic, Esri, DeLorme, HERE, UNEP-WCMC, USGS, NASA, ESA, METI, NRCAN, GEBCO, NOAA, increment P Corp.

Projection: Azimuthal Equidistant
Datum: WGS 1984
False Easting: 0.0000
False Northing: 0.0000
Central Meridian: 24.2085
Latitude Of Origin: 58.7906

N W E S

Food and Agriculture Organization of the United Nations

Finland: Baltic Region

©FAO, 2018

Kilometers
0 125 250 375 500 625

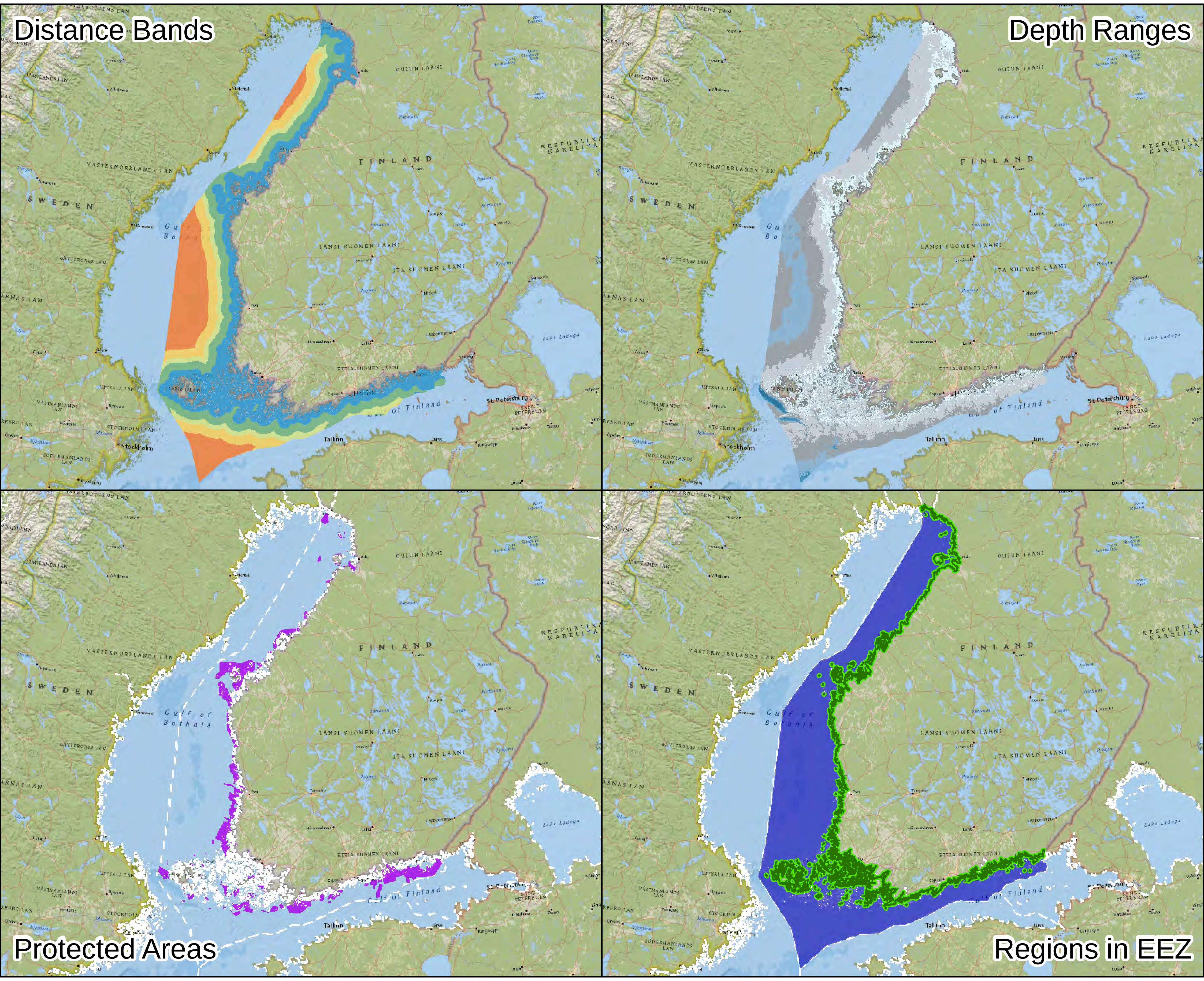

Distance Bands
- 0 to 5 NM
- 5 to 12 NM
- 12 to 16 NM
- 16 to 24 NM
- 24 to 200 NM

Depth Bands
- > -10m
- -10m to -50m
- -50m to -100m
- -100m to -150m
- -150m to -200m
- < -200m

Protected
- Protected Areas

Coastline Length
9,494 Km

Region
- Baltic

All Areas in Finland EEZ: Cell Values = Area in Km² [% Depth Band (Row), % Distance Band (Column), % EEZ]

	0 to 5 NM	5 to 12 NM	12 to 16 NM	16 to 24 NM	24 to 200 NM	> 200 NM	Total
> -10m	12,220 [92%, 46%, 15%]	1,048 [8%, 6%, 1%]	13 [0%, 0%, 0%]	2 [0%, 0%, 0%]	0 [0%, 0%, 0%]	0 [0%, 0%, 0%]	13,283 [17% of Total]
-10m to -50m	13,767 [41%, 52%, 17%]	13,219 [40%, 74%, 17%]	3,411 [10%, 43%, 4%]	2,506 [8%, 24%, 3%]	463 [1%, 3%, 1%]	0 [0%, 0%, 0%]	33,367 [42% of Total]
-50m to -100m	471 [2%, 2%, 1%]	3,031 [13%, 17%, 4%]	4,279 [18%, 54%, 5%]	7,630 [32%, 73%, 10%]	8,107 [34%, 48%, 10%]	0 [0%, 0%, 0%]	23,518 [29% of Total]
-100m to -150m	87 [1%, 0%, 0%]	159 [2%, 1%, 0%]	93 [1%, 1%, 0%]	341 [4%, 3%, 0%]	8,151 [92%, 48%, 10%]	0 [0%, 0%, 0%]	8,831 [11% of Total]
-150m to -200m	53 [10%, 0%, 0%]	179 [35%, 1%, 0%]	115 [22%, 1%, 0%]	12 [2%, 0%, 0%]	154 [30%, 1%, 0%]	0 [0%, 0%, 0%]	513 [1% of Total]
<-200m	74 [23%, 0%, 0%]	240 [74%, 1%, 0%]	5 [1%, 0%, 0%]	0.602 [0%, 0%, 0%]	6 [2%, 0%, 0%]	0 [0%, 0%, 0%]	326 [0% of Total]
Total	26,673 [33% of Total]	17,876 [22% of Total]	7,916 [10% of Total]	10,491 [13% of Total]	16,882 [21% of Total]	0.000 [0% of Total]	79,838 Km²

Areas in Finland EEZ Excluding Protected Areas: Cell Values = Area in Km² [% Depth Band (Row), % Distance Band (Column), % EEZ]

8,597 [11%] Km² Protected	0 to 5 NM	5 to 12 NM	12 to 16 NM	16 to 24 NM	24 to 200 NM	> 200 NM	Total
> -10m	8,720 [91%, 45%, 12%]	813 [9%, 5%, 1%]	10 [0%, 0%, 0%]	2 [0%, 0%, 0%]	0 [0%, 0%, 0%]	0 [0%, 0%, 0%]	9,545 [13% of Total]
-10m to -50m	10,280 [36%, 53%, 14%]	12,111 [42%, 74%, 17%]	3,339 [12%, 43%, 5%]	2,506 [9%, 24%, 4%]	463 [2%, 3%, 1%]	0 [0%, 0%, 0%]	28,700 [40% of Total]
-50m to -100m	360 [2%, 2%, 1%]	2,964 [13%, 18%, 4%]	4,270 [18%, 55%, 6%]	7,630 [33%, 73%, 11%]	8,107 [35%, 48%, 11%]	0 [0%, 0%, 0%]	23,331 [33% of Total]
-100m to -150m	87 [1%, 0%, 0%]	155 [2%, 1%, 0%]	93 [1%, 1%, 0%]	341 [4%, 3%, 0%]	8,151 [92%, 48%, 11%]	0 [0%, 0%, 0%]	8,827 [12% of Total]
-150m to -200m	53 [10%, 0%, 0%]	178 [35%, 1%, 0%]	115 [22%, 1%, 0%]	12 [2%, 0%, 0%]	154 [30%, 1%, 0%]	0 [0%, 0%, 0%]	513 [1% of Total]
<-200m	74 [23%, 0%, 0%]	240 [74%, 1%, 0%]	5 [1%, 0%, 0%]	0.602 [0%, 0%, 0%]	6 [2%, 0%, 0%]	0 [0%, 0%, 0%]	326 [0% of Total]
Total	19,575 [27% of Total]	16,462 [23% of Total]	7,832 [11% of Total]	10,491 [15% of Total]	16,882 [24% of Total]	0.000 [0% of Total]	71,241 Km²

The designations employed and the presentation of material in the map do not imply the expression of any opinion whatsoever on the part of FAO concerning the legal or constitutional status of any country, territory or sea area, or concerning the delimitation of frontiers.

Background reference map from National Geographic. Content may not reflect National Geographic's current map policy. Sources: National Geographic, Esri, DeLorme, HERE, UNEP-WCMC, USGS, NASA, ESA, METI, NRCAN, GEBCO, NOAA, increment P Corp.

Projection: Azimuthal Equidistant
Datum: WGS 1984
False Easting: 0.0000
False Northing: 0.0000
Central Meridian: 23.4572
Latitude Of Origin: 62.3342

N W E S

Food and Agriculture Organization of the United Nations

France: Atlantic and Mediterranean Regions

©FAO, 2018

Kilometers
0 125 250 375 500 625 750 875 1,000 1,125

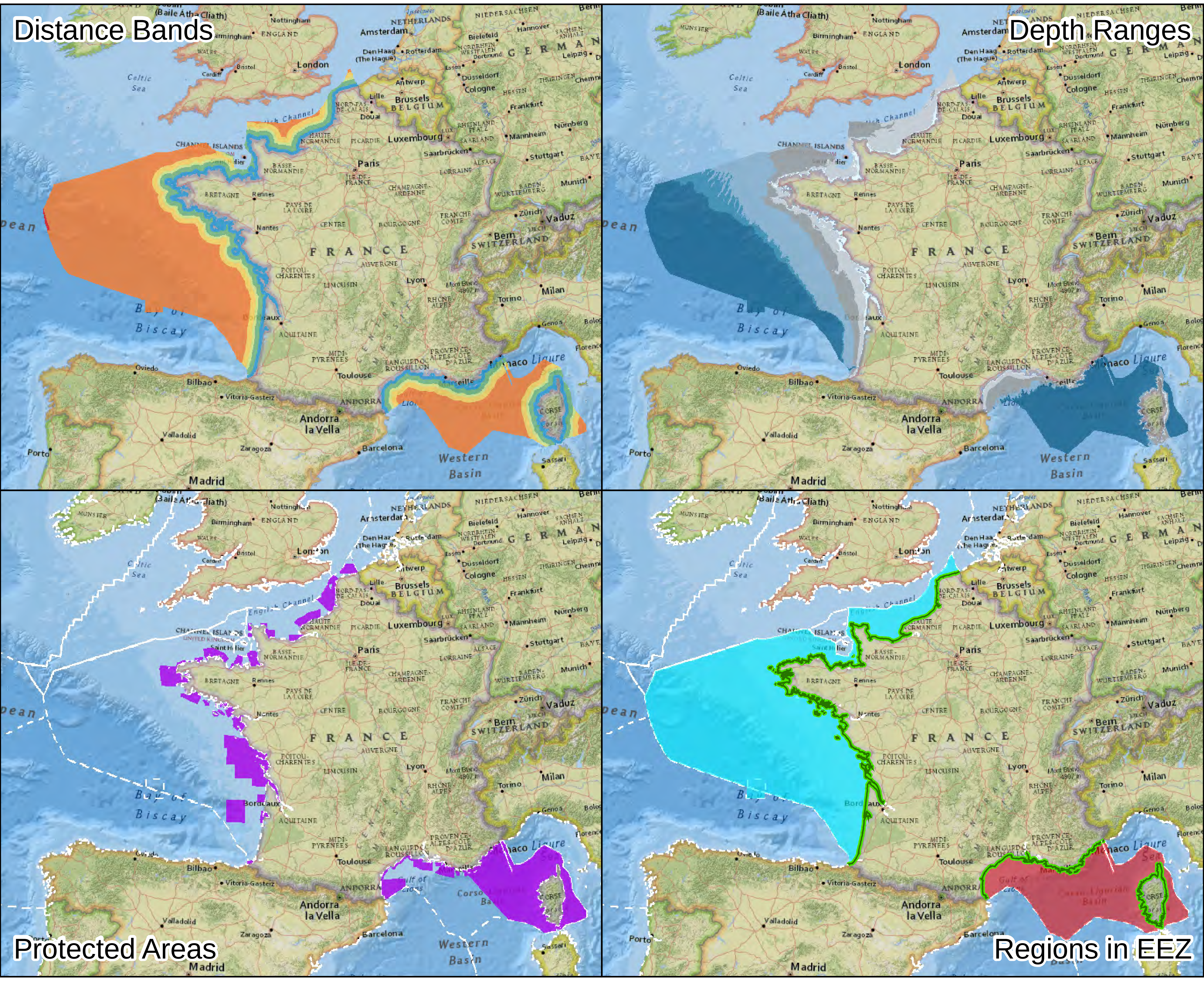

Distance Bands

0 to 5 NM
5 to 12 NM
12 to 16 NM
16 to 24 NM
24 to 200 NM
> 200 NM

Depth Bands

> -10m
-10m to -50m
-50m to -100m
-100m to -150m
-150m to -200m
< -200m

Protected

Protected Areas

Coastline Length

5,686 Km

Regions

Mediterranean
Atlantic

All Areas in France EEZ: Cell Values = Area in Km² [% Depth Band (Row), % Distance Band (Column), % EEZ]

	0 to 5 NM	5 to 12 NM	12 to 16 NM	16 to 24 NM	24 to 200 NM	> 200 NM	Total
> -10m	7,625 [97%, 23%, 2%]	197 [3%, 1%, 0%]	3 [0%, 0%, 0%]	0.470 [0%, 0%, 0%]	0 [0%, 0%, 0%]	0 [0%, 0%, 0%]	7,825 [2% of Total]
-10m to -50m	15,995 [40%, 49%, 5%]	12,601 [32%, 37%, 4%]	4,147 [10%, 24%, 1%]	4,628 [12%, 15%, 1%]	2,203 [6%, 1%, 1%]	0 [0%, 0%, 0%]	39,574 [11% of Total]
-50m to -100m	4,654 [11%, 14%, 1%]	10,641 [25%, 31%, 3%]	6,125 [14%, 36%, 2%]	10,315 [24%, 33%, 3%]	11,378 [26%, 5%, 3%]	0 [0%, 0%, 0%]	43,114 [13% of Total]
-100m to -150m	827 [1%, 3%, 0%]	2,110 [4%, 6%, 1%]	2,080 [3%, 12%, 1%]	6,846 [12%, 22%, 2%]	47,662 [80%, 21%, 14%]	0 [0%, 0%, 0%]	59,525 [17% of Total]
-150m to -200m	353 [2%, 1%, 0%]	254 [1%, 1%, 0%]	47 [0%, 0%, 0%]	65 [0%, 0%, 0%]	16,584 [96%, 7%, 5%]	0 [0%, 0%, 0%]	17,304 [5% of Total]
<-200m	3,073 [2%, 9%, 1%]	8,223 [5%, 24%, 2%]	4,720 [3%, 28%, 1%]	9,093 [5%, 29%, 3%]	151,349 [86%, 66%, 44%]	380 [0%, 100%, 0%]	176,839 [51% of Total]
Total	32,526 [9% of Total]	34,026 [10% of Total]	17,122 [5% of Total]	30,949 [9% of Total]	229,177 [67% of Total]	380 [0% of Total]	344,179 Km²

Areas in France EEZ Excluding Protected Areas: Cell Values = Area in Km² [% Depth Band (Row), % Distance Band (Column), % EEZ]

87,574 [25%] Km² Protected	0 to 5 NM	5 to 12 NM	12 to 16 NM	16 to 24 NM	24 to 200 NM	> 200 NM	Total
> -10m	1,711 [97%, 20%, 1%]	49 [3%, 0%, 0%]	2 [0%, 0%, 0%]	0.470 [0%, 0%, 0%]	0 [0%, 0%, 0%]	0 [0%, 0%, 0%]	1,763 [1% of Total]
-10m to -50m	5,510 [29%, 63%, 2%]	5,801 [31%, 40%, 2%]	2,389 [13%, 24%, 1%]	3,308 [17%, 16%, 1%]	1,968 [10%, 1%, 1%]	0 [0%, 0%, 0%]	18,976 [7% of Total]
-50m to -100m	1,307 [4%, 15%, 1%]	6,896 [22%, 48%, 3%]	5,187 [17%, 53%, 2%]	8,329 [27%, 41%, 3%]	8,947 [29%, 4%, 3%]	0 [0%, 0%, 0%]	30,666 [12% of Total]
-100m to -150m	54 [0%, 1%, 0%]	982 [2%, 7%, 0%]	1,632 [3%, 17%, 1%]	6,434 [12%, 32%, 3%]	46,269 [84%, 23%, 18%]	0 [0%, 0%, 0%]	55,371 [22% of Total]
-150m to -200m	13 [0%, 0%, 0%]	22 [0%, 0%, 0%]	4 [0%, 0%, 0%]	56 [0%, 0%, 0%]	16,317 [99%, 8%, 6%]	0 [0%, 0%, 0%]	16,412 [6% of Total]
<-200m	87 [0%, 1%, 0%]	731 [1%, 5%, 0%]	649 [0%, 7%, 0%]	2,006 [2%, 10%, 1%]	129,566 [97%, 64%, 50%]	380 [0%, 100%, 0%]	133,418 [52% of Total]
Total	8,682 [3% of Total]	14,482 [6% of Total]	9,863 [4% of Total]	20,133 [8% of Total]	203,067 [79% of Total]	380 [0% of Total]	256,606 Km²

The designations employed and the presentation of material in the map do not imply the expression of any opinion whatsoever on the part of FAO concerning the legal or constitutional status of any country, territory or sea area, or concerning the delimitation of frontiers.

Background reference map from National Geographic. Content may not reflect National Geographic's current map policy. Sources: National Geographic, Esri, DeLorme, HERE, UNEP-WCMC, USGS, NASA, ESA, METI, NRCAN, GEBCO, NOAA, increment P Corp.

Projection: Azimuthal Equidistant
Datum: WGS 1984
False Easting: 0.0000
False Northing: 0.0000
Central Meridian: 0.1705
Latitude Of Origin: 46.4006

Food and Agriculture Organization of the United Nations

France: Caribbean Region

©FAO, 2018

Kilometers
0 125 250 375 500 625 750 875 1,0001,125

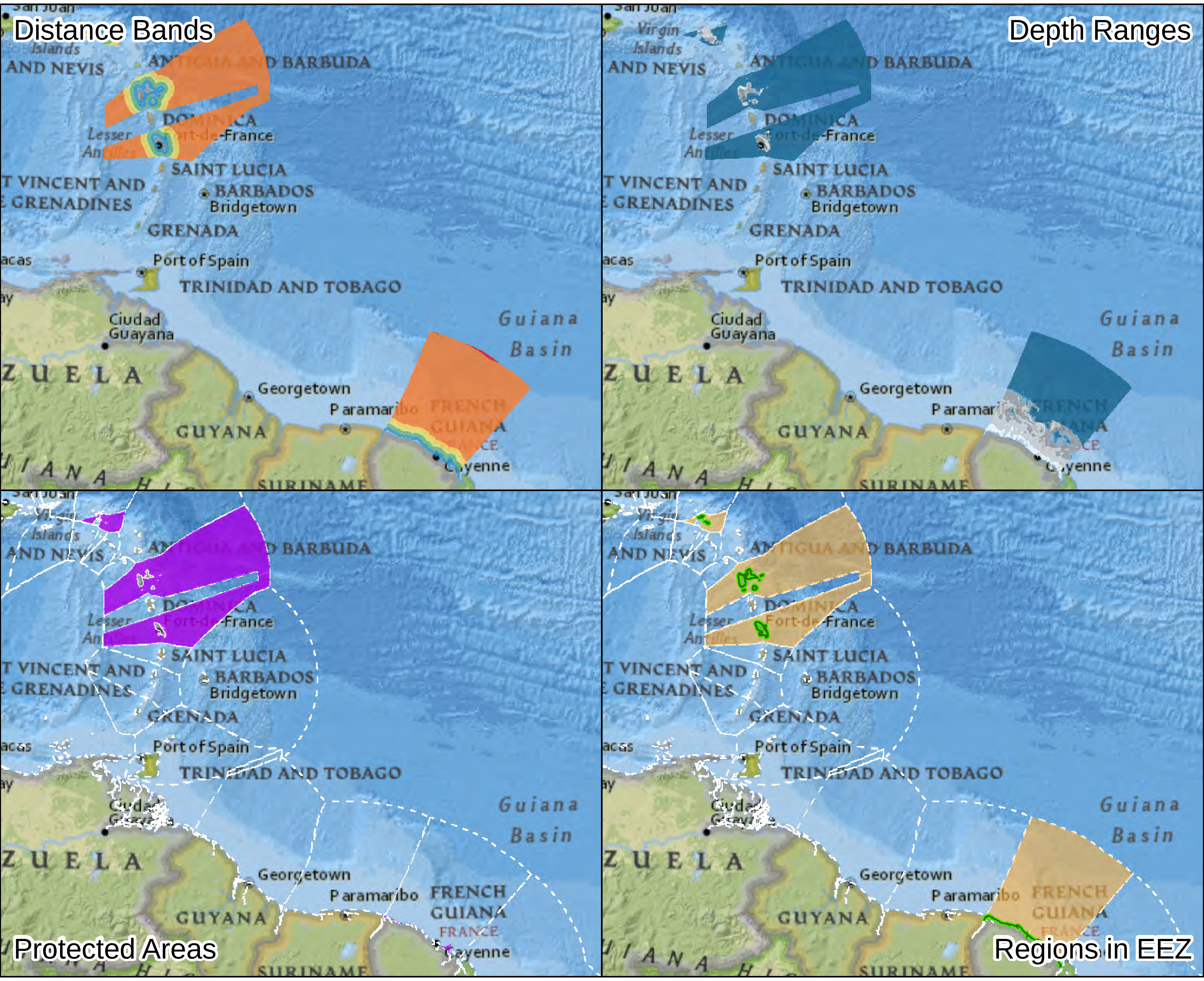

Distance Bands

- 0 to 5 NM
- 5 to 12 NM
- 12 to 16 NM
- 16 to 24 NM
- 24 to 200 NM
- > 200 NM

Depth Bands

- > -10m
- -10m to -50m
- -50m to -100m
- -100m to -150m
- -150m to -200m
- < -200m

Protected

- Protected Areas

Coastline Length

2,932 Km

Region

- Caribbean

All Areas in France EEZ: Cell Values = Area in Km^2 [% Depth Band (Row), % Distance Band (Column), % EEZ]

	0 to 5 NM	5 to 12 NM	12 to 16 NM	16 to 24 NM	24 to 200 NM	> 200 NM	Total
> -10m	3,243 [57%, 29%, 1%]	1,959 [35%, 14%, 1%]	165 [3%, 2%, 0%]	143 [3%, 1%, 0%]	157 [3%, 0%, 0%]	0 [0%, 0%, 0%]	5,667 [2% of Total]
-10m to -50m	2,544 [13%, 23%, 1%]	2,810 [14%, 20%, 1%]	2,220 [11%, 30%, 1%]	3,940 [20%, 29%, 1%]	8,086 [41%, 4%, 3%]	0 [0%, 0%, 0%]	19,601 [7% of Total]
-50m to -100m	982 [7%, 9%, 0%]	422 [3%, 3%, 0%]	191 [1%, 3%, 0%]	754 [6%, 6%, 0%]	11,344 [83%, 5%, 4%]	0 [0%, 0%, 0%]	13,693 [5% of Total]
-100m to -150m	441 [8%, 4%, 0%]	162 [3%, 1%, 0%]	49 [1%, 1%, 0%]	40 [1%, 0%, 0%]	4,641 [87%, 2%, 2%]	0 [0%, 0%, 0%]	5,334 [2% of Total]
-150m to -200m	371 [12%, 3%, 0%]	181 [6%, 1%, 0%]	41 [1%, 1%, 0%]	29 [1%, 0%, 0%]	2,353 [79%, 1%, 1%]	0 [0%, 0%, 0%]	2,975 [1% of Total]
<-200m	3,463 [2%, 31%, 1%]	8,602 [4%, 61%, 3%]	4,754 [2%, 64%, 2%]	8,753 [4%, 64%, 3%]	202,389 [88%, 88%, 73%]	752 [0%, 100%, 0%]	228,713 [83% of Total]
Total	11,045 [4% of Total]	14,136 [5% of Total]	7,421 [3% of Total]	13,660 [5% of Total]	228,969 [83% of Total]	752 [0% of Total]	275,982 Km^2

Areas in France EEZ Excluding Protected Areas: Cell Values = Area in Km^2 [% Depth Band (Row), % Distance Band (Column), % EEZ]

144,023 [52%] Km^2 Protected	0 to 5 NM	5 to 12 NM	12 to 16 NM	16 to 24 NM	24 to 200 NM	> 200 NM	Total
> -10m	2,282 [49%, 73%, 2%]	1,951 [42%, 45%, 1%]	141 [3%, 6%, 0%]	141 [3%, 3%, 0%]	157 [3%, 0%, 0%]	0 [0%, 0%, 0%]	4,674 [4% of Total]
-10m to -50m	833 [5%, 27%, 1%]	2,320 [13%, 53%, 2%]	2,125 [12%, 89%, 2%]	3,915 [23%, 83%, 3%]	8,086 [47%, 7%, 6%]	0 [0%, 0%, 0%]	17,279 [13% of Total]
-50m to -100m	11 [0%, 0%, 0%]	6 [0%, 0%, 0%]	100 [1%, 4%, 0%]	646 [5%, 14%, 0%]	11,334 [94%, 10%, 9%]	0 [0%, 0%, 0%]	12,097 [9% of Total]
-100m to -150m	0.002 [0%, 0%, 0%]	0 [0%, 0%, 0%]	0.000 [0%, 0%, 0%]	0.000 [0%, 0%, 0%]	4,634 [100%, 4%, 4%]	0 [0%, 0%, 0%]	4,634 [4% of Total]
-150m to -200m	0.721 [0%, 0%, 0%]	0.003 [0%, 0%, 0%]	0 [0%, 0%, 0%]	0 [0%, 0%, 0%]	2,346 [100%, 2%, 2%]	0 [0%, 0%, 0%]	2,346 [2% of Total]
<-200m	0.167 [0%, 0%, 0%]	72 [0%, 2%, 0%]	13 [0%, 1%, 0%]	27 [0%, 1%, 0%]	90,067 [99%, 77%, 68%]	750 [1%, 100%, 1%]	90,929 [69% of Total]
Total	3,128 [2% of Total]	4,349 [3% of Total]	2,380 [2% of Total]	4,730 [4% of Total]	116,624 [88% of Total]	750 [1% of Total]	131,959 Km^2

The designations employed and the presentation of material in the map do not imply the expression of any opinion whatsoever on the part of FAO concerning the legal or constitutional status of any country, territory or sea area, or concerning the delimitation of frontiers.

Background reference map from National Geographic. Content may not reflect National Geographic's current map policy. Sources: National Geographic, Esri, DeLorme, HERE, UNEP-WCMC, USGS, NASA, ESA, METI, NRCAN, GEBCO, NOAA, increment P Corp.

Projection: Azimuthal Equidistant
Datum: WGS 1984
False Easting: 0.0000
False Northing: 0.0000
Central Meridian: -56.5201
Latitude Of Origin: 11.3630

©FAO, 2018

Food and Agriculture Organization of the United Nations

Georgia: Black Sea Region

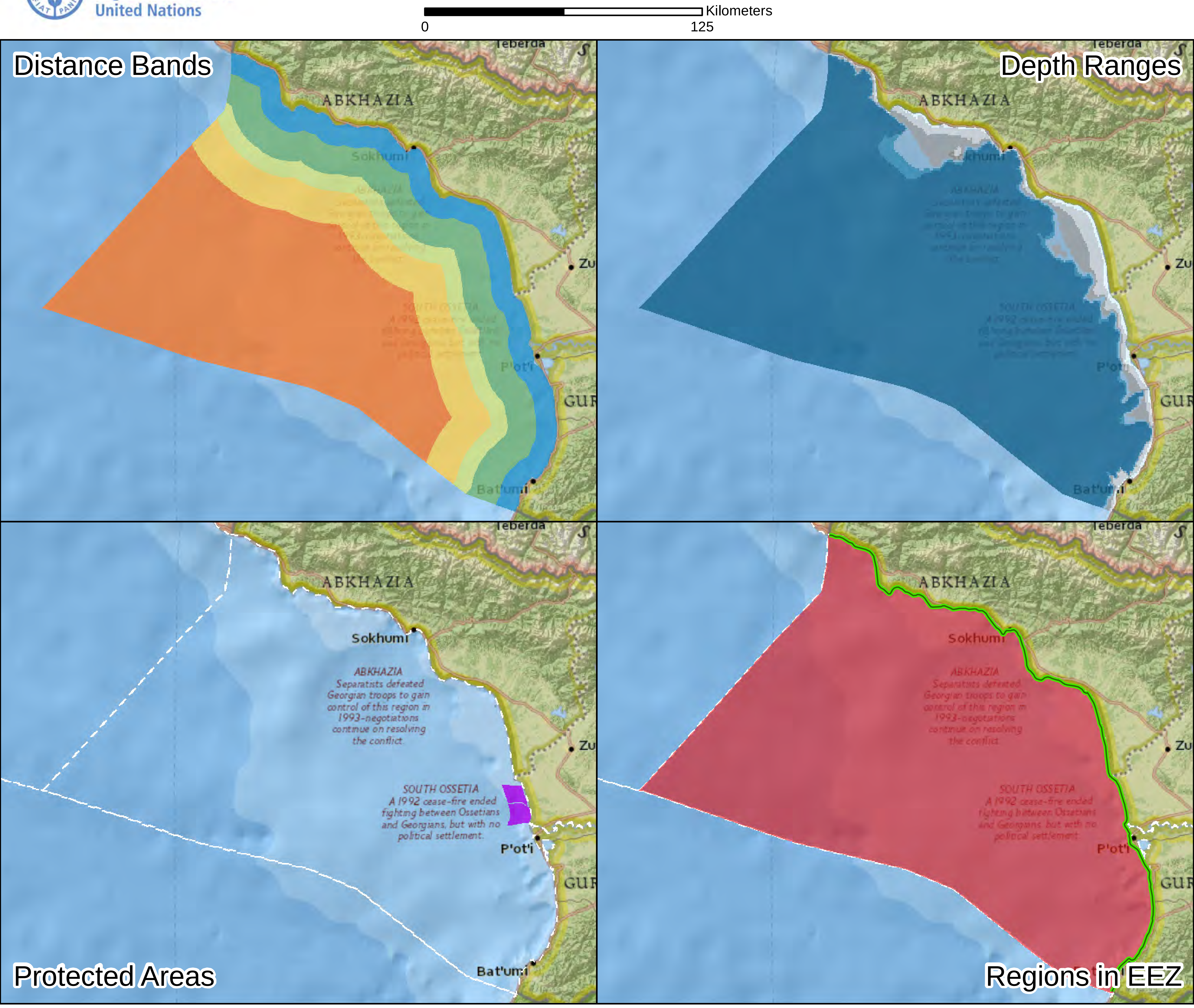

Distance Bands	Depth Bands	Protected	Region
0 to 5 NM	> -10m	Protected Areas	Mediterranean
5 to 12 NM	-10m to -50m		
12 to 16 NM	-50m to -100m		
16 to 24 NM	-100m to -150m		
24 to 200 NM	-150m to -200m		
	< -200m		

Coastline Length: 311 Km

All Areas in Georgia EEZ: Cell Values = Area in Km^2 [% Depth Band (Row), % Distance Band (Column), % EEZ]

	0 to 5 NM	5 to 12 NM	12 to 16 NM	16 to 24 NM	24 to 200 NM	> 200 NM	Total
> -10m	206 [100%, 7%, 1%]	0 [0%, 0%, 0%]	0 [0%, 0%, 0%]	0 [0%, 0%, 0%]	0 [0%, 0%, 0%]	0 [0%, 0%, 0%]	206 [1% of Total]
-10m to -50m	630 [98%, 23%, 3%]	11 [2%, 0%, 0%]	0 [0%, 0%, 0%]	0 [0%, 0%, 0%]	0 [0%, 0%, 0%]	0 [0%, 0%, 0%]	641 [3% of Total]
-50m to -100m	516 [67%, 19%, 2%]	250 [33%, 7%, 1%]	0 [0%, 0%, 0%]	0 [0%, 0%, 0%]	0 [0%, 0%, 0%]	0 [0%, 0%, 0%]	766 [3% of Total]
-100m to -150m	171 [36%, 6%, 1%]	301 [64%, 9%, 1%]	0.702 [0%, 0%, 0%]	0 [0%, 0%, 0%]	0 [0%, 0%, 0%]	0 [0%, 0%, 0%]	473 [2% of Total]
-150m to -200m	112 [32%, 4%, 0%]	194 [54%, 6%, 1%]	50 [14%, 3%, 0%]	0 [0%, 0%, 0%]	0 [0%, 0%, 0%]	0 [0%, 0%, 0%]	356 [2% of Total]
<-200m	1,117 [5%, 41%, 5%]	2,727 [13%, 78%, 12%]	1,768 [9%, 97%, 8%]	3,310 [16%, 100%, 15%]	11,443 [56%, 100%, 50%]	0 [0%, 0%, 0%]	20,365 [89% of Total]
Total	2,753 [12% of Total]	3,482 [15% of Total]	1,819 [8% of Total]	3,310 [15% of Total]	11,443 [50% of Total]	0.000 [0% of Total]	22,808 Km^2

Areas in Georgia EEZ Excluding Protected Areas: Cell Values = Area in Km^2 [% Depth Band (Row), % Distance Band (Column), % EEZ]

152 [1%] Km^2 Protected	0 to 5 NM	5 to 12 NM	12 to 16 NM	16 to 24 NM	24 to 200 NM	> 200 NM	Total
> -10m	194 [100%, 7%, 1%]	0 [0%, 0%, 0%]	0 [0%, 0%, 0%]	0 [0%, 0%, 0%]	0 [0%, 0%, 0%]	0 [0%, 0%, 0%]	194 [1% of Total]
-10m to -50m	594 [98%, 23%, 3%]	11 [2%, 0%, 0%]	0 [0%, 0%, 0%]	0 [0%, 0%, 0%]	0 [0%, 0%, 0%]	0 [0%, 0%, 0%]	605 [3% of Total]
-50m to -100m	493 [66%, 19%, 2%]	250 [34%, 7%, 1%]	0 [0%, 0%, 0%]	0 [0%, 0%, 0%]	0 [0%, 0%, 0%]	0 [0%, 0%, 0%]	743 [3% of Total]
-100m to -150m	155 [34%, 6%, 1%]	301 [66%, 9%, 1%]	0.702 [0%, 0%, 0%]	0 [0%, 0%, 0%]	0 [0%, 0%, 0%]	0 [0%, 0%, 0%]	457 [2% of Total]
-150m to -200m	101 [29%, 4%, 0%]	194 [56%, 6%, 1%]	50 [14%, 3%, 0%]	0 [0%, 0%, 0%]	0 [0%, 0%, 0%]	0 [0%, 0%, 0%]	345 [2% of Total]
<-200m	1,064 [5%, 41%, 5%]	2,726 [13%, 78%, 12%]	1,768 [9%, 97%, 8%]	3,310 [16%, 100%, 15%]	11,443 [56%, 100%, 51%]	0 [0%, 0%, 0%]	20,312 [90% of Total]
Total	2,602 [11% of Total]	3,482 [15% of Total]	1,819 [8% of Total]	3,310 [15% of Total]	11,443 [51% of Total]	0.000 [0% of Total]	22,656 Km^2

The designations employed and the presentation of material in the map do not imply the expression of any opinion whatsoever on the part of FAO concerning the legal or constitutional status of any country, territory or sea area, or concerning the delimitation of frontiers.

Background reference map from National Geographic. Content may not reflect National Geographic's current map policy. Sources: National Geographic, Esri, DeLorme, HERE, UNEP-WCMC, USGS, NASA, ESA, METI, NRCAN, GEBCO, NOAA, increment P Corp.

Projection: Azimuthal Equidistant
Datum: WGS 1984
False Easting: 0.0000
False Northing: 0.0000
Central Meridian: 40.3762
Latitude Of Origin: 42.4522

Food and Agriculture Organization of the United Nations

Germany: North Sea and Baltic Regions

©FAO, 2018

Kilometers
0 125 250 375 500

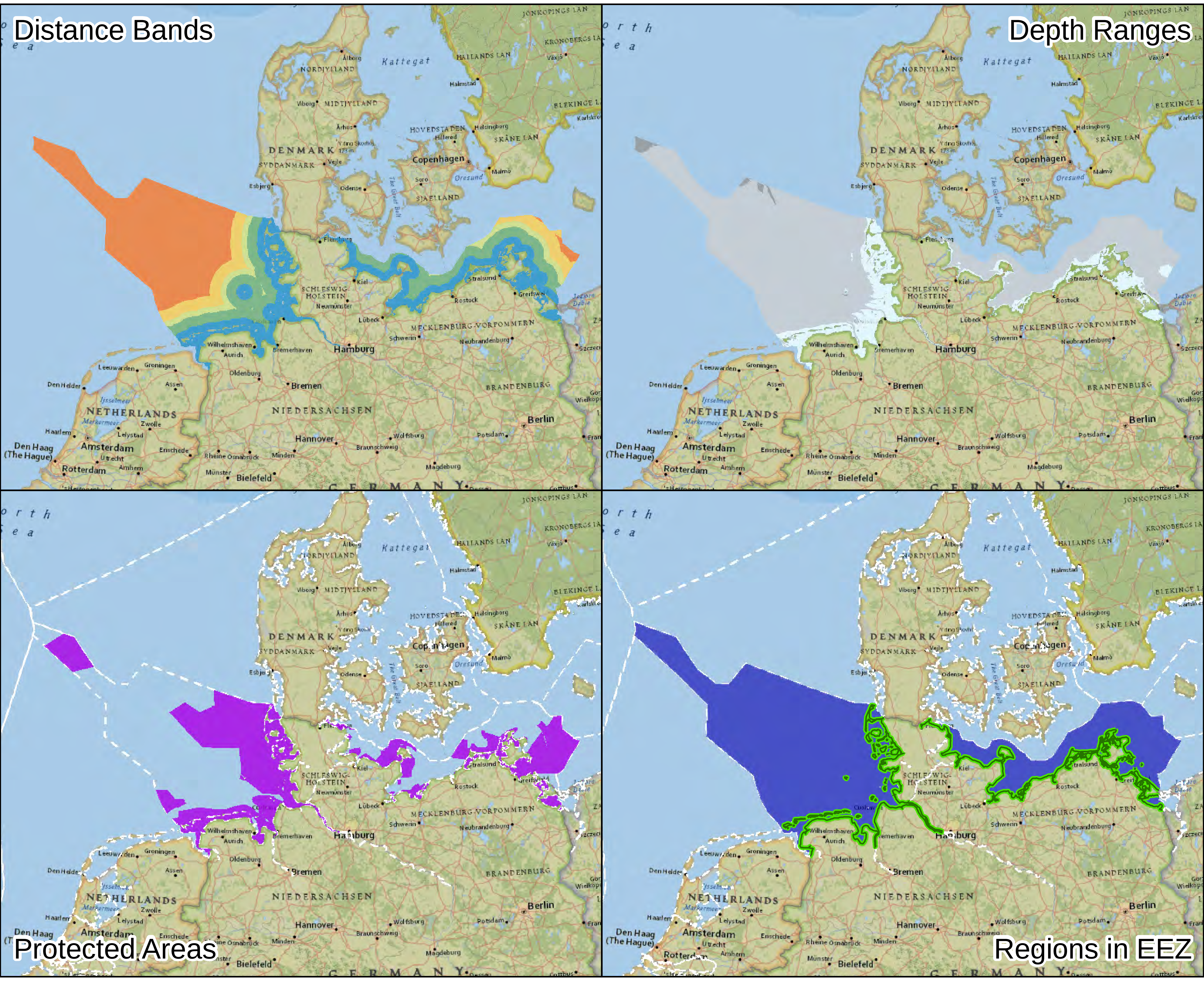

Distance Bands
- 0 to 5 NM
- 5 to 12 NM
- 12 to 16 NM
- 16 to 24 NM
- 24 to 200 NM

Depth Bands
- > -10m
- -10m to -50m
- -50m to -100m

Protected
- Protected Areas

Coastline Length
- 3,329 Km

Region
- Baltic

All Areas in Germany EEZ: Cell Values = Area in Km^2 [% Depth Band (Row), % Distance Band (Column), % EEZ]

	0 to 5 NM	5 to 12 NM	12 to 16 NM	16 to 24 NM	24 to 200 NM	> 200 NM	Total
> -10m	8,488 [92%, 60%, 15%]	556 [6%, 6%, 1%]	67 [1%, 2%, 0%]	141 [2%, 3%, 0%]	0 [0%, 0%, 0%]	0 [0%, 0%, 0%]	9,252 [17% of Total]
-10m to -50m	5,623 [12%, 40%, 10%]	9,007 [20%, 94%, 16%]	3,117 [7%, 98%, 6%]	4,842 [11%, 97%, 9%]	23,332 [51%, 97%, 42%]	0 [0%, 0%, 0%]	45,922 [82% of Total]
-50m to -100m	10 [2%, 0%, 0%]	0 [0%, 0%, 0%]	0 [0%, 0%, 0%]	0 [0%, 0%, 0%]	618 [98%, 3%, 1%]	0 [0%, 0%, 0%]	628 [1% of Total]
-100m to -150m	0 [0%, 0%, 0%]	0 [0%, 0%, 0%]	0 [0%, 0%, 0%]	0 [0%, 0%, 0%]	0 [0%, 0%, 0%]	0 [0%, 0%, 0%]	0.000 [0% of Total]
-150m to -200m	0 [0%, 0%, 0%]	0 [0%, 0%, 0%]	0 [0%, 0%, 0%]	0 [0%, 0%, 0%]	0 [0%, 0%, 0%]	0 [0%, 0%, 0%]	0.000 [0% of Total]
<-200m	0 [0%, 0%, 0%]	0 [0%, 0%, 0%]	0 [0%, 0%, 0%]	0 [0%, 0%, 0%]	0 [0%, 0%, 0%]	0 [0%, 0%, 0%]	0.000 [0% of Total]
Total	14,121 [25% of Total]	9,563 [17% of Total]	3,185 [6% of Total]	4,983 [9% of Total]	23,951 [43% of Total]	0.000 [0% of Total]	55,802 Km^2

Areas in Germany EEZ Excluding Protected Areas: Cell Values = Area in Km^2 [% Depth Band (Row), % Distance Band (Column), % EEZ]

25,120 [45%] Km^2 Protected	0 to 5 NM	5 to 12 NM	12 to 16 NM	16 to 24 NM	24 to 200 NM	> 200 NM	Total
> -10m	482 [96%, 15%, 2%]	21 [4%, 0%, 0%]	0.011 [0%, 0%, 0%]	0.118 [0%, 0%, 0%]	0 [0%, 0%, 0%]	0 [0%, 0%, 0%]	503 [2% of Total]
-10m to -50m	2,692 [9%, 85%, 9%]	5,112 [17%, 100%, 17%]	1,722 [6%, 100%, 6%]	2,651 [9%, 100%, 9%]	17,374 [59%, 97%, 57%]	0 [0%, 0%, 0%]	29,551 [96% of Total]
-50m to -100m	10 [2%, 0%, 0%]	0 [0%, 0%, 0%]	0 [0%, 0%, 0%]	0 [0%, 0%, 0%]	618 [98%, 3%, 2%]	0 [0%, 0%, 0%]	628 [2% of Total]
-100m to -150m	0 [0%, 0%, 0%]	0 [0%, 0%, 0%]	0 [0%, 0%, 0%]	0 [0%, 0%, 0%]	0 [0%, 0%, 0%]	0 [0%, 0%, 0%]	0.000 [0% of Total]
-150m to -200m	0 [0%, 0%, 0%]	0 [0%, 0%, 0%]	0 [0%, 0%, 0%]	0 [0%, 0%, 0%]	0 [0%, 0%, 0%]	0 [0%, 0%, 0%]	0.000 [0% of Total]
<-200m	0 [0%, 0%, 0%]	0 [0%, 0%, 0%]	0 [0%, 0%, 0%]	0 [0%, 0%, 0%]	0 [0%, 0%, 0%]	0 [0%, 0%, 0%]	0.000 [0% of Total]
Total	3,183 [10% of Total]	5,133 [17% of Total]	1,722 [6% of Total]	2,651 [9% of Total]	17,993 [59% of Total]	0.000 [0% of Total]	30,682 Km^2

The designations employed and the presentation of material in the map do not imply the expression of any opinion whatsoever on the part of FAO concerning the legal or constitutional status of any country, territory or sea area, or concerning the delimitation of frontiers.

Background reference map from National Geographic. Content may not reflect National Geographic's current map policy. Sources: National Geographic, Esri, DeLorme, HERE, UNEP-WCMC, USGS, NASA, ESA, METI, NRCAN, GEBCO, NOAA, increment P Corp.

Projection: Azimuthal Equidistant
Datum: WGS 1984
False Easting: 0.0000
False Northing: 0.0000
Central Meridian: 9.0500
Latitude Of Origin: 54.5817

N W E S

Greece: Mediterranean Region

Food and Agriculture Organization of the United Nations

©FAO, 2018

0 125 250 375 500 625 Kilometers

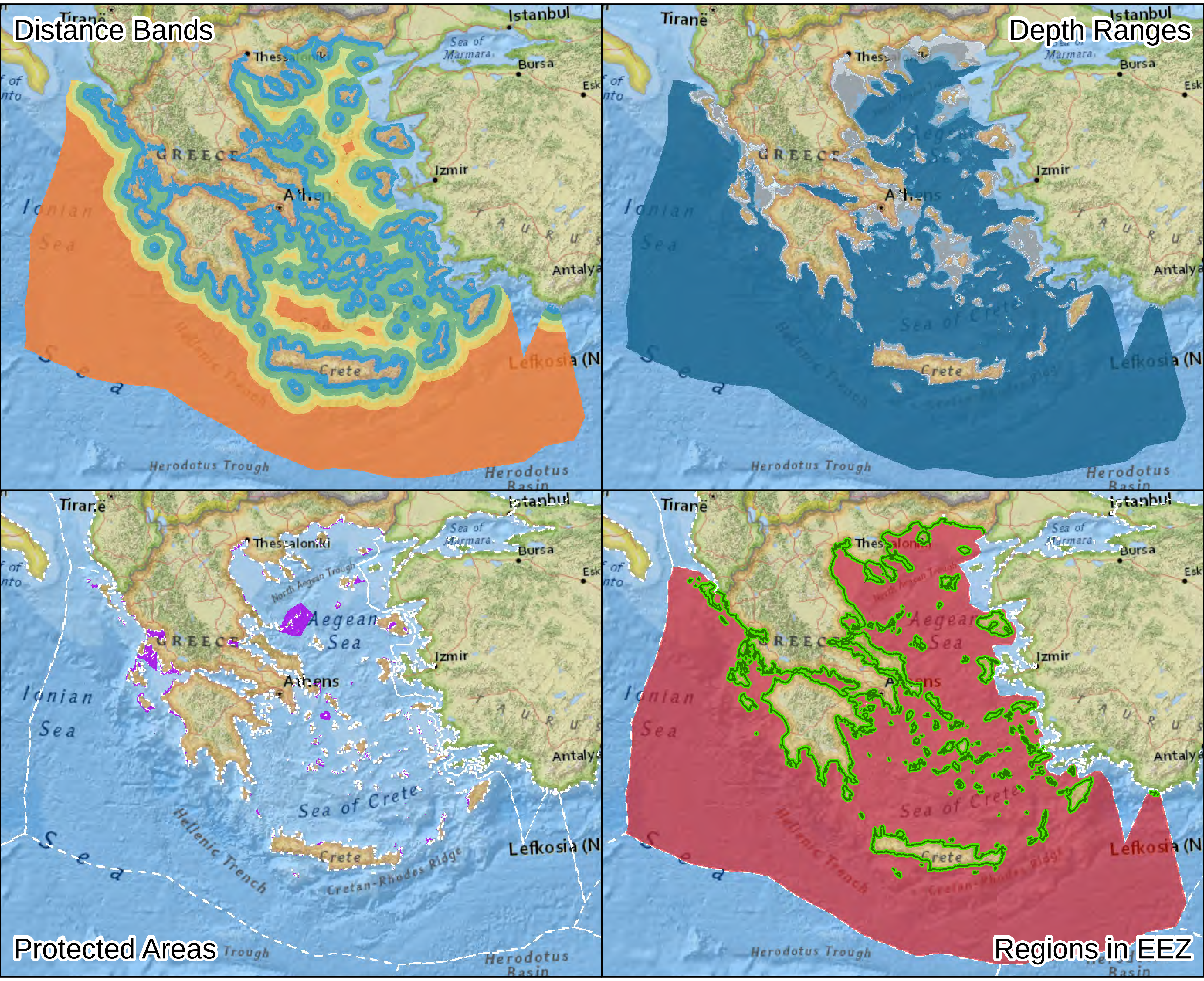

Distance Bands

- 0 to 5 NM
- 5 to 12 NM
- 12 to 16 NM
- 16 to 24 NM
- 24 to 200 NM

Depth Bands

- > -10m
- -10m to -50m
- -50m to -100m
- -100m to -150m
- -150m to -200m
- < -200m

Protected

- Protected Areas

Region

- Mediterranean

Coastline Length

11,695 Km

All Areas in Greece EEZ: Cell Values = Area in Km² [% Depth Band (Row), % Distance Band (Column), % EEZ]

	0 to 5 NM	5 to 12 NM	12 to 16 NM	16 to 24 NM	24 to 200 NM	> 200 NM	Total
> -10m	4,919 [100%, 5%, 1%]	14 [0%, 0%, 0%]	0.690 [0%, 0%, 0%]	0 [0%, 0%, 0%]	0 [0%, 0%, 0%]	0 [0%, 0%, 0%]	4,933 [1% of Total]
-10m to -50m	10,392 [92%, 11%, 2%]	881 [8%, 1%, 0%]	0.677 [0%, 0%, 0%]	3 [0%, 0%, 0%]	0 [0%, 0%, 0%]	0 [0%, 0%, 0%]	11,277 [2% of Total]
-50m to -100m	17,868 [76%, 20%, 4%]	4,896 [21%, 6%, 1%]	563 [2%, 2%, 0%]	48 [0%, 0%, 0%]	0.667 [0%, 0%, 0%]	0 [0%, 0%, 0%]	23,376 [5% of Total]
-100m to -150m	10,481 [69%, 12%, 2%]	3,932 [26%, 5%, 1%]	460 [3%, 1%, 0%]	380 [2%, 1%, 0%]	13 [0%, 0%, 0%]	0 [0%, 0%, 0%]	15,267 [3% of Total]
-150m to -200m	7,123 [63%, 8%, 1%]	2,876 [25%, 3%, 1%]	828 [7%, 3%, 0%]	428 [4%, 1%, 0%]	50 [0%, 0%, 0%]	0 [0%, 0%, 0%]	11,305 [2% of Total]
<-200m	40,312 [10%, 44%, 8%]	74,060 [18%, 85%, 15%]	29,808 [7%, 94%, 6%]	39,339 [9%, 98%, 8%]	231,219 [56%, 100%, 48%]	0 [0%, 0%, 0%]	414,739 [86% of Total]
Total	91,096 [19% of Total]	86,658 [18% of Total]	31,661 [7% of Total]	40,199 [8% of Total]	231,283 [48% of Total]	0.000 [0% of Total]	480,897 Km²

Areas in Greece EEZ Excluding Protected Areas: Cell Values = Area in Km² [% Depth Band (Row), % Distance Band (Column), % EEZ]

6,859 [1%] Km² Protected	0 to 5 NM	5 to 12 NM	12 to 16 NM	16 to 24 NM	24 to 200 NM	> 200 NM	Total
> -10m	3,387 [100%, 4%, 1%]	5 [0%, 0%, 0%]	0.183 [0%, 0%, 0%]	0 [0%, 0%, 0%]	0 [0%, 0%, 0%]	0 [0%, 0%, 0%]	3,392 [1% of Total]
-10m to -50m	8,946 [91%, 11%, 2%]	858 [9%, 1%, 0%]	0.677 [0%, 0%, 0%]	0.107 [0%, 0%, 0%]	0 [0%, 0%, 0%]	0 [0%, 0%, 0%]	9,805 [2% of Total]
-50m to -100m	17,113 [76%, 20%, 4%]	4,895 [22%, 6%, 1%]	563 [2%, 2%, 0%]	42 [0%, 0%, 0%]	0.667 [0%, 0%, 0%]	0 [0%, 0%, 0%]	22,614 [5% of Total]
-100m to -150m	9,835 [67%, 12%, 2%]	3,932 [27%, 5%, 1%]	460 [3%, 1%, 0%]	376 [3%, 1%, 0%]	13 [0%, 0%, 0%]	0 [0%, 0%, 0%]	14,616 [3% of Total]
-150m to -200m	6,631 [61%, 8%, 1%]	2,876 [27%, 3%, 1%]	828 [8%, 3%, 0%]	427 [4%, 1%, 0%]	50 [0%, 0%, 0%]	0 [0%, 0%, 0%]	10,812 [2% of Total]
<-200m	38,792 [9%, 46%, 8%]	73,644 [18%, 85%, 16%]	29,807 [7%, 94%, 6%]	39,336 [10%, 98%, 8%]	231,219 [56%, 100%, 49%]	0 [0%, 0%, 0%]	412,799 [87% of Total]
Total	84,704 [18% of Total]	86,210 [18% of Total]	31,660 [7% of Total]	40,181 [8% of Total]	231,283 [49% of Total]	0.000 [0% of Total]	474,038 Km²

The designations employed and the presentation of material in the map do not imply the expression of any opinion whatsoever on the part of FAO concerning the legal or constitutional status of any country, territory or sea area, or concerning the delimitation of frontiers.

Background reference map from National Geographic. Content may not reflect National Geographic's current map policy. Sources: National Geographic, Esri, DeLorme, HERE, UNEP-WCMC, USGS, NASA, ESA, METI, NRCAN, GEBCO, NOAA, increment P Corp.

Projection: Azimuthal Equidistant
Datum: WGS 1984
False Easting: 0.0000
False Northing: 0.0000
Central Meridian: 24.1826
Latitude Of Origin: 37.1464

N W E S

Food and Agriculture Organization of the United Nations

Grenada: Caribbean Region

©FAO, 2018

Kilometers
0 125

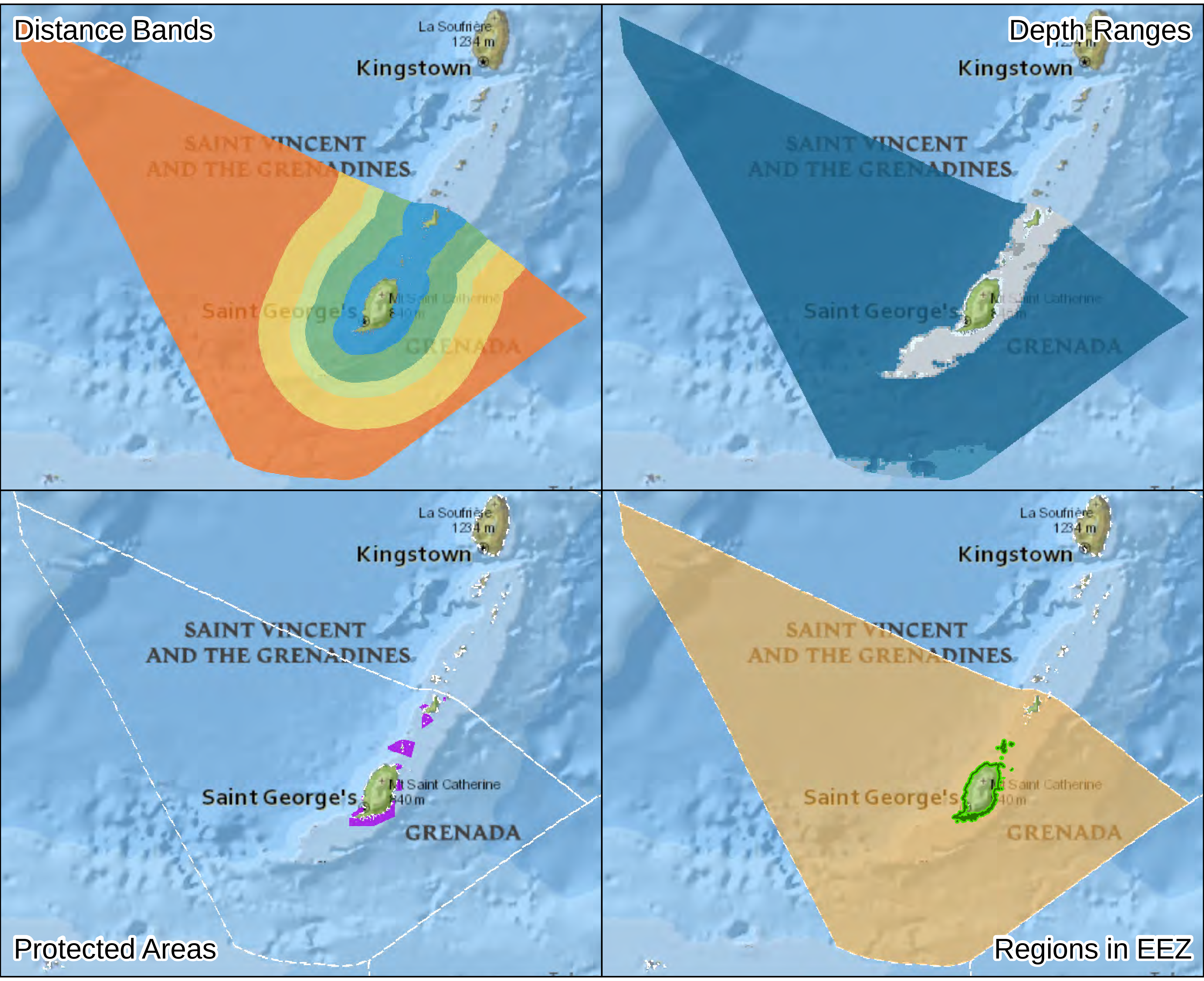

Distance Bands	Depth Bands	Protected	Region
0 to 5 NM	> -10m	Protected Areas	Caribbean
5 to 12 NM	-10m to -50m		
12 to 16 NM	-50m to -100m		
16 to 24 NM	-100m to -150m		
24 to 200 NM	-150m to -200m		
	< -200m		

Coastline Length

190 Km

All Areas in Grenada EEZ: Cell Values = Area in Km^2 [% Depth Band (Row), % Distance Band (Column), % EEZ]

	0 to 5 NM	5 to 12 NM	12 to 16 NM	16 to 24 NM	24 to 200 NM	> 200 NM	Total
> -10m	140 [81%, 8%, 1%]	18 [11%, 1%, 0%]	9 [5%, 1%, 0%]	7 [4%, 0%, 0%]	0 [0%, 0%, 0%]	0 [0%, 0%, 0%]	173 [1% of Total]
-10m to -50m	897 [72%, 49%, 3%]	243 [19%, 9%, 1%]	88 [7%, 5%, 0%]	23 [2%, 1%, 0%]	0 [0%, 0%, 0%]	0 [0%, 0%, 0%]	1,252 [5% of Total]
-50m to -100m	117 [58%, 6%, 0%]	56 [28%, 2%, 0%]	15 [8%, 1%, 0%]	10 [5%, 0%, 0%]	3 [1%, 0%, 0%]	0 [0%, 0%, 0%]	203 [1% of Total]
-100m to -150m	53 [23%, 3%, 0%]	32 [14%, 1%, 0%]	10 [4%, 1%, 0%]	15 [6%, 0%, 0%]	125 [53%, 1%, 0%]	0 [0%, 0%, 0%]	236 [1% of Total]
-150m to -200m	26 [5%, 1%, 0%]	22 [4%, 1%, 0%]	4 [1%, 0%, 0%]	6 [1%, 0%, 0%]	438 [88%, 3%, 2%]	0 [0%, 0%, 0%]	496 [2% of Total]
<-200m	602 [3%, 33%, 2%]	2,246 [10%, 86%, 9%]	1,567 [7%, 93%, 6%]	3,790 [16%, 98%, 15%]	15,107 [65%, 96%, 59%]	0 [0%, 0%, 0%]	23,312 [91% of Total]
Total	1,836 [7% of Total]	2,618 [10% of Total]	1,693 [7% of Total]	3,851 [15% of Total]	15,672 [61% of Total]	0.000 [0% of Total]	25,671 Km^2

Areas in Grenada EEZ Excluding Protected Areas: Cell Values = Area in Km^2 [% Depth Band (Row), % Distance Band (Column), % EEZ]

216 [1%] Km^2 Protected	0 to 5 NM	5 to 12 NM	12 to 16 NM	16 to 24 NM	24 to 200 NM	> 200 NM	Total
> -10m	71 [68%, 4%, 0%]	18 [17%, 1%, 0%]	9 [8%, 1%, 0%]	7 [6%, 0%, 0%]	0 [0%, 0%, 0%]	0 [0%, 0%, 0%]	105 [0% of Total]
-10m to -50m	770 [68%, 48%, 3%]	243 [22%, 9%, 1%]	88 [8%, 5%, 0%]	23 [2%, 1%, 0%]	0 [0%, 0%, 0%]	0 [0%, 0%, 0%]	1,125 [4% of Total]
-50m to -100m	112 [57%, 7%, 0%]	56 [29%, 2%, 0%]	15 [8%, 1%, 0%]	10 [5%, 0%, 0%]	3 [1%, 0%, 0%]	0 [0%, 0%, 0%]	197 [1% of Total]
-100m to -150m	49 [21%, 3%, 0%]	32 [14%, 1%, 0%]	10 [4%, 1%, 0%]	15 [7%, 0%, 0%]	125 [54%, 1%, 0%]	0 [0%, 0%, 0%]	231 [1% of Total]
-150m to -200m	24 [5%, 2%, 0%]	22 [4%, 1%, 0%]	4 [1%, 0%, 0%]	6 [1%, 0%, 0%]	438 [89%, 3%, 2%]	0 [0%, 0%, 0%]	494 [2% of Total]
<-200m	593 [3%, 37%, 2%]	2,246 [10%, 86%, 9%]	1,567 [7%, 93%, 6%]	3,790 [16%, 98%, 15%]	15,107 [65%, 96%, 59%]	0 [0%, 0%, 0%]	23,303 [92% of Total]
Total	1,620 [6% of Total]	2,618 [10% of Total]	1,693 [7% of Total]	3,851 [15% of Total]	15,672 [62% of Total]	0.000 [0% of Total]	25,455 Km^2

The designations employed and the presentation of material in the map do not imply the expression of any opinion whatsoever on the part of FAO concerning the legal or constitutional status of any country, territory or sea area, or concerning the delimitation of frontiers.

Background reference map from National Geographic. Content may not reflect National Geographic's current map policy. Sources: National Geographic, Esri, DeLorme, HERE, UNEP-WCMC, USGS, NASA, ESA, METI, NRCAN, GEBCO, NOAA, increment P Corp.

Projection: Azimuthal Equidistant
Datum: WGS 1984
False Easting: 0.0000
False Northing: 0.0000
Central Meridian: -62.0241
Latitude Of Origin: 12.3592

N W E S

Food and Agriculture Organization of the United Nations

Guatemala: Caribbean Region

©FAO, 2018

Kilometers
0 50

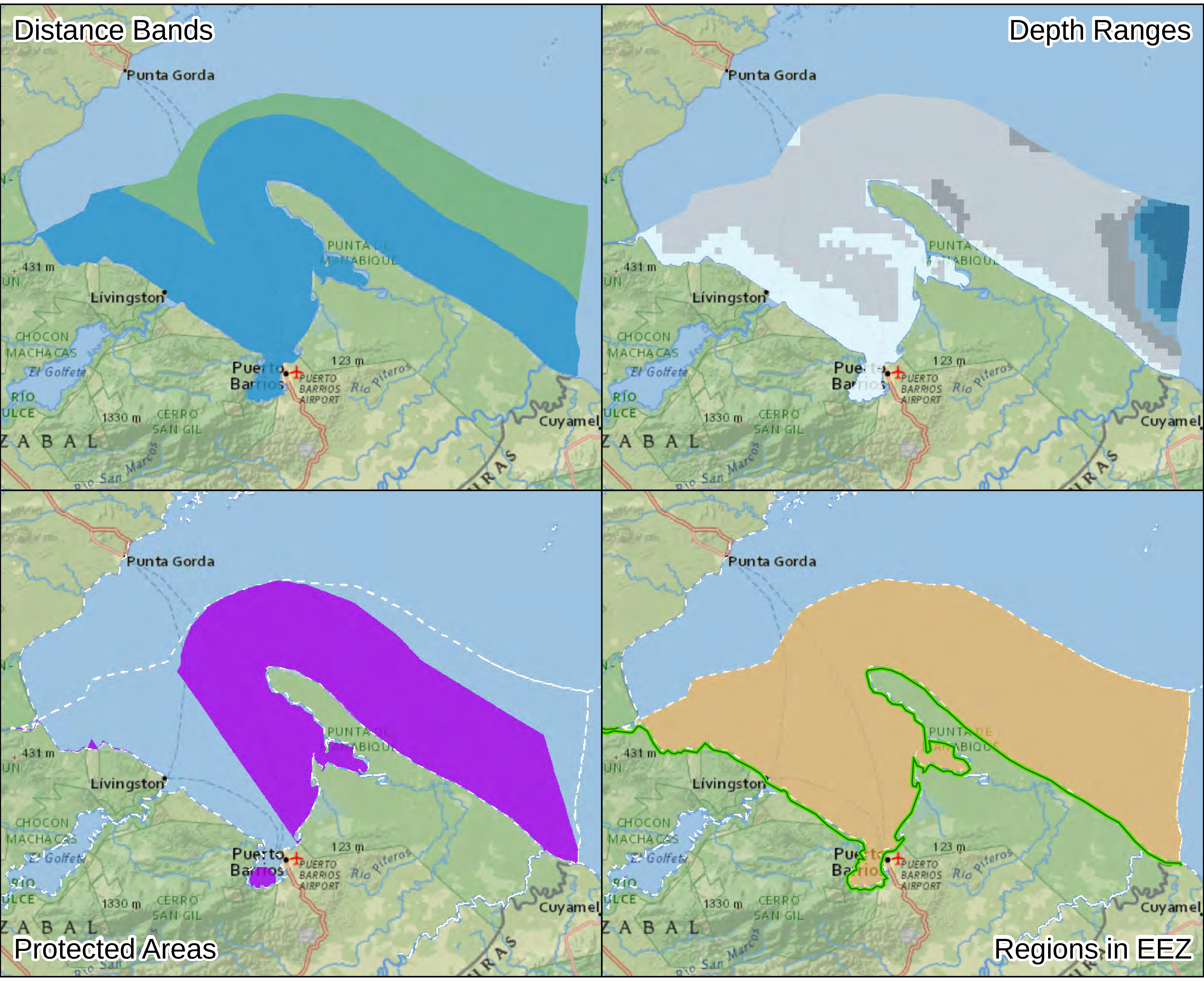

Distance Bands
- 0 to 5 NM
- 5 to 12 NM

Depth Bands
- > -10m
- -10m to -50m
- -50m to -100m
- -100m to -150m
- -150m to -200m
- < -200m

Protected
- Protected Areas

Region
- Caribbean

Coastline Length
- 179 Km

All Areas in Guatemala EEZ: Cell Values = Area in Km^2 [% Depth Band (Row), % Distance Band (Column), % EEZ]

	0 to 5 NM	5 to 12 NM	12 to 16 NM	16 to 24 NM	24 to 200 NM	> 200 NM	Total
> -10m	297 [99%, 28%, 20%]	3 [1%, 1%, 0%]	0 [0%, 0%, 0%]	0 [0%, 0%, 0%]	0 [0%, 0%, 0%]	0 [0%, 0%, 0%]	300 [20% of Total]
-10m to -50m	676 [69%, 63%, 45%]	304 [31%, 72%, 20%]	0 [0%, 0%, 0%]	0 [0%, 0%, 0%]	0 [0%, 0%, 0%]	0 [0%, 0%, 0%]	979 [66% of Total]
-50m to -100m	56 [64%, 5%, 4%]	31 [36%, 7%, 2%]	0 [0%, 0%, 0%]	0 [0%, 0%, 0%]	0 [0%, 0%, 0%]	0 [0%, 0%, 0%]	88 [6% of Total]
-100m to -150m	18 [59%, 2%, 1%]	13 [41%, 3%, 1%]	0 [0%, 0%, 0%]	0 [0%, 0%, 0%]	0 [0%, 0%, 0%]	0 [0%, 0%, 0%]	31 [2% of Total]
-150m to -200m	12 [38%, 1%, 1%]	20 [62%, 5%, 1%]	0 [0%, 0%, 0%]	0 [0%, 0%, 0%]	0 [0%, 0%, 0%]	0 [0%, 0%, 0%]	32 [2% of Total]
<-200m	6 [10%, 1%, 0%]	51 [90%, 12%, 3%]	0 [0%, 0%, 0%]	0 [0%, 0%, 0%]	0 [0%, 0%, 0%]	0 [0%, 0%, 0%]	56 [4% of Total]
Total	1,066 [72% of Total]	421 [28% of Total]	0.000 [0% of Total]	0.000 [0% of Total]	0.000 [0% of Total]	0.000 [0% of Total]	1,487 Km^2

Areas in Guatemala EEZ Excluding Protected Areas: Cell Values = Area in Km^2 [% Depth Band (Row), % Distance Band (Column), % EEZ]

923 [62%] Km^2 Protected	0 to 5 NM	5 to 12 NM	12 to 16 NM	16 to 24 NM	24 to 200 NM	> 200 NM	Total
> -10m	160 [99%, 50%, 28%]	2 [1%, 1%, 0%]	0 [0%, 0%, 0%]	0 [0%, 0%, 0%]	0 [0%, 0%, 0%]	0 [0%, 0%, 0%]	162 [29% of Total]
-10m to -50m	143 [48%, 45%, 25%]	154 [52%, 62%, 27%]	0 [0%, 0%, 0%]	0 [0%, 0%, 0%]	0 [0%, 0%, 0%]	0 [0%, 0%, 0%]	297 [53% of Total]
-50m to -100m	2 [8%, 1%, 0%]	18 [92%, 7%, 3%]	0 [0%, 0%, 0%]	0 [0%, 0%, 0%]	0 [0%, 0%, 0%]	0 [0%, 0%, 0%]	19 [3% of Total]
-100m to -150m	2 [33%, 1%, 0%]	5 [67%, 2%, 1%]	0 [0%, 0%, 0%]	0 [0%, 0%, 0%]	0 [0%, 0%, 0%]	0 [0%, 0%, 0%]	7 [1% of Total]
-150m to -200m	4 [19%, 1%, 1%]	18 [81%, 7%, 3%]	0 [0%, 0%, 0%]	0 [0%, 0%, 0%]	0 [0%, 0%, 0%]	0 [0%, 0%, 0%]	23 [4% of Total]
<-200m	5 [10%, 2%, 1%]	51 [90%, 21%, 9%]	0 [0%, 0%, 0%]	0 [0%, 0%, 0%]	0 [0%, 0%, 0%]	0 [0%, 0%, 0%]	56 [10% of Total]
Total	317 [56% of Total]	247 [44% of Total]	0.000 [0% of Total]	0.000 [0% of Total]	0.000 [0% of Total]	0.000 [0% of Total]	564 Km^2

The designations employed and the presentation of material in the map do not imply the expression of any opinion whatsoever on the part of FAO concerning the legal or constitutional status of any country, territory or sea area, or concerning the delimitation of frontiers.

Background reference map from National Geographic. Content may not reflect National Geographic's current map policy. Sources: National Geographic, Esri, DeLorme, HERE, UNEP-WCMC, USGS, NASA, ESA, METI, NRCAN, GEBCO, NOAA, increment P Corp.

Projection: Azimuthal Equidistant
Datum: WGS 1984
False Easting: 0.0000
False Northing: 0.0000
Central Meridian: -88.5775
Latitude Of Origin: 15.8828

Food and Agriculture Organization of the United Nations

Guyana: Caribbean Region

©FAO, 2018

Kilometers
0 125 250 375

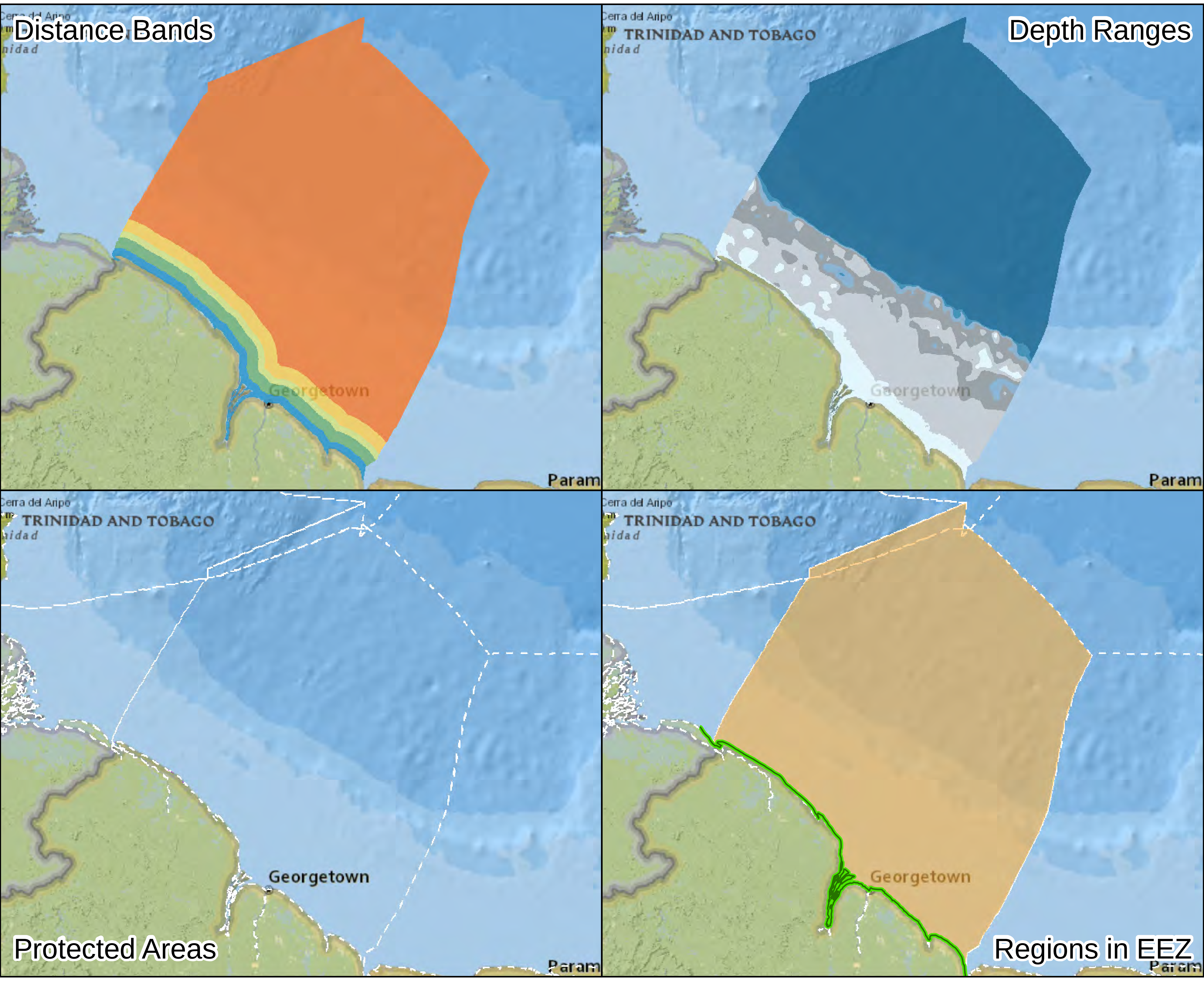

Distance Bands
- 0 to 5 NM
- 5 to 12 NM
- 12 to 16 NM
- 16 to 24 NM
- 24 to 200 NM

Depth Bands
- > -10m
- -10m to -50m
- -50m to -100m
- -100m to -150m
- -150m to -200m
- < -200m

Protected
- Protected Areas

Coastline Length
- 913 Km

Region
- Caribbean

All Areas in Guyana EEZ: Cell Values = Area in Km^2 [% Depth Band (Row), % Distance Band (Column), % EEZ]

	0 to 5 NM	5 to 12 NM	12 to 16 NM	16 to 24 NM	24 to 200 NM	> 200 NM	Total
> -10m	3,415 [44%, 79%, 2%]	3,015 [39%, 55%, 2%]	506 [7%, 16%, 0%]	292 [4%, 5%, 0%]	538 [7%, 0%, 0%]	0 [0%, 0%, 0%]	7,765 [6% of Total]
-10m to -50m	886 [4%, 20%, 1%]	2,467 [10%, 45%, 2%]	2,637 [10%, 84%, 2%]	5,638 [22%, 90%, 4%]	13,575 [54%, 11%, 10%]	0 [0%, 0%, 0%]	25,203 [18% of Total]
-50m to -100m	24 [0%, 1%, 0%]	0 [0%, 0%, 0%]	0 [0%, 0%, 0%]	324 [3%, 5%, 0%]	12,145 [97%, 10%, 9%]	0 [0%, 0%, 0%]	12,493 [9% of Total]
-100m to -150m	0 [0%, 0%, 0%]	0 [0%, 0%, 0%]	0 [0%, 0%, 0%]	0 [0%, 0%, 0%]	2,563 [100%, 2%, 2%]	0 [0%, 0%, 0%]	2,563 [2% of Total]
-150m to -200m	0 [0%, 0%, 0%]	0 [0%, 0%, 0%]	0 [0%, 0%, 0%]	0 [0%, 0%, 0%]	1,054 [100%, 1%, 1%]	0 [0%, 0%, 0%]	1,054 [1% of Total]
<-200m	0 [0%, 0%, 0%]	0 [0%, 0%, 0%]	0 [0%, 0%, 0%]	0 [0%, 0%, 0%]	89,643 [100%, 75%, 65%]	0 [0%, 0%, 0%]	89,643 [65% of Total]
Total	4,324 [3% of Total]	5,481 [4% of Total]	3,143 [2% of Total]	6,255 [5% of Total]	119,519 [86% of Total]	0.000 [0% of Total]	138,722 Km^2

Areas in Guyana EEZ Excluding Protected Areas: Cell Values = Area in Km^2 [% Depth Band (Row), % Distance Band (Column), % EEZ]

3 [0%] Km^2 Protected	0 to 5 NM	5 to 12 NM	12 to 16 NM	16 to 24 NM	24 to 200 NM	> 200 NM	Total
> -10m	3,412 [44%, 79%, 2%]	3,015 [39%, 55%, 2%]	506 [7%, 16%, 0%]	292 [4%, 5%, 0%]	538 [7%, 0%, 0%]	0 [0%, 0%, 0%]	7,762 [6% of Total]
-10m to -50m	886 [4%, 20%, 1%]	2,467 [10%, 45%, 2%]	2,637 [10%, 84%, 2%]	5,638 [22%, 90%, 4%]	13,575 [54%, 11%, 10%]	0 [0%, 0%, 0%]	25,203 [18% of Total]
-50m to -100m	24 [0%, 1%, 0%]	0 [0%, 0%, 0%]	0 [0%, 0%, 0%]	324 [3%, 5%, 0%]	12,145 [97%, 10%, 9%]	0 [0%, 0%, 0%]	12,493 [9% of Total]
-100m to -150m	0 [0%, 0%, 0%]	0 [0%, 0%, 0%]	0 [0%, 0%, 0%]	0 [0%, 0%, 0%]	2,563 [100%, 2%, 2%]	0 [0%, 0%, 0%]	2,563 [2% of Total]
-150m to -200m	0 [0%, 0%, 0%]	0 [0%, 0%, 0%]	0 [0%, 0%, 0%]	0 [0%, 0%, 0%]	1,054 [100%, 1%, 1%]	0 [0%, 0%, 0%]	1,054 [1% of Total]
<-200m	0 [0%, 0%, 0%]	0 [0%, 0%, 0%]	0 [0%, 0%, 0%]	0 [0%, 0%, 0%]	89,643 [100%, 75%, 65%]	0 [0%, 0%, 0%]	89,643 [65% of Total]
Total	4,321 [3% of Total]	5,481 [4% of Total]	3,143 [2% of Total]	6,255 [5% of Total]	119,519 [86% of Total]	0.000 [0% of Total]	138,719 Km^2

The designations employed and the presentation of material in the map do not imply the expression of any opinion whatsoever on the part of FAO concerning the legal or constitutional status of any country, territory or sea area, or concerning the delimitation of frontiers.

Background reference map from National Geographic. Content may not reflect National Geographic's current map policy. Sources: National Geographic, Esri, DeLorme, HERE, UNEP-WCMC, USGS, NASA, ESA, METI, NRCAN, GEBCO, NOAA, increment P Corp.

Projection: Azimuthal Equidistant
Datum: WGS 1984
False Easting: 0.0000
False Northing: 0.0000
Central Meridian: -57.8061
Latitude Of Origin: 8.4886

Food and Agriculture Organization of the United Nations

Haiti: Caribbean Region

©FAO, 2018

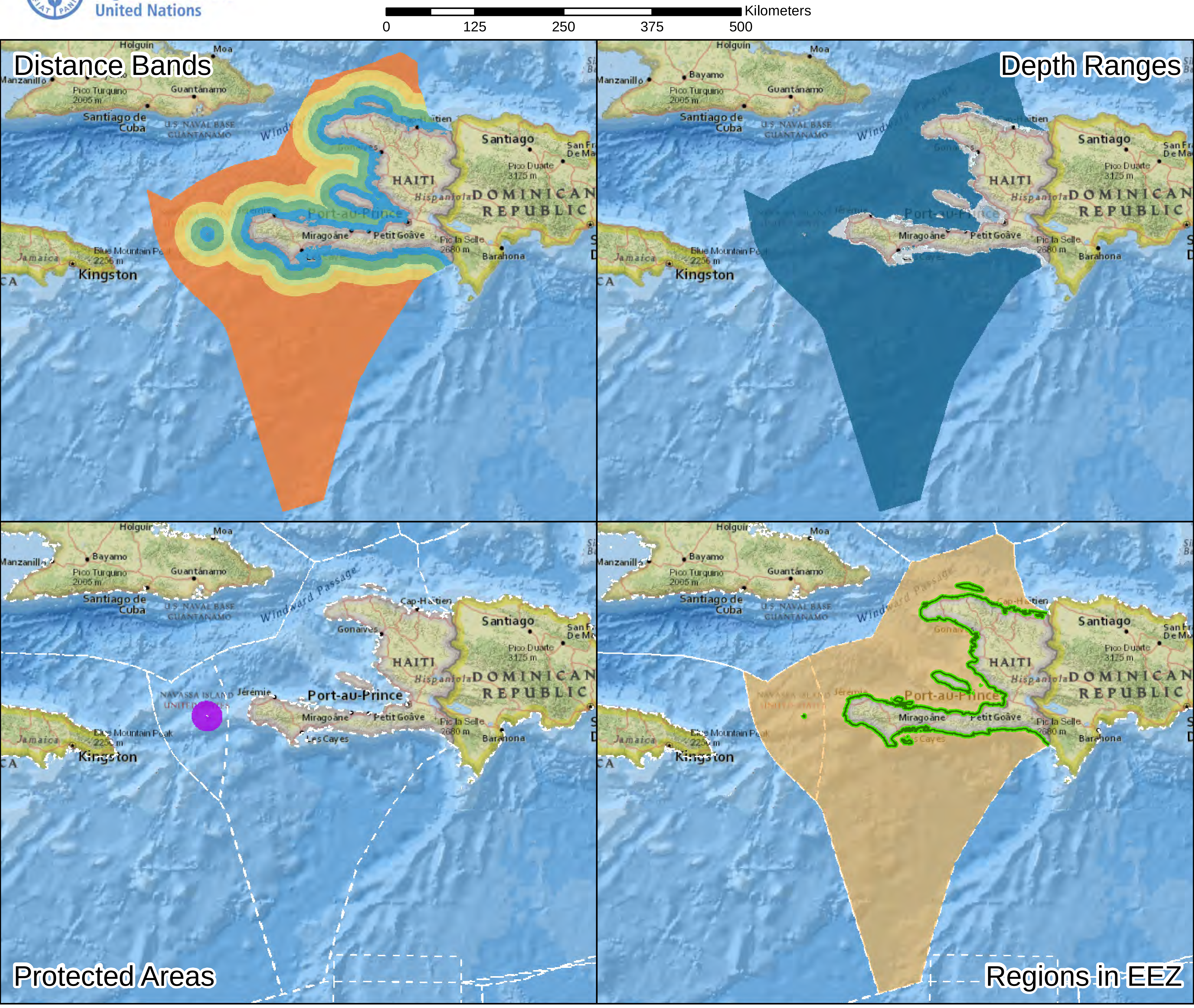

Distance Bands

- 0 to 5 NM
- 5 to 12 NM
- 12 to 16 NM
- 16 to 24 NM
- 24 to 200 NM

Depth Bands

- > -10m
- -10m to -50m
- -50m to -100m
- -100m to -150m
- -150m to -200m
- < -200m

Protected

- Protected Areas

Coastline Length

2,135 Km

Region

- Caribbean

All Areas in Haiti EEZ: Cell Values = Area in Km^2 [% Depth Band (Row), % Distance Band (Column), % EEZ]

	0 to 5 NM	5 to 12 NM	12 to 16 NM	16 to 24 NM	24 to 200 NM	> 200 NM	Total
> -10m	1,340 [99%, 10%, 1%]	17 [1%, 0%, 0%]	0 [0%, 0%, 0%]	0 [0%, 0%, 0%]	0.037 [0%, 0%, 0%]	0 [0%, 0%, 0%]	1,357 [1% of Total]
-10m to -50m	2,103 [91%, 16%, 2%]	211 [9%, 1%, 0%]	0 [0%, 0%, 0%]	0 [0%, 0%, 0%]	3 [0%, 0%, 0%]	0 [0%, 0%, 0%]	2,317 [2% of Total]
-50m to -100m	678 [93%, 5%, 1%]	52 [7%, 0%, 0%]	0.082 [0%, 0%, 0%]	0 [0%, 0%, 0%]	0.814 [0%, 0%, 0%]	0 [0%, 0%, 0%]	731 [1% of Total]
-100m to -150m	684 [88%, 5%, 1%]	84 [11%, 1%, 0%]	0.059 [0%, 0%, 0%]	0 [0%, 0%, 0%]	7 [1%, 0%, 0%]	0 [0%, 0%, 0%]	774 [1% of Total]
-150m to -200m	432 [95%, 3%, 0%]	16 [4%, 0%, 0%]	0.034 [0%, 0%, 0%]	0 [0%, 0%, 0%]	7 [2%, 0%, 0%]	0 [0%, 0%, 0%]	455 [0% of Total]
<-200m	7,968 [7%, 60%, 7%]	14,431 [13%, 97%, 12%]	8,036 [7%, 100%, 7%]	14,862 [13%, 100%, 13%]	66,796 [60%, 100%, 57%]	0 [0%, 0%, 0%]	112,093 [95% of Total]
Total	13,205 [11% of Total]	14,811 [13% of Total]	8,036 [7% of Total]	14,862 [13% of Total]	66,813 [57% of Total]	0.000 [0% of Total]	117,728 Km^2

Areas in Haiti EEZ Excluding Protected Areas: Cell Values = Area in Km^2 [% Depth Band (Row), % Distance Band (Column), % EEZ]

1,473 [1%] Km^2 Protected	0 to 5 NM	5 to 12 NM	12 to 16 NM	16 to 24 NM	24 to 200 NM	> 200 NM	Total
> -10m	1,337 [99%, 10%, 1%]	17 [1%, 0%, 0%]	0 [0%, 0%, 0%]	0 [0%, 0%, 0%]	0.037 [0%, 0%, 0%]	0 [0%, 0%, 0%]	1,354 [1% of Total]
-10m to -50m	2,101 [91%, 16%, 2%]	211 [9%, 2%, 0%]	0 [0%, 0%, 0%]	0 [0%, 0%, 0%]	3 [0%, 0%, 0%]	0 [0%, 0%, 0%]	2,315 [2% of Total]
-50m to -100m	673 [93%, 5%, 1%]	52 [7%, 0%, 0%]	0.082 [0%, 0%, 0%]	0 [0%, 0%, 0%]	0.814 [0%, 0%, 0%]	0 [0%, 0%, 0%]	726 [1% of Total]
-100m to -150m	681 [88%, 5%, 1%]	84 [11%, 1%, 0%]	0.059 [0%, 0%, 0%]	0 [0%, 0%, 0%]	7 [1%, 0%, 0%]	0 [0%, 0%, 0%]	772 [1% of Total]
-150m to -200m	431 [95%, 3%, 0%]	16 [4%, 0%, 0%]	0.034 [0%, 0%, 0%]	0 [0%, 0%, 0%]	7 [2%, 0%, 0%]	0 [0%, 0%, 0%]	454 [0% of Total]
<-200m	7,630 [7%, 59%, 7%]	13,310 [12%, 97%, 11%]	8,036 [7%, 100%, 7%]	14,862 [13%, 100%, 13%]	66,796 [60%, 100%, 57%]	0 [0%, 0%, 0%]	110,635 [95% of Total]
Total	12,853 [11% of Total]	13,691 [12% of Total]	8,036 [7% of Total]	14,862 [13% of Total]	66,813 [57% of Total]	0.000 [0% of Total]	116,255 Km^2

The designations employed and the presentation of material in the map do not imply the expression of any opinion whatsoever on the part of FAO concerning the legal or constitutional status of any country, territory or sea area, or concerning the delimitation of frontiers.

Background reference map from National Geographic. Content may not reflect National Geographic's current map policy. Sources: National Geographic, Esri, DeLorme, HERE, UNEP-WCMC, USGS, NASA, ESA, METI, NRCAN, GEBCO, NOAA, increment P Corp.

Projection: Azimuthal Equidistant
Datum: WGS 1984
False Easting: 0.0000
False Northing: 0.0000
Central Meridian: -73.7955
Latitude Of Origin: 17.8003

Food and Agriculture Organization of the United Nations

Honduras: Caribbean Region

©FAO, 2018

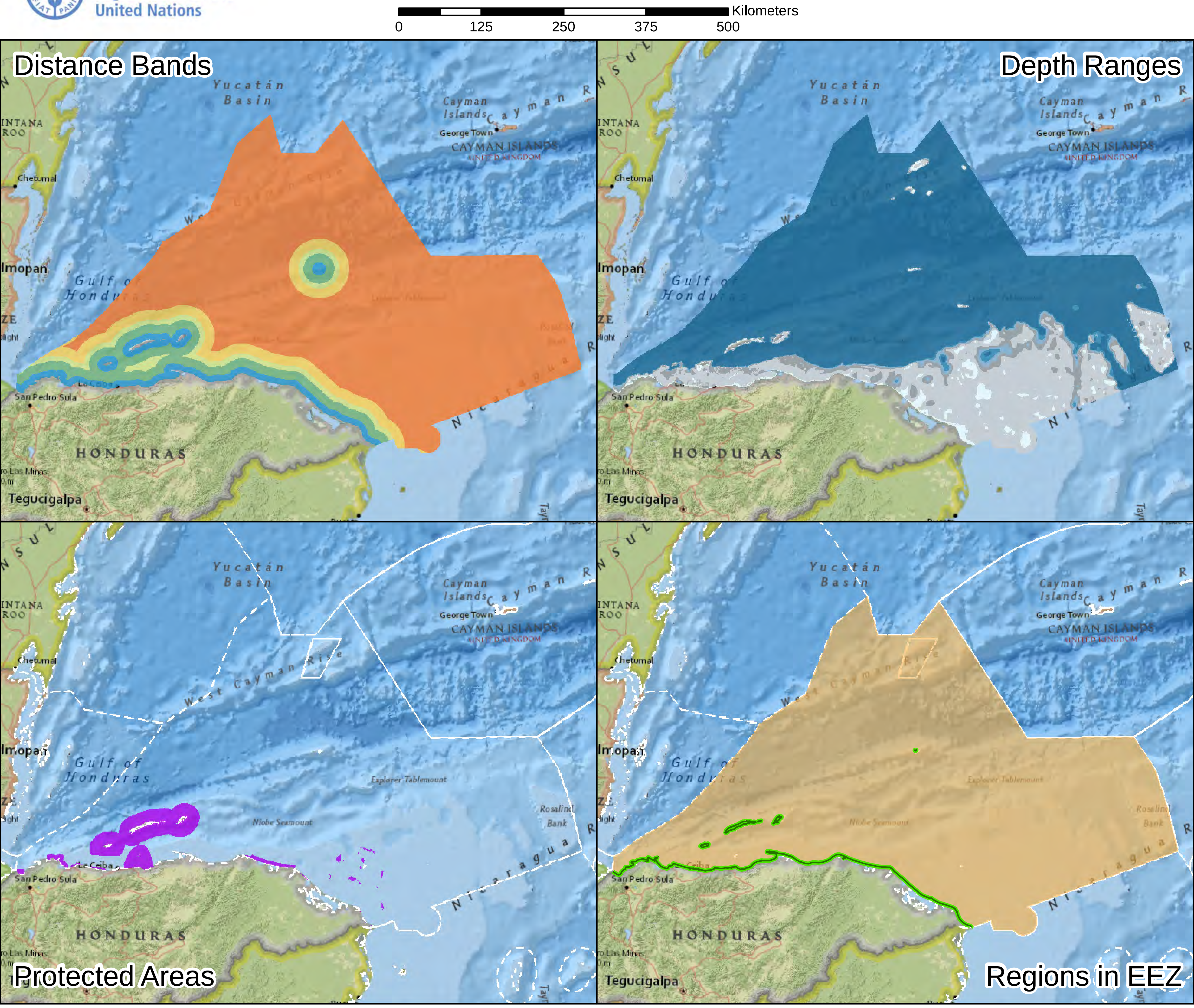

Distance Bands		Depth Bands		Protected	Region
0 to 5 NM	16 to 24 NM	> -10m	-100m to -150m	Protected Areas	Caribbean
5 to 12 NM	24 to 200 NM	-10m to -50m	-150m to -200m		
12 to 16 NM		-50m to -100m	< -200m		

Coastline Length: 932 Km

All Areas in Honduras EEZ: Cell Values = Area in Km^2 [% Depth Band (Row), % Distance Band (Column), % EEZ]

	0 to 5 NM	5 to 12 NM	12 to 16 NM	16 to 24 NM	24 to 200 NM	> 200 NM	Total
> -10m	1,507 [30%, 17%, 1%]	360 [7%, 2%, 0%]	37 [1%, 1%, 0%]	103 [2%, 1%, 0%]	2,941 [59%, 2%, 1%]	0 [0%, 0%, 0%]	4,947 [2% of Total]
-10m to -50m	3,895 [10%, 44%, 2%]	4,299 [11%, 30%, 2%]	1,554 [4%, 21%, 1%]	2,733 [7%, 20%, 1%]	26,991 [68%, 16%, 13%]	0 [0%, 0%, 0%]	39,472 [19% of Total]
-50m to -100m	901 [10%, 10%, 0%]	1,913 [21%, 13%, 1%]	618 [7%, 9%, 0%]	99 [1%, 1%, 0%]	5,547 [61%, 3%, 3%]	0 [0%, 0%, 0%]	9,078 [4% of Total]
-100m to -150m	302 [9%, 3%, 0%]	225 [6%, 2%, 0%]	160 [5%, 2%, 0%]	41 [1%, 0%, 0%]	2,733 [79%, 2%, 1%]	0 [0%, 0%, 0%]	3,462 [2% of Total]
-150m to -200m	192 [7%, 2%, 0%]	153 [5%, 1%, 0%]	127 [4%, 2%, 0%]	42 [1%, 0%, 0%]	2,354 [82%, 1%, 1%]	0 [0%, 0%, 0%]	2,869 [1% of Total]
<-200m	1,992 [1%, 23%, 1%]	7,453 [5%, 52%, 4%]	4,736 [3%, 65%, 2%]	10,859 [7%, 78%, 5%]	124,579 [83%, 75%, 59%]	0 [0%, 0%, 0%]	149,619 [71% of Total]
Total	8,789 [4% of Total]	14,403 [7% of Total]	7,232 [3% of Total]	13,877 [7% of Total]	165,145 [79% of Total]	0.000 [0% of Total]	209,446 Km^2

Areas in Honduras EEZ Excluding Protected Areas: Cell Values = Area in Km^2 [% Depth Band (Row), % Distance Band (Column), % EEZ]

8,711 [4%] Km^2 Protected	0 to 5 NM	5 to 12 NM	12 to 16 NM	16 to 24 NM	24 to 200 NM	> 200 NM	Total
> -10m	1,020 [23%, 20%, 1%]	334 [8%, 3%, 0%]	36 [1%, 1%, 0%]	103 [2%, 1%, 0%]	2,893 [66%, 2%, 1%]	0 [0%, 0%, 0%]	4,385 [2% of Total]
-10m to -50m	2,829 [8%, 56%, 1%]	3,684 [10%, 37%, 2%]	1,483 [4%, 21%, 1%]	2,643 [7%, 19%, 1%]	26,857 [72%, 16%, 13%]	0 [0%, 0%, 0%]	37,497 [19% of Total]
-50m to -100m	567 [7%, 11%, 0%]	1,718 [20%, 17%, 1%]	508 [6%, 7%, 0%]	99 [1%, 1%, 0%]	5,547 [66%, 3%, 3%]	0 [0%, 0%, 0%]	8,439 [4% of Total]
-100m to -150m	144 [4%, 3%, 0%]	193 [6%, 2%, 0%]	149 [5%, 2%, 0%]	41 [1%, 0%, 0%]	2,733 [84%, 2%, 1%]	0 [0%, 0%, 0%]	3,260 [2% of Total]
-150m to -200m	68 [2%, 1%, 0%]	128 [5%, 1%, 0%]	116 [4%, 2%, 0%]	41 [2%, 0%, 0%]	2,354 [87%, 1%, 1%]	0 [0%, 0%, 0%]	2,707 [1% of Total]
<-200m	399 [0%, 8%, 0%]	3,961 [3%, 40%, 2%]	4,651 [3%, 67%, 2%]	10,857 [8%, 79%, 5%]	124,579 [86%, 76%, 62%]	0 [0%, 0%, 0%]	144,447 [72% of Total]
Total	5,026 [3% of Total]	10,019 [5% of Total]	6,943 [3% of Total]	13,785 [7% of Total]	164,963 [82% of Total]	0.000 [0% of Total]	200,735 Km^2

The designations employed and the presentation of material in the map do not imply the expression of any opinion whatsoever on the part of FAO concerning the legal or constitutional status of any country, territory or sea area, or concerning the delimitation of frontiers.

Background reference map from National Geographic. Content may not reflect National Geographic's current map policy. Sources: National Geographic, Esri, DeLorme, HERE, UNEP-WCMC, USGS, NASA, ESA, METI, NRCAN, GEBCO, NOAA, increment P Corp.

Projection: Azimuthal Equidistant
Datum: WGS 1984
False Easting: 0.0000
False Northing: 0.0000
Central Meridian: -84.2294
Latitude Of Origin: 17.2094

©FAO, 2018

Food and Agriculture Organization of the United Nations

Ireland: Atlantic Region

0 125 250 375 500 625 750 Kilometers

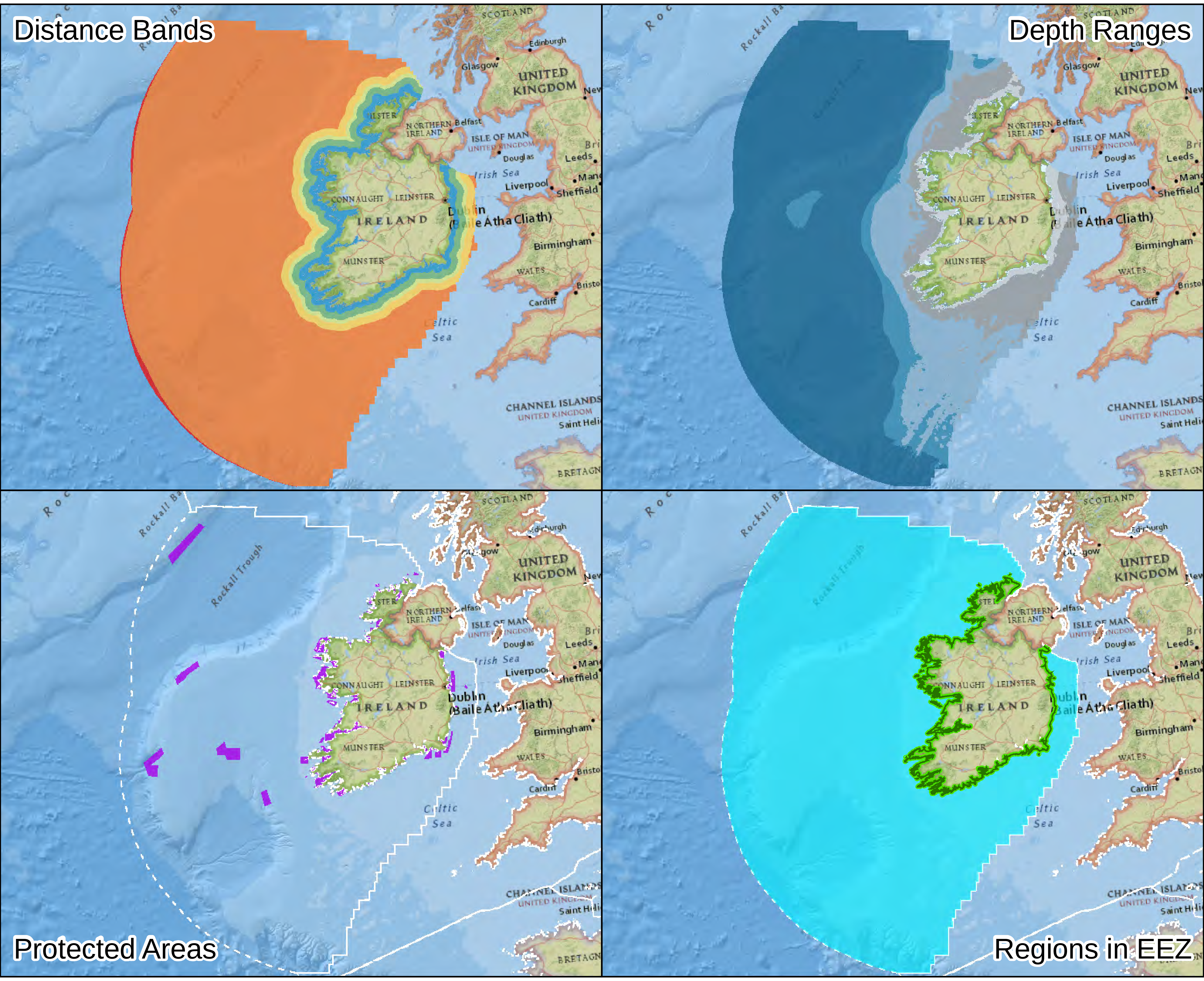

Distance Bands		Depth Bands		Protected	Region
0 to 5 NM	16 to 24 NM	> -10m	-100m to -150m	Protected Areas	Atlantic
5 to 12 NM	24 to 200 NM	-10m to -50m	-150m to -200m	**Coastline Length**	
12 to 16 NM	> 200 NM	-50m to -100m	< -200m	5,520 Km	

All Areas in Ireland EEZ: Cell Values = Area in Km² [% Depth Band (Row), % Distance Band (Column), % EEZ]

	0 to 5 NM	5 to 12 NM	12 to 16 NM	16 to 24 NM	24 to 200 NM	> 200 NM	Total
> -10m	2,586 [100%, 14%, 1%]	11 [0%, 0%, 0%]	0 [0%, 0%, 0%]	0 [0%, 0%, 0%]	0 [0%, 0%, 0%]	0 [0%, 0%, 0%]	2,597 [1% of Total]
-10m to -50m	9,998 [80%, 53%, 2%]	2,282 [18%, 13%, 1%]	278 [2%, 3%, 0%]	17 [0%, 0%, 0%]	0 [0%, 0%, 0%]	0 [0%, 0%, 0%]	12,575 [3% of Total]
-50m to -100m	5,905 [13%, 32%, 1%]	11,890 [27%, 69%, 3%]	6,099 [14%, 63%, 1%]	9,875 [22%, 52%, 2%]	10,745 [24%, 3%, 3%]	0 [0%, 0%, 0%]	44,514 [10% of Total]
-100m to -150m	203 [0%, 1%, 0%]	3,100 [5%, 18%, 1%]	3,102 [5%, 32%, 1%]	6,679 [11%, 35%, 2%]	48,454 [79%, 14%, 11%]	0 [0%, 0%, 0%]	61,538 [14% of Total]
-150m to -200m	0 [0%, 0%, 0%]	2 [0%, 0%, 0%]	182 [1%, 2%, 0%]	2,028 [8%, 11%, 0%]	24,521 [92%, 7%, 6%]	0 [0%, 0%, 0%]	26,733 [6% of Total]
<-200m	0 [0%, 0%, 0%]	0 [0%, 0%, 0%]	0 [0%, 0%, 0%]	355 [0%, 2%, 0%]	272,919 [99%, 77%, 64%]	3,577 [1%, 100%, 1%]	276,851 [65% of Total]
Total	18,692 [4% of Total]	17,285 [4% of Total]	9,660 [2% of Total]	18,954 [4% of Total]	356,640 [84% of Total]	3,577 [1% of Total]	424,808 Km²

Areas in Ireland EEZ Excluding Protected Areas: Cell Values = Area in Km² [% Depth Band (Row), % Distance Band (Column), % EEZ]

9,630 [2%] Km² Protected	0 to 5 NM	5 to 12 NM	12 to 16 NM	16 to 24 NM	24 to 200 NM	> 200 NM	Total
> -10m	1,039 [99%, 7%, 0%]	10 [1%, 0%, 0%]	0 [0%, 0%, 0%]	0 [0%, 0%, 0%]	0 [0%, 0%, 0%]	0 [0%, 0%, 0%]	1,048 [0% of Total]
-10m to -50m	7,302 [75%, 52%, 2%]	2,163 [22%, 13%, 1%]	278 [3%, 3%, 0%]	17 [0%, 0%, 0%]	0 [0%, 0%, 0%]	0 [0%, 0%, 0%]	9,759 [2% of Total]
-50m to -100m	5,482 [12%, 39%, 1%]	11,882 [27%, 69%, 3%]	6,075 [14%, 63%, 1%]	9,869 [22%, 52%, 2%]	10,745 [24%, 3%, 3%]	0 [0%, 0%, 0%]	44,053 [11% of Total]
-100m to -150m	187 [0%, 1%, 0%]	3,100 [5%, 18%, 1%]	3,102 [5%, 32%, 1%]	6,679 [11%, 35%, 2%]	48,454 [79%, 14%, 12%]	0 [0%, 0%, 0%]	61,523 [15% of Total]
-150m to -200m	0 [0%, 0%, 0%]	2 [0%, 0%, 0%]	182 [1%, 2%, 0%]	2,028 [8%, 11%, 0%]	24,521 [92%, 7%, 6%]	0 [0%, 0%, 0%]	26,733 [6% of Total]
<-200m	0 [0%, 0%, 0%]	0 [0%, 0%, 0%]	0 [0%, 0%, 0%]	355 [0%, 2%, 0%]	268,128 [99%, 76%, 65%]	3,577 [1%, 100%, 1%]	272,061 [66% of Total]
Total	14,010 [3% of Total]	17,157 [4% of Total]	9,637 [2% of Total]	18,948 [5% of Total]	351,849 [85% of Total]	3,577 [1% of Total]	415,178 Km²

The designations employed and the presentation of material in the map do not imply the expression of any opinion whatsoever on the part of FAO concerning the legal or constitutional status of any country, territory or sea area, or concerning the delimitation of frontiers.

Background reference map from National Geographic. Content may not reflect National Geographic's current map policy. Sources: National Geographic, Esri, DeLorme, HERE, UNEP-WCMC, USGS, NASA, ESA, METI, NRCAN, GEBCO, NOAA, increment P Corp.

Projection: Azimuthal Equidistant
Datum: WGS 1984
False Easting: 0.0000
False Northing: 0.0000
Central Meridian: -10.6731
Latitude Of Origin: 52.4394

N W E S

Food and Agriculture Organization of the United Nations

Israel: Mediterranean Region

©FAO, 2018

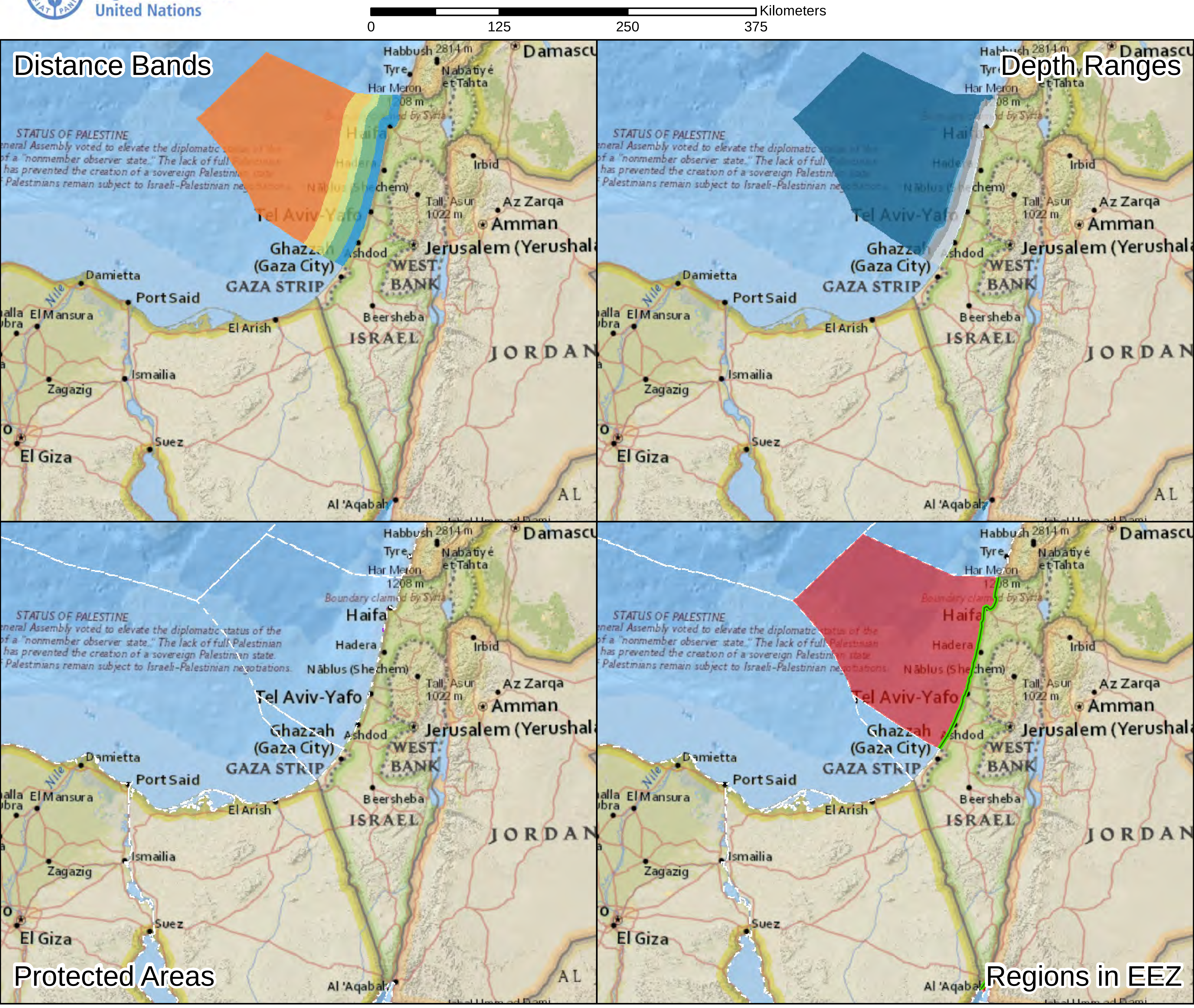

Distance Bands	Depth Bands	Protected	Region
0 to 5 NM	> -10m	Protected Areas	Mediterranean
5 to 12 NM	-10m to -50m		
12 to 16 NM	-50m to -100m		
16 to 24 NM	-100m to -150m		
24 to 200 NM	-150m to -200m		
	< -200m		

Coastline Length

199 Km

All Areas in Israel EEZ: Cell Values = Area in Km^2 [% Depth Band (Row), % Distance Band (Column), % EEZ]

	0 to 5 NM	5 to 12 NM	12 to 16 NM	16 to 24 NM	24 to 200 NM	> 200 NM	Total
> -10m	136 [100%, 8%, 1%]	0 [0%, 0%, 0%]	0 [0%, 0%, 0%]	0 [0%, 0%, 0%]	0 [0%, 0%, 0%]	0 [0%, 0%, 0%]	136 [1% of Total]
-10m to -50m	1,272 [93%, 77%, 5%]	89 [7%, 4%, 0%]	0 [0%, 0%, 0%]	0 [0%, 0%, 0%]	0 [0%, 0%, 0%]	0 [0%, 0%, 0%]	1,361 [6% of Total]
-50m to -100m	201 [22%, 12%, 1%]	694 [78%, 31%, 3%]	0 [0%, 0%, 0%]	0 [0%, 0%, 0%]	0 [0%, 0%, 0%]	0 [0%, 0%, 0%]	895 [4% of Total]
-100m to -150m	8 [2%, 0%, 0%]	343 [98%, 15%, 1%]	0 [0%, 0%, 0%]	0 [0%, 0%, 0%]	0 [0%, 0%, 0%]	0 [0%, 0%, 0%]	350 [1% of Total]
-150m to -200m	3 [1%, 0%, 0%]	227 [92%, 10%, 1%]	17 [7%, 1%, 0%]	0 [0%, 0%, 0%]	0 [0%, 0%, 0%]	0 [0%, 0%, 0%]	247 [1% of Total]
<-200m	34 [0%, 2%, 0%]	893 [4%, 40%, 4%]	1,225 [6%, 99%, 5%]	2,398 [12%, 100%, 10%]	15,982 [78%, 100%, 68%]	0 [0%, 0%, 0%]	20,532 [87% of Total]
Total	1,655 [7% of Total]	2,246 [10% of Total]	1,241 [5% of Total]	2,398 [10% of Total]	15,982 [68% of Total]	0.000 [0% of Total]	23,522 Km^2

Areas in Israel EEZ Excluding Protected Areas: Cell Values = Area in Km^2 [% Depth Band (Row), % Distance Band (Column), % EEZ]

10 [0%] Km^2 Protected	0 to 5 NM	5 to 12 NM	12 to 16 NM	16 to 24 NM	24 to 200 NM	> 200 NM	Total
> -10m	133 [100%, 8%, 1%]	0 [0%, 0%, 0%]	0 [0%, 0%, 0%]	0 [0%, 0%, 0%]	0 [0%, 0%, 0%]	0 [0%, 0%, 0%]	133 [1% of Total]
-10m to -50m	1,266 [93%, 77%, 5%]	89 [7%, 4%, 0%]	0 [0%, 0%, 0%]	0 [0%, 0%, 0%]	0 [0%, 0%, 0%]	0 [0%, 0%, 0%]	1,356 [6% of Total]
-50m to -100m	201 [22%, 12%, 1%]	694 [78%, 31%, 3%]	0 [0%, 0%, 0%]	0 [0%, 0%, 0%]	0 [0%, 0%, 0%]	0 [0%, 0%, 0%]	895 [4% of Total]
-100m to -150m	7 [2%, 0%, 0%]	343 [98%, 15%, 1%]	0 [0%, 0%, 0%]	0 [0%, 0%, 0%]	0 [0%, 0%, 0%]	0 [0%, 0%, 0%]	350 [1% of Total]
-150m to -200m	3 [1%, 0%, 0%]	227 [92%, 10%, 1%]	17 [7%, 1%, 0%]	0 [0%, 0%, 0%]	0 [0%, 0%, 0%]	0 [0%, 0%, 0%]	247 [1% of Total]
<-200m	34 [0%, 2%, 0%]	893 [4%, 40%, 4%]	1,225 [6%, 99%, 5%]	2,398 [12%, 100%, 10%]	15,982 [78%, 100%, 68%]	0 [0%, 0%, 0%]	20,532 [87% of Total]
Total	1,644 [7% of Total]	2,246 [10% of Total]	1,241 [5% of Total]	2,398 [10% of Total]	15,982 [68% of Total]	0.000 [0% of Total]	23,512 Km^2

The designations employed and the presentation of material in the map do not imply the expression of any opinion whatsoever on the part of FAO concerning the legal or constitutional status of any country, territory or sea area, or concerning the delimitation of frontiers.

Background reference map from National Geographic. Content may not reflect National Geographic's current map policy. Sources: National Geographic, Esri, DeLorme, HERE, UNEP-WCMC, USGS, NASA, ESA, METI, NRCAN, GEBCO, NOAA, increment P Corp.

Projection: Azimuthal Equidistant
Datum: WGS 1984
False Easting: 0.0000
False Northing: 0.0000
Central Meridian: 34.0389
Latitude Of Origin: 31.4655

N W E S

Italy: Mediterranean Region

Food and Agriculture Organization of the United Nations

©FAO, 2018

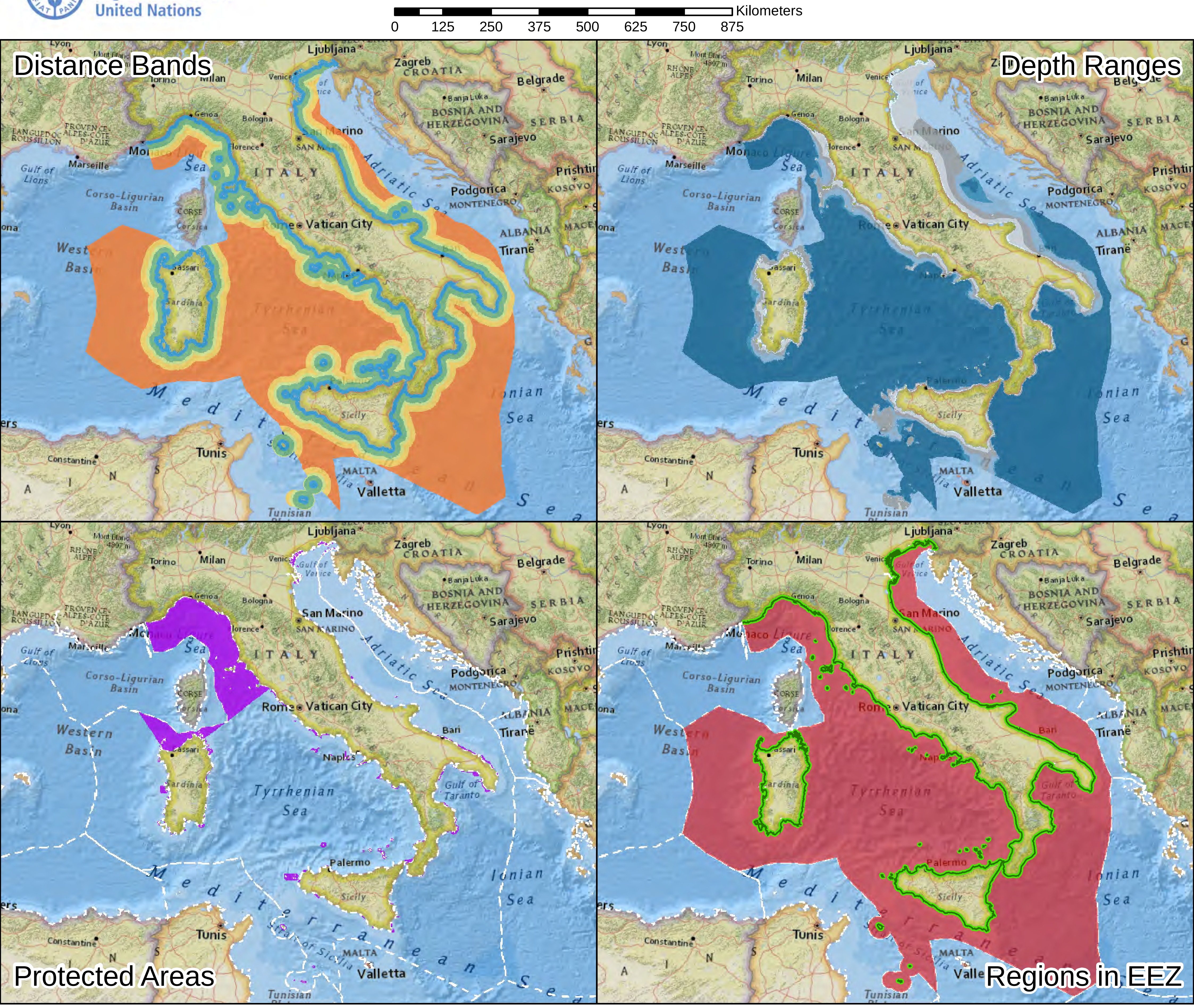

Distance Bands

- 0 to 5 NM
- 5 to 12 NM
- 12 to 16 NM
- 16 to 24 NM
- 24 to 200 NM

Depth Bands

- > -10m
- -10m to -50m
- -50m to -100m
- -100m to -150m
- -150m to -200m
- < -200m

Protected

- Protected Areas

Coastline Length

- 6,954 Km

Region

- Mediterranean

All Areas in Italy EEZ: Cell Values = Area in Km^2 [% Depth Band (Row), % Distance Band (Column), % EEZ]

	0 to 5 NM	5 to 12 NM	12 to 16 NM	16 to 24 NM	24 to 200 NM	> 200 NM	Total
> -10m	5,211 [100%, 9%, 1%]	1 [0%, 0%, 0%]	0 [0%, 0%, 0%]	0 [0%, 0%, 0%]	0.688 [0%, 0%, 0%]	0 [0%, 0%, 0%]	5,213 [1% of Total]
-10m to -50m	18,850 [61%, 33%, 4%]	7,317 [24%, 9%, 1%]	1,660 [5%, 4%, 0%]	2,099 [7%, 3%, 0%]	922 [3%, 0%, 0%]	0 [0%, 0%, 0%]	30,848 [6% of Total]
-50m to -100m	11,972 [35%, 21%, 2%]	9,983 [29%, 13%, 2%]	2,950 [9%, 8%, 1%]	4,868 [14%, 7%, 1%]	4,774 [14%, 2%, 1%]	0 [0%, 0%, 0%]	34,548 [6% of Total]
-100m to -150m	5,437 [18%, 9%, 1%]	12,960 [44%, 16%, 2%]	3,942 [13%, 10%, 1%]	4,434 [15%, 7%, 1%]	2,760 [9%, 1%, 1%]	0 [0%, 0%, 0%]	29,533 [6% of Total]
-150m to -200m	1,969 [16%, 3%, 0%]	4,544 [37%, 6%, 1%]	1,603 [13%, 4%, 0%]	2,177 [18%, 3%, 0%]	2,038 [17%, 1%, 0%]	0 [0%, 0%, 0%]	12,332 [2% of Total]
<-200m	13,861 [3%, 24%, 3%]	44,392 [11%, 56%, 8%]	28,921 [7%, 74%, 5%]	54,567 [13%, 80%, 10%]	280,552 [66%, 96%, 52%]	0 [0%, 0%, 0%]	422,292 [79% of Total]
Total	57,301 [11% of Total]	79,198 [15% of Total]	39,077 [7% of Total]	68,145 [13% of Total]	291,046 [54% of Total]	0.000 [0% of Total]	534,766 Km^2

Areas in Italy EEZ Excluding Protected Areas: Cell Values = Area in Km^2 [% Depth Band (Row), % Distance Band (Column), % EEZ]

47,073 [9%] Km^2 Protected	0 to 5 NM	5 to 12 NM	12 to 16 NM	16 to 24 NM	24 to 200 NM	> 200 NM	Total
> -10m	3,248 [100%, 8%, 1%]	0.347 [0%, 0%, 0%]	0 [0%, 0%, 0%]	0 [0%, 0%, 0%]	0.688 [0%, 0%, 0%]	0 [0%, 0%, 0%]	3,249 [1% of Total]
-10m to -50m	13,977 [55%, 33%, 3%]	6,591 [26%, 10%, 1%]	1,660 [7%, 5%, 0%]	2,099 [8%, 3%, 0%]	922 [4%, 0%, 0%]	0 [0%, 0%, 0%]	25,249 [5% of Total]
-50m to -100m	8,284 [28%, 20%, 2%]	8,425 [29%, 13%, 2%]	2,897 [10%, 8%, 1%]	4,868 [17%, 8%, 1%]	4,774 [16%, 2%, 1%]	0 [0%, 0%, 0%]	29,248 [6% of Total]
-100m to -150m	3,715 [15%, 9%, 1%]	10,361 [42%, 16%, 2%]	3,436 [14%, 10%, 1%]	4,412 [18%, 7%, 1%]	2,760 [11%, 1%, 1%]	0 [0%, 0%, 0%]	24,682 [5% of Total]
-150m to -200m	1,254 [12%, 3%, 0%]	3,462 [33%, 5%, 1%]	1,468 [14%, 4%, 0%]	2,148 [21%, 3%, 0%]	2,038 [20%, 1%, 0%]	0 [0%, 0%, 0%]	10,369 [2% of Total]
<-200m	11,690 [3%, 28%, 2%]	36,820 [9%, 56%, 8%]	24,760 [6%, 72%, 5%]	48,626 [12%, 78%, 10%]	273,000 [69%, 96%, 56%]	0 [0%, 0%, 0%]	394,896 [81% of Total]
Total	42,167 [9% of Total]	65,658 [13% of Total]	34,221 [7% of Total]	62,153 [13% of Total]	283,495 [58% of Total]	0.000 [0% of Total]	487,693 Km^2

The designations employed and the presentation of material in the map do not imply the expression of any opinion whatsoever on the part of FAO concerning the legal or constitutional status of any country, territory or sea area, or concerning the delimitation of frontiers.

Background reference map from National Geographic. Content may not reflect National Geographic's current map policy. Sources: National Geographic, Esri, DeLorme, HERE, UNEP-WCMC, USGS, NASA, ESA, METI, NRCAN, GEBCO, NOAA, increment P Corp.

Projection: Azimuthal Equidistant
Datum: WGS 1984
False Easting: 0.0000
False Northing: 0.0000
Central Meridian: 12.4425
Latitude Of Origin: 40.4298

N W E S

Jamaica + Joint Regime: Caribbean Region

Food and Agriculture Organization of the United Nations

©FAO, 2018

Kilometers: 0 125 250 375 500

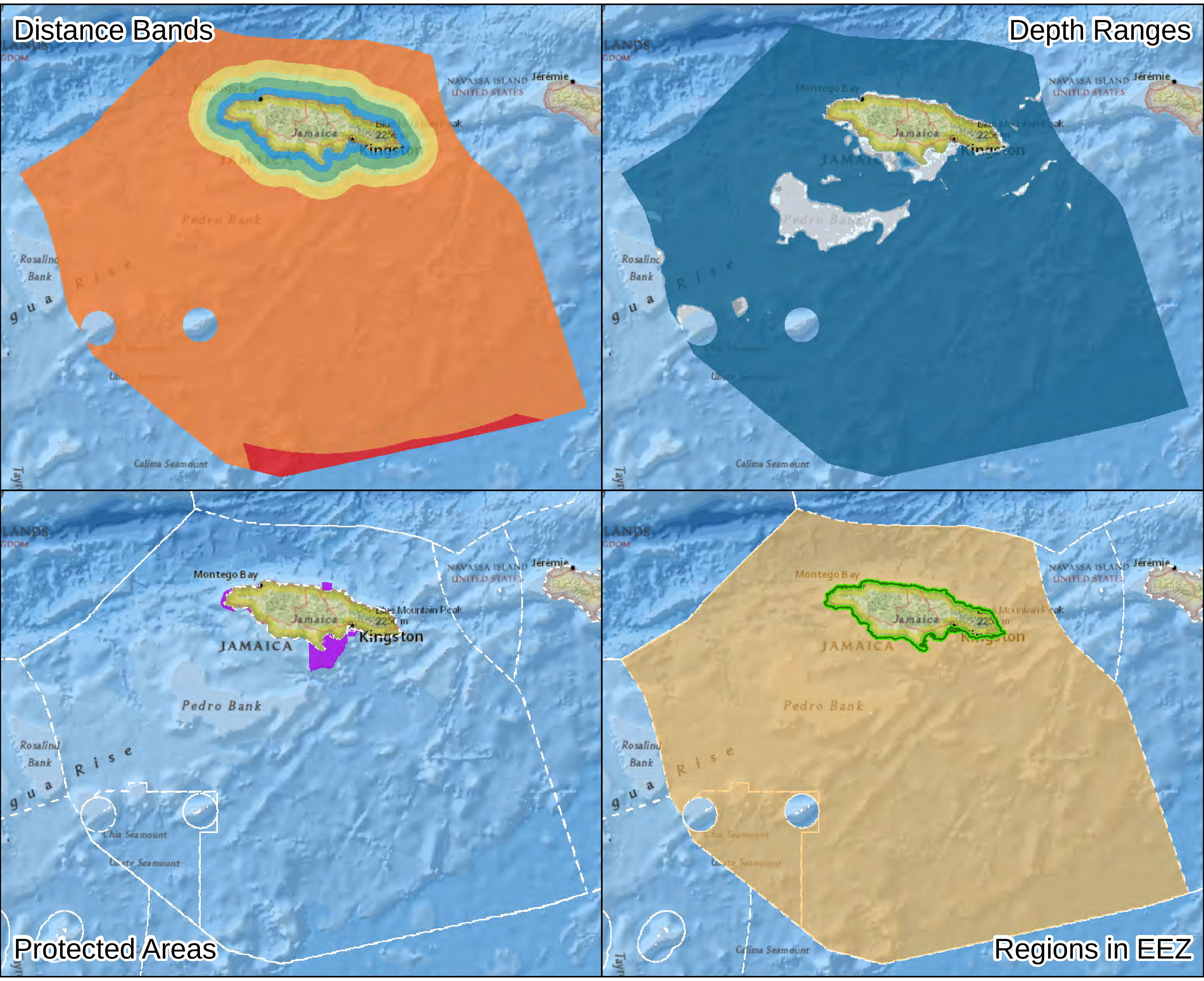

Distance Bands

- 0 to 5 NM
- 5 to 12 NM
- 12 to 16 NM
- 16 to 24 NM
- 24 to 200 NM
- > 200 NM

Depth Bands

- > -10m
- -10m to -50m
- -50m to -100m
- -100m to -150m
- -150m to -200m
- < -200m

Protected

- Protected Areas

Coastline Length

913 Km

Region

- Caribbean

All Areas in Jamaica + Joint Regime EEZ: Cell Values = Area in Km^2 [% Depth Band (Row), % Distance Band (Column), % EEZ]

	0 to 5 NM	5 to 12 NM	12 to 16 NM	16 to 24 NM	24 to 200 NM	> 200 NM	Total
> -10m	951 [41%, 16%, 0%]	193 [8%, 2%, 0%]	14 [1%, 0%, 0%]	2 [0%, 0%, 0%]	1,159 [50%, 0%, 0%]	0 [0%, 0%, 0%]	2,319 [1% of Total]
-10m to -50m	1,112 [13%, 19%, 0%]	576 [7%, 7%, 0%]	17 [0%, 0%, 0%]	10 [0%, 0%, 0%]	6,944 [80%, 3%, 3%]	0 [0%, 0%, 0%]	8,659 [3% of Total]
-50m to -100m	406 [24%, 7%, 0%]	206 [12%, 2%, 0%]	28 [2%, 1%, 0%]	7 [0%, 0%, 0%]	1,029 [61%, 0%, 0%]	0 [0%, 0%, 0%]	1,675 [1% of Total]
-100m to -150m	292 [24%, 5%, 0%]	214 [18%, 3%, 0%]	22 [2%, 0%, 0%]	11 [1%, 0%, 0%]	667 [55%, 0%, 0%]	0 [0%, 0%, 0%]	1,206 [0% of Total]
-150m to -200m	321 [24%, 6%, 0%]	228 [17%, 3%, 0%]	31 [2%, 1%, 0%]	10 [1%, 0%, 0%]	772 [57%, 0%, 0%]	0 [0%, 0%, 0%]	1,361 [0% of Total]
<-200m	2,713 [1%, 47%, 1%]	6,965 [3%, 83%, 3%]	5,078 [2%, 98%, 2%]	11,308 [4%, 100%, 4%]	225,949 [88%, 96%, 83%]	5,953 [2%, 100%, 2%]	257,967 [94% of Total]
Total	5,796 [2% of Total]	8,382 [3% of Total]	5,190 [2% of Total]	11,347 [4% of Total]	236,519 [87% of Total]	5,953 [2% of Total]	273,187 Km^2

Areas in Jamaica + Joint Regime EEZ Excluding Protected Areas: Cell Values = Area in Km^2 [% Depth Band (Row), % Distance Band (Column), % EEZ]

1,851 [1%] Km^2 Protected	0 to 5 NM	5 to 12 NM	12 to 16 NM	16 to 24 NM	24 to 200 NM	> 200 NM	Total
> -10m	615 [34%, 13%, 0%]	54 [3%, 1%, 0%]	5 [0%, 0%, 0%]	2 [0%, 0%, 0%]	1,159 [63%, 0%, 0%]	0 [0%, 0%, 0%]	1,836 [1% of Total]
-10m to -50m	653 [8%, 14%, 0%]	205 [3%, 3%, 0%]	12 [0%, 0%, 0%]	10 [0%, 0%, 0%]	6,944 [89%, 3%, 3%]	0 [0%, 0%, 0%]	7,824 [3% of Total]
-50m to -100m	324 [21%, 7%, 0%]	164 [11%, 2%, 0%]	20 [1%, 0%, 0%]	7 [0%, 0%, 0%]	1,029 [67%, 0%, 0%]	0 [0%, 0%, 0%]	1,544 [1% of Total]
-100m to -150m	227 [21%, 5%, 0%]	183 [17%, 2%, 0%]	18 [2%, 0%, 0%]	11 [1%, 0%, 0%]	667 [60%, 0%, 0%]	0 [0%, 0%, 0%]	1,107 [0% of Total]
-150m to -200m	270 [21%, 6%, 0%]	202 [16%, 3%, 0%]	23 [2%, 0%, 0%]	10 [1%, 0%, 0%]	772 [60%, 0%, 0%]	0 [0%, 0%, 0%]	1,276 [0% of Total]
<-200m	2,559 [1%, 55%, 1%]	6,936 [3%, 90%, 3%]	5,043 [2%, 98%, 2%]	11,308 [4%, 100%, 4%]	225,949 [88%, 96%, 83%]	5,953 [2%, 100%, 2%]	257,748 [95% of Total]
Total	4,650 [2% of Total]	7,744 [3% of Total]	5,122 [2% of Total]	11,347 [4% of Total]	236,519 [87% of Total]	5,953 [2% of Total]	271,336 Km^2

The designations employed and the presentation of material in the map do not imply the expression of any opinion whatsoever on the part of FAO concerning the legal or constitutional status of any country, territory or sea area, or concerning the delimitation of frontiers.

Background reference map from National Geographic. Content may not reflect National Geographic's current map policy. Sources: National Geographic, Esri, DeLorme, HERE, UNEP-WCMC, USGS, NASA, ESA, METI, NRCAN, GEBCO, NOAA, increment P Corp.

Projection: Azimuthal Equidistant
Datum: WGS 1984
False Easting: 0.0000
False Northing: 0.0000
Central Meridian: -77.4212
Latitude Of Origin: 16.7206

N W E S

Jamaica: Caribbean Region

©FAO, 2018

Food and Agriculture Organization of the United Nations

Kilometers
0 125 250 375 500

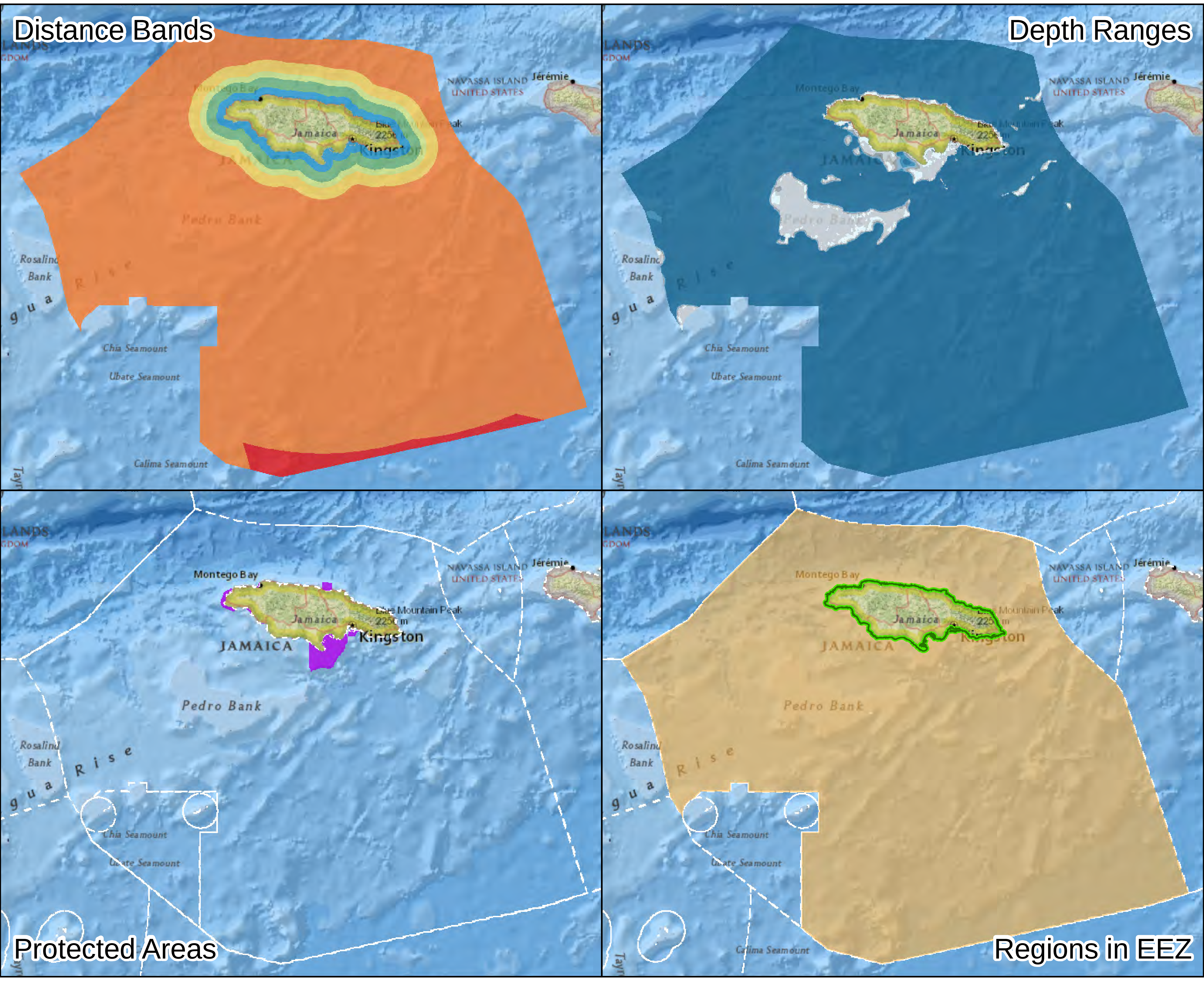

Distance Bands

- 0 to 5 NM
- 5 to 12 NM
- 12 to 16 NM
- 16 to 24 NM
- 24 to 200 NM
- > 200 NM

Depth Bands

- > -10m
- -10m to -50m
- -50m to -100m
- -100m to -150m
- -150m to -200m
- < -200m

Protected

- Protected Areas

Coastline Length

913 Km

Region

- Caribbean

All Areas in Jamaica EEZ: Cell Values = Area in Km^2 [% Depth Band (Row), % Distance Band (Column), % EEZ]

	0 to 5 NM	5 to 12 NM	12 to 16 NM	16 to 24 NM	24 to 200 NM	> 200 NM	Total
> -10m	951 [41%, 16%, 0%]	193 [8%, 2%, 0%]	14 [1%, 0%, 0%]	2 [0%, 0%, 0%]	1,159 [50%, 1%, 0%]	0 [0%, 0%, 0%]	2,319 [1% of Total]
-10m to -50m	1,112 [13%, 19%, 0%]	576 [7%, 7%, 0%]	17 [0%, 0%, 0%]	10 [0%, 0%, 0%]	6,771 [80%, 3%, 3%]	0 [0%, 0%, 0%]	8,486 [3% of Total]
-50m to -100m	406 [28%, 7%, 0%]	206 [14%, 2%, 0%]	28 [2%, 1%, 0%]	7 [0%, 0%, 0%]	819 [56%, 0%, 0%]	0 [0%, 0%, 0%]	1,465 [1% of Total]
-100m to -150m	292 [26%, 5%, 0%]	214 [19%, 3%, 0%]	22 [2%, 0%, 0%]	11 [1%, 0%, 0%]	597 [53%, 0%, 0%]	0 [0%, 0%, 0%]	1,137 [0% of Total]
-150m to -200m	321 [25%, 6%, 0%]	228 [18%, 3%, 0%]	31 [2%, 1%, 0%]	10 [1%, 0%, 0%]	698 [54%, 0%, 0%]	0 [0%, 0%, 0%]	1,287 [0% of Total]
<-200m	2,713 [1%, 47%, 1%]	6,965 [3%, 83%, 3%]	5,078 [2%, 98%, 2%]	11,308 [5%, 100%, 4%]	211,174 [87%, 95%, 82%]	5,953 [2%, 100%, 2%]	243,192 [94% of Total]
Total	5,796 [2% of Total]	8,382 [3% of Total]	5,190 [2% of Total]	11,347 [4% of Total]	221,218 [86% of Total]	5,953 [2% of Total]	257,886 Km^2

Areas in Jamaica EEZ Excluding Protected Areas: Cell Values = Area in Km^2 [% Depth Band (Row), % Distance Band (Column), % EEZ]

1,851 [1%] Km^2 Protected	0 to 5 NM	5 to 12 NM	12 to 16 NM	16 to 24 NM	24 to 200 NM	> 200 NM	Total
> -10m	615 [34%, 13%, 0%]	54 [3%, 1%, 0%]	5 [0%, 0%, 0%]	2 [0%, 0%, 0%]	1,159 [63%, 1%, 0%]	0 [0%, 0%, 0%]	1,836 [1% of Total]
-10m to -50m	653 [9%, 14%, 0%]	205 [3%, 3%, 0%]	12 [0%, 0%, 0%]	10 [0%, 0%, 0%]	6,771 [88%, 3%, 3%]	0 [0%, 0%, 0%]	7,651 [3% of Total]
-50m to -100m	324 [24%, 7%, 0%]	164 [12%, 2%, 0%]	20 [2%, 0%, 0%]	7 [0%, 0%, 0%]	819 [61%, 0%, 0%]	0 [0%, 0%, 0%]	1,335 [1% of Total]
-100m to -150m	227 [22%, 5%, 0%]	183 [18%, 2%, 0%]	18 [2%, 0%, 0%]	11 [1%, 0%, 0%]	597 [58%, 0%, 0%]	0 [0%, 0%, 0%]	1,037 [0% of Total]
-150m to -200m	270 [22%, 6%, 0%]	202 [17%, 3%, 0%]	23 [2%, 0%, 0%]	10 [1%, 0%, 0%]	698 [58%, 0%, 0%]	0 [0%, 0%, 0%]	1,202 [0% of Total]
<-200m	2,559 [1%, 55%, 1%]	6,936 [3%, 90%, 3%]	5,043 [2%, 98%, 2%]	11,308 [5%, 100%, 4%]	211,174 [87%, 95%, 82%]	5,953 [2%, 100%, 2%]	242,974 [95% of Total]
Total	4,650 [2% of Total]	7,744 [3% of Total]	5,122 [2% of Total]	11,347 [4% of Total]	221,218 [86% of Total]	5,953 [2% of Total]	256,035 Km^2

The designations employed and the presentation of material in the map do not imply the expression of any opinion whatsoever on the part of FAO concerning the legal or constitutional status of any country, territory or sea area, or concerning the delimitation of frontiers.

Background reference map from National Geographic. Content may not reflect National Geographic's current map policy. Sources: National Geographic, Esri, DeLorme, HERE, UNEP-WCMC, USGS, NASA, ESA, METI, NRCAN, GEBCO, NOAA, increment P Corp.

Projection: Azimuthal Equidistant
Datum: WGS 1984
False Easting: 0.0000
False Northing: 0.0000
Central Meridian: -77.4212
Latitude Of Origin: 16.7206

Joint Regime: Caribbean Region

©FAO, 2018

Food and Agriculture Organization of the United Nations

Kilometers: 0 | 125 | 250 | 375 | 500 | 625

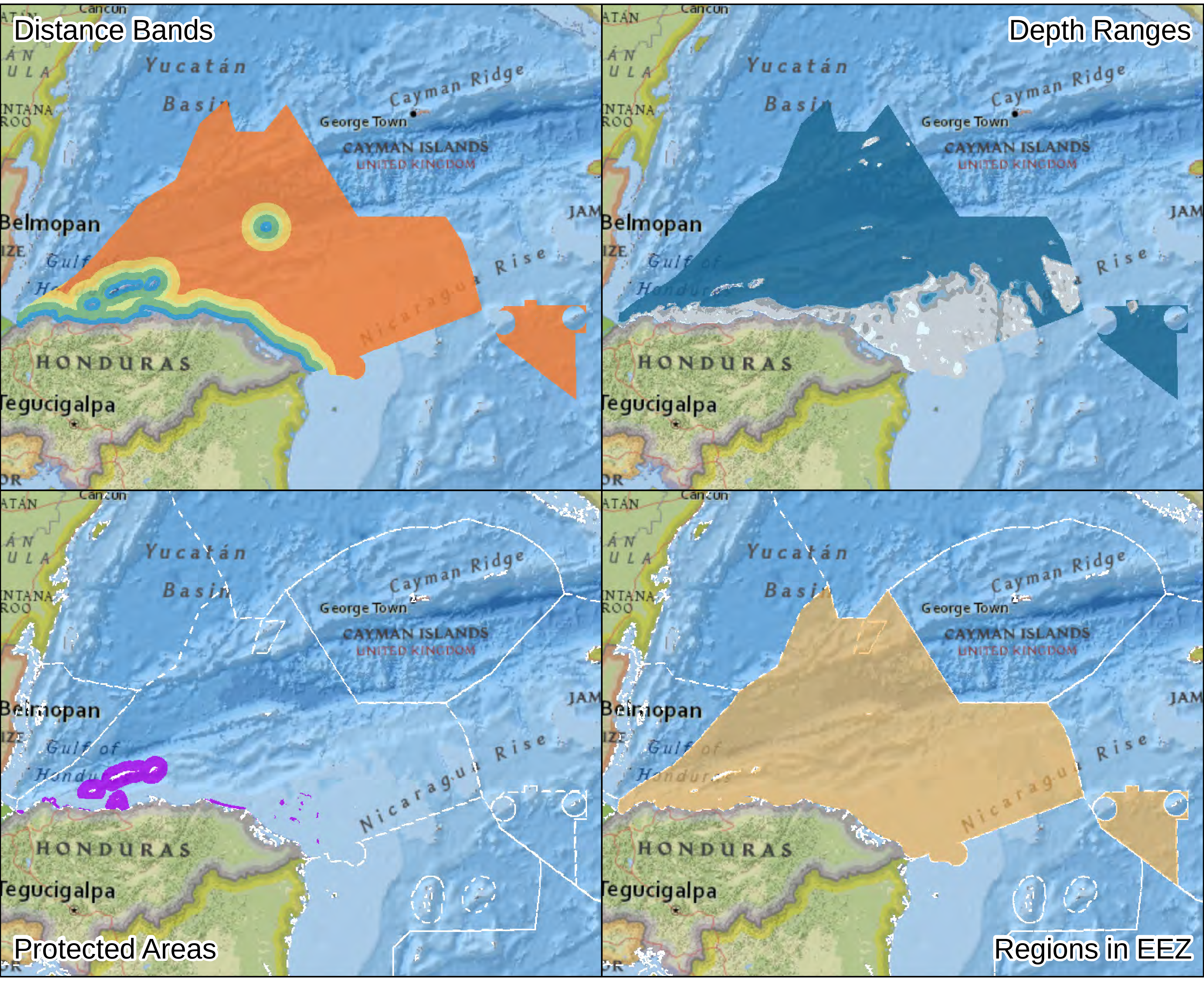

Distance Bands: 0 to 5 NM; 5 to 12 NM; 12 to 16 NM; 16 to 24 NM; 24 to 200 NM

Depth Bands: > -10m; -10m to -50m; -50m to -100m; -100m to -150m; -150m to -200m; < -200m

Protected: Protected Areas

Region: Caribbean

Coastline Length: 0 Km

All Areas in Joint Regime EEZ: Cell Values = Area in Km^2 [% Depth Band (Row), % Distance Band (Column), % EEZ]

	0 to 5 NM	5 to 12 NM	12 to 16 NM	16 to 24 NM	24 to 200 NM	> 200 NM	Total
> -10m	1,507 [30%, 17%, 1%]	360 [7%, 2%, 0%]	37 [1%, 1%, 0%]	103 [2%, 1%, 0%]	2,941 [59%, 2%, 1%]	0 [0%, 0%, 0%]	4,947 [2% of Total]
-10m to -50m	3,895 [10%, 44%, 2%]	4,299 [11%, 30%, 2%]	1,554 [4%, 21%, 1%]	2,733 [7%, 20%, 1%]	27,164 [69%, 15%, 12%]	0 [0%, 0%, 0%]	39,645 [18% of Total]
-50m to -100m	901 [10%, 10%, 0%]	1,913 [21%, 13%, 1%]	618 [7%, 9%, 0%]	99 [1%, 1%, 0%]	5,757 [62%, 3%, 3%]	0 [0%, 0%, 0%]	9,288 [4% of Total]
-100m to -150m	302 [9%, 3%, 0%]	225 [6%, 2%, 0%]	160 [5%, 2%, 0%]	41 [1%, 0%, 0%]	2,803 [79%, 2%, 1%]	0 [0%, 0%, 0%]	3,531 [2% of Total]
-150m to -200m	192 [7%, 2%, 0%]	153 [5%, 1%, 0%]	127 [4%, 2%, 0%]	42 [1%, 0%, 0%]	2,428 [83%, 1%, 1%]	0 [0%, 0%, 0%]	2,943 [1% of Total]
<-200m	1,992 [1%, 23%, 1%]	7,453 [5%, 52%, 3%]	4,736 [3%, 65%, 2%]	10,859 [7%, 78%, 5%]	139,353 [85%, 77%, 62%]	0 [0%, 0%, 0%]	164,393 [73% of Total]
Total	8,789 [4% of Total]	14,403 [6% of Total]	7,232 [3% of Total]	13,877 [6% of Total]	180,446 [80% of Total]	0.000 [0% of Total]	224,747 Km^2

Areas in Joint Regime EEZ Excluding Protected Areas: Cell Values = Area in Km^2 [% Depth Band (Row), % Distance Band (Column), % EEZ]

8,711 [4%] Km^2 Protected	0 to 5 NM	5 to 12 NM	12 to 16 NM	16 to 24 NM	24 to 200 NM	> 200 NM	Total
> -10m	1,020 [23%, 20%, 0%]	334 [8%, 3%, 0%]	36 [1%, 1%, 0%]	103 [2%, 1%, 0%]	2,893 [66%, 2%, 1%]	0 [0%, 0%, 0%]	4,385 [2% of Total]
-10m to -50m	2,829 [8%, 56%, 1%]	3,684 [10%, 37%, 2%]	1,483 [4%, 21%, 1%]	2,643 [7%, 19%, 1%]	27,030 [72%, 15%, 13%]	0 [0%, 0%, 0%]	37,670 [17% of Total]
-50m to -100m	567 [7%, 11%, 0%]	1,718 [20%, 17%, 1%]	508 [6%, 7%, 0%]	99 [1%, 1%, 0%]	5,757 [67%, 3%, 3%]	0 [0%, 0%, 0%]	8,649 [4% of Total]
-100m to -150m	144 [4%, 3%, 0%]	193 [6%, 2%, 0%]	149 [4%, 2%, 0%]	41 [1%, 0%, 0%]	2,803 [84%, 2%, 1%]	0 [0%, 0%, 0%]	3,330 [2% of Total]
-150m to -200m	68 [2%, 1%, 0%]	128 [5%, 1%, 0%]	116 [4%, 2%, 0%]	41 [1%, 0%, 0%]	2,428 [87%, 1%, 1%]	0 [0%, 0%, 0%]	2,781 [1% of Total]
<-200m	399 [0%, 8%, 0%]	3,961 [2%, 40%, 2%]	4,651 [3%, 67%, 2%]	10,857 [7%, 79%, 5%]	139,353 [88%, 77%, 65%]	0 [0%, 0%, 0%]	159,221 [74% of Total]
Total	5,026 [2% of Total]	10,019 [5% of Total]	6,943 [3% of Total]	13,785 [6% of Total]	180,264 [83% of Total]	0.000 [0% of Total]	216,036 Km^2

The designations employed and the presentation of material in the map do not imply the expression of any opinion whatsoever on the part of FAO concerning the legal or constitutional status of any country, territory or sea area, or concerning the delimitation of frontiers.

Background reference map from National Geographic. Content may not reflect National Geographic's current map policy. Sources: National Geographic, Esri, DeLorme, HERE, UNEP-WCMC, USGS, NASA, ESA, METI, NRCAN, GEBCO, NOAA, increment P Corp.

Projection: Azimuthal Equidistant
Datum: WGS 1984
False Easting: 0.0000
False Northing: 0.0000
Central Meridian: -83.3327
Latitude Of Origin: 17.0171

Food and Agriculture Organization of the United Nations

Latvia: Baltic Region

©FAO, 2018

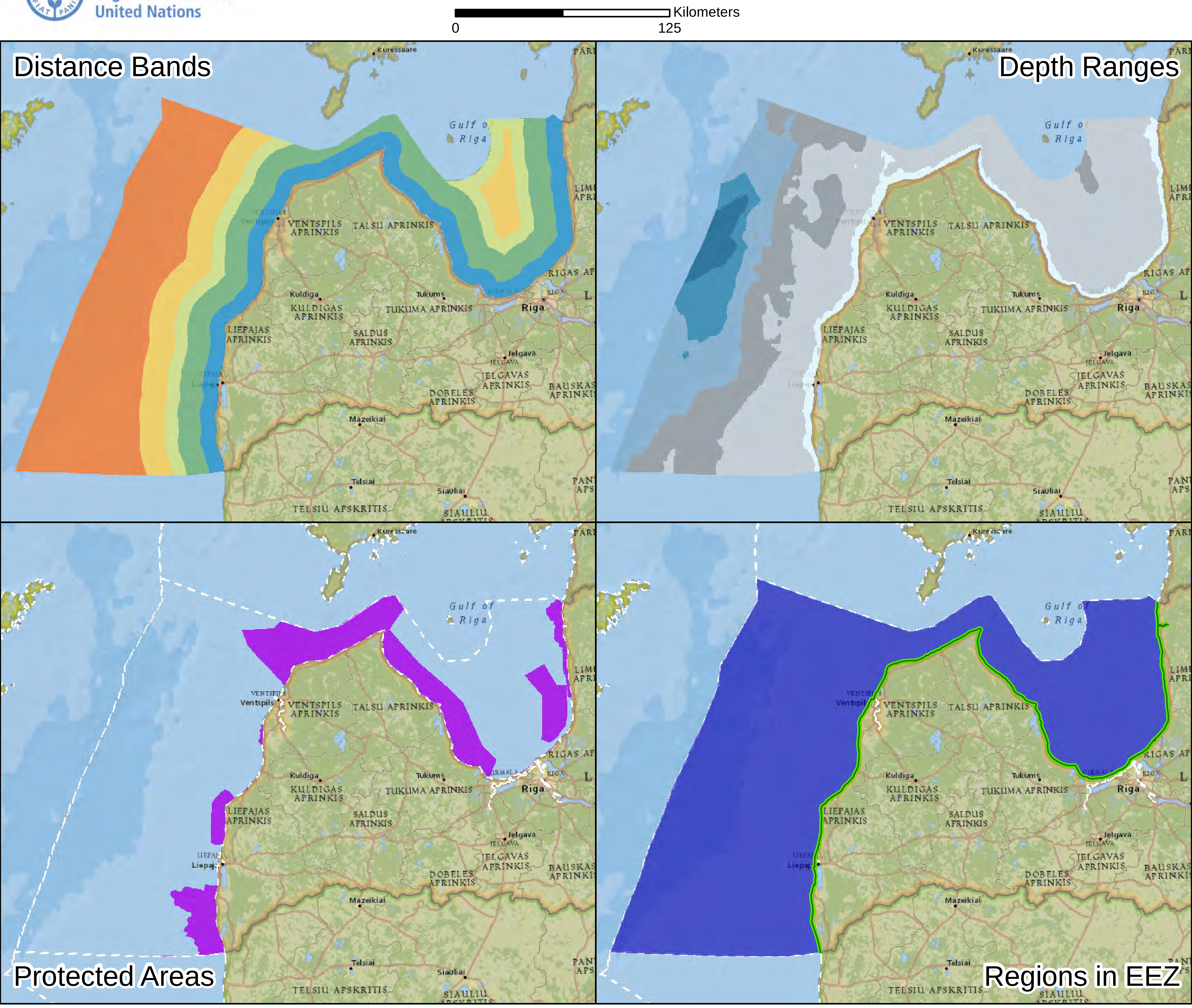

Distance Bands
- 0 to 5 NM
- 5 to 12 NM
- 12 to 16 NM
- 16 to 24 NM
- 24 to 200 NM

Depth Bands
- > -10m
- -10m to -50m
- -50m to -100m
- -100m to -150m
- -150m to -200m
- < -200m

Protected
- Protected Areas

Coastline Length
- 510 Km

Region
- Baltic

All Areas in Latvia EEZ: Cell Values = Area in Km² [% Depth Band (Row), % Distance Band (Column), % EEZ]

	0 to 5 NM	5 to 12 NM	12 to 16 NM	16 to 24 NM	24 to 200 NM	> 200 NM	Total
> -10m	1,639 [98%, 37%, 6%]	40 [2%, 1%, 0%]	1 [0%, 0%, 0%]	0 [0%, 0%, 0%]	0 [0%, 0%, 0%]	0 [0%, 0%, 0%]	1,679 [6% of Total]
-10m to -50m	2,841 [21%, 63%, 10%]	5,618 [41%, 96%, 20%]	2,376 [17%, 80%, 8%]	2,211 [16%, 55%, 8%]	579 [4%, 5%, 2%]	0 [0%, 0%, 0%]	13,624 [48% of Total]
-50m to -100m	0 [0%, 0%, 0%]	208 [4%, 4%, 1%]	578 [10%, 20%, 2%]	1,799 [31%, 45%, 6%]	3,138 [55%, 29%, 11%]	0 [0%, 0%, 0%]	5,722 [20% of Total]
-100m to -150m	0 [0%, 0%, 0%]	0 [0%, 0%, 0%]	0 [0%, 0%, 0%]	2 [0%, 0%, 0%]	4,751 [100%, 44%, 17%]	0 [0%, 0%, 0%]	4,753 [17% of Total]
-150m to -200m	0 [0%, 0%, 0%]	0 [0%, 0%, 0%]	0 [0%, 0%, 0%]	0 [0%, 0%, 0%]	1,583 [100%, 15%, 6%]	0 [0%, 0%, 0%]	1,583 [6% of Total]
<-200m	0 [0%, 0%, 0%]	0 [0%, 0%, 0%]	0 [0%, 0%, 0%]	0 [0%, 0%, 0%]	753 [100%, 7%, 3%]	0 [0%, 0%, 0%]	753 [3% of Total]
Total	4,480 [16% of Total]	5,866 [21% of Total]	2,955 [11% of Total]	4,012 [14% of Total]	10,803 [38% of Total]	0.000 [0% of Total]	28,116 Km²

Areas in Latvia EEZ Excluding Protected Areas: Cell Values = Area in Km² [% Depth Band (Row), % Distance Band (Column), % EEZ]

4,812 [17%] Km² Protected	0 to 5 NM	5 to 12 NM	12 to 16 NM	16 to 24 NM	24 to 200 NM	> 200 NM	Total
> -10m	601 [100%, 36%, 3%]	0 [0%, 0%, 0%]	0 [0%, 0%, 0%]	0 [0%, 0%, 0%]	0 [0%, 0%, 0%]	0 [0%, 0%, 0%]	601 [3% of Total]
-10m to -50m	1,056 [11%, 64%, 5%]	3,897 [39%, 95%, 17%]	2,204 [22%, 80%, 9%]	2,171 [22%, 55%, 9%]	579 [6%, 5%, 2%]	0 [0%, 0%, 0%]	9,907 [43% of Total]
-50m to -100m	0 [0%, 0%, 0%]	208 [4%, 5%, 1%]	563 [10%, 20%, 2%]	1,797 [31%, 45%, 8%]	3,138 [55%, 29%, 13%]	0 [0%, 0%, 0%]	5,706 [24% of Total]
-100m to -150m	0 [0%, 0%, 0%]	0 [0%, 0%, 0%]	0 [0%, 0%, 0%]	2 [0%, 0%, 0%]	4,751 [100%, 44%, 20%]	0 [0%, 0%, 0%]	4,753 [20% of Total]
-150m to -200m	0 [0%, 0%, 0%]	0 [0%, 0%, 0%]	0 [0%, 0%, 0%]	0 [0%, 0%, 0%]	1,583 [100%, 15%, 7%]	0 [0%, 0%, 0%]	1,583 [7% of Total]
<-200m	0 [0%, 0%, 0%]	0 [0%, 0%, 0%]	0 [0%, 0%, 0%]	0 [0%, 0%, 0%]	753 [100%, 7%, 3%]	0 [0%, 0%, 0%]	753 [3% of Total]
Total	1,657 [7% of Total]	4,106 [18% of Total]	2,767 [12% of Total]	3,971 [17% of Total]	10,803 [46% of Total]	0.000 [0% of Total]	23,304 Km²

The designations employed and the presentation of material in the map do not imply the expression of any opinion whatsoever on the part of FAO concerning the legal or constitutional status of any country, territory or sea area, or concerning the delimitation of frontiers.

Background reference map from National Geographic. Content may not reflect National Geographic's current map policy. Sources: National Geographic, Esri, DeLorme, HERE, UNEP-WCMC, USGS, NASA, ESA, METI, NRCAN, GEBCO, NOAA, increment P Corp.

Projection: Azimuthal Equidistant
Datum: WGS 1984
False Easting: 0.0000
False Northing: 0.0000
Central Meridian: 21.7550
Latitude Of Origin: 57.0329

N W E S

Food and Agriculture Organization of the United Nations

Lebanon: Mediterranean Region

©FAO, 2018

Kilometers
0 125

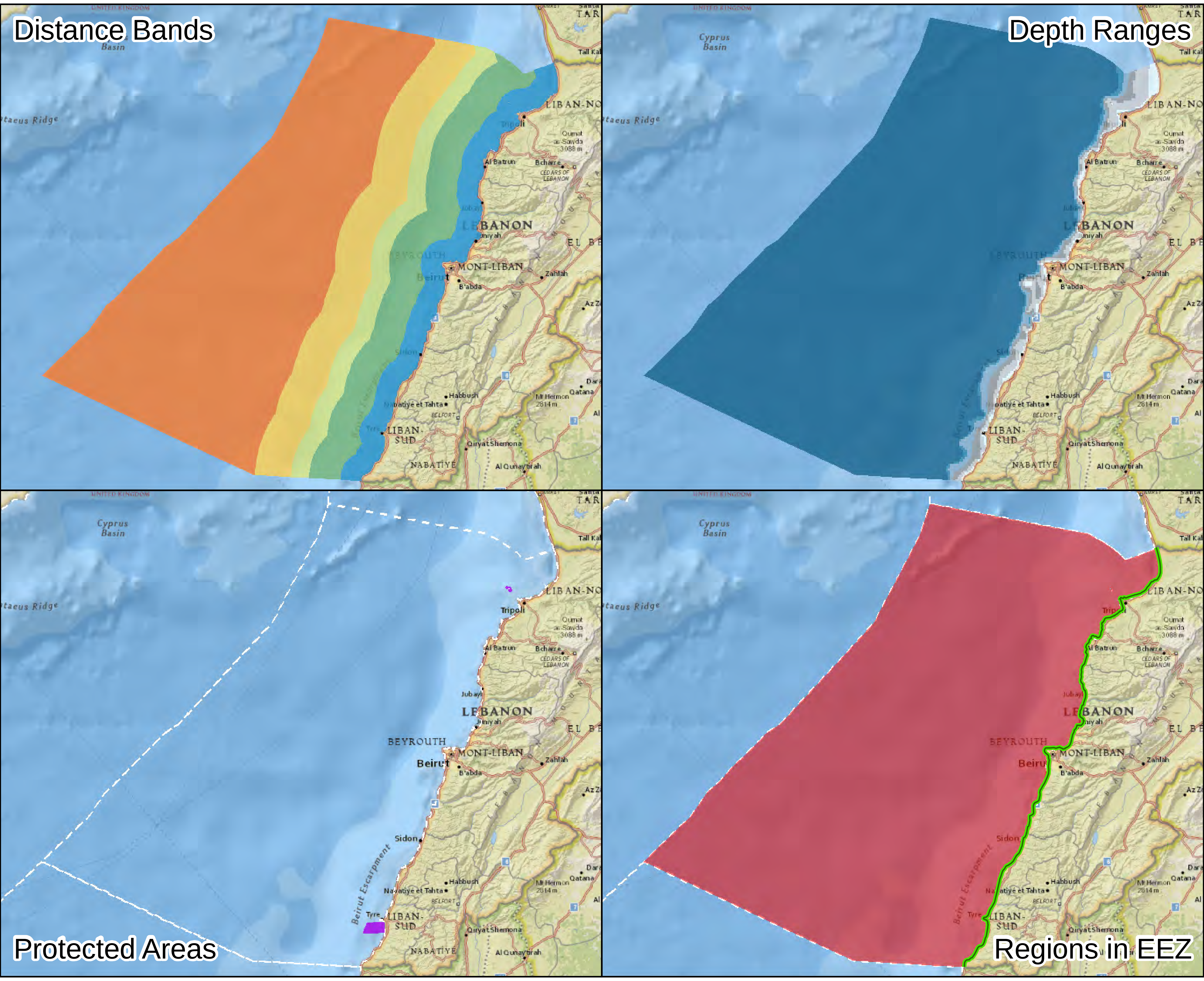

Distance Bands	Depth Bands	Protected	Region
0 to 5 NM	> -10m	Protected Areas	Mediterranean
5 to 12 NM	-10m to -50m		
12 to 16 NM	-50m to -100m		
16 to 24 NM	-100m to -150m		
24 to 200 NM	-150m to -200m		
	< -200m		

Coastline Length

225 Km

All Areas in Lebanon EEZ: Cell Values = Area in Km^2 [% Depth Band (Row), % Distance Band (Column), % EEZ]

	0 to 5 NM	5 to 12 NM	12 to 16 NM	16 to 24 NM	24 to 200 NM	> 200 NM	Total
> -10m	163 [99%, 9%, 1%]	1 [1%, 0%, 0%]	0 [0%, 0%, 0%]	0 [0%, 0%, 0%]	0 [0%, 0%, 0%]	0 [0%, 0%, 0%]	164 [1% of Total]
-10m to -50m	380 [99%, 20%, 2%]	5 [1%, 0%, 0%]	0 [0%, 0%, 0%]	0 [0%, 0%, 0%]	0 [0%, 0%, 0%]	0 [0%, 0%, 0%]	385 [2% of Total]
-50m to -100m	293 [94%, 16%, 1%]	17 [6%, 1%, 0%]	0 [0%, 0%, 0%]	0 [0%, 0%, 0%]	0 [0%, 0%, 0%]	0 [0%, 0%, 0%]	310 [2% of Total]
-100m to -150m	249 [92%, 13%, 1%]	22 [8%, 1%, 0%]	0 [0%, 0%, 0%]	0 [0%, 0%, 0%]	0 [0%, 0%, 0%]	0 [0%, 0%, 0%]	271 [1% of Total]
-150m to -200m	142 [82%, 8%, 1%]	31 [18%, 1%, 0%]	0 [0%, 0%, 0%]	0 [0%, 0%, 0%]	0 [0%, 0%, 0%]	0 [0%, 0%, 0%]	172 [1% of Total]
<-200m	642 [3%, 34%, 3%]	2,530 [13%, 97%, 13%]	1,485 [8%, 100%, 7%]	2,947 [16%, 100%, 15%]	11,273 [60%, 100%, 56%]	0 [0%, 0%, 0%]	18,877 [94% of Total]
Total	1,868 [9% of Total]	2,606 [13% of Total]	1,485 [7% of Total]	2,947 [15% of Total]	11,273 [56% of Total]	0.000 [0% of Total]	20,179 Km^2

Areas in Lebanon EEZ Excluding Protected Areas: Cell Values = Area in Km^2 [% Depth Band (Row), % Distance Band (Column), % EEZ]

41 [0%] Km^2 Protected	0 to 5 NM	5 to 12 NM	12 to 16 NM	16 to 24 NM	24 to 200 NM	> 200 NM	Total
> -10m	158 [99%, 9%, 1%]	1 [1%, 0%, 0%]	0 [0%, 0%, 0%]	0 [0%, 0%, 0%]	0 [0%, 0%, 0%]	0 [0%, 0%, 0%]	159 [1% of Total]
-10m to -50m	370 [99%, 20%, 2%]	5 [1%, 0%, 0%]	0 [0%, 0%, 0%]	0 [0%, 0%, 0%]	0 [0%, 0%, 0%]	0 [0%, 0%, 0%]	375 [2% of Total]
-50m to -100m	281 [94%, 15%, 1%]	17 [6%, 1%, 0%]	0 [0%, 0%, 0%]	0 [0%, 0%, 0%]	0 [0%, 0%, 0%]	0 [0%, 0%, 0%]	298 [1% of Total]
-100m to -150m	236 [91%, 13%, 1%]	22 [9%, 1%, 0%]	0 [0%, 0%, 0%]	0 [0%, 0%, 0%]	0 [0%, 0%, 0%]	0 [0%, 0%, 0%]	258 [1% of Total]
-150m to -200m	142 [82%, 8%, 1%]	31 [18%, 1%, 0%]	0 [0%, 0%, 0%]	0 [0%, 0%, 0%]	0 [0%, 0%, 0%]	0 [0%, 0%, 0%]	172 [1% of Total]
<-200m	642 [3%, 35%, 3%]	2,530 [13%, 97%, 13%]	1,485 [8%, 100%, 7%]	2,947 [16%, 100%, 15%]	11,273 [60%, 100%, 56%]	0 [0%, 0%, 0%]	18,877 [94% of Total]
Total	1,828 [9% of Total]	2,606 [13% of Total]	1,485 [7% of Total]	2,947 [15% of Total]	11,273 [56% of Total]	0.000 [0% of Total]	20,139 Km^2

The designations employed and the presentation of material in the map do not imply the expression of any opinion whatsoever on the part of FAO concerning the legal or constitutional status of any country, territory or sea area, or concerning the delimitation of frontiers.

Background reference map from National Geographic. Content may not reflect National Geographic's current map policy. Sources: National Geographic, Esri, DeLorme, HERE, UNEP-WCMC, USGS, NASA, ESA, METI, NRCAN, GEBCO, NOAA, increment P Corp.

Projection: Azimuthal Equidistant
Datum: WGS 1984
False Easting: 0.0000
False Northing: 0.0000
Central Meridian: 34.8431
Latitude Of Origin: 33.9538

N W E S

Libya: Mediterranean Region

©FAO, 2018

Food and Agriculture Organization of the United Nations

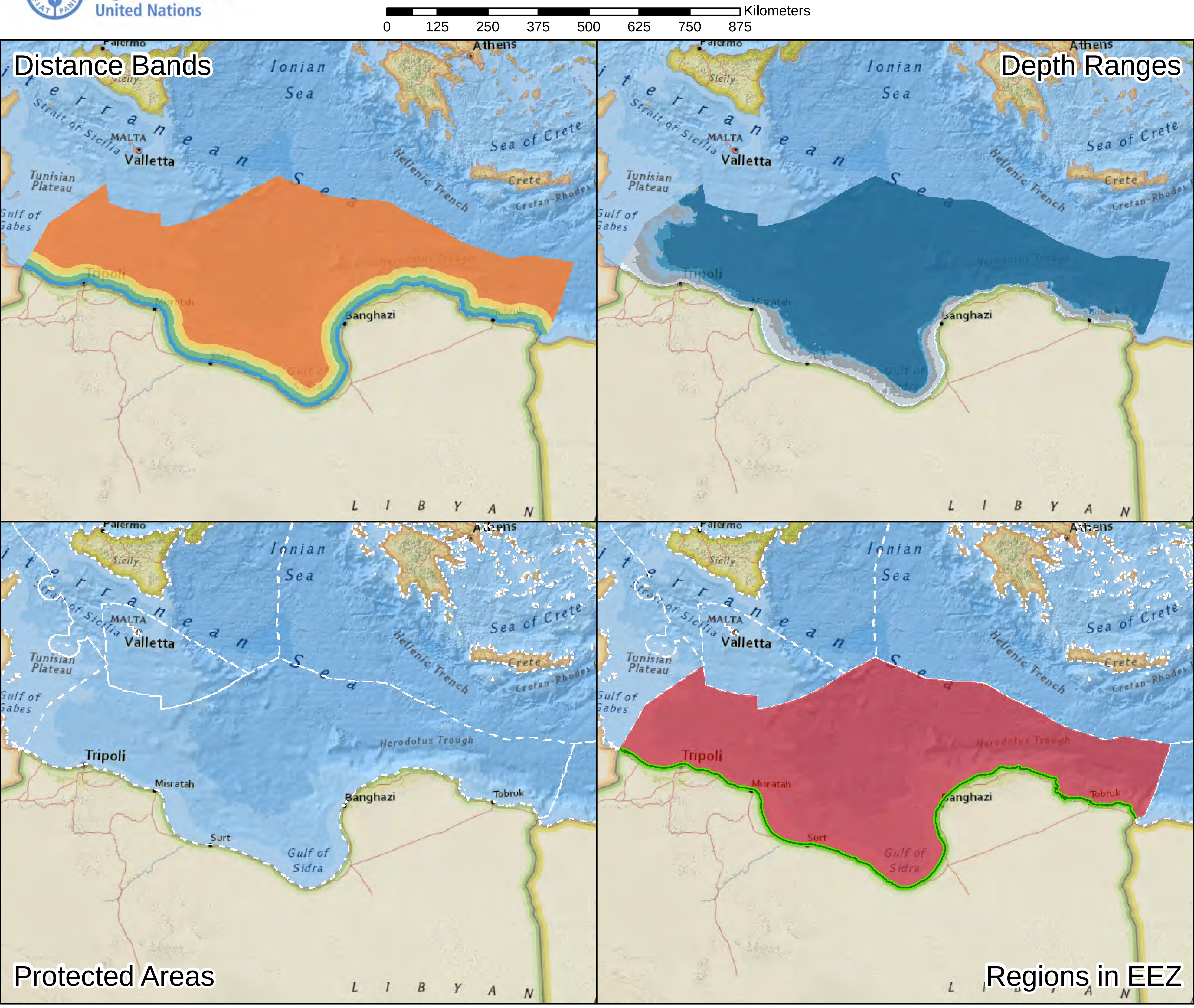

Distance Bands

- 0 to 5 NM
- 5 to 12 NM
- 12 to 16 NM
- 16 to 24 NM
- 24 to 200 NM

Depth Bands

- > -10m
- -10m to -50m
- -50m to -100m
- -100m to -150m
- -150m to -200m
- < -200m

Protected

- Protected Areas

Region

- Mediterranean

Coastline Length

1,836 Km

All Areas in Libya EEZ: Cell Values = Area in Km^2 [% Depth Band (Row), % Distance Band (Column), % EEZ]

	0 to 5 NM	5 to 12 NM	12 to 16 NM	16 to 24 NM	24 to 200 NM	> 200 NM	Total
> -10m	3,092 [98%, 19%, 1%]	58 [2%, 0%, 0%]	6 [0%, 0%, 0%]	0 [0%, 0%, 0%]	0 [0%, 0%, 0%]	0 [0%, 0%, 0%]	3,155 [1% of Total]
-10m to -50m	9,332 [55%, 58%, 3%]	6,130 [36%, 27%, 2%]	801 [5%, 6%, 0%]	513 [3%, 2%, 0%]	330 [2%, 0%, 0%]	0 [0%, 0%, 0%]	17,107 [5% of Total]
-50m to -100m	2,566 [12%, 16%, 1%]	7,502 [36%, 34%, 2%]	3,651 [18%, 29%, 1%]	3,260 [16%, 13%, 1%]	3,635 [18%, 1%, 1%]	0 [0%, 0%, 0%]	20,614 [6% of Total]
-100m to -150m	437 [3%, 3%, 0%]	3,035 [20%, 14%, 1%]	1,982 [13%, 16%, 1%]	2,963 [20%, 12%, 1%]	6,652 [44%, 2%, 2%]	0 [0%, 0%, 0%]	15,069 [4% of Total]
-150m to -200m	182 [2%, 1%, 0%]	1,469 [14%, 7%, 0%]	1,631 [15%, 13%, 0%]	3,025 [29%, 12%, 1%]	4,216 [40%, 1%, 1%]	0 [0%, 0%, 0%]	10,522 [3% of Total]
<-200m	483 [0%, 3%, 0%]	4,162 [1%, 19%, 1%]	4,656 [2%, 37%, 1%]	15,524 [5%, 61%, 4%]	272,773 [92%, 95%, 75%]	0 [0%, 0%, 0%]	297,597 [82% of Total]
Total	16,091 [4% of Total]	22,357 [6% of Total]	12,726 [3% of Total]	25,285 [7% of Total]	287,606 [79% of Total]	0.000 [0% of Total]	364,065 Km^2

Areas in Libya EEZ Excluding Protected Areas: Cell Values = Area in Km^2 [% Depth Band (Row), % Distance Band (Column), % EEZ]

0 [0%] Km^2 Protected	0 to 5 NM	5 to 12 NM	12 to 16 NM	16 to 24 NM	24 to 200 NM	> 200 NM	Total
> -10m	3,092 [98%, 19%, 1%]	58 [2%, 0%, 0%]	6 [0%, 0%, 0%]	0 [0%, 0%, 0%]	0 [0%, 0%, 0%]	0 [0%, 0%, 0%]	3,155 [1% of Total]
-10m to -50m	9,332 [55%, 58%, 3%]	6,130 [36%, 27%, 2%]	801 [5%, 6%, 0%]	513 [3%, 2%, 0%]	330 [2%, 0%, 0%]	0 [0%, 0%, 0%]	17,107 [5% of Total]
-50m to -100m	2,566 [12%, 16%, 1%]	7,502 [36%, 34%, 2%]	3,651 [18%, 29%, 1%]	3,260 [16%, 13%, 1%]	3,635 [18%, 1%, 1%]	0 [0%, 0%, 0%]	20,614 [6% of Total]
-100m to -150m	437 [3%, 3%, 0%]	3,035 [20%, 14%, 1%]	1,982 [13%, 16%, 1%]	2,963 [20%, 12%, 1%]	6,652 [44%, 2%, 2%]	0 [0%, 0%, 0%]	15,069 [4% of Total]
-150m to -200m	182 [2%, 1%, 0%]	1,469 [14%, 7%, 0%]	1,631 [15%, 13%, 0%]	3,025 [29%, 12%, 1%]	4,216 [40%, 1%, 1%]	0 [0%, 0%, 0%]	10,522 [3% of Total]
<-200m	483 [0%, 3%, 0%]	4,162 [1%, 19%, 1%]	4,656 [2%, 37%, 1%]	15,524 [5%, 61%, 4%]	272,773 [92%, 95%, 75%]	0 [0%, 0%, 0%]	297,597 [82% of Total]
Total	16,091 [4% of Total]	22,357 [6% of Total]	12,726 [3% of Total]	25,285 [7% of Total]	287,606 [79% of Total]	0.000 [0% of Total]	364,065 Km^2

The designations employed and the presentation of material in the map do not imply the expression of any opinion whatsoever on the part of FAO concerning the legal or constitutional status of any country, territory or sea area, or concerning the delimitation of frontiers.

Background reference map from National Geographic. Content may not reflect National Geographic's current map policy. Sources: National Geographic, Esri, DeLorme, HERE, UNEP-WCMC, USGS, NASA, ESA, METI, NRCAN, GEBCO, NOAA, increment P Corp.

Projection: Azimuthal Equidistant
Datum: WGS 1984
False Easting: 0.0000
False Northing: 0.0000
Central Meridian: 18.8768
Latitude Of Origin: 32.8461

Food and Agriculture Organization of the United Nations

Lithuania: Baltic Region

©FAO, 2018

Kilometers
0 90

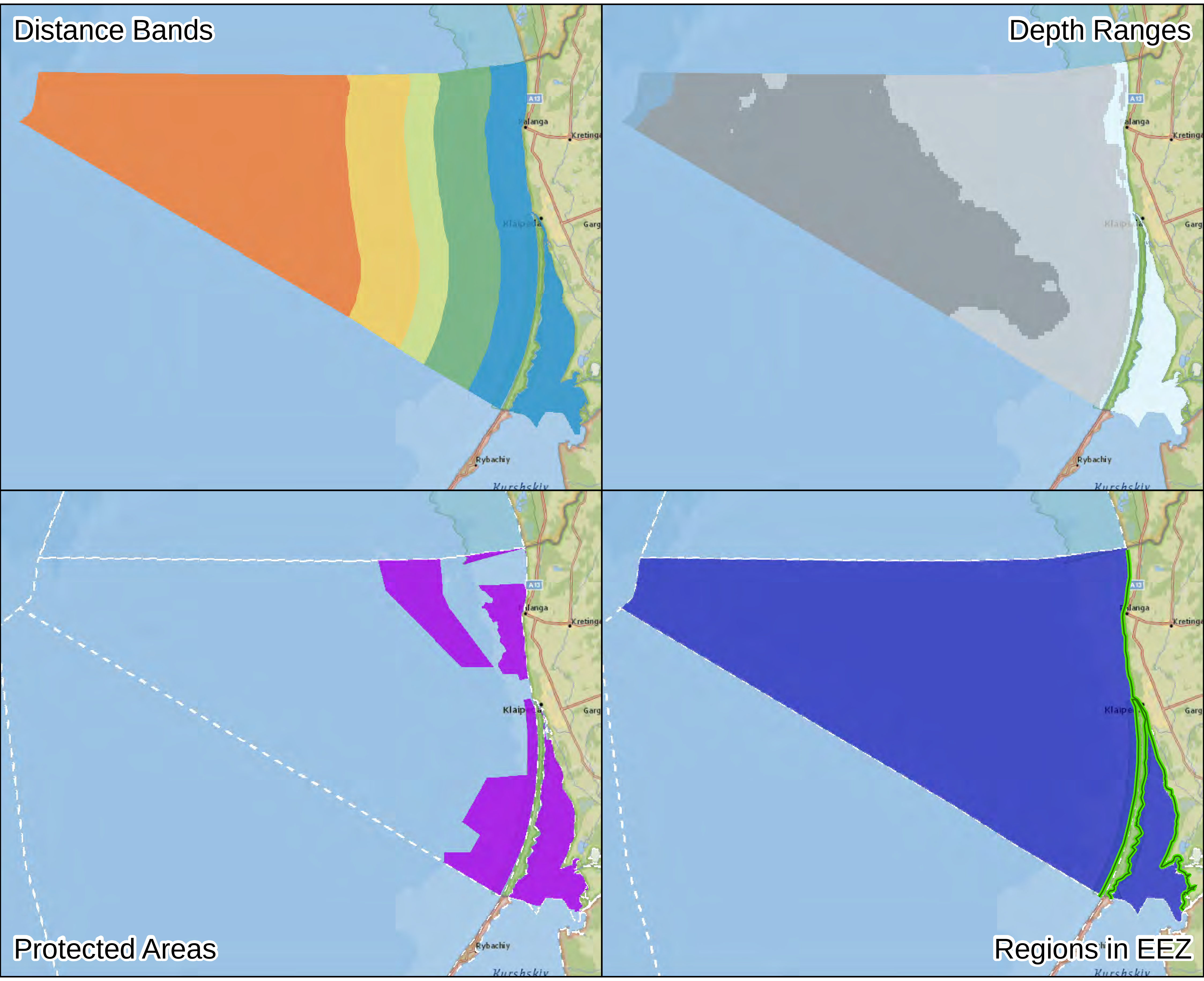

Distance Bands
- 0 to 5 NM
- 5 to 12 NM
- 12 to 16 NM
- 16 to 24 NM
- 24 to 200 NM

Depth Bands
- > -10m
- -10m to -50m
- -50m to -100m
- -100m to -150m

Protected
- Protected Areas

Coastline Length
- 224 Km

Region
- Baltic

All Areas in Lithuania EEZ: Cell Values = Area in Km² [% Depth Band (Row), % Distance Band (Column), % EEZ]

	0 to 5 NM	5 to 12 NM	12 to 16 NM	16 to 24 NM	24 to 200 NM	> 200 NM	Total
> -10m	514 [100%, 44%, 8%]	0 [0%, 0%, 0%]	0 [0%, 0%, 0%]	0 [0%, 0%, 0%]	0 [0%, 0%, 0%]	0 [0%, 0%, 0%]	514 [8% of Total]
-10m to -50m	659 [23%, 56%, 10%]	967 [33%, 94%, 14%]	400 [14%, 74%, 6%]	589 [20%, 60%, 9%]	286 [10%, 9%, 4%]	0 [0%, 0%, 0%]	2,902 [43% of Total]
-50m to -100m	0 [0%, 0%, 0%]	59 [2%, 6%, 1%]	137 [4%, 26%, 2%]	390 [12%, 40%, 6%]	2,655 [82%, 87%, 39%]	0 [0%, 0%, 0%]	3,241 [48% of Total]
-100m to -150m	0 [0%, 0%, 0%]	0 [0%, 0%, 0%]	0 [0%, 0%, 0%]	0 [0%, 0%, 0%]	100 [100%, 3%, 1%]	0 [0%, 0%, 0%]	100 [1% of Total]
-150m to -200m	0 [0%, 0%, 0%]	0 [0%, 0%, 0%]	0 [0%, 0%, 0%]	0 [0%, 0%, 0%]	0 [0%, 0%, 0%]	0 [0%, 0%, 0%]	0.000 [0% of Total]
<-200m	0 [0%, 0%, 0%]	0 [0%, 0%, 0%]	0 [0%, 0%, 0%]	0 [0%, 0%, 0%]	0 [0%, 0%, 0%]	0 [0%, 0%, 0%]	0.000 [0% of Total]
Total	1,173 [17% of Total]	1,026 [15% of Total]	538 [8% of Total]	979 [14% of Total]	3,042 [45% of Total]	0.000 [0% of Total]	6,758 Km²

Areas in Lithuania EEZ Excluding Protected Areas: Cell Values = Area in Km² [% Depth Band (Row), % Distance Band (Column), % EEZ]

1,296 [19%] Km² Protected	0 to 5 NM	5 to 12 NM	12 to 16 NM	16 to 24 NM	24 to 200 NM	> 200 NM	Total
> -10m	42 [100%, 13%, 1%]	0 [0%, 0%, 0%]	0 [0%, 0%, 0%]	0 [0%, 0%, 0%]	0 [0%, 0%, 0%]	0 [0%, 0%, 0%]	42 [1% of Total]
-10m to -50m	283 [14%, 87%, 5%]	702 [34%, 92%, 13%]	279 [13%, 67%, 5%]	527 [25%, 57%, 10%]	286 [14%, 9%, 5%]	0 [0%, 0%, 0%]	2,079 [38% of Total]
-50m to -100m	0 [0%, 0%, 0%]	59 [2%, 8%, 1%]	137 [4%, 33%, 3%]	390 [12%, 43%, 7%]	2,655 [82%, 87%, 49%]	0 [0%, 0%, 0%]	3,241 [59% of Total]
-100m to -150m	0 [0%, 0%, 0%]	0 [0%, 0%, 0%]	0 [0%, 0%, 0%]	0 [0%, 0%, 0%]	100 [100%, 3%, 2%]	0 [0%, 0%, 0%]	100 [2% of Total]
-150m to -200m	0 [0%, 0%, 0%]	0 [0%, 0%, 0%]	0 [0%, 0%, 0%]	0 [0%, 0%, 0%]	0 [0%, 0%, 0%]	0 [0%, 0%, 0%]	0.000 [0% of Total]
<-200m	0 [0%, 0%, 0%]	0 [0%, 0%, 0%]	0 [0%, 0%, 0%]	0 [0%, 0%, 0%]	0 [0%, 0%, 0%]	0 [0%, 0%, 0%]	0.000 [0% of Total]
Total	325 [6% of Total]	761 [14% of Total]	417 [8% of Total]	917 [17% of Total]	3,042 [56% of Total]	0.000 [0% of Total]	5,462 Km²

The designations employed and the presentation of material in the map do not imply the expression of any opinion whatsoever on the part of FAO concerning the legal or constitutional status of any country, territory or sea area, or concerning the delimitation of frontiers.

Background reference map from National Geographic. Content may not reflect National Geographic's current map policy. Sources: National Geographic, Esri, DeLorme, HERE, UNEP-WCMC, USGS, NASA, ESA, METI, NRCAN, GEBCO, NOAA, increment P Corp.

Projection: Azimuthal Equidistant
Datum: WGS 1984
False Easting: 0.0000
False Northing: 0.0000
Central Meridian: 20.1605
Latitude Of Origin: 55.6456

N W E S

Food and Agriculture Organization of the United Nations

Malta: Mediterranean Region

©FAO, 2018

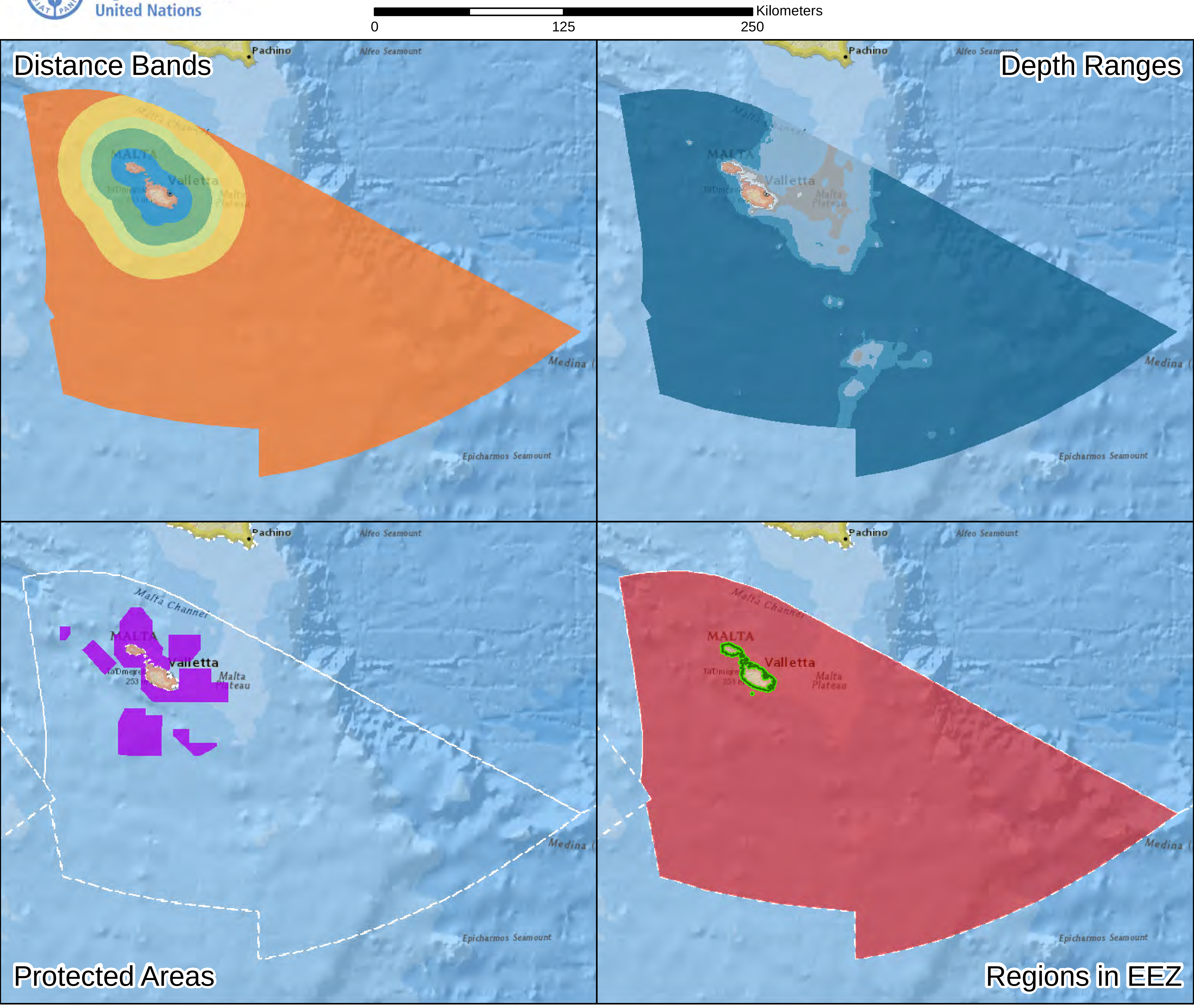

Distance Bands		Depth Bands		Protected	Region
0 to 5 NM	16 to 24 NM	> -10m	-100m to -150m	Protected Areas	Mediterranean
5 to 12 NM	24 to 200 NM	-10m to -50m	-150m to -200m		
12 to 16 NM		-50m to -100m	< -200m		

Coastline Length: 246 Km

All Areas in Malta EEZ: Cell Values = Area in Km^2 [% Depth Band (Row), % Distance Band (Column), % EEZ]

	0 to 5 NM	5 to 12 NM	12 to 16 NM	16 to 24 NM	24 to 200 NM	> 200 NM	Total
> -10m	56 [100%, 4%, 0%]	0 [0%, 0%, 0%]	0 [0%, 0%, 0%]	0 [0%, 0%, 0%]	0 [0%, 0%, 0%]	0 [0%, 0%, 0%]	56 [0% of Total]
-10m to -50m	83 [92%, 6%, 0%]	7 [8%, 0%, 0%]	0 [0%, 0%, 0%]	0 [0%, 0%, 0%]	0 [0%, 0%, 0%]	0 [0%, 0%, 0%]	90 [0% of Total]
-50m to -100m	193 [13%, 14%, 0%]	208 [14%, 8%, 0%]	206 [14%, 10%, 0%]	552 [37%, 11%, 1%]	346 [23%, 1%, 1%]	0 [0%, 0%, 0%]	1,504 [3% of Total]
-100m to -150m	282 [7%, 21%, 1%]	545 [14%, 21%, 1%]	416 [10%, 21%, 1%]	1,047 [26%, 22%, 2%]	1,723 [43%, 4%, 3%]	0 [0%, 0%, 0%]	4,011 [8% of Total]
-150m to -200m	412 [18%, 31%, 1%]	212 [9%, 8%, 0%]	95 [4%, 5%, 0%]	118 [5%, 2%, 0%]	1,478 [64%, 4%, 3%]	0 [0%, 0%, 0%]	2,315 [4% of Total]
<-200m	311 [1%, 23%, 1%]	1,673 [4%, 63%, 3%]	1,260 [3%, 64%, 2%]	3,119 [7%, 65%, 6%]	38,583 [86%, 92%, 73%]	0 [0%, 0%, 0%]	44,946 [85% of Total]
Total	1,336 [3% of Total]	2,645 [5% of Total]	1,976 [4% of Total]	4,836 [9% of Total]	42,129 [80% of Total]	0.000 [0% of Total]	52,922 Km^2

Areas in Malta EEZ Excluding Protected Areas: Cell Values = Area in Km^2 [% Depth Band (Row), % Distance Band (Column), % EEZ]

3,480 [7%] Km^2 Protected	0 to 5 NM	5 to 12 NM	12 to 16 NM	16 to 24 NM	24 to 200 NM	> 200 NM	Total
> -10m	13 [100%, 4%, 0%]	0 [0%, 0%, 0%]	0 [0%, 0%, 0%]	0 [0%, 0%, 0%]	0 [0%, 0%, 0%]	0 [0%, 0%, 0%]	13 [0% of Total]
-10m to -50m	10 [100%, 3%, 0%]	0 [0%, 0%, 0%]	0 [0%, 0%, 0%]	0 [0%, 0%, 0%]	0 [0%, 0%, 0%]	0 [0%, 0%, 0%]	10 [0% of Total]
-50m to -100m	18 [2%, 6%, 0%]	15 [2%, 1%, 0%]	109 [11%, 8%, 0%]	498 [51%, 12%, 1%]	346 [35%, 1%, 1%]	0 [0%, 0%, 0%]	985 [2% of Total]
-100m to -150m	54 [2%, 18%, 0%]	214 [6%, 15%, 0%]	310 [9%, 22%, 1%]	1,038 [31%, 25%, 2%]	1,720 [52%, 4%, 3%]	0 [0%, 0%, 0%]	3,336 [7% of Total]
-150m to -200m	102 [5%, 34%, 0%]	185 [9%, 13%, 0%]	91 [5%, 6%, 0%]	110 [6%, 3%, 0%]	1,474 [75%, 4%, 3%]	0 [0%, 0%, 0%]	1,962 [4% of Total]
<-200m	103 [0%, 34%, 0%]	1,029 [2%, 71%, 2%]	929 [2%, 65%, 2%]	2,522 [6%, 61%, 5%]	38,551 [89%, 92%, 78%]	0 [0%, 0%, 0%]	43,134 [87% of Total]
Total	301 [1% of Total]	1,444 [3% of Total]	1,439 [3% of Total]	4,167 [8% of Total]	42,091 [85% of Total]	0.000 [0% of Total]	49,441 Km^2

The designations employed and the presentation of material in the map do not imply the expression of any opinion whatsoever on the part of FAO concerning the legal or constitutional status of any country, territory or sea area, or concerning the delimitation of frontiers.

Background reference map from National Geographic. Content may not reflect National Geographic's current map policy. Sources: National Geographic, Esri, DeLorme, HERE, UNEP-WCMC, USGS, NASA, ESA, METI, NRCAN, GEBCO, NOAA, increment P Corp.

Projection: Azimuthal Equidistant
Datum: WGS 1984
False Easting: 0.0000
False Northing: 0.0000
Central Meridian: 15.4571
Latitude Of Origin: 35.3628

Mexico: Gulf of Mexico and Caribbean Regions

©FAO, 2018

Food and Agriculture Organization of the United Nations

Kilometers: 0, 125, 250, 375, 500, 625, 750, 875

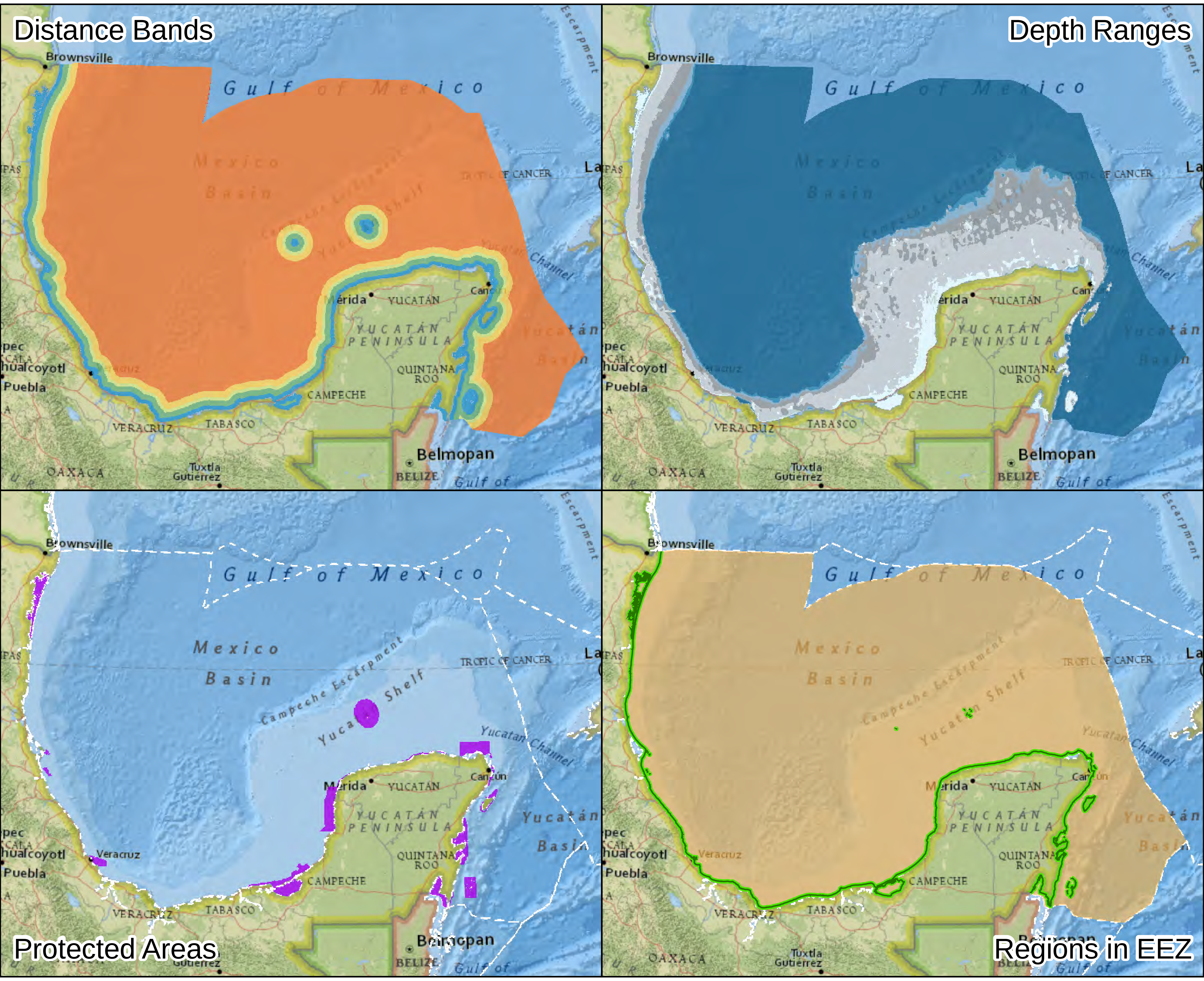

Distance Bands		Depth Bands		Protected	Region
0 to 5 NM	16 to 24 NM	> -10m	-100m to -150m	Protected Areas	Caribbean
5 to 12 NM	24 to 200 NM	-10m to -50m	-150m to -200m		
12 to 16 NM	> 200 NM	-50m to -100m	< -200m		

Coastline Length: 4,818 Km

All Areas in Mexico EEZ: Cell Values = Area in Km^2 [% Depth Band (Row), % Distance Band (Column), % EEZ]

	0 to 5 NM	5 to 12 NM	12 to 16 NM	16 to 24 NM	24 to 200 NM	> 200 NM	Total
> -10m	13,683 [58%, 41%, 2%]	5,708 [24%, 15%, 1%]	1,627 [7%, 7%, 0%]	1,430 [6%, 3%, 0%]	1,246 [5%, 0%, 0%]	0 [0%, 0%, 0%]	23,693 [3% of Total]
-10m to -50m	12,825 [11%, 39%, 2%]	17,730 [15%, 45%, 2%]	9,425 [8%, 43%, 1%]	15,773 [14%, 35%, 2%]	61,072 [52%, 9%, 7%]	0 [0%, 0%, 0%]	116,826 [14% of Total]
-50m to -100m	2,488 [4%, 8%, 0%]	6,258 [10%, 16%, 1%]	3,857 [6%, 17%, 0%]	8,750 [15%, 19%, 1%]	38,775 [64%, 6%, 5%]	0 [0%, 0%, 0%]	60,128 [7% of Total]
-100m to -150m	844 [4%, 3%, 0%]	1,631 [8%, 4%, 0%]	1,239 [6%, 6%, 0%]	3,009 [15%, 7%, 0%]	13,125 [66%, 2%, 2%]	0 [0%, 0%, 0%]	19,848 [2% of Total]
-150m to -200m	592 [5%, 2%, 0%]	461 [4%, 1%, 0%]	635 [5%, 3%, 0%]	1,090 [9%, 2%, 0%]	9,015 [76%, 1%, 1%]	0 [0%, 0%, 0%]	11,793 [1% of Total]
<-200m	2,684 [0%, 8%, 0%]	7,369 [1%, 19%, 1%]	5,342 [1%, 24%, 1%]	15,543 [3%, 34%, 2%]	566,279 [95%, 82%, 68%]	79 [0%, 100%, 0%]	597,296 [72% of Total]
Total	33,116 [4% of Total]	39,157 [5% of Total]	22,124 [3% of Total]	45,595 [5% of Total]	689,512 [83% of Total]	79 [0% of Total]	829,583 Km^2

Areas in Mexico EEZ Excluding Protected Areas: Cell Values = Area in Km^2 [% Depth Band (Row), % Distance Band (Column), % EEZ]

18,984 [2%] Km^2 Protected	0 to 5 NM	5 to 12 NM	12 to 16 NM	16 to 24 NM	24 to 200 NM	> 200 NM	Total
> -10m	5,693 [43%, 28%, 1%]	3,453 [26%, 10%, 0%]	1,531 [11%, 7%, 0%]	1,430 [11%, 3%, 0%]	1,246 [9%, 0%, 0%]	0 [0%, 0%, 0%]	13,354 [2% of Total]
-10m to -50m	10,087 [9%, 49%, 1%]	16,266 [15%, 48%, 2%]	8,969 [8%, 42%, 1%]	15,765 [14%, 35%, 2%]	61,072 [54%, 9%, 8%]	0 [0%, 0%, 0%]	112,159 [14% of Total]
-50m to -100m	1,631 [3%, 8%, 0%]	4,584 [8%, 14%, 1%]	3,696 [6%, 17%, 0%]	8,750 [15%, 19%, 1%]	38,775 [68%, 6%, 5%]	0 [0%, 0%, 0%]	57,435 [7% of Total]
-100m to -150m	541 [3%, 3%, 0%]	1,556 [8%, 5%, 0%]	1,205 [6%, 6%, 0%]	3,009 [15%, 7%, 0%]	13,125 [68%, 2%, 2%]	0 [0%, 0%, 0%]	19,436 [2% of Total]
-150m to -200m	381 [3%, 2%, 0%]	454 [4%, 1%, 0%]	635 [5%, 3%, 0%]	1,090 [9%, 2%, 0%]	9,015 [78%, 1%, 1%]	0 [0%, 0%, 0%]	11,574 [1% of Total]
<-200m	2,127 [0%, 10%, 0%]	7,271 [1%, 22%, 1%]	5,342 [1%, 25%, 1%]	15,543 [3%, 34%, 2%]	566,279 [95%, 82%, 70%]	79 [0%, 100%, 0%]	596,641 [74% of Total]
Total	20,460 [3% of Total]	33,584 [4% of Total]	21,377 [3% of Total]	45,587 [6% of Total]	689,512 [85% of Total]	79 [0% of Total]	810,599 Km^2

The designations employed and the presentation of material in the map do not imply the expression of any opinion whatsoever on the part of FAO concerning the legal or constitutional status of any country, territory or sea area, or concerning the delimitation of frontiers.

Background reference map from National Geographic. Content may not reflect National Geographic's current map policy. Sources: National Geographic, Esri, DeLorme, HERE, UNEP-WCMC, USGS, NASA, ESA, METI, NRCAN, GEBCO, NOAA, increment P Corp.

Projection: Azimuthal Equidistant
Datum: WGS 1984
False Easting: 0.0000
False Northing: 0.0000
Central Meridian: -91.2527
Latitude Of Origin: 21.8964

Food and Agriculture Organization of the United Nations

Monaco: Mediterranean Region

©FAO, 2018

0 — 70 Kilometers

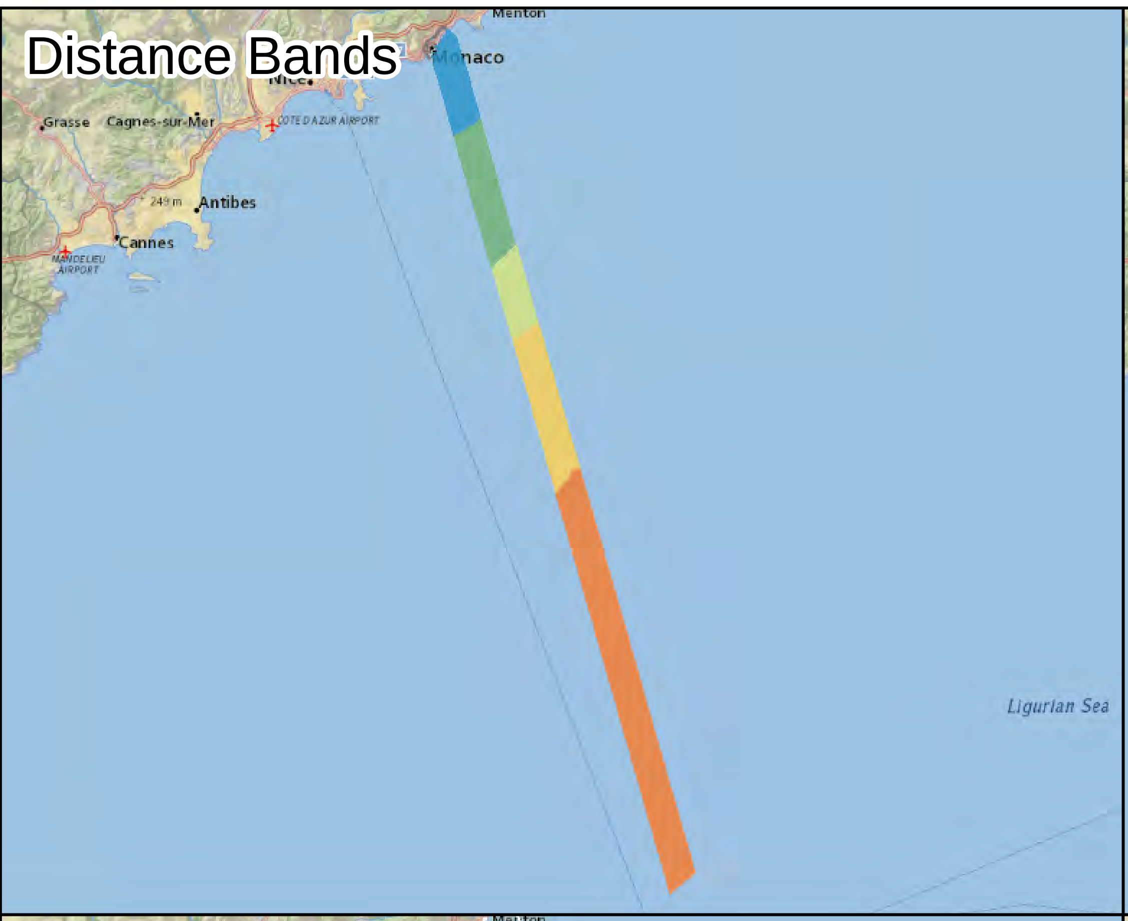

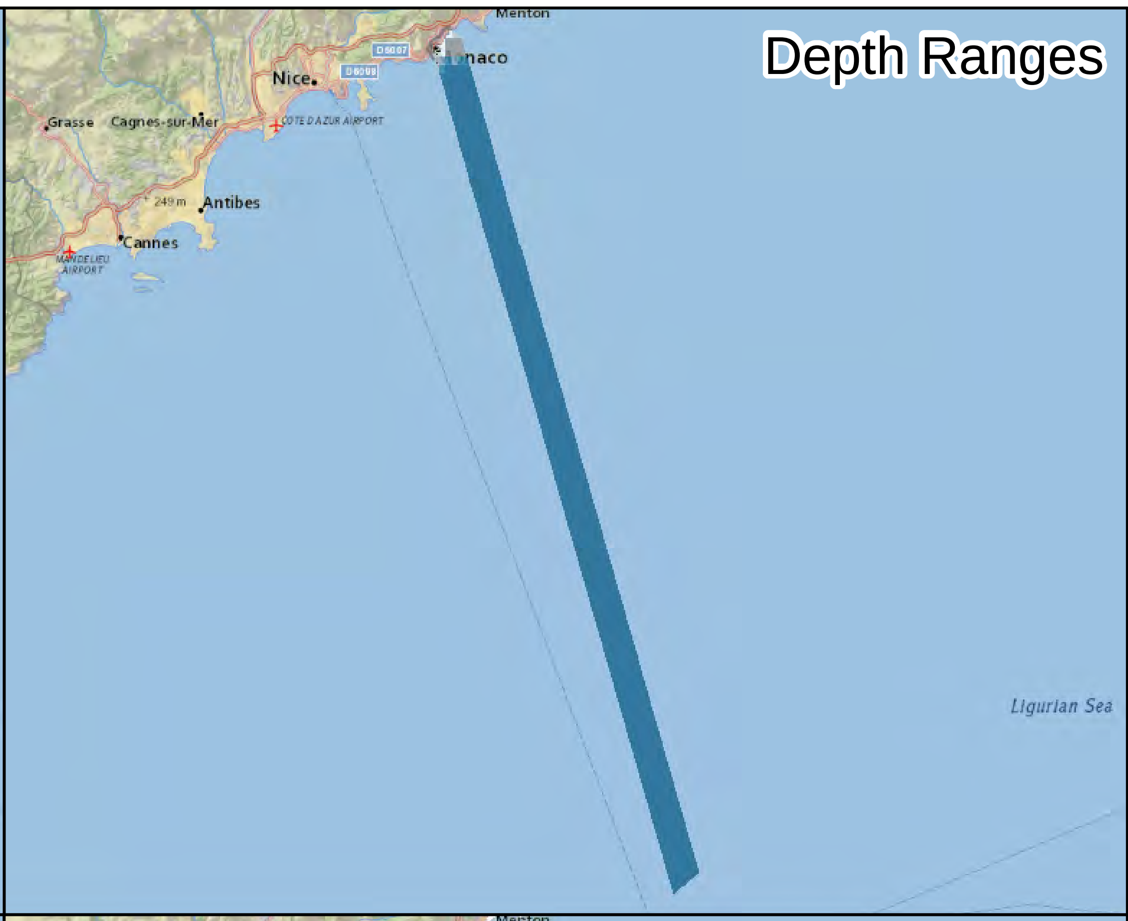

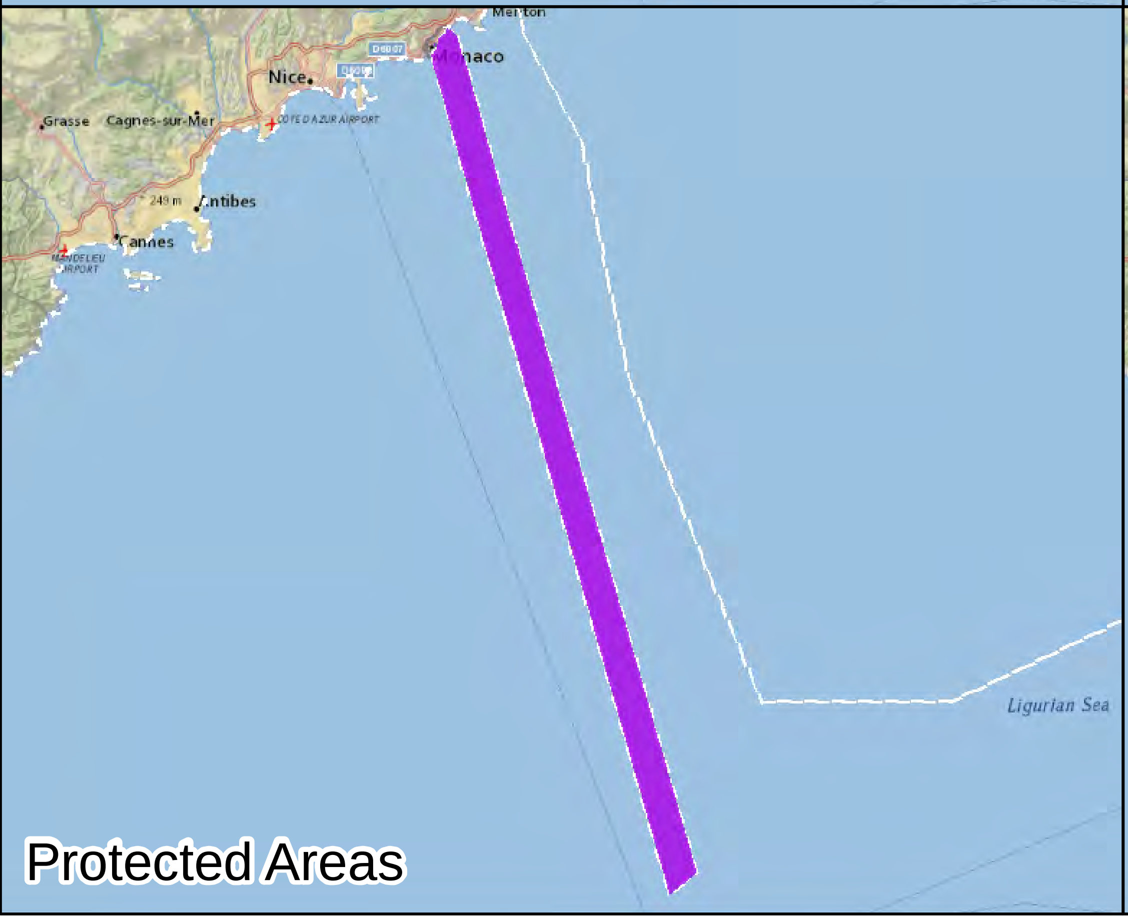

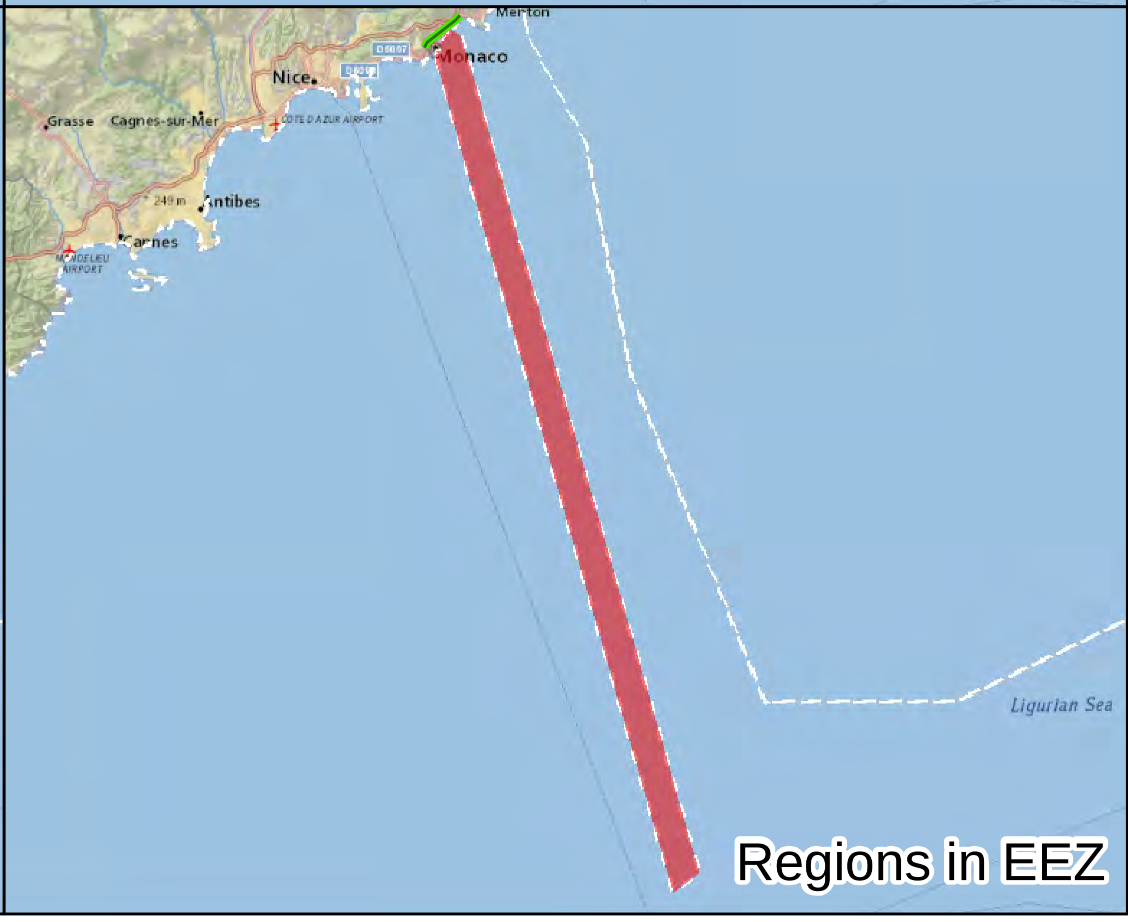

Distance Bands
- 0 to 5 NM
- 5 to 12 NM
- 12 to 16 NM
- 16 to 24 NM
- 24 to 200 NM

Depth Bands
- > -10m
- -10m to -50m
- -50m to -100m
- -100m to -150m
- -150m to -200m
- < -200m

Protected
- Protected Areas

Coastline Length
- 5 Km

Region
- Mediterranean

All Areas in Monaco EEZ: Cell Values = Area in Km^2 [% Depth Band (Row), % Distance Band (Column), % EEZ]

	0 to 5 NM	5 to 12 NM	12 to 16 NM	16 to 24 NM	24 to 200 NM	> 200 NM	Total
> -10m	1 [100%, 5%, 0%]	0 [0%, 0%, 0%]	0 [0%, 0%, 0%]	0 [0%, 0%, 0%]	0 [0%, 0%, 0%]	0 [0%, 0%, 0%]	1 [0% of Total]
-10m to -50m	0.746 [100%, 3%, 0%]	0 [0%, 0%, 0%]	0 [0%, 0%, 0%]	0 [0%, 0%, 0%]	0 [0%, 0%, 0%]	0 [0%, 0%, 0%]	0.746 [0% of Total]
-50m to -100m	4 [100%, 12%, 1%]	0 [0%, 0%, 0%]	0 [0%, 0%, 0%]	0 [0%, 0%, 0%]	0 [0%, 0%, 0%]	0 [0%, 0%, 0%]	4 [1% of Total]
-100m to -150m	1 [100%, 4%, 0%]	0 [0%, 0%, 0%]	0 [0%, 0%, 0%]	0 [0%, 0%, 0%]	0 [0%, 0%, 0%]	0 [0%, 0%, 0%]	1 [0% of Total]
-150m to -200m	2 [100%, 7%, 1%]	0 [0%, 0%, 0%]	0 [0%, 0%, 0%]	0 [0%, 0%, 0%]	0 [0%, 0%, 0%]	0 [0%, 0%, 0%]	2 [1% of Total]
<-200m	20 [7%, 69%, 7%]	44 [16%, 100%, 15%]	25 [9%, 100%, 9%]	50 [18%, 100%, 18%]	138 [50%, 100%, 48%]	0 [0%, 0%, 0%]	278 [97% of Total]
Total	30 [10% of Total]	44 [15% of Total]	25 [9% of Total]	50 [18% of Total]	138 [48% of Total]	0.000 [0% of Total]	287 Km^2

Areas in Monaco EEZ Excluding Protected Areas: Cell Values = Area in Km^2 [% Depth Band (Row), % Distance Band (Column), % EEZ]

287 [100%] Km^2 Protected	0 to 5 NM	5 to 12 NM	12 to 16 NM	16 to 24 NM	24 to 200 NM	> 200 NM	Total
> -10m	0.021 [100%, 100%, 100%]	0 [0%, 0%, 0%]	0 [0%, 0%, 0%]	0 [0%, 0%, 0%]	0 [0%, 0%, 0%]	0 [0%, 0%, 0%]	0.021 [100% of Total]
-10m to -50m	0 [0%, 0%, 0%]	0 [0%, 0%, 0%]	0 [0%, 0%, 0%]	0 [0%, 0%, 0%]	0 [0%, 0%, 0%]	0 [0%, 0%, 0%]	0.000 [0% of Total]
-50m to -100m	0 [0%, 0%, 0%]	0 [0%, 0%, 0%]	0 [0%, 0%, 0%]	0 [0%, 0%, 0%]	0 [0%, 0%, 0%]	0 [0%, 0%, 0%]	0.000 [0% of Total]
-100m to -150m	0 [0%, 0%, 0%]	0 [0%, 0%, 0%]	0 [0%, 0%, 0%]	0 [0%, 0%, 0%]	0 [0%, 0%, 0%]	0 [0%, 0%, 0%]	0.000 [0% of Total]
-150m to -200m	0 [0%, 0%, 0%]	0 [0%, 0%, 0%]	0 [0%, 0%, 0%]	0 [0%, 0%, 0%]	0 [0%, 0%, 0%]	0 [0%, 0%, 0%]	0.000 [0% of Total]
<-200m	0 [0%, 0%, 0%]	0 [0%, 0%, 0%]	0 [0%, 0%, 0%]	0 [0%, 0%, 0%]	0 [0%, 0%, 0%]	0 [0%, 0%, 0%]	0.000 [0% of Total]
Total	0.021 [100% of Total]	0.000 [0% of Total]	0.000 [0% of Total]	0.000 [0% of Total]	0.000 [0% of Total]	0.000 [0% of Total]	0.021 Km^2

The designations employed and the presentation of material in the map do not imply the expression of any opinion whatsoever on the part of FAO concerning the legal or constitutional status of any country, territory or sea area, or concerning the delimitation of frontiers.

Background reference map from National Geographic. Content may not reflect National Geographic's current map policy. Sources: National Geographic, Esri, DeLorme, HERE, UNEP-WCMC, USGS, NASA, ESA, METI, NRCAN, GEBCO, NOAA, increment P Corp.

Projection: Azimuthal Equidistant
Datum: WGS 1984
False Easting: 0.0000
False Northing: 0.0000
Central Meridian: 7.5884
Latitude Of Origin: 43.3481

Montenegro: Mediterranean Region

Food and Agriculture Organization of the United Nations

©FAO, 2018

Kilometers
0 100

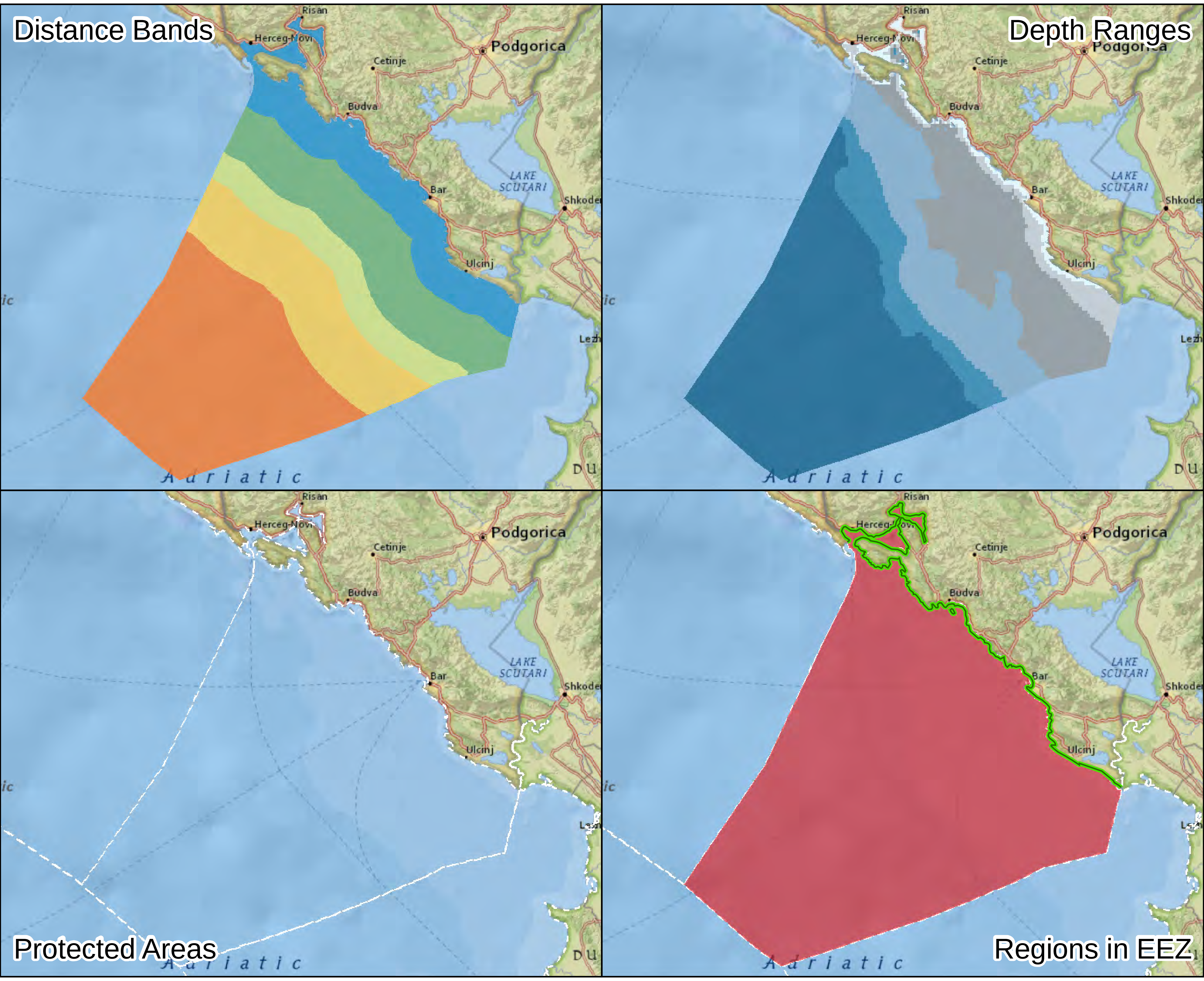

Distance Bands

- 0 to 5 NM
- 16 to 24 NM
- 5 to 12 NM
- 24 to 200 NM
- 12 to 16 NM

Depth Bands

- > -10m
- -100m to -150m
- -10m to -50m
- -150m to -200m
- -50m to -100m
- < -200m

Protected

- Protected Areas

Coastline Length

218 Km

Region

- Mediterranean

All Areas in Montenegro EEZ: Cell Values = Area in Km^2 [% Depth Band (Row), % Distance Band (Column), % EEZ]

	0 to 5 NM	5 to 12 NM	12 to 16 NM	16 to 24 NM	24 to 200 NM	> 200 NM	Total
> -10m	134 [100%, 13%, 2%]	0 [0%, 0%, 0%]	0 [0%, 0%, 0%]	0 [0%, 0%, 0%]	0 [0%, 0%, 0%]	0 [0%, 0%, 0%]	134 [2% of Total]
-10m to -50m	238 [100%, 24%, 4%]	1 [0%, 0%, 0%]	0 [0%, 0%, 0%]	0 [0%, 0%, 0%]	0 [0%, 0%, 0%]	0 [0%, 0%, 0%]	239 [4% of Total]
-50m to -100m	473 [37%, 48%, 7%]	737 [57%, 60%, 12%]	82 [6%, 13%, 1%]	0 [0%, 0%, 0%]	0 [0%, 0%, 0%]	0 [0%, 0%, 0%]	1,292 [20% of Total]
-100m to -150m	148 [13%, 15%, 2%]	370 [32%, 30%, 6%]	364 [32%, 58%, 6%]	266 [23%, 24%, 4%]	0 [0%, 0%, 0%]	0 [0%, 0%, 0%]	1,148 [18% of Total]
-150m to -200m	2 [0%, 0%, 0%]	84 [15%, 7%, 1%]	138 [25%, 22%, 2%]	323 [59%, 29%, 5%]	0.612 [0%, 0%, 0%]	0 [0%, 0%, 0%]	547 [9% of Total]
<-200m	0 [0%, 0%, 0%]	30 [1%, 2%, 0%]	42 [1%, 7%, 1%]	517 [17%, 47%, 8%]	2,406 [80%, 100%, 38%]	0 [0%, 0%, 0%]	2,995 [47% of Total]
Total	995 [16% of Total]	1,222 [19% of Total]	626 [10% of Total]	1,105 [17% of Total]	2,407 [38% of Total]	0.000 [0% of Total]	6,355 Km^2

Areas in Montenegro EEZ Excluding Protected Areas: Cell Values = Area in Km^2 [% Depth Band (Row), % Distance Band (Column), % EEZ]

0 [0%] Km^2 Protected	0 to 5 NM	5 to 12 NM	12 to 16 NM	16 to 24 NM	24 to 200 NM	> 200 NM	Total
> -10m	134 [100%, 13%, 2%]	0 [0%, 0%, 0%]	0 [0%, 0%, 0%]	0 [0%, 0%, 0%]	0 [0%, 0%, 0%]	0 [0%, 0%, 0%]	134 [2% of Total]
-10m to -50m	238 [100%, 24%, 4%]	1 [0%, 0%, 0%]	0 [0%, 0%, 0%]	0 [0%, 0%, 0%]	0 [0%, 0%, 0%]	0 [0%, 0%, 0%]	239 [4% of Total]
-50m to -100m	473 [37%, 48%, 7%]	737 [57%, 60%, 12%]	82 [6%, 13%, 1%]	0 [0%, 0%, 0%]	0 [0%, 0%, 0%]	0 [0%, 0%, 0%]	1,292 [20% of Total]
-100m to -150m	148 [13%, 15%, 2%]	370 [32%, 30%, 6%]	364 [32%, 58%, 6%]	266 [23%, 24%, 4%]	0 [0%, 0%, 0%]	0 [0%, 0%, 0%]	1,148 [18% of Total]
-150m to -200m	2 [0%, 0%, 0%]	84 [15%, 7%, 1%]	138 [25%, 22%, 2%]	323 [59%, 29%, 5%]	0.612 [0%, 0%, 0%]	0 [0%, 0%, 0%]	547 [9% of Total]
<-200m	0 [0%, 0%, 0%]	30 [1%, 2%, 0%]	42 [1%, 7%, 1%]	517 [17%, 47%, 8%]	2,406 [80%, 100%, 38%]	0 [0%, 0%, 0%]	2,995 [47% of Total]
Total	995 [16% of Total]	1,222 [19% of Total]	626 [10% of Total]	1,105 [17% of Total]	2,407 [38% of Total]	0.000 [0% of Total]	6,355 Km^2

The designations employed and the presentation of material in the map do not imply the expression of any opinion whatsoever on the part of FAO concerning the legal or constitutional status of any country, territory or sea area, or concerning the delimitation of frontiers.

Background reference map from National Geographic. Content may not reflect National Geographic's current map policy. Sources: National Geographic, Esri, DeLorme, HERE, UNEP-WCMC, USGS, NASA, ESA, METI, NRCAN, GEBCO, NOAA, increment P Corp.

Projection: Azimuthal Equidistant
Datum: WGS 1984
False Easting: 0.0000
False Northing: 0.0000
Central Meridian: 18.6958
Latitude Of Origin: 41.9781

Morocco: Mediterranean Region

Food and Agriculture Organization of the United Nations

©FAO, 2018

Kilometers: 0 125 250 375 500 625 750

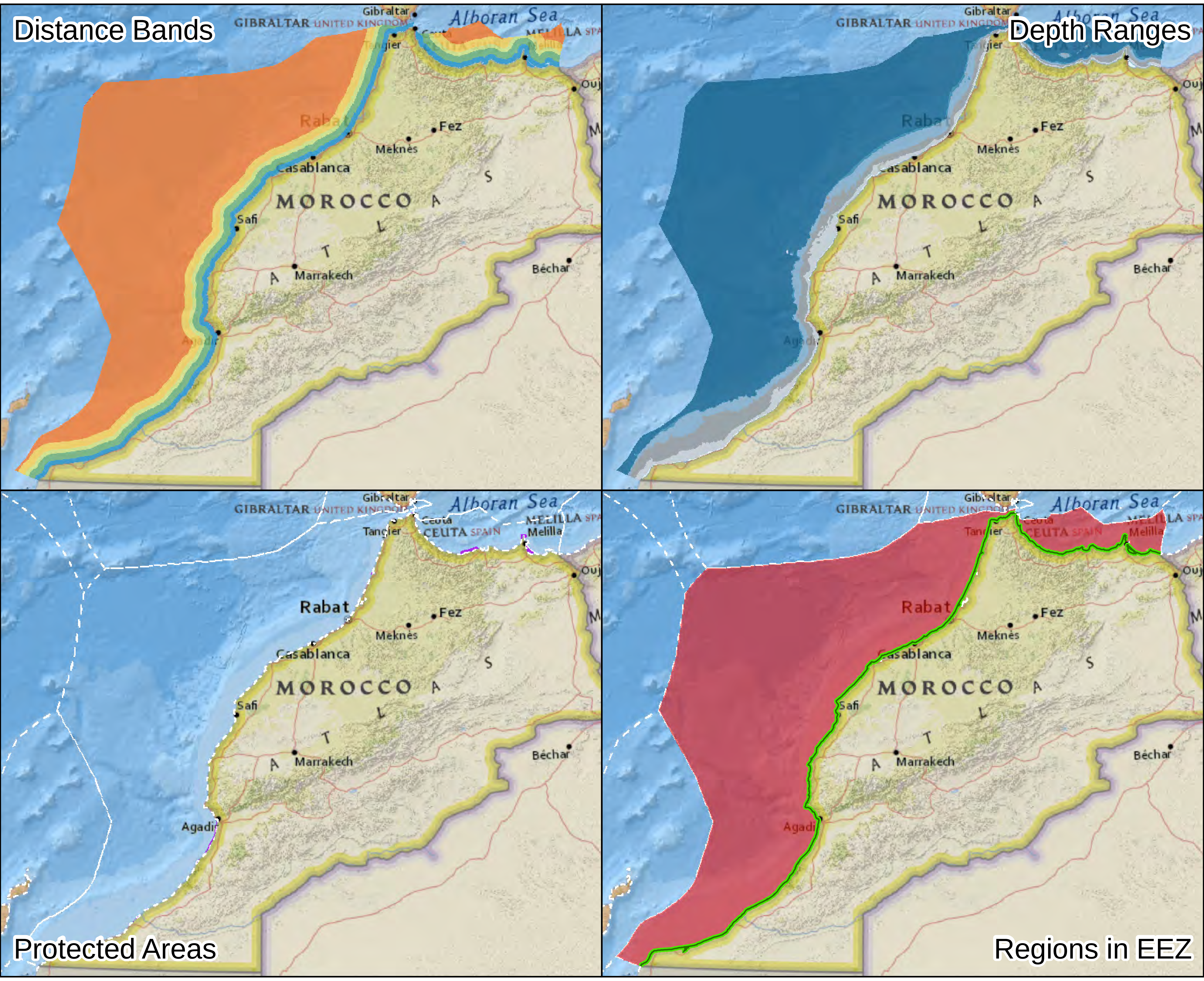

Distance Bands

- 0 to 5 NM
- 5 to 12 NM
- 12 to 16 NM
- 16 to 24 NM
- 24 to 200 NM
- > 200 NM

Depth Bands

- > -10m
- -10m to -50m
- -50m to -100m
- -100m to -150m
- -150m to -200m
- < -200m

Protected

- Protected Areas

Coastline Length

1,867 Km

Region

- Mediterranean

All Areas in Morocco EEZ: Cell Values = Area in Km^2 [% Depth Band (Row), % Distance Band (Column), % EEZ]

	0 to 5 NM	5 to 12 NM	12 to 16 NM	16 to 24 NM	24 to 200 NM	> 200 NM	Total
> -10m	1,138 [99%, 7%, 0%]	0 [0%, 0%, 0%]	0 [0%, 0%, 0%]	0 [0%, 0%, 0%]	10 [1%, 0%, 0%]	0 [0%, 0%, 0%]	1,148 [0% of Total]
-10m to -50m	8,408 [69%, 53%, 3%]	3,281 [27%, 15%, 1%]	385 [3%, 3%, 0%]	71 [1%, 0%, 0%]	7 [0%, 0%, 0%]	0 [0%, 0%, 0%]	12,151 [4% of Total]
-50m to -100m	4,964 [22%, 31%, 2%]	9,106 [39%, 43%, 3%]	3,675 [16%, 31%, 1%]	3,766 [16%, 17%, 1%]	1,552 [7%, 1%, 1%]	0 [0%, 0%, 0%]	23,063 [8% of Total]
-100m to -150m	515 [3%, 3%, 0%]	4,499 [24%, 21%, 2%]	3,907 [21%, 33%, 1%]	5,377 [29%, 24%, 2%]	4,178 [23%, 2%, 1%]	0 [0%, 0%, 0%]	18,476 [7% of Total]
-150m to -200m	322 [8%, 2%, 0%]	627 [15%, 3%, 0%]	746 [18%, 6%, 0%]	1,658 [40%, 7%, 1%]	843 [20%, 0%, 0%]	0 [0%, 0%, 0%]	4,196 [1% of Total]
<-200m	655 [0%, 4%, 0%]	3,825 [2%, 18%, 1%]	3,079 [1%, 26%, 1%]	11,380 [5%, 51%, 4%]	201,768 [91%, 97%, 72%]	5 [0%, 100%, 0%]	220,713 [79% of Total]
Total	16,001 [6% of Total]	21,338 [8% of Total]	11,792 [4% of Total]	22,252 [8% of Total]	208,358 [74% of Total]	5 [0% of Total]	279,746 Km^2

Areas in Morocco EEZ Excluding Protected Areas: Cell Values = Area in Km^2 [% Depth Band (Row), % Distance Band (Column), % EEZ]

586 [0%] Km^2 Protected	0 to 5 NM	5 to 12 NM	12 to 16 NM	16 to 24 NM	24 to 200 NM	> 200 NM	Total
> -10m	916 [99%, 6%, 0%]	0 [0%, 0%, 0%]	0 [0%, 0%, 0%]	0 [0%, 0%, 0%]	10 [1%, 0%, 0%]	0 [0%, 0%, 0%]	926 [0% of Total]
-10m to -50m	8,222 [69%, 53%, 3%]	3,281 [27%, 15%, 1%]	385 [3%, 3%, 0%]	71 [1%, 0%, 0%]	7 [0%, 0%, 0%]	0 [0%, 0%, 0%]	11,965 [4% of Total]
-50m to -100m	4,862 [21%, 32%, 2%]	9,106 [40%, 43%, 3%]	3,675 [16%, 31%, 1%]	3,766 [16%, 17%, 1%]	1,552 [7%, 1%, 1%]	0 [0%, 0%, 0%]	22,961 [8% of Total]
-100m to -150m	464 [3%, 3%, 0%]	4,499 [24%, 21%, 2%]	3,907 [21%, 33%, 1%]	5,377 [29%, 24%, 2%]	4,178 [23%, 2%, 1%]	0 [0%, 0%, 0%]	18,425 [7% of Total]
-150m to -200m	298 [7%, 2%, 0%]	627 [15%, 3%, 0%]	746 [18%, 6%, 0%]	1,658 [40%, 7%, 1%]	843 [20%, 0%, 0%]	0 [0%, 0%, 0%]	4,172 [1% of Total]
<-200m	653 [0%, 4%, 0%]	3,825 [2%, 18%, 1%]	3,079 [1%, 26%, 1%]	11,380 [5%, 51%, 4%]	201,768 [91%, 97%, 72%]	5 [0%, 100%, 0%]	220,711 [79% of Total]
Total	15,415 [6% of Total]	21,338 [8% of Total]	11,792 [4% of Total]	22,252 [8% of Total]	208,358 [75% of Total]	5 [0% of Total]	279,160 Km^2

The designations employed and the presentation of material in the map do not imply the expression of any opinion whatsoever on the part of FAO concerning the legal or constitutional status of any country, territory or sea area, or concerning the delimitation of frontiers.

Background reference map from National Geographic. Content may not reflect National Geographic's current map policy. Sources: National Geographic, Esri, DeLorme, HERE, UNEP-WCMC, USGS, NASA, ESA, METI, NRCAN, GEBCO, NOAA, increment P Corp.

Projection: Azimuthal Equidistant
Datum: WGS 1984
False Easting: 0.0000
False Northing: 0.0000
Central Meridian: -7.8611
Latitude Of Origin: 31.8385

Netherlands: North Sea Region

Food and Agriculture Organization of the United Nations

©FAO, 2018

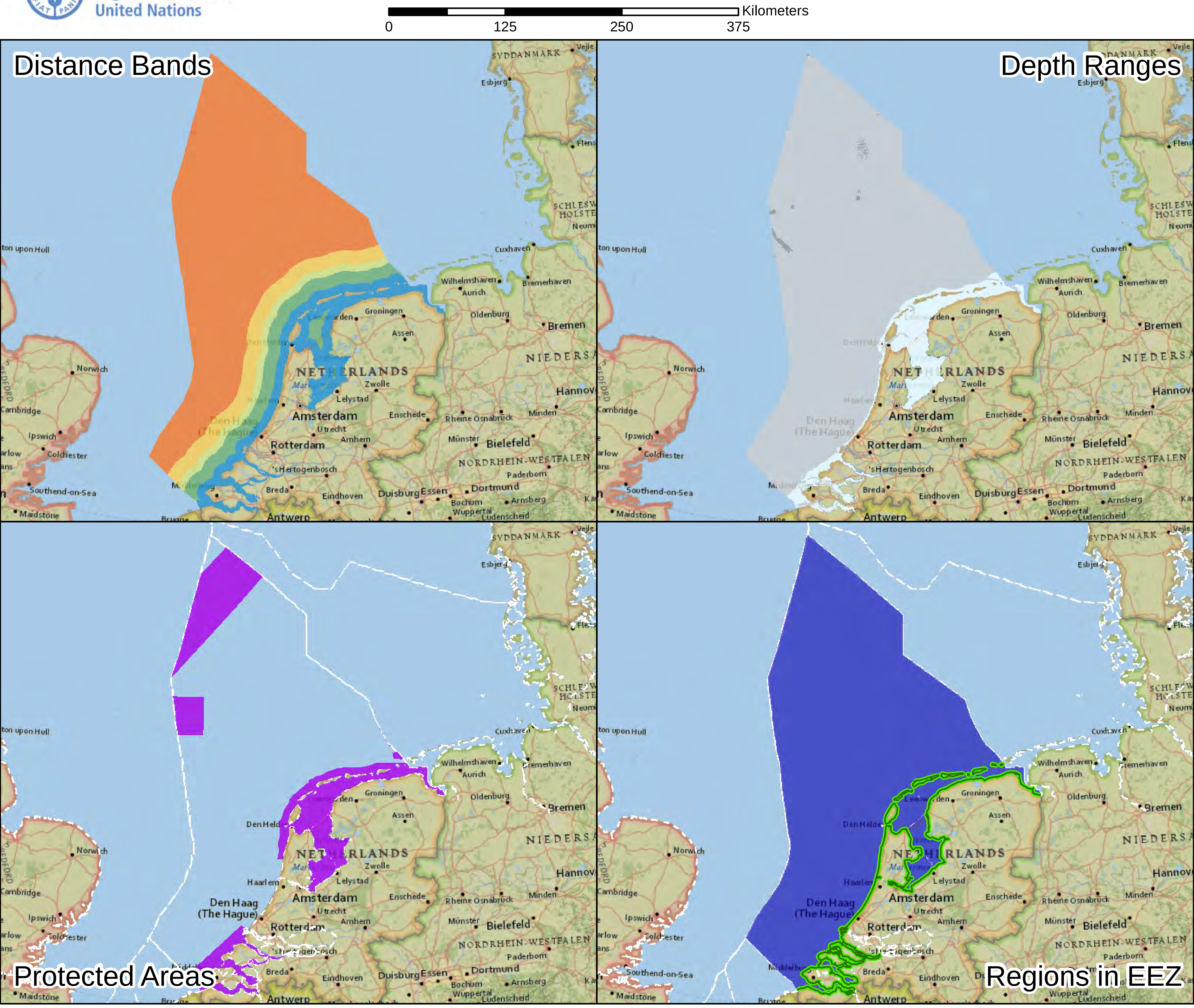

Distance Bands

- 0 to 5 NM
- 5 to 12 NM
- 12 to 16 NM
- 16 to 24 NM
- 24 to 200 NM

Depth Bands

- > -10m
- -10m to -50m
- -50m to -100m

Protected

- Protected Areas

Coastline Length

1,676 Km

Region

- Baltic

All Areas in Netherlands EEZ: Cell Values = Area in Km^2 [% Depth Band (Row), % Distance Band (Column), % EEZ]

	0 to 5 NM	5 to 12 NM	12 to 16 NM	16 to 24 NM	24 to 200 NM	> 200 NM	Total
> -10m	5,930 [92%, 73%, 9%]	540 [8%, 10%, 1%]	0 [0%, 0%, 0%]	0 [0%, 0%, 0%]	0 [0%, 0%, 0%]	0 [0%, 0%, 0%]	6,470 [10% of Total]
-10m to -50m	2,212 [4%, 27%, 3%]	4,695 [8%, 90%, 7%]	2,697 [5%, 100%, 4%]	5,409 [9%, 100%, 8%]	41,982 [74%, 99%, 66%]	0 [0%, 0%, 0%]	56,994 [89% of Total]
-50m to -100m	0 [0%, 0%, 0%]	0 [0%, 0%, 0%]	0 [0%, 0%, 0%]	0 [0%, 0%, 0%]	309 [100%, 1%, 0%]	0 [0%, 0%, 0%]	309 [0% of Total]
-100m to -150m	0 [0%, 0%, 0%]	0 [0%, 0%, 0%]	0 [0%, 0%, 0%]	0 [0%, 0%, 0%]	0 [0%, 0%, 0%]	0 [0%, 0%, 0%]	0.000 [0% of Total]
-150m to -200m	0 [0%, 0%, 0%]	0 [0%, 0%, 0%]	0 [0%, 0%, 0%]	0 [0%, 0%, 0%]	0 [0%, 0%, 0%]	0 [0%, 0%, 0%]	0.000 [0% of Total]
<-200m	0 [0%, 0%, 0%]	0 [0%, 0%, 0%]	0 [0%, 0%, 0%]	0 [0%, 0%, 0%]	0 [0%, 0%, 0%]	0 [0%, 0%, 0%]	0.000 [0% of Total]
Total	8,142 [13% of Total]	5,234 [8% of Total]	2,697 [4% of Total]	5,409 [8% of Total]	42,291 [66% of Total]	0.000 [0% of Total]	63,773 Km^2

Areas in Netherlands EEZ Excluding Protected Areas: Cell Values = Area in Km^2 [% Depth Band (Row), % Distance Band (Column), % EEZ]

13,599 [21%] Km^2 Protected	0 to 5 NM	5 to 12 NM	12 to 16 NM	16 to 24 NM	24 to 200 NM	> 200 NM	Total
> -10m	218 [100%, 18%, 0%]	0.272 [0%, 0%, 0%]	0 [0%, 0%, 0%]	0 [0%, 0%, 0%]	0 [0%, 0%, 0%]	0 [0%, 0%, 0%]	218 [0% of Total]
-10m to -50m	986 [2%, 82%, 2%]	4,471 [9%, 100%, 9%]	2,696 [5%, 100%, 5%]	5,407 [11%, 100%, 11%]	36,221 [73%, 100%, 72%]	0 [0%, 0%, 0%]	49,781 [99% of Total]
-50m to -100m	0 [0%, 0%, 0%]	0 [0%, 0%, 0%]	0 [0%, 0%, 0%]	0 [0%, 0%, 0%]	175 [100%, 0%, 0%]	0 [0%, 0%, 0%]	175 [0% of Total]
-100m to -150m	0 [0%, 0%, 0%]	0 [0%, 0%, 0%]	0 [0%, 0%, 0%]	0 [0%, 0%, 0%]	0 [0%, 0%, 0%]	0 [0%, 0%, 0%]	0.000 [0% of Total]
-150m to -200m	0 [0%, 0%, 0%]	0 [0%, 0%, 0%]	0 [0%, 0%, 0%]	0 [0%, 0%, 0%]	0 [0%, 0%, 0%]	0 [0%, 0%, 0%]	0.000 [0% of Total]
<-200m	0 [0%, 0%, 0%]	0 [0%, 0%, 0%]	0 [0%, 0%, 0%]	0 [0%, 0%, 0%]	0 [0%, 0%, 0%]	0 [0%, 0%, 0%]	0.000 [0% of Total]
Total	1,203 [2% of Total]	4,471 [9% of Total]	2,696 [5% of Total]	5,407 [11% of Total]	36,396 [73% of Total]	0.000 [0% of Total]	50,173 Km^2

The designations employed and the presentation of material in the map do not imply the expression of any opinion whatsoever on the part of FAO concerning the legal or constitutional status of any country, territory or sea area, or concerning the delimitation of frontiers.

Background reference map from National Geographic. Content may not reflect National Geographic's current map policy. Sources: National Geographic, Esri, DeLorme, HERE, UNEP-WCMC, USGS, NASA, ESA, METI, NRCAN, GEBCO, NOAA, increment P Corp.

Projection: Azimuthal Equidistant
Datum: WGS 1984
False Easting: 0.0000
False Northing: 0.0000
Central Meridian: 4.8748
Latitude Of Origin: 53.5526

Netherlands: Caribbean Region

©FAO, 2018

Food and Agriculture Organization of the United Nations

Kilometers: 0 125 250 375 500

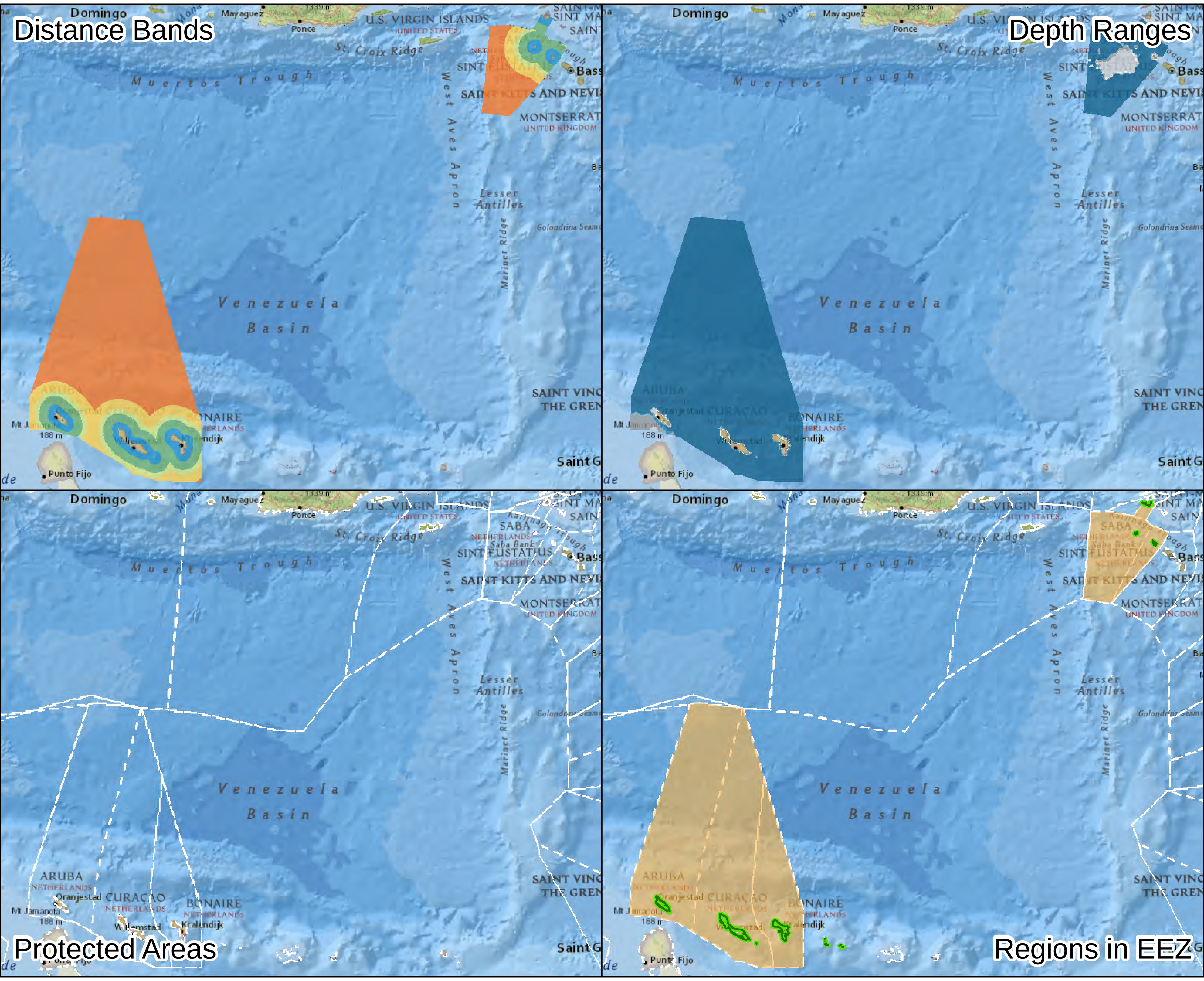

Distance Bands

- 0 to 5 NM
- 5 to 12 NM
- 12 to 16 NM
- 16 to 24 NM
- 24 to 200 NM

Depth Bands

- > -10m
- -10m to -50m
- -50m to -100m
- -100m to -150m
- -150m to -200m
- < -200m

Protected

- Protected Areas

Coastline Length

611 Km

Region

- Caribbean

All Areas in Netherlands EEZ: Cell Values = Area in Km² [% Depth Band (Row), % Distance Band (Column), % EEZ]

	0 to 5 NM	5 to 12 NM	12 to 16 NM	16 to 24 NM	24 to 200 NM	> 200 NM	Total
> -10m	177 [56%, 4%, 0%]	25 [8%, 0%, 0%]	5 [2%, 0%, 0%]	30 [9%, 0%, 0%]	78 [25%, 0%, 0%]	0 [0%, 0%, 0%]	315 [0% of Total]
-10m to -50m	308 [16%, 6%, 0%]	373 [19%, 4%, 0%]	284 [15%, 6%, 0%]	518 [27%, 6%, 1%]	442 [23%, 1%, 1%]	0 [0%, 0%, 0%]	1,925 [2% of Total]
-50m to -100m	288 [22%, 6%, 0%]	383 [29%, 4%, 0%]	215 [17%, 4%, 0%]	312 [24%, 4%, 0%]	104 [8%, 0%, 0%]	0 [0%, 0%, 0%]	1,302 [2% of Total]
-100m to -150m	216 [47%, 4%, 0%]	107 [23%, 1%, 0%]	33 [7%, 1%, 0%]	45 [10%, 1%, 0%]	57 [13%, 0%, 0%]	0 [0%, 0%, 0%]	457 [1% of Total]
-150m to -200m	332 [62%, 7%, 0%]	85 [16%, 1%, 0%]	30 [6%, 1%, 0%]	37 [7%, 0%, 0%]	51 [10%, 0%, 0%]	0 [0%, 0%, 0%]	536 [1% of Total]
<-200m	3,566 [5%, 73%, 4%]	8,661 [11%, 90%, 11%]	4,587 [6%, 89%, 6%]	7,741 [10%, 89%, 10%]	51,706 [68%, 99%, 64%]	0 [0%, 0%, 0%]	76,262 [94% of Total]
Total	4,887 [6% of Total]	9,633 [12% of Total]	5,154 [6% of Total]	8,683 [11% of Total]	52,439 [65% of Total]	0.000 [0% of Total]	80,796 Km²

Areas in Netherlands EEZ Excluding Protected Areas: Cell Values = Area in Km² [% Depth Band (Row), % Distance Band (Column), % EEZ]

29 [0%] Km² Protected	0 to 5 NM	5 to 12 NM	12 to 16 NM	16 to 24 NM	24 to 200 NM	> 200 NM	Total
> -10m	165 [54%, 3%, 0%]	25 [8%, 0%, 0%]	5 [2%, 0%, 0%]	30 [10%, 0%, 0%]	78 [26%, 0%, 0%]	0 [0%, 0%, 0%]	303 [0% of Total]
-10m to -50m	303 [16%, 6%, 0%]	373 [19%, 4%, 0%]	284 [15%, 6%, 0%]	518 [27%, 6%, 1%]	442 [23%, 1%, 1%]	0 [0%, 0%, 0%]	1,920 [2% of Total]
-50m to -100m	285 [22%, 6%, 0%]	383 [29%, 4%, 0%]	215 [17%, 4%, 0%]	312 [24%, 4%, 0%]	104 [8%, 0%, 0%]	0 [0%, 0%, 0%]	1,299 [2% of Total]
-100m to -150m	212 [47%, 4%, 0%]	107 [24%, 1%, 0%]	33 [7%, 1%, 0%]	45 [10%, 1%, 0%]	57 [13%, 0%, 0%]	0 [0%, 0%, 0%]	454 [1% of Total]
-150m to -200m	330 [62%, 7%, 0%]	85 [16%, 1%, 0%]	30 [6%, 1%, 0%]	37 [7%, 0%, 0%]	51 [10%, 0%, 0%]	0 [0%, 0%, 0%]	533 [1% of Total]
<-200m	3,564 [5%, 73%, 4%]	8,660 [11%, 90%, 11%]	4,587 [6%, 89%, 6%]	7,741 [10%, 89%, 10%]	51,706 [68%, 99%, 64%]	0 [0%, 0%, 0%]	76,259 [94% of Total]
Total	4,859 [6% of Total]	9,633 [12% of Total]	5,154 [6% of Total]	8,683 [11% of Total]	52,439 [65% of Total]	0.000 [0% of Total]	80,767 Km²

The designations employed and the presentation of material in the map do not imply the expression of any opinion whatsoever on the part of FAO concerning the legal or constitutional status of any country, territory or sea area, or concerning the delimitation of frontiers.

Background reference map from National Geographic. Content may not reflect National Geographic's current map policy. Sources: National Geographic, Esri, DeLorme, HERE, UNEP-WCMC, USGS, NASA, ESA, METI, NRCAN, GEBCO, NOAA, increment P Corp.

Projection: Azimuthal Equidistant
Datum: WGS 1984
False Easting: 0.0000
False Northing: 0.0000
Central Meridian: -66.5931
Latitude Of Origin: 14.8612

Nicaragua: Caribbean Region

©FAO, 2018

Food and Agriculture Organization of the United Nations

Kilometers: 0 125 250 375

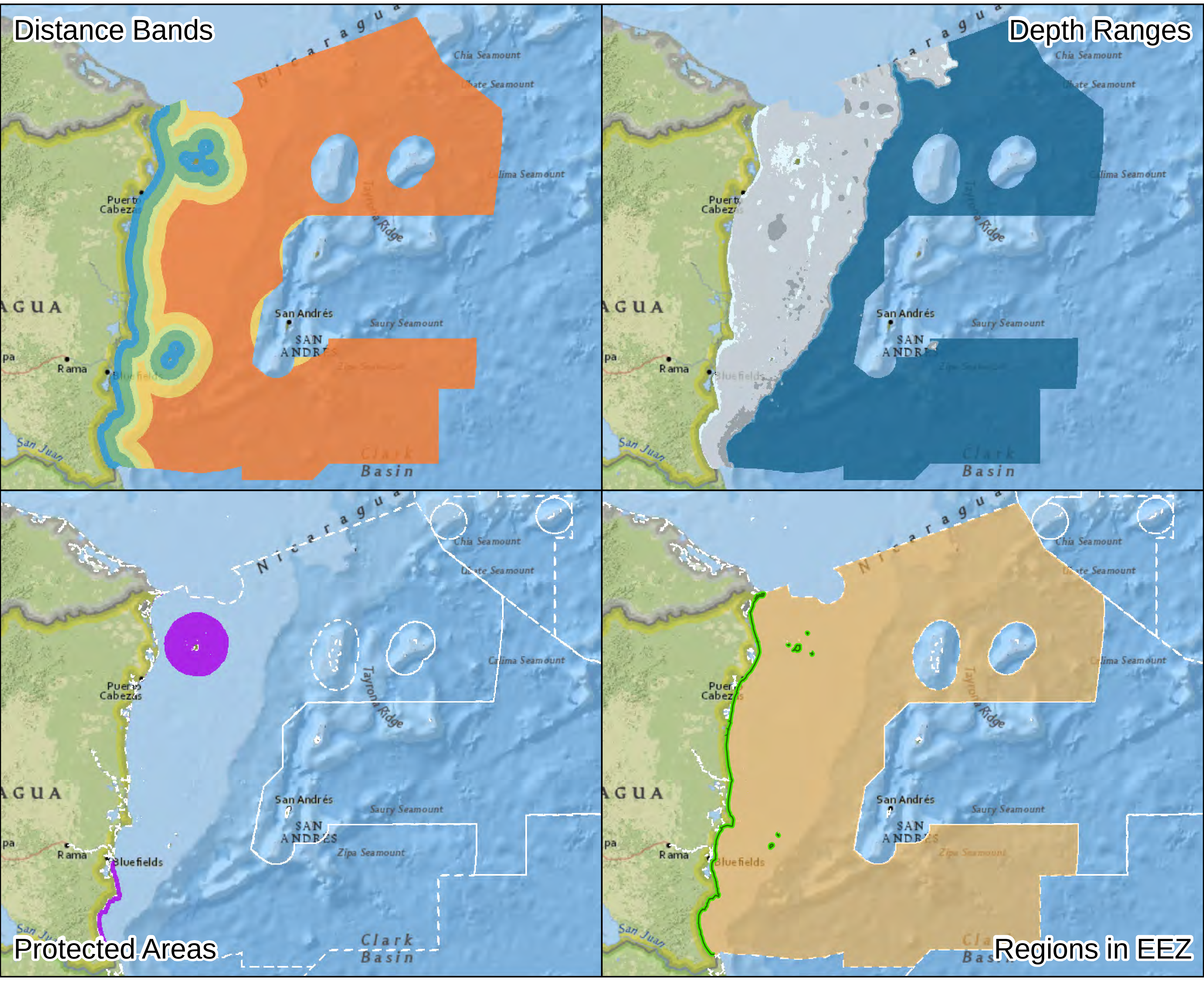

Distance Bands	Depth Bands	Protected	Region
0 to 5 NM	> -10m	Protected Areas	Caribbean
5 to 12 NM	-10m to -50m		
12 to 16 NM	-50m to -100m		
16 to 24 NM	-100m to -150m		
24 to 200 NM	-150m to -200m		
	< -200m		

Coastline Length: 630 Km

All Areas in Nicaragua EEZ: Cell Values = Area in Km^2 [% Depth Band (Row), % Distance Band (Column), % EEZ]

	0 to 5 NM	5 to 12 NM	12 to 16 NM	16 to 24 NM	24 to 200 NM	> 200 NM	Total
> -10m	2,412 [50%, 37%, 1%]	698 [14%, 6%, 0%]	147 [3%, 2%, 0%]	337 [7%, 3%, 0%]	1,225 [25%, 1%, 1%]	0 [0%, 0%, 0%]	4,819 [3% of Total]
-10m to -50m	4,101 [8%, 63%, 2%]	9,805 [20%, 90%, 6%]	5,190 [11%, 82%, 3%]	7,835 [16%, 66%, 5%]	21,464 [44%, 16%, 13%]	0 [0%, 0%, 0%]	48,395 [29% of Total]
-50m to -100m	30 [1%, 0%, 0%]	334 [8%, 3%, 0%]	487 [12%, 8%, 0%]	616 [15%, 5%, 0%]	2,652 [64%, 2%, 2%]	0 [0%, 0%, 0%]	4,118 [2% of Total]
-100m to -150m	0 [0%, 0%, 0%]	37 [4%, 0%, 0%]	58 [6%, 1%, 0%]	97 [10%, 1%, 0%]	817 [81%, 1%, 0%]	0 [0%, 0%, 0%]	1,010 [1% of Total]
-150m to -200m	0 [0%, 0%, 0%]	18 [2%, 0%, 0%]	79 [8%, 1%, 0%]	81 [8%, 1%, 0%]	843 [83%, 1%, 1%]	0 [0%, 0%, 0%]	1,020 [1% of Total]
<-200m	0 [0%, 0%, 0%]	47 [0%, 0%, 0%]	387 [0%, 6%, 0%]	2,872 [3%, 24%, 2%]	104,043 [97%, 79%, 62%]	0 [0%, 0%, 0%]	107,349 [64% of Total]
Total	6,543 [4% of Total]	10,939 [7% of Total]	6,348 [4% of Total]	11,838 [7% of Total]	131,044 [79% of Total]	0.000 [0% of Total]	166,712 Km^2

Areas in Nicaragua EEZ Excluding Protected Areas: Cell Values = Area in Km^2 [% Depth Band (Row), % Distance Band (Column), % EEZ]

5,721 [3%] Km^2 Protected	0 to 5 NM	5 to 12 NM	12 to 16 NM	16 to 24 NM	24 to 200 NM	> 200 NM	Total
> -10m	1,361 [43%, 31%, 1%]	155 [5%, 2%, 0%]	81 [3%, 1%, 0%]	337 [11%, 3%, 0%]	1,225 [39%, 1%, 1%]	0 [0%, 0%, 0%]	3,158 [2% of Total]
-10m to -50m	3,068 [7%, 69%, 2%]	7,586 [17%, 93%, 5%]	4,511 [10%, 81%, 3%]	7,709 [17%, 66%, 5%]	21,464 [48%, 16%, 13%]	0 [0%, 0%, 0%]	44,338 [28% of Total]
-50m to -100m	30 [1%, 1%, 0%]	334 [8%, 4%, 0%]	485 [12%, 9%, 0%]	616 [15%, 5%, 0%]	2,652 [64%, 2%, 2%]	0 [0%, 0%, 0%]	4,116 [3% of Total]
-100m to -150m	0 [0%, 0%, 0%]	37 [4%, 0%, 0%]	58 [6%, 1%, 0%]	97 [10%, 1%, 0%]	817 [81%, 1%, 1%]	0 [0%, 0%, 0%]	1,010 [1% of Total]
-150m to -200m	0 [0%, 0%, 0%]	18 [2%, 0%, 0%]	79 [8%, 1%, 0%]	81 [8%, 1%, 0%]	843 [83%, 1%, 1%]	0 [0%, 0%, 0%]	1,020 [1% of Total]
<-200m	0 [0%, 0%, 0%]	47 [0%, 1%, 0%]	387 [0%, 7%, 0%]	2,872 [3%, 25%, 2%]	104,043 [97%, 79%, 65%]	0 [0%, 0%, 0%]	107,349 [67% of Total]
Total	4,458 [3% of Total]	8,177 [5% of Total]	5,600 [3% of Total]	11,712 [7% of Total]	131,044 [81% of Total]	0.000 [0% of Total]	160,991 Km^2

The designations employed and the presentation of material in the map do not imply the expression of any opinion whatsoever on the part of FAO concerning the legal or constitutional status of any country, territory or sea area, or concerning the delimitation of frontiers.

Background reference map from National Geographic. Content may not reflect National Geographic's current map policy. Sources: National Geographic, Esri, DeLorme, HERE, UNEP-WCMC, USGS, NASA, ESA, METI, NRCAN, GEBCO, NOAA, increment P Corp.

Projection: Azimuthal Equidistant
Datum: WGS 1984
False Easting: 0.0000
False Northing: 0.0000
Central Meridian: -81.5571
Latitude Of Origin: 13.4127

Norway: North Sea and Norwegian Sea Regions

©FAO, 2018

Food and Agriculture Organization of the United Nations

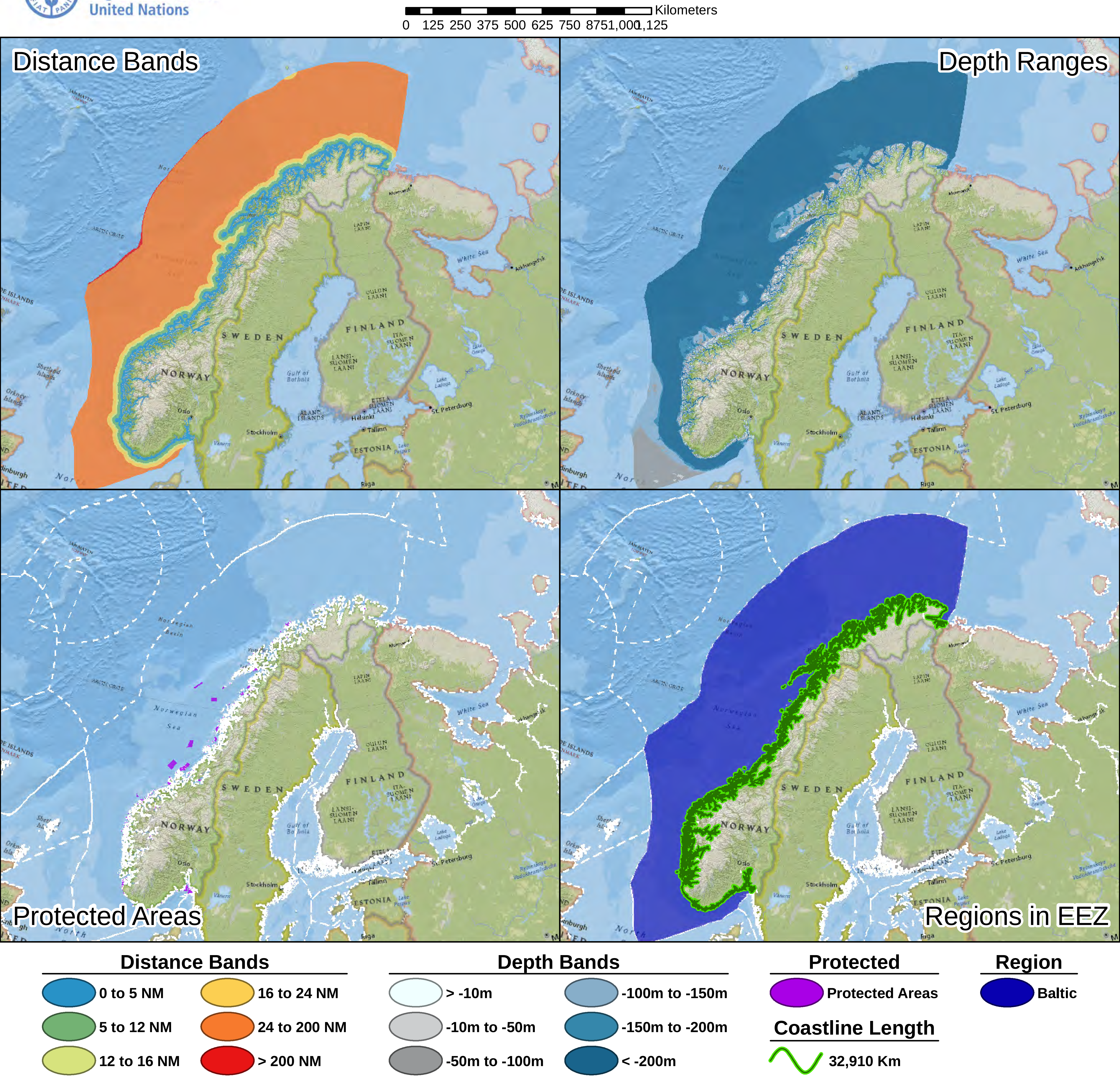

All Areas in Norway EEZ: Cell Values = Area in Km^2 [% Depth Band (Row), % Distance Band (Column), % EEZ]

	0 to 5 NM	5 to 12 NM	12 to 16 NM	16 to 24 NM	24 to 200 NM	> 200 NM	Total
> -10m	6,486 [98%, 9%, 1%]	141 [2%, 0%, 0%]	1 [0%, 0%, 0%]	0 [0%, 0%, 0%]	0 [0%, 0%, 0%]	0 [0%, 0%, 0%]	6,628 [1% of Total]
-10m to -50m	15,485 [87%, 21%, 2%]	1,497 [8%, 4%, 0%]	59 [0%, 0%, 0%]	51 [0%, 0%, 0%]	673 [4%, 0%, 0%]	0 [0%, 0%, 0%]	17,765 [2% of Total]
-50m to -100m	15,939 [23%, 22%, 2%]	4,739 [7%, 11%, 1%]	1,406 [2%, 6%, 0%]	1,233 [2%, 3%, 0%]	44,650 [66%, 6%, 5%]	0 [0%, 0%, 0%]	67,967 [7% of Total]
-100m to -150m	10,771 [20%, 15%, 1%]	5,780 [11%, 14%, 1%]	2,274 [4%, 10%, 0%]	3,850 [7%, 9%, 0%]	30,420 [57%, 4%, 3%]	0 [0%, 0%, 0%]	53,094 [6% of Total]
-150m to -200m	7,781 [19%, 11%, 1%]	6,084 [15%, 14%, 1%]	2,163 [5%, 10%, 0%]	5,371 [13%, 13%, 1%]	19,288 [47%, 3%, 2%]	0 [0%, 0%, 0%]	40,688 [4% of Total]
<-200m	16,635 [2%, 23%, 2%]	24,538 [3%, 57%, 3%]	16,751 [2%, 74%, 2%]	31,871 [4%, 75%, 3%]	642,589 [87%, 87%, 70%]	4,628 [1%, 100%, 1%]	737,013 [80% of Total]
Total	73,097 [8% of Total]	42,779 [5% of Total]	22,654 [2% of Total]	42,377 [5% of Total]	737,620 [80% of Total]	4,628 [1% of Total]	923,156 Km^2

Areas in Norway EEZ Excluding Protected Areas: Cell Values = Area in Km^2 [% Depth Band (Row), % Distance Band (Column), % EEZ]

6,598 [1%] Km^2 Protected	0 to 5 NM	5 to 12 NM	12 to 16 NM	16 to 24 NM	24 to 200 NM	> 200 NM	Total
> -10m	5,788 [99%, 8%, 1%]	63 [1%, 0%, 0%]	1 [0%, 0%, 0%]	0 [0%, 0%, 0%]	0 [0%, 0%, 0%]	0 [0%, 0%, 0%]	5,852 [1% of Total]
-10m to -50m	14,033 [88%, 20%, 2%]	1,190 [7%, 3%, 0%]	57 [0%, 0%, 0%]	51 [0%, 0%, 0%]	673 [4%, 0%, 0%]	0 [0%, 0%, 0%]	16,003 [2% of Total]
-50m to -100m	15,291 [23%, 22%, 2%]	4,487 [7%, 11%, 0%]	1,380 [2%, 6%, 0%]	1,233 [2%, 3%, 0%]	44,650 [67%, 6%, 5%]	0 [0%, 0%, 0%]	67,042 [7% of Total]
-100m to -150m	10,522 [20%, 15%, 1%]	5,663 [11%, 14%, 1%]	2,251 [4%, 10%, 0%]	3,845 [7%, 9%, 0%]	30,419 [58%, 4%, 3%]	0 [0%, 0%, 0%]	52,700 [6% of Total]
-150m to -200m	7,676 [19%, 11%, 1%]	6,038 [15%, 14%, 1%]	2,155 [5%, 10%, 0%]	5,367 [13%, 13%, 1%]	19,260 [48%, 3%, 2%]	0 [0%, 0%, 0%]	40,496 [4% of Total]
<-200m	16,497 [2%, 24%, 2%]	24,508 [3%, 58%, 3%]	16,734 [2%, 74%, 2%]	31,411 [4%, 75%, 3%]	640,687 [87%, 87%, 70%]	4,628 [1%, 100%, 1%]	734,465 [80% of Total]
Total	69,808 [8% of Total]	41,947 [5% of Total]	22,578 [2% of Total]	41,908 [5% of Total]	735,689 [80% of Total]	4,628 [1% of Total]	916,558 Km^2

The designations employed and the presentation of material in the map do not imply the expression of any opinion whatsoever on the part of FAO concerning the legal or constitutional status of any country, territory or sea area, or concerning the delimitation of frontiers.

Background reference map from National Geographic. Content may not reflect National Geographic's current map policy. Sources: National Geographic, Esri, DeLorme, HERE, UNEP-WCMC, USGS, NASA, ESA, METI, NRCAN, GEBCO, NOAA, increment P Corp.

Projection: Azimuthal Equidistant
Datum: WGS 1984
False Easting: 0.0000
False Northing: 0.0000
Central Meridian: 18.0262
Latitude Of Origin: 65.2957

Food and Agriculture Organization of the United Nations

Palestine: Mediterranean Region

©FAO, 2018

Kilometers
0 60

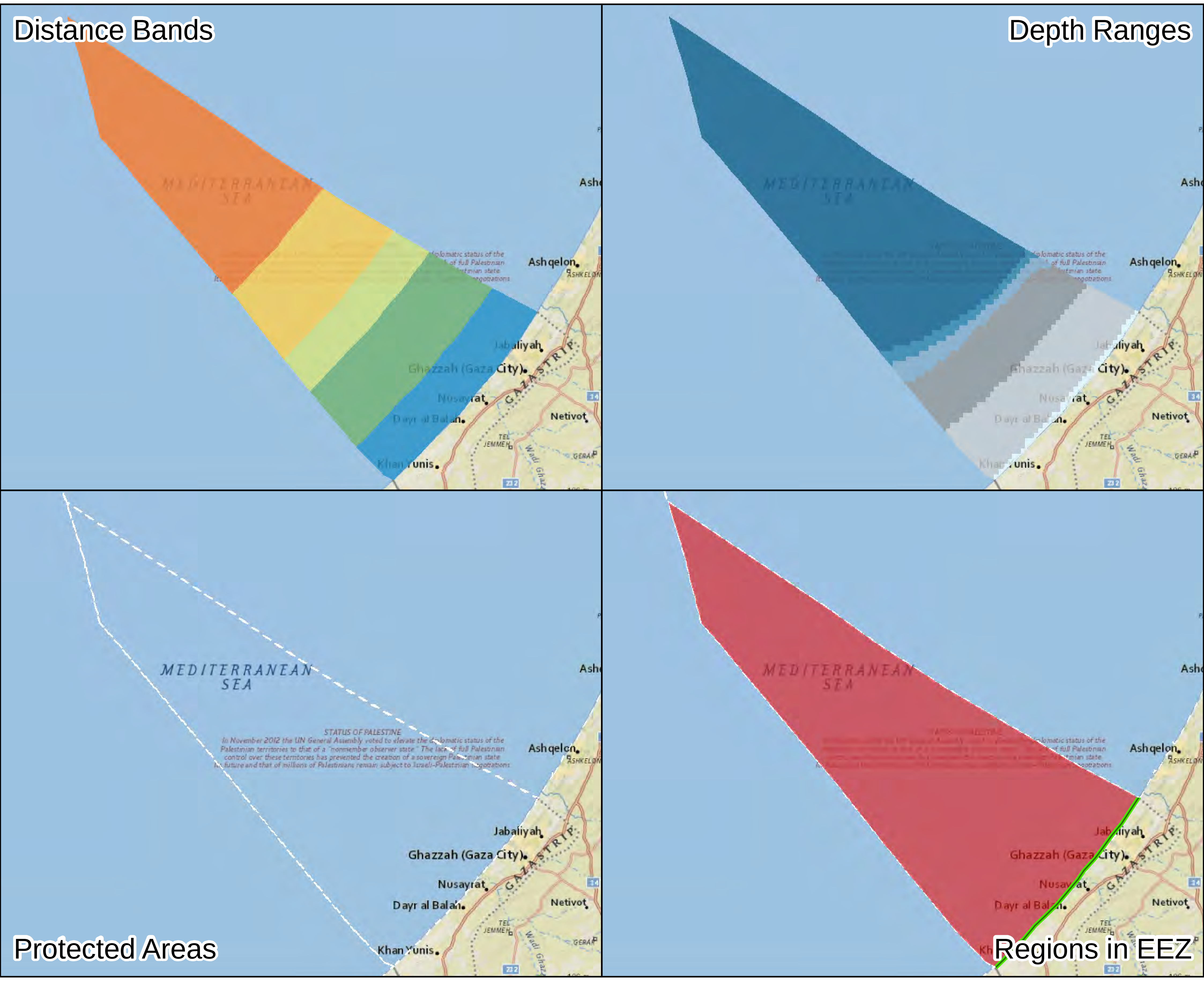

Distance Bands: 0 to 5 NM; 5 to 12 NM; 12 to 16 NM; 16 to 24 NM; 24 to 200 NM

Depth Bands: > -10m; -10m to -50m; -50m to -100m; -100m to -150m; -150m to -200m; < -200m

Protected: Protected Areas

Region: Mediterranean

Coastline Length: 40 Km

All Areas in Palestine EEZ: Cell Values = Area in Km^2 [% Depth Band (Row), % Distance Band (Column), % EEZ]

	0 to 5 NM	5 to 12 NM	12 to 16 NM	16 to 24 NM	24 to 200 NM	> 200 NM	Total
> -10m	31 [100%, 9%, 1%]	0 [0%, 0%, 0%]	0 [0%, 0%, 0%]	0 [0%, 0%, 0%]	0 [0%, 0%, 0%]	0 [0%, 0%, 0%]	31 [1% of Total]
-10m to -50m	326 [80%, 91%, 14%]	82 [20%, 18%, 4%]	0 [0%, 0%, 0%]	0 [0%, 0%, 0%]	0 [0%, 0%, 0%]	0 [0%, 0%, 0%]	408 [18% of Total]
-50m to -100m	0 [0%, 0%, 0%]	335 [99%, 73%, 14%]	5 [1%, 2%, 0%]	0 [0%, 0%, 0%]	0 [0%, 0%, 0%]	0 [0%, 0%, 0%]	339 [15% of Total]
-100m to -150m	0 [0%, 0%, 0%]	36 [43%, 8%, 2%]	48 [57%, 20%, 2%]	0 [0%, 0%, 0%]	0 [0%, 0%, 0%]	0 [0%, 0%, 0%]	84 [4% of Total]
-150m to -200m	0 [0%, 0%, 0%]	6 [11%, 1%, 0%]	49 [85%, 21%, 2%]	2 [4%, 1%, 0%]	0 [0%, 0%, 0%]	0 [0%, 0%, 0%]	57 [2% of Total]
<-200m	0 [0%, 0%, 0%]	0 [0%, 0%, 0%]	134 [9%, 57%, 6%]	408 [29%, 99%, 18%]	869 [62%, 100%, 37%]	0 [0%, 0%, 0%]	1,411 [61% of Total]
Total	358 [15% of Total]	459 [20% of Total]	235 [10% of Total]	410 [18% of Total]	869 [37% of Total]	0.000 [0% of Total]	2,331 Km^2

Areas in Palestine EEZ Excluding Protected Areas: Cell Values = Area in Km^2 [% Depth Band (Row), % Distance Band (Column), % EEZ]

0 [0%] Km^2 Protected	0 to 5 NM	5 to 12 NM	12 to 16 NM	16 to 24 NM	24 to 200 NM	> 200 NM	Total
> -10m	31 [100%, 9%, 1%]	0 [0%, 0%, 0%]	0 [0%, 0%, 0%]	0 [0%, 0%, 0%]	0 [0%, 0%, 0%]	0 [0%, 0%, 0%]	31 [1% of Total]
-10m to -50m	326 [80%, 91%, 14%]	82 [20%, 18%, 4%]	0 [0%, 0%, 0%]	0 [0%, 0%, 0%]	0 [0%, 0%, 0%]	0 [0%, 0%, 0%]	408 [18% of Total]
-50m to -100m	0 [0%, 0%, 0%]	335 [99%, 73%, 14%]	5 [1%, 2%, 0%]	0 [0%, 0%, 0%]	0 [0%, 0%, 0%]	0 [0%, 0%, 0%]	339 [15% of Total]
-100m to -150m	0 [0%, 0%, 0%]	36 [43%, 8%, 2%]	48 [57%, 20%, 2%]	0 [0%, 0%, 0%]	0 [0%, 0%, 0%]	0 [0%, 0%, 0%]	84 [4% of Total]
-150m to -200m	0 [0%, 0%, 0%]	6 [11%, 1%, 0%]	49 [85%, 21%, 2%]	2 [4%, 1%, 0%]	0 [0%, 0%, 0%]	0 [0%, 0%, 0%]	57 [2% of Total]
<-200m	0 [0%, 0%, 0%]	0 [0%, 0%, 0%]	134 [9%, 57%, 6%]	408 [29%, 99%, 18%]	869 [62%, 100%, 37%]	0 [0%, 0%, 0%]	1,411 [61% of Total]
Total	358 [15% of Total]	459 [20% of Total]	235 [10% of Total]	410 [18% of Total]	869 [37% of Total]	0.000 [0% of Total]	2,331 Km^2

The designations employed and the presentation of material in the map do not imply the expression of any opinion whatsoever on the part of FAO concerning the legal or constitutional status of any country, territory or sea area, or concerning the delimitation of frontiers.

Background reference map from National Geographic. Content may not reflect National Geographic's current map policy. Sources: National Geographic, Esri, DeLorme, HERE, UNEP-WCMC, USGS, NASA, ESA, METI, NRCAN, GEBCO, NOAA, increment P Corp.

Projection: Azimuthal Equidistant
Datum: WGS 1984
False Easting: 0.0000
False Northing: 0.0000
Central Meridian: 34.0439
Latitude Of Origin: 31.7000

N W E S

Panama: Caribbean Region

Food and Agriculture Organization of the United Nations

©FAO, 2018

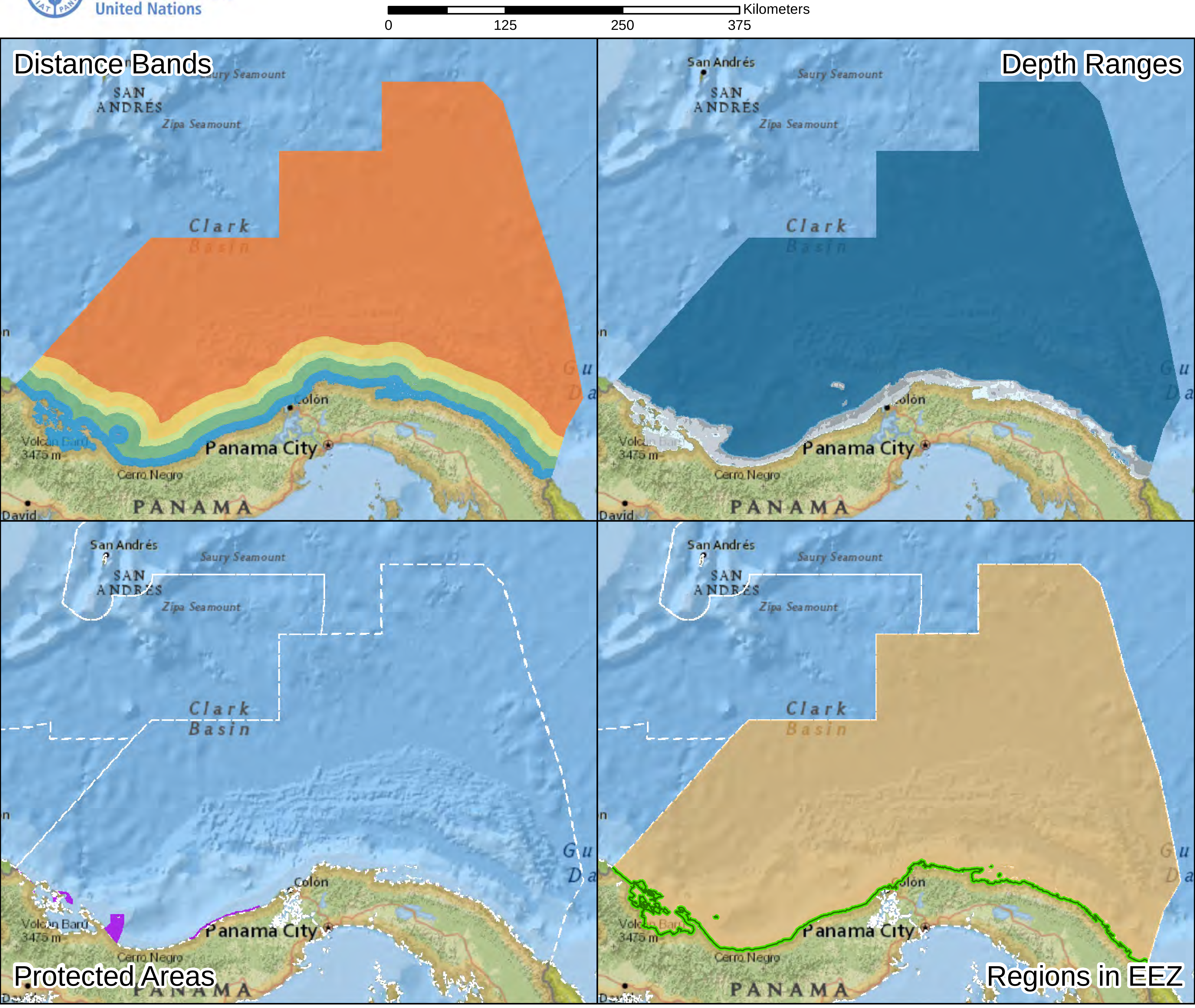

Distance Bands

- 0 to 5 NM
- 5 to 12 NM
- 12 to 16 NM
- 16 to 24 NM
- 24 to 200 NM

Depth Bands

- > -10m
- -10m to -50m
- -50m to -100m
- -100m to -150m
- -150m to -200m
- < -200m

Protected

- Protected Areas

Coastline Length

- 1,356 Km

Region

- Caribbean

All Areas in Panama EEZ: Cell Values = Area in Km^2 [% Depth Band (Row), % Distance Band (Column), % EEZ]

	0 to 5 NM	5 to 12 NM	12 to 16 NM	16 to 24 NM	24 to 200 NM	> 200 NM	Total
> -10m	1,862 [94%, 22%, 1%]	109 [6%, 1%, 0%]	0 [0%, 0%, 0%]	0 [0%, 0%, 0%]	0 [0%, 0%, 0%]	0 [0%, 0%, 0%]	1,971 [1% of Total]
-10m to -50m	4,733 [88%, 55%, 3%]	615 [11%, 7%, 0%]	0 [0%, 0%, 0%]	7 [0%, 0%, 0%]	21 [0%, 0%, 0%]	0 [0%, 0%, 0%]	5,376 [4% of Total]
-50m to -100m	1,283 [51%, 15%, 1%]	1,206 [48%, 13%, 1%]	10 [0%, 0%, 0%]	13 [1%, 0%, 0%]	25 [1%, 0%, 0%]	0 [0%, 0%, 0%]	2,537 [2% of Total]
-100m to -150m	312 [40%, 4%, 0%]	429 [56%, 5%, 0%]	10 [1%, 0%, 0%]	11 [1%, 0%, 0%]	10 [1%, 0%, 0%]	0 [0%, 0%, 0%]	772 [1% of Total]
-150m to -200m	134 [22%, 2%, 0%]	444 [73%, 5%, 0%]	15 [2%, 0%, 0%]	5 [1%, 0%, 0%]	13 [2%, 0%, 0%]	0 [0%, 0%, 0%]	611 [0% of Total]
<-200m	261 [0%, 3%, 0%]	6,384 [5%, 69%, 4%]	4,964 [4%, 99%, 3%]	9,702 [7%, 100%, 7%]	109,961 [84%, 100%, 77%]	0 [0%, 0%, 0%]	131,273 [92% of Total]
Total	8,586 [6% of Total]	9,186 [6% of Total]	5,000 [4% of Total]	9,739 [7% of Total]	110,029 [77% of Total]	0.000 [0% of Total]	142,540 Km^2

Areas in Panama EEZ Excluding Protected Areas: Cell Values = Area in Km^2 [% Depth Band (Row), % Distance Band (Column), % EEZ]

678 [0%] Km^2 Protected	0 to 5 NM	5 to 12 NM	12 to 16 NM	16 to 24 NM	24 to 200 NM	> 200 NM	Total
> -10m	1,706 [94%, 21%, 1%]	109 [6%, 1%, 0%]	0 [0%, 0%, 0%]	0 [0%, 0%, 0%]	0 [0%, 0%, 0%]	0 [0%, 0%, 0%]	1,814 [1% of Total]
-10m to -50m	4,280 [88%, 54%, 3%]	581 [12%, 6%, 0%]	0 [0%, 0%, 0%]	7 [0%, 0%, 0%]	21 [0%, 0%, 0%]	0 [0%, 0%, 0%]	4,888 [3% of Total]
-50m to -100m	1,265 [50%, 16%, 1%]	1,193 [48%, 13%, 1%]	10 [0%, 0%, 0%]	13 [1%, 0%, 0%]	25 [1%, 0%, 0%]	0 [0%, 0%, 0%]	2,506 [2% of Total]
-100m to -150m	310 [40%, 4%, 0%]	428 [56%, 5%, 0%]	10 [1%, 0%, 0%]	11 [1%, 0%, 0%]	10 [1%, 0%, 0%]	0 [0%, 0%, 0%]	769 [1% of Total]
-150m to -200m	134 [22%, 2%, 0%]	444 [73%, 5%, 0%]	15 [2%, 0%, 0%]	5 [1%, 0%, 0%]	13 [2%, 0%, 0%]	0 [0%, 0%, 0%]	611 [0% of Total]
<-200m	260 [0%, 3%, 0%]	6,384 [5%, 70%, 5%]	4,964 [4%, 99%, 3%]	9,702 [7%, 100%, 7%]	109,961 [84%, 100%, 78%]	0 [0%, 0%, 0%]	131,272 [93% of Total]
Total	7,954 [6% of Total]	9,139 [6% of Total]	5,000 [4% of Total]	9,739 [7% of Total]	110,029 [78% of Total]	0.000 [0% of Total]	141,862 Km^2

The designations employed and the presentation of material in the map do not imply the expression of any opinion whatsoever on the part of FAO concerning the legal or constitutional status of any country, territory or sea area, or concerning the delimitation of frontiers.

Background reference map from National Geographic. Content may not reflect National Geographic's current map policy. Sources: National Geographic, Esri, DeLorme, HERE, UNEP-WCMC, USGS, NASA, ESA, METI, NRCAN, GEBCO, NOAA, increment P Corp.

Projection: Azimuthal Equidistant
Datum: WGS 1984
False Easting: 0.0000
False Northing: 0.0000
Central Meridian: -79.8078
Latitude Of Origin: 10.5820

Food and Agriculture Organization of the United Nations

Poland: Baltic Region

©FAO, 2018

Kilometers
0 125 250

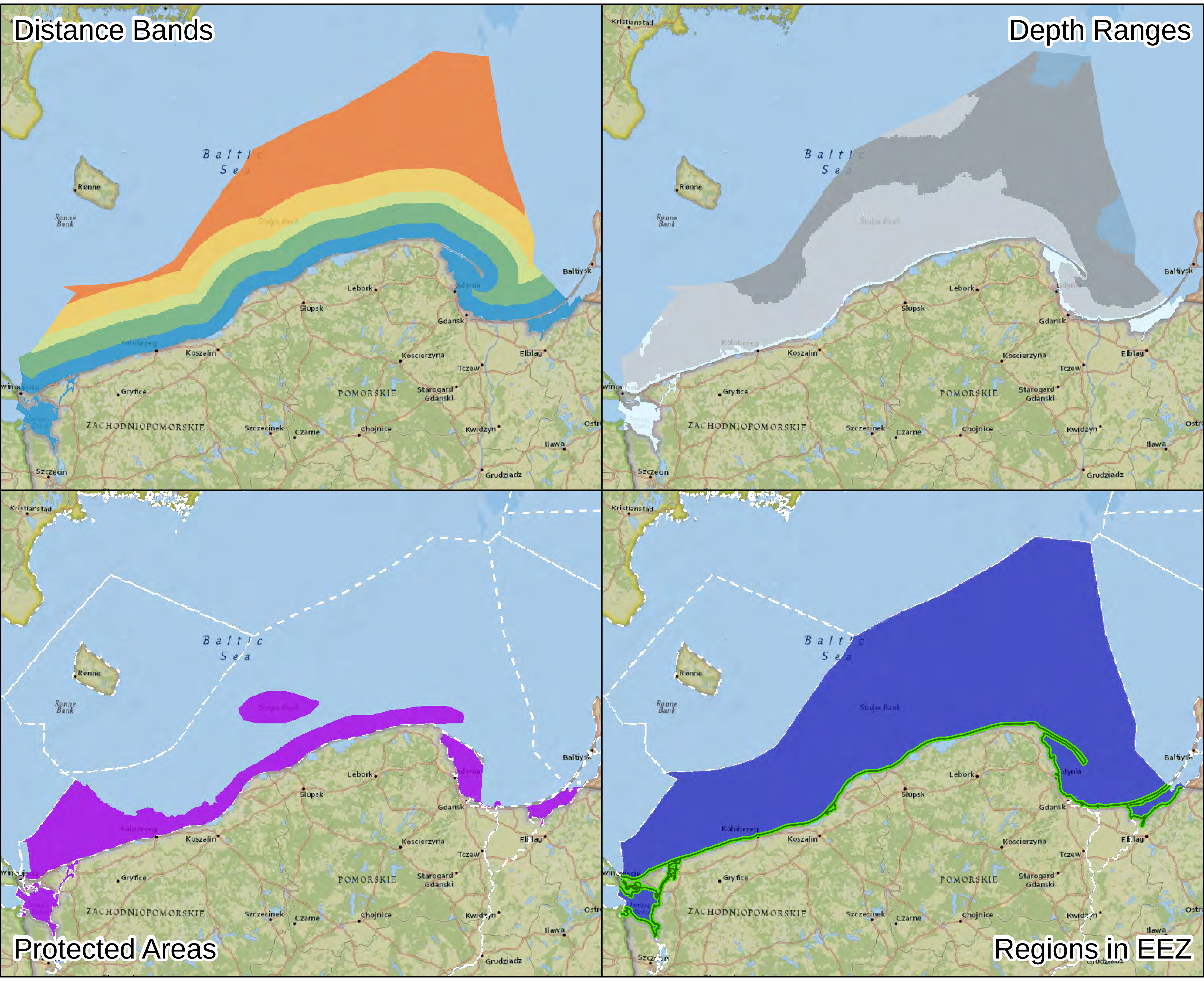

Distance Bands
- 0 to 5 NM
- 5 to 12 NM
- 12 to 16 NM
- 16 to 24 NM
- 24 to 200 NM

Depth Bands
- > -10m
- -10m to -50m
- -50m to -100m
- -100m to -150m

Protected
- Protected Areas

Coastline Length
- 883 Km

Region
- Baltic

All Areas in Poland EEZ: Cell Values = Area in Km² [% Depth Band (Row), % Distance Band (Column), % EEZ]

	0 to 5 NM	5 to 12 NM	12 to 16 NM	16 to 24 NM	24 to 200 NM	> 200 NM	Total
> -10m	1,460 [95%, 29%, 5%]	8 [1%, 0%, 0%]	35 [2%, 1%, 0%]	27 [2%, 1%, 0%]	2 [0%, 0%, 0%]	0 [0%, 0%, 0%]	1,532 [5% of Total]
-10m to -50m	3,257 [24%, 64%, 11%]	4,109 [30%, 77%, 14%]	1,886 [14%, 68%, 6%]	2,791 [20%, 54%, 9%]	1,575 [12%, 14%, 5%]	0 [0%, 0%, 0%]	13,617 [46% of Total]
-50m to -100m	337 [3%, 7%, 1%]	1,222 [9%, 23%, 4%]	728 [6%, 26%, 2%]	1,905 [14%, 37%, 6%]	8,972 [68%, 80%, 30%]	0 [0%, 0%, 0%]	13,164 [45% of Total]
-100m to -150m	0 [0%, 0%, 0%]	0.394 [0%, 0%, 0%]	130 [11%, 5%, 0%]	425 [35%, 8%, 1%]	659 [54%, 6%, 2%]	0 [0%, 0%, 0%]	1,214 [4% of Total]
-150m to -200m	0 [0%, 0%, 0%]	0 [0%, 0%, 0%]	0 [0%, 0%, 0%]	0 [0%, 0%, 0%]	0 [0%, 0%, 0%]	0 [0%, 0%, 0%]	0.000 [0% of Total]
<-200m	0 [0%, 0%, 0%]	0 [0%, 0%, 0%]	0 [0%, 0%, 0%]	0 [0%, 0%, 0%]	0 [0%, 0%, 0%]	0 [0%, 0%, 0%]	0.000 [0% of Total]
Total	5,054 [17% of Total]	5,339 [18% of Total]	2,778 [9% of Total]	5,148 [17% of Total]	11,207 [38% of Total]	0.000 [0% of Total]	29,526 Km²

Areas in Poland EEZ Excluding Protected Areas: Cell Values = Area in Km² [% Depth Band (Row), % Distance Band (Column), % EEZ]

7,130 [24%] Km² Protected	0 to 5 NM	5 to 12 NM	12 to 16 NM	16 to 24 NM	24 to 200 NM	> 200 NM	Total
> -10m	97 [100%, 9%, 0%]	0 [0%, 0%, 0%]	0 [0%, 0%, 0%]	0 [0%, 0%, 0%]	0 [0%, 0%, 0%]	0 [0%, 0%, 0%]	97 [0% of Total]
-10m to -50m	680 [9%, 63%, 3%]	2,558 [32%, 68%, 11%]	1,355 [17%, 61%, 6%]	1,895 [24%, 45%, 8%]	1,464 [18%, 13%, 7%]	0 [0%, 0%, 0%]	7,952 [36% of Total]
-50m to -100m	307 [2%, 28%, 1%]	1,222 [9%, 32%, 5%]	728 [6%, 33%, 3%]	1,905 [15%, 45%, 9%]	8,972 [68%, 81%, 40%]	0 [0%, 0%, 0%]	13,133 [59% of Total]
-100m to -150m	0 [0%, 0%, 0%]	0.394 [0%, 0%, 0%]	130 [11%, 6%, 1%]	425 [35%, 10%, 2%]	659 [54%, 6%, 3%]	0 [0%, 0%, 0%]	1,214 [5% of Total]
-150m to -200m	0 [0%, 0%, 0%]	0 [0%, 0%, 0%]	0 [0%, 0%, 0%]	0 [0%, 0%, 0%]	0 [0%, 0%, 0%]	0 [0%, 0%, 0%]	0.000 [0% of Total]
<-200m	0 [0%, 0%, 0%]	0 [0%, 0%, 0%]	0 [0%, 0%, 0%]	0 [0%, 0%, 0%]	0 [0%, 0%, 0%]	0 [0%, 0%, 0%]	0.000 [0% of Total]
Total	1,084 [5% of Total]	3,781 [17% of Total]	2,213 [10% of Total]	4,224 [19% of Total]	11,095 [50% of Total]	0.000 [0% of Total]	22,397 Km²

The designations employed and the presentation of material in the map do not imply the expression of any opinion whatsoever on the part of FAO concerning the legal or constitutional status of any country, territory or sea area, or concerning the delimitation of frontiers.

Background reference map from National Geographic. Content may not reflect National Geographic's current map policy. Sources: National Geographic, Esri, DeLorme, HERE, UNEP-WCMC, USGS, NASA, ESA, METI, NRCAN, GEBCO, NOAA, increment P Corp.

Projection: Azimuthal Equidistant
Datum: WGS 1984
False Easting: 0.0000
False Northing: 0.0000
Central Meridian: 16.9999
Latitude Of Origin: 54.7521

Portugal: Atlantic Region

©FAO, 2018

Food and Agriculture Organization of the United Nations

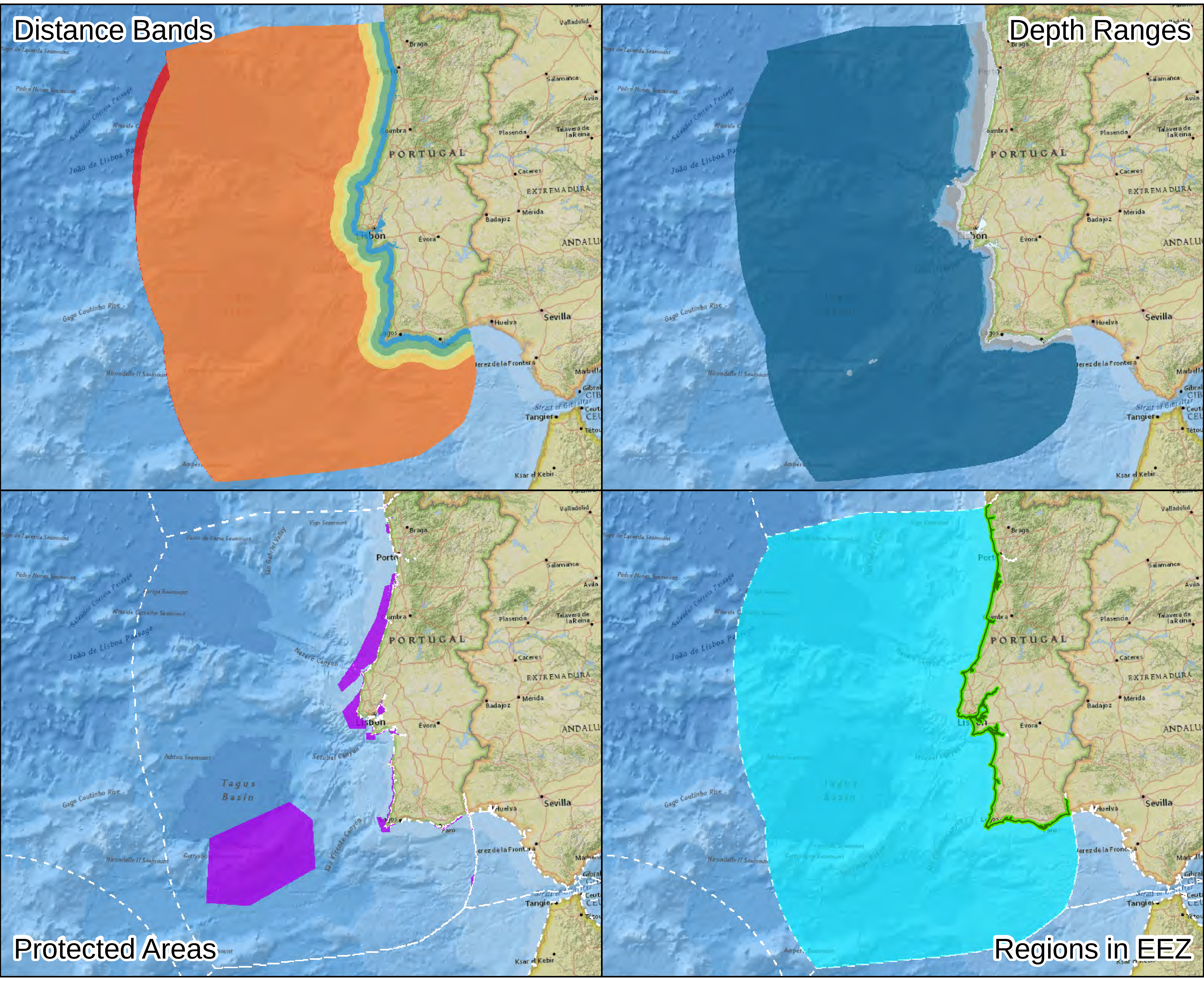

Distance Bands

- 0 to 5 NM
- 5 to 12 NM
- 12 to 16 NM
- 16 to 24 NM
- 24 to 200 NM
- > 200 NM

Depth Bands

- > -10m
- -10m to -50m
- -50m to -100m
- -100m to -150m
- -150m to -200m
- < -200m

Protected

- Protected Areas

Coastline Length

- 1,567 Km

Region

- Atlantic

All Areas in Portugal EEZ: Cell Values = Area in Km^2 [% Depth Band (Row), % Distance Band (Column), % EEZ]

	0 to 5 NM	5 to 12 NM	12 to 16 NM	16 to 24 NM	24 to 200 NM	> 200 NM	Total
> -10m	1,074 [99%, 14%, 0%]	4 [0%, 0%, 0%]	7 [1%, 0%, 0%]	0 [0%, 0%, 0%]	0 [0%, 0%, 0%]	0 [0%, 0%, 0%]	1,085 [0% of Total]
-10m to -50m	4,077 [84%, 52%, 1%]	791 [16%, 7%, 0%]	6 [0%, 0%, 0%]	0 [0%, 0%, 0%]	0 [0%, 0%, 0%]	0 [0%, 0%, 0%]	4,873 [2% of Total]
-50m to -100m	2,008 [26%, 26%, 1%]	4,055 [53%, 38%, 1%]	1,305 [17%, 22%, 0%]	236 [3%, 2%, 0%]	36 [0%, 0%, 0%]	0 [0%, 0%, 0%]	7,639 [2% of Total]
-100m to -150m	513 [6%, 7%, 0%]	2,627 [32%, 25%, 1%]	1,326 [16%, 22%, 0%]	3,007 [37%, 25%, 1%]	701 [9%, 0%, 0%]	0 [0%, 0%, 0%]	8,174 [3% of Total]
-150m to -200m	63 [2%, 1%, 0%]	664 [25%, 6%, 0%]	223 [8%, 4%, 0%]	723 [27%, 6%, 0%]	1,009 [38%, 0%, 0%]	0 [0%, 0%, 0%]	2,683 [1% of Total]
<-200m	123 [0%, 2%, 0%]	2,418 [1%, 23%, 1%]	3,152 [1%, 52%, 1%]	8,051 [3%, 67%, 3%]	273,126 [94%, 99%, 87%]	3,777 [1%, 100%, 1%]	290,647 [92% of Total]
Total	7,858 [2% of Total]	10,558 [3% of Total]	6,020 [2% of Total]	12,017 [4% of Total]	274,872 [87% of Total]	3,777 [1% of Total]	315,102 Km^2

Areas in Portugal EEZ Excluding Protected Areas: Cell Values = Area in Km^2 [% Depth Band (Row), % Distance Band (Column), % EEZ]

29,693 [9%] Km^2 Protected	0 to 5 NM	5 to 12 NM	12 to 16 NM	16 to 24 NM	24 to 200 NM	> 200 NM	Total
> -10m	409 [100%, 9%, 0%]	0 [0%, 0%, 0%]	0.696 [0%, 0%, 0%]	0 [0%, 0%, 0%]	0 [0%, 0%, 0%]	0 [0%, 0%, 0%]	410 [0% of Total]
-10m to -50m	2,400 [80%, 52%, 1%]	609 [20%, 8%, 0%]	3 [0%, 0%, 0%]	0 [0%, 0%, 0%]	0 [0%, 0%, 0%]	0 [0%, 0%, 0%]	3,011 [1% of Total]
-50m to -100m	1,281 [24%, 28%, 0%]	2,694 [51%, 34%, 1%]	1,123 [21%, 21%, 0%]	231 [4%, 2%, 0%]	0.669 [0%, 0%, 0%]	0 [0%, 0%, 0%]	5,330 [2% of Total]
-100m to -150m	404 [6%, 9%, 0%]	1,787 [27%, 23%, 1%]	1,051 [16%, 19%, 0%]	2,862 [43%, 24%, 1%]	616 [9%, 0%, 0%]	0 [0%, 0%, 0%]	6,720 [2% of Total]
-150m to -200m	39 [2%, 1%, 0%]	579 [23%, 7%, 0%]	188 [8%, 3%, 0%]	699 [28%, 6%, 0%]	964 [39%, 0%, 0%]	0 [0%, 0%, 0%]	2,468 [1% of Total]
<-200m	70 [0%, 2%, 0%]	2,151 [1%, 28%, 1%]	3,092 [1%, 57%, 1%]	8,051 [3%, 68%, 3%]	250,328 [94%, 99%, 88%]	3,777 [1%, 100%, 1%]	267,469 [94% of Total]
Total	4,603 [2% of Total]	7,820 [3% of Total]	5,457 [2% of Total]	11,843 [4% of Total]	251,909 [88% of Total]	3,777 [1% of Total]	285,408 Km^2

The designations employed and the presentation of material in the map do not imply the expression of any opinion whatsoever on the part of FAO concerning the legal or constitutional status of any country, territory or sea area, or concerning the delimitation of frontiers.

Background reference map from National Geographic. Content may not reflect National Geographic's current map policy. Sources: National Geographic, Esri, DeLorme, HERE, UNEP-WCMC, USGS, NASA, ESA, METI, NRCAN, GEBCO, NOAA, increment P Corp.

Projection: Azimuthal Equidistant
Datum: WGS 1984
False Easting: 0.0000
False Northing: 0.0000
Central Meridian: -10.5611
Latitude Of Origin: 38.3938

Romania: Black Sea Region

Food and Agriculture Organization of the United Nations

©FAO, 2018

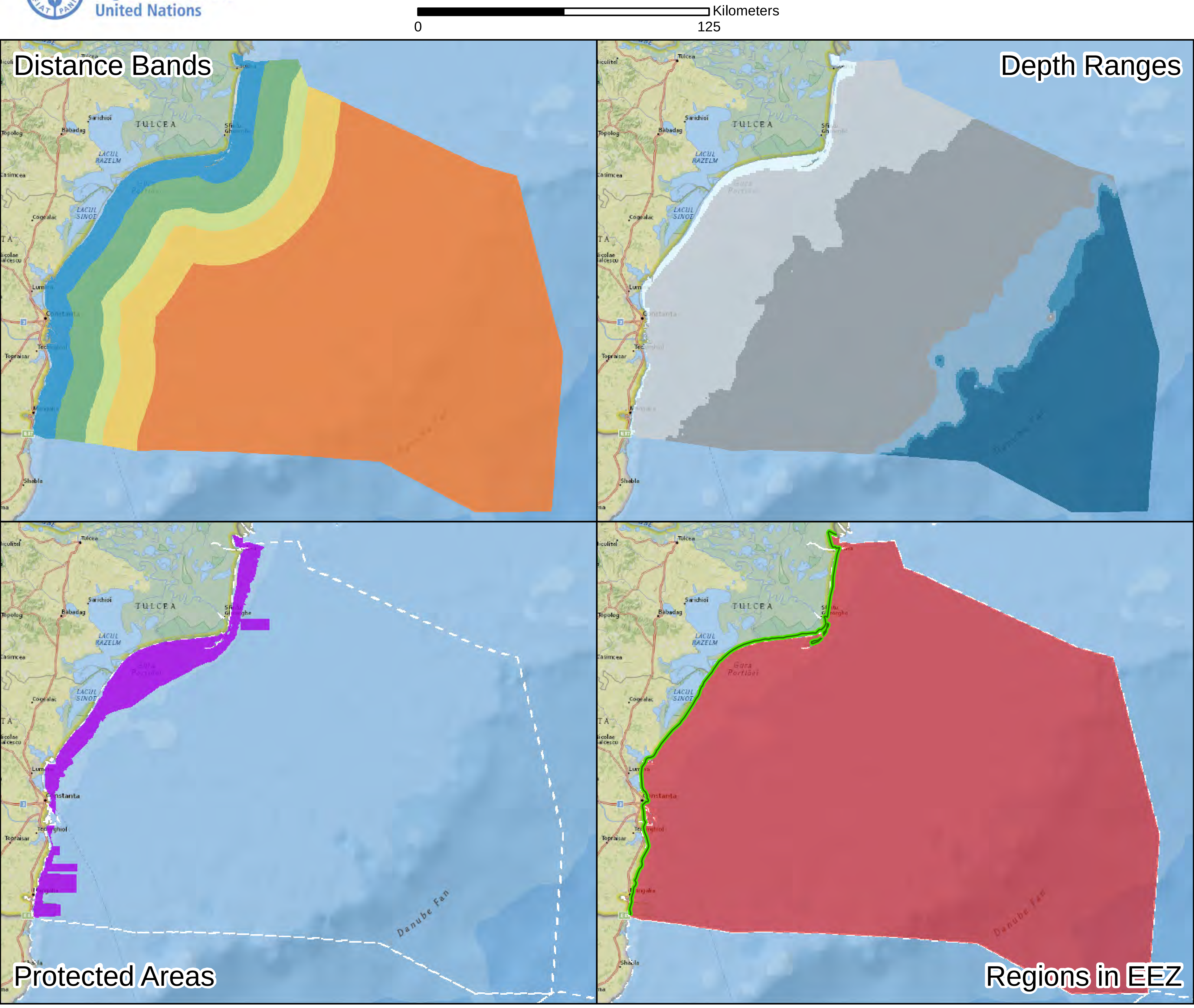

Distance Bands

- 0 to 5 NM
- 5 to 12 NM
- 12 to 16 NM
- 16 to 24 NM
- 24 to 200 NM

Depth Bands

- > -10m
- -10m to -50m
- -50m to -100m
- -100m to -150m
- -150m to -200m
- < -200m

Protected

- Protected Areas

Coastline Length

- 245 Km

Region

- Mediterranean

All Areas in Romania EEZ: Cell Values = Area in Km^2 [% Depth Band (Row), % Distance Band (Column), % EEZ]

	0 to 5 NM	5 to 12 NM	12 to 16 NM	16 to 24 NM	24 to 200 NM	> 200 NM	Total
> -10m	543 [100%, 27%, 2%]	0 [0%, 0%, 0%]	0 [0%, 0%, 0%]	0 [0%, 0%, 0%]	0 [0%, 0%, 0%]	0 [0%, 0%, 0%]	543 [2% of Total]
-10m to -50m	1,461 [21%, 73%, 5%]	2,700 [38%, 97%, 9%]	1,196 [17%, 78%, 4%]	1,511 [21%, 51%, 5%]	239 [3%, 1%, 1%]	0 [0%, 0%, 0%]	7,105 [24% of Total]
-50m to -100m	0 [0%, 0%, 0%]	92 [1%, 3%, 0%]	338 [3%, 22%, 1%]	1,454 [12%, 49%, 5%]	10,325 [85%, 51%, 35%]	0 [0%, 0%, 0%]	12,208 [41% of Total]
-100m to -150m	0 [0%, 0%, 0%]	0 [0%, 0%, 0%]	0 [0%, 0%, 0%]	0 [0%, 0%, 0%]	2,325 [100%, 12%, 8%]	0 [0%, 0%, 0%]	2,325 [8% of Total]
-150m to -200m	0 [0%, 0%, 0%]	0 [0%, 0%, 0%]	0 [0%, 0%, 0%]	0 [0%, 0%, 0%]	511 [100%, 3%, 2%]	0 [0%, 0%, 0%]	511 [2% of Total]
<-200m	0 [0%, 0%, 0%]	0 [0%, 0%, 0%]	0 [0%, 0%, 0%]	0 [0%, 0%, 0%]	6,764 [100%, 34%, 23%]	0 [0%, 0%, 0%]	6,764 [23% of Total]
Total	2,003 [7% of Total]	2,791 [9% of Total]	1,533 [5% of Total]	2,965 [10% of Total]	20,163 [68% of Total]	0.000 [0% of Total]	29,456 Km^2

Areas in Romania EEZ Excluding Protected Areas: Cell Values = Area in Km^2 [% Depth Band (Row), % Distance Band (Column), % EEZ]

1,778 [6%] Km^2 Protected	0 to 5 NM	5 to 12 NM	12 to 16 NM	16 to 24 NM	24 to 200 NM	> 200 NM	Total
> -10m	8 [100%, 1%, 0%]	0 [0%, 0%, 0%]	0 [0%, 0%, 0%]	0 [0%, 0%, 0%]	0 [0%, 0%, 0%]	0 [0%, 0%, 0%]	8 [0% of Total]
-10m to -50m	604 [10%, 99%, 2%]	2,313 [39%, 96%, 8%]	1,196 [20%, 78%, 4%]	1,511 [26%, 51%, 5%]	239 [4%, 1%, 1%]	0 [0%, 0%, 0%]	5,862 [21% of Total]
-50m to -100m	0 [0%, 0%, 0%]	92 [1%, 4%, 0%]	338 [3%, 22%, 1%]	1,454 [12%, 49%, 5%]	10,325 [85%, 51%, 37%]	0 [0%, 0%, 0%]	12,208 [44% of Total]
-100m to -150m	0 [0%, 0%, 0%]	0 [0%, 0%, 0%]	0 [0%, 0%, 0%]	0 [0%, 0%, 0%]	2,325 [100%, 12%, 8%]	0 [0%, 0%, 0%]	2,325 [8% of Total]
-150m to -200m	0 [0%, 0%, 0%]	0 [0%, 0%, 0%]	0 [0%, 0%, 0%]	0 [0%, 0%, 0%]	511 [100%, 3%, 2%]	0 [0%, 0%, 0%]	511 [2% of Total]
<-200m	0 [0%, 0%, 0%]	0 [0%, 0%, 0%]	0 [0%, 0%, 0%]	0 [0%, 0%, 0%]	6,764 [100%, 34%, 24%]	0 [0%, 0%, 0%]	6,764 [24% of Total]
Total	613 [2% of Total]	2,405 [9% of Total]	1,533 [6% of Total]	2,965 [11% of Total]	20,163 [73% of Total]	0.000 [0% of Total]	27,679 Km^2

The designations employed and the presentation of material in the map do not imply the expression of any opinion whatsoever on the part of FAO concerning the legal or constitutional status of any country, territory or sea area, or concerning the delimitation of frontiers.

Background reference map from National Geographic. Content may not reflect National Geographic's current map policy. Sources: National Geographic, Esri, DeLorme, HERE, UNEP-WCMC, USGS, NASA, ESA, METI, NRCAN, GEBCO, NOAA, increment P Corp.

Projection: Azimuthal Equidistant
Datum: WGS 1984
False Easting: 0.0000
False Northing: 0.0000
Central Meridian: 29.9956
Latitude Of Origin: 44.3263

Food and Agriculture Organization of the United Nations

Russia: Baltic and Black Sea Regions

©FAO, 2018

0 125 250 375 500 625 750 875 1,000 1,125 Kilometers

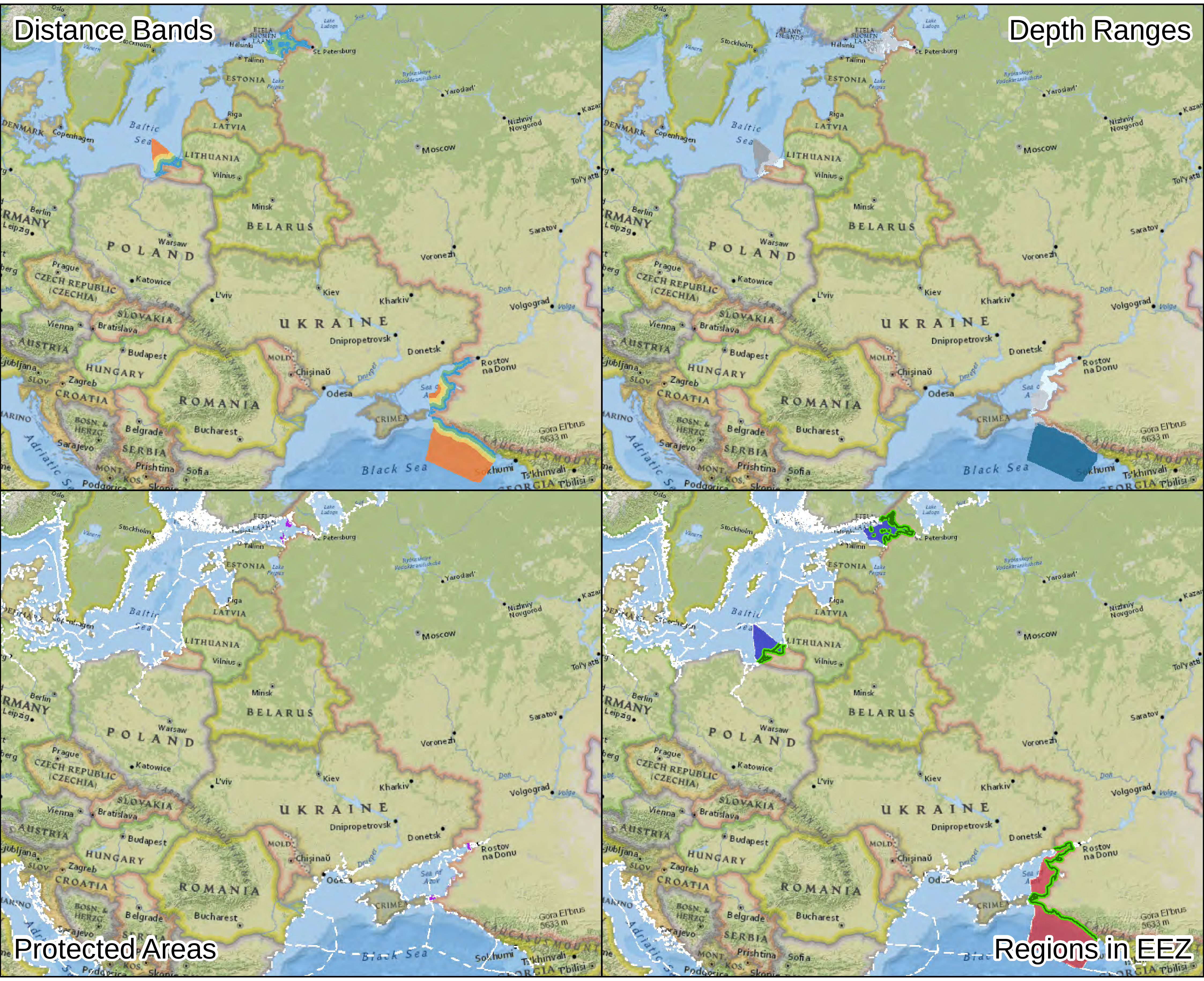

Distance Bands

- 0 to 5 NM
- 5 to 12 NM
- 12 to 16 NM
- 16 to 24 NM
- 24 to 200 NM

Depth Bands

- > -10m
- -10m to -50m
- -50m to -100m
- -100m to -150m
- -150m to -200m
- < -200m

Protected

- Protected Areas

Coastline Length

- 2,639 Km

Regions

- Mediterranean
- Baltic

All Areas in Russia EEZ: Cell Values = Area in Km^2 [% Depth Band (Row), % Distance Band (Column), % EEZ]

	0 to 5 NM	5 to 12 NM	12 to 16 NM	16 to 24 NM	24 to 200 NM	> 200 NM	Total
> -10m	8,568 [66%, 50%, 10%]	3,176 [24%, 20%, 4%]	572 [4%, 11%, 1%]	526 [4%, 6%, 1%]	173 [1%, 0%, 0%]	0 [0%, 0%, 0%]	13,015 [15% of Total]
-10m to -50m	6,596 [37%, 38%, 7%]	6,448 [37%, 41%, 7%]	1,321 [7%, 25%, 1%]	1,921 [11%, 22%, 2%]	1,359 [8%, 3%, 2%]	0 [0%, 0%, 0%]	17,646 [20% of Total]
-50m to -100m	1,223 [13%, 7%, 1%]	2,185 [23%, 14%, 2%]	1,023 [11%, 19%, 1%]	1,031 [11%, 12%, 1%]	3,941 [42%, 9%, 4%]	0 [0%, 0%, 0%]	9,403 [11% of Total]
-100m to -150m	154 [18%, 1%, 0%]	92 [11%, 1%, 0%]	62 [7%, 1%, 0%]	472 [55%, 5%, 1%]	82 [9%, 0%, 0%]	0 [0%, 0%, 0%]	862 [1% of Total]
-150m to -200m	95 [50%, 1%, 0%]	63 [33%, 0%, 0%]	10 [6%, 0%, 0%]	21 [11%, 0%, 0%]	2 [1%, 0%, 0%]	0 [0%, 0%, 0%]	191 [0% of Total]
<-200m	606 [1%, 4%, 1%]	3,680 [8%, 24%, 4%]	2,323 [5%, 44%, 3%]	4,617 [10%, 54%, 5%]	35,932 [76%, 87%, 41%]	0 [0%, 0%, 0%]	47,158 [53% of Total]
Total	17,242 [20% of Total]	15,644 [18% of Total]	5,312 [6% of Total]	8,587 [10% of Total]	41,489 [47% of Total]	0.000 [0% of Total]	88,274 Km^2

Areas in Russia EEZ Excluding Protected Areas: Cell Values = Area in Km^2 [% Depth Band (Row), % Distance Band (Column), % EEZ]

1,552 [2%] Km^2 Protected	0 to 5 NM	5 to 12 NM	12 to 16 NM	16 to 24 NM	24 to 200 NM	> 200 NM	Total
> -10m	7,550 [63%, 48%, 9%]	3,140 [26%, 20%, 4%]	572 [5%, 11%, 1%]	526 [4%, 6%, 1%]	173 [1%, 0%, 0%]	0 [0%, 0%, 0%]	11,961 [14% of Total]
-10m to -50m	6,215 [36%, 39%, 7%]	6,333 [37%, 41%, 7%]	1,321 [8%, 25%, 2%]	1,921 [11%, 22%, 2%]	1,359 [8%, 3%, 2%]	0 [0%, 0%, 0%]	17,150 [20% of Total]
-50m to -100m	1,220 [13%, 8%, 1%]	2,185 [23%, 14%, 3%]	1,023 [11%, 19%, 1%]	1,031 [11%, 12%, 1%]	3,941 [42%, 9%, 5%]	0 [0%, 0%, 0%]	9,400 [11% of Total]
-100m to -150m	154 [18%, 1%, 0%]	92 [11%, 1%, 0%]	62 [7%, 1%, 0%]	472 [55%, 5%, 1%]	82 [9%, 0%, 0%]	0 [0%, 0%, 0%]	862 [1% of Total]
-150m to -200m	95 [50%, 1%, 0%]	63 [33%, 0%, 0%]	10 [6%, 0%, 0%]	21 [11%, 0%, 0%]	2 [1%, 0%, 0%]	0 [0%, 0%, 0%]	191 [0% of Total]
<-200m	606 [1%, 4%, 1%]	3,680 [8%, 24%, 4%]	2,323 [5%, 44%, 3%]	4,617 [10%, 54%, 5%]	35,932 [76%, 87%, 41%]	0 [0%, 0%, 0%]	47,158 [54% of Total]
Total	15,841 [18% of Total]	15,493 [18% of Total]	5,312 [6% of Total]	8,587 [10% of Total]	41,489 [48% of Total]	0.000 [0% of Total]	86,722 Km^2

The designations employed and the presentation of material in the map do not imply the expression of any opinion whatsoever on the part of FAO concerning the legal or constitutional status of any country, territory or sea area, or concerning the delimitation of frontiers.

Background reference map from National Geographic. Content may not reflect National Geographic's current map policy. Sources: National Geographic, Esri, DeLorme, HERE, UNEP-WCMC, USGS, NASA, ESA, METI, NRCAN, GEBCO, NOAA, increment P Corp.

Projection: Azimuthal Equidistant
Datum: WGS 1984
False Easting: 0.0000
False Northing: 0.0000
Central Meridian: 29.4644
Latitude Of Origin: 51.5576

Saint Kitts and Nevis: Caribbean Region

©FAO, 2018

Food and Agriculture Organization of the United Nations

0 — 120 Kilometers

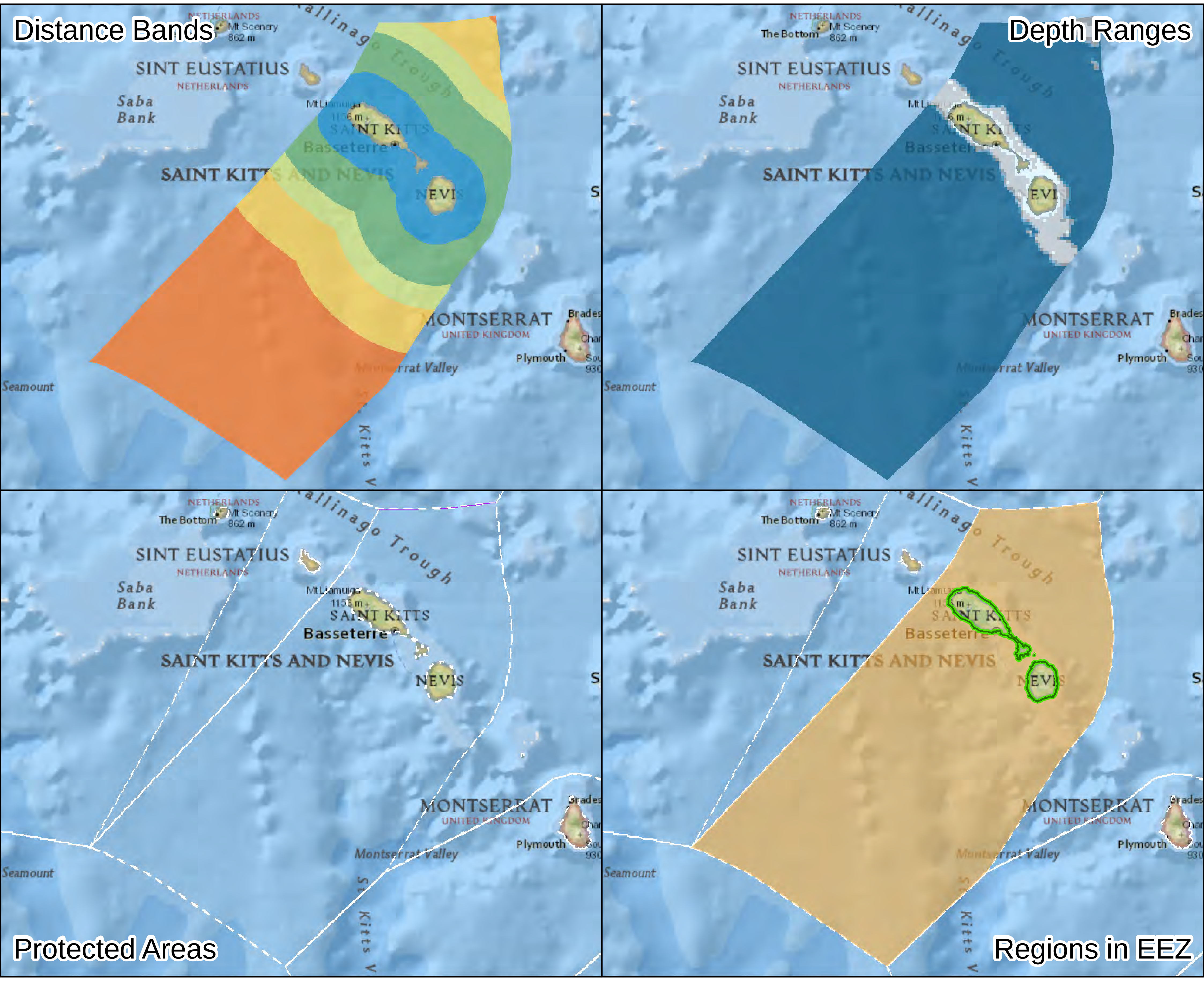

Distance Bands

- 0 to 5 NM
- 5 to 12 NM
- 12 to 16 NM
- 16 to 24 NM
- 24 to 200 NM

Depth Bands

- > -10m
- -10m to -50m
- -50m to -100m
- -100m to -150m
- -150m to -200m
- < -200m

Protected

- Protected Areas

Coastline Length

130 Km

Region

- Caribbean

All Areas in Saint Kitts and Nevis EEZ: Cell Values = Area in Km² [% Depth Band (Row), % Distance Band (Column), % EEZ]

	0 to 5 NM	5 to 12 NM	12 to 16 NM	16 to 24 NM	24 to 200 NM	> 200 NM	Total
> -10m	120 [99%, 9%, 1%]	0.821 [1%, 0%, 0%]	0 [0%, 0%, 0%]	0 [0%, 0%, 0%]	0 [0%, 0%, 0%]	0 [0%, 0%, 0%]	121 [1% of Total]
-10m to -50m	311 [79%, 24%, 3%]	54 [14%, 3%, 1%]	0 [0%, 0%, 0%]	30 [8%, 2%, 0%]	0.469 [0%, 0%, 0%]	0 [0%, 0%, 0%]	396 [4% of Total]
-50m to -100m	51 [48%, 4%, 1%]	8 [8%, 0%, 0%]	0 [0%, 0%, 0%]	44 [41%, 3%, 0%]	3 [3%, 0%, 0%]	0 [0%, 0%, 0%]	106 [1% of Total]
-100m to -150m	45 [70%, 4%, 0%]	7 [10%, 0%, 0%]	0 [0%, 0%, 0%]	12 [19%, 1%, 0%]	0.133 [0%, 0%, 0%]	0 [0%, 0%, 0%]	64 [1% of Total]
-150m to -200m	38 [77%, 3%, 0%]	3 [7%, 0%, 0%]	0 [0%, 0%, 0%]	8 [16%, 1%, 0%]	0 [0%, 0%, 0%]	0 [0%, 0%, 0%]	49 [1% of Total]
<-200m	715 [8%, 56%, 7%]	1,807 [21%, 96%, 19%]	904 [10%, 100%, 9%]	1,285 [15%, 93%, 13%]	4,085 [46%, 100%, 43%]	0 [0%, 0%, 0%]	8,796 [92% of Total]
Total	1,281 [13% of Total]	1,880 [20% of Total]	904 [9% of Total]	1,379 [14% of Total]	4,088 [43% of Total]	0.000 [0% of Total]	9,533 Km²

Areas in Saint Kitts and Nevis EEZ Excluding Protected Areas: Cell Values = Area in Km² [% Depth Band (Row), % Distance Band (Column), % EEZ]

6 [0%] Km² Protected	0 to 5 NM	5 to 12 NM	12 to 16 NM	16 to 24 NM	24 to 200 NM	> 200 NM	Total
> -10m	120 [99%, 9%, 1%]	0.821 [1%, 0%, 0%]	0 [0%, 0%, 0%]	0 [0%, 0%, 0%]	0 [0%, 0%, 0%]	0 [0%, 0%, 0%]	121 [1% of Total]
-10m to -50m	311 [79%, 24%, 3%]	54 [14%, 3%, 1%]	0 [0%, 0%, 0%]	30 [8%, 2%, 0%]	0.469 [0%, 0%, 0%]	0 [0%, 0%, 0%]	396 [4% of Total]
-50m to -100m	51 [49%, 4%, 1%]	8 [8%, 0%, 0%]	0 [0%, 0%, 0%]	42 [41%, 3%, 0%]	2 [2%, 0%, 0%]	0 [0%, 0%, 0%]	104 [1% of Total]
-100m to -150m	45 [71%, 4%, 0%]	7 [10%, 0%, 0%]	0 [0%, 0%, 0%]	12 [19%, 1%, 0%]	0.045 [0%, 0%, 0%]	0 [0%, 0%, 0%]	64 [1% of Total]
-150m to -200m	38 [77%, 3%, 0%]	3 [7%, 0%, 0%]	0 [0%, 0%, 0%]	8 [16%, 1%, 0%]	0 [0%, 0%, 0%]	0 [0%, 0%, 0%]	49 [1% of Total]
<-200m	715 [8%, 56%, 8%]	1,807 [21%, 96%, 19%]	903 [10%, 100%, 9%]	1,283 [15%, 93%, 13%]	4,085 [46%, 100%, 43%]	0 [0%, 0%, 0%]	8,793 [92% of Total]
Total	1,281 [13% of Total]	1,880 [20% of Total]	903 [9% of Total]	1,376 [14% of Total]	4,088 [43% of Total]	0.000 [0% of Total]	9,527 Km²

The designations employed and the presentation of material in the map do not imply the expression of any opinion whatsoever on the part of FAO concerning the legal or constitutional status of any country, territory or sea area, or concerning the delimitation of frontiers.

Background reference map from National Geographic. Content may not reflect National Geographic's current map policy. Sources: National Geographic, Esri, DeLorme, HERE, UNEP-WCMC, USGS, NASA, ESA, METI, NRCAN, GEBCO, NOAA, increment P Corp.

Projection: Azimuthal Equidistant
Datum: WGS 1984
False Easting: 0.0000
False Northing: 0.0000
Central Meridian: -62.9996
Latitude Of Origin: 17.0016

N W E S

Saint Lucia: Caribbean Region

©FAO, 2018

Food and Agriculture Organization of the United Nations

Kilometers 0 125

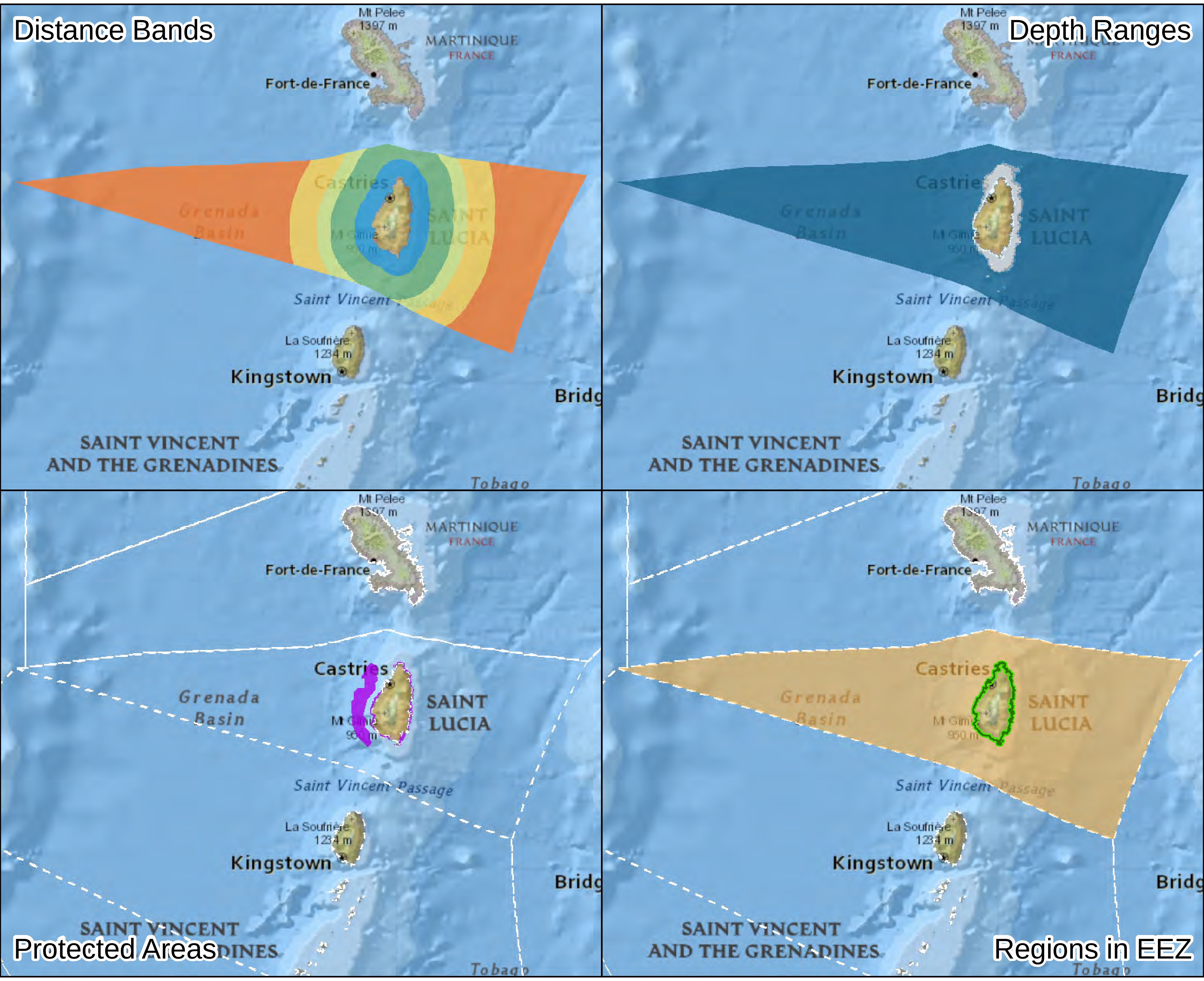

Distance Bands: 0 to 5 NM; 5 to 12 NM; 12 to 16 NM; 16 to 24 NM; 24 to 200 NM

Depth Bands: > -10m; -10m to -50m; -50m to -100m; -100m to -150m; -150m to -200m; < -200m

Protected: Protected Areas

Region: Caribbean

Coastline Length: 217 Km

All Areas in Saint Lucia EEZ: Cell Values = Area in Km^2 [% Depth Band (Row), % Distance Band (Column), % EEZ]

	0 to 5 NM	5 to 12 NM	12 to 16 NM	16 to 24 NM	24 to 200 NM	> 200 NM	Total
> -10m	79 [100%, 6%, 1%]	0 [0%, 0%, 0%]	0 [0%, 0%, 0%]	0 [0%, 0%, 0%]	0 [0%, 0%, 0%]	0 [0%, 0%, 0%]	79 [1% of Total]
-10m to -50m	342 [100%, 26%, 2%]	0 [0%, 0%, 0%]	0 [0%, 0%, 0%]	0 [0%, 0%, 0%]	0 [0%, 0%, 0%]	0 [0%, 0%, 0%]	342 [2% of Total]
-50m to -100m	86 [100%, 6%, 1%]	0 [0%, 0%, 0%]	0 [0%, 0%, 0%]	0 [0%, 0%, 0%]	0 [0%, 0%, 0%]	0 [0%, 0%, 0%]	86 [1% of Total]
-100m to -150m	42 [94%, 3%, 0%]	3 [6%, 0%, 0%]	0 [0%, 0%, 0%]	0 [0%, 0%, 0%]	0 [0%, 0%, 0%]	0 [0%, 0%, 0%]	44 [0% of Total]
-150m to -200m	44 [90%, 3%, 0%]	5 [10%, 0%, 0%]	0 [0%, 0%, 0%]	0 [0%, 0%, 0%]	0 [0%, 0%, 0%]	0 [0%, 0%, 0%]	48 [0% of Total]
<-200m	728 [5%, 55%, 5%]	2,492 [17%, 100%, 16%]	1,296 [9%, 100%, 8%]	2,402 [16%, 100%, 16%]	7,952 [53%, 100%, 51%]	0 [0%, 0%, 0%]	14,871 [96% of Total]
Total	1,319 [9% of Total]	2,500 [16% of Total]	1,296 [8% of Total]	2,402 [16% of Total]	7,952 [51% of Total]	0.000 [0% of Total]	15,469 Km^2

Areas in Saint Lucia EEZ Excluding Protected Areas: Cell Values = Area in Km^2 [% Depth Band (Row), % Distance Band (Column), % EEZ]

381 [2%] Km^2 Protected	0 to 5 NM	5 to 12 NM	12 to 16 NM	16 to 24 NM	24 to 200 NM	> 200 NM	Total
> -10m	33 [100%, 3%, 0%]	0 [0%, 0%, 0%]	0 [0%, 0%, 0%]	0 [0%, 0%, 0%]	0 [0%, 0%, 0%]	0 [0%, 0%, 0%]	33 [0% of Total]
-10m to -50m	326 [100%, 32%, 2%]	0 [0%, 0%, 0%]	0 [0%, 0%, 0%]	0 [0%, 0%, 0%]	0 [0%, 0%, 0%]	0 [0%, 0%, 0%]	326 [2% of Total]
-50m to -100m	83 [100%, 8%, 1%]	0 [0%, 0%, 0%]	0 [0%, 0%, 0%]	0 [0%, 0%, 0%]	0 [0%, 0%, 0%]	0 [0%, 0%, 0%]	83 [1% of Total]
-100m to -150m	40 [94%, 4%, 0%]	3 [6%, 0%, 0%]	0 [0%, 0%, 0%]	0 [0%, 0%, 0%]	0 [0%, 0%, 0%]	0 [0%, 0%, 0%]	43 [0% of Total]
-150m to -200m	43 [90%, 4%, 0%]	5 [10%, 0%, 0%]	0 [0%, 0%, 0%]	0 [0%, 0%, 0%]	0 [0%, 0%, 0%]	0 [0%, 0%, 0%]	47 [0% of Total]
<-200m	502 [3%, 49%, 3%]	2,403 [17%, 100%, 16%]	1,296 [9%, 100%, 9%]	2,402 [16%, 100%, 16%]	7,952 [55%, 100%, 53%]	0 [0%, 0%, 0%]	14,556 [96% of Total]
Total	1,028 [7% of Total]	2,410 [16% of Total]	1,296 [9% of Total]	2,402 [16% of Total]	7,952 [53% of Total]	0.000 [0% of Total]	15,089 Km^2

The designations employed and the presentation of material in the map do not imply the expression of any opinion whatsoever on the part of FAO concerning the legal or constitutional status of any country, territory or sea area, or concerning the delimitation of frontiers.

Background reference map from National Geographic. Content may not reflect National Geographic's current map policy. Sources: National Geographic, Esri, DeLorme, HERE, UNEP-WCMC, USGS, NASA, ESA, METI, NRCAN, GEBCO, NOAA, increment P Corp.

Projection: Azimuthal Equidistant
Datum: WGS 1984
False Easting: 0.0000
False Northing: 0.0000
Central Meridian: -61.4319
Latitude Of Origin: 13.7579

N W E S

Food and Agriculture Organization of the United Nations

Saint Vincent and the Grenadines: Caribbean Region

©FAO, 2018

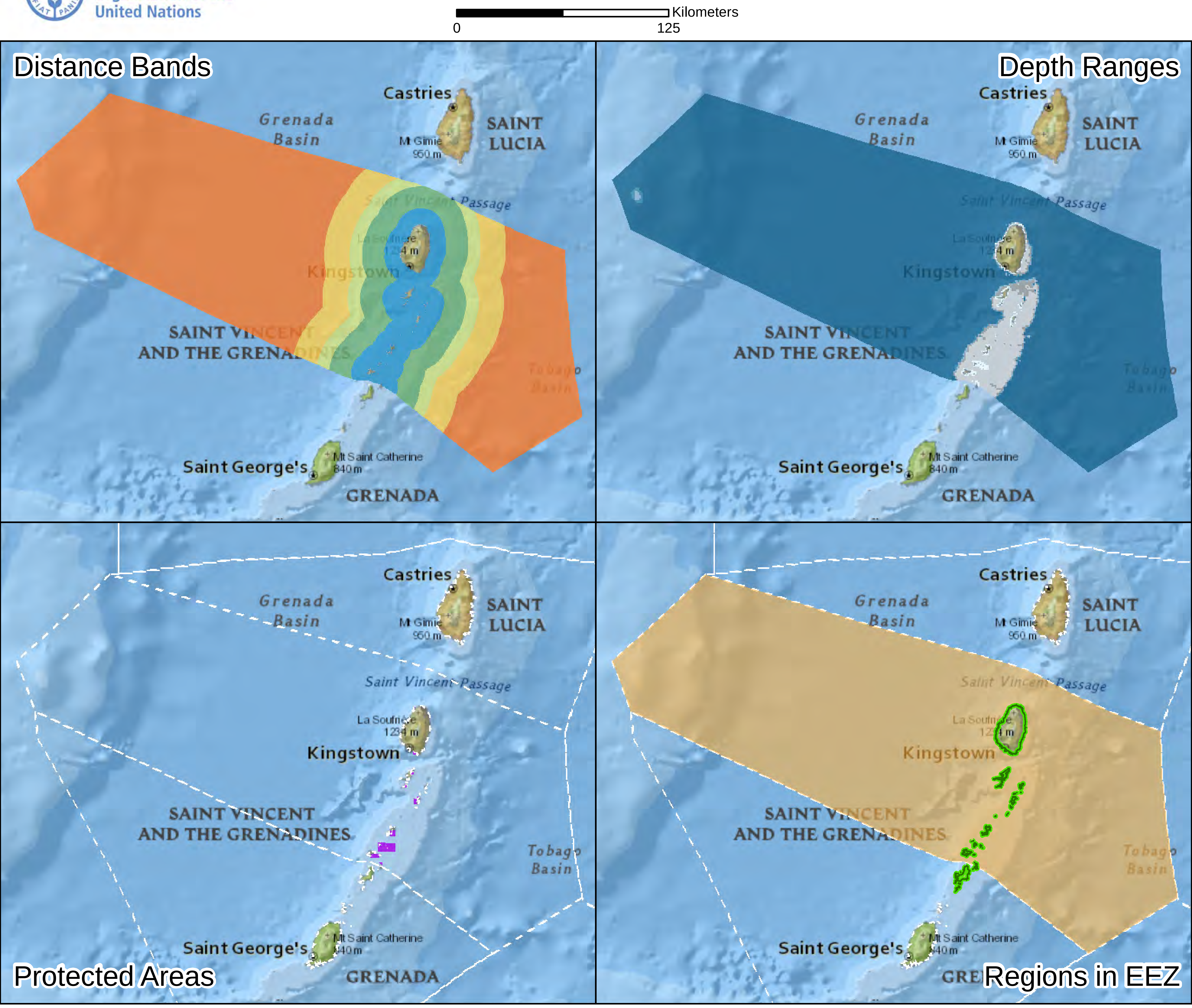

Distance Bands		Depth Bands		Protected	Region
0 to 5 NM	16 to 24 NM	> -10m	-100m to -150m	Protected Areas	Caribbean
5 to 12 NM	24 to 200 NM	-10m to -50m	-150m to -200m		
12 to 16 NM		-50m to -100m	< -200m		

Coastline Length: 360 Km

All Areas in Saint Vincent and the Grenadines EEZ: Cell Values = Area in Km^2 [% Depth Band (Row), % Distance Band (Column), % EEZ]

	0 to 5 NM	5 to 12 NM	12 to 16 NM	16 to 24 NM	24 to 200 NM	> 200 NM	Total
> -10m	153 [100%, 6%, 0%]	0 [0%, 0%, 0%]	0 [0%, 0%, 0%]	0 [0%, 0%, 0%]	0 [0%, 0%, 0%]	0 [0%, 0%, 0%]	153 [0% of Total]
-10m to -50m	1,140 [83%, 42%, 3%]	238 [17%, 7%, 1%]	0 [0%, 0%, 0%]	0 [0%, 0%, 0%]	0 [0%, 0%, 0%]	0 [0%, 0%, 0%]	1,378 [4% of Total]
-50m to -100m	203 [83%, 7%, 1%]	42 [17%, 1%, 0%]	0 [0%, 0%, 0%]	0 [0%, 0%, 0%]	0 [0%, 0%, 0%]	0 [0%, 0%, 0%]	245 [1% of Total]
-100m to -150m	71 [62%, 3%, 0%]	28 [24%, 1%, 0%]	0 [0%, 0%, 0%]	0 [0%, 0%, 0%]	16 [14%, 0%, 0%]	0 [0%, 0%, 0%]	115 [0% of Total]
-150m to -200m	64 [57%, 2%, 0%]	16 [14%, 0%, 0%]	0 [0%, 0%, 0%]	0 [0%, 0%, 0%]	32 [28%, 0%, 0%]	0 [0%, 0%, 0%]	112 [0% of Total]
<-200m	1,081 [3%, 40%, 3%]	3,261 [9%, 91%, 9%]	1,978 [6%, 100%, 5%]	3,825 [11%, 100%, 11%]	24,230 [70%, 100%, 67%]	0 [0%, 0%, 0%]	34,375 [94% of Total]
Total	2,713 [7% of Total]	3,585 [10% of Total]	1,978 [5% of Total]	3,825 [11% of Total]	24,278 [67% of Total]	0.000 [0% of Total]	36,379 Km^2

Areas in Saint Vincent and the Grenadines EEZ Excluding Protected Areas: Cell Values = Area in Km^2 [% Depth Band (Row), % Distance Band (Column), % EEZ]

84 [0%] Km^2 Protected	0 to 5 NM	5 to 12 NM	12 to 16 NM	16 to 24 NM	24 to 200 NM	> 200 NM	Total
> -10m	103 [100%, 4%, 0%]	0 [0%, 0%, 0%]	0 [0%, 0%, 0%]	0 [0%, 0%, 0%]	0 [0%, 0%, 0%]	0 [0%, 0%, 0%]	103 [0% of Total]
-10m to -50m	1,107 [82%, 42%, 3%]	238 [18%, 7%, 1%]	0 [0%, 0%, 0%]	0 [0%, 0%, 0%]	0 [0%, 0%, 0%]	0 [0%, 0%, 0%]	1,345 [4% of Total]
-50m to -100m	203 [83%, 8%, 1%]	42 [17%, 1%, 0%]	0 [0%, 0%, 0%]	0 [0%, 0%, 0%]	0 [0%, 0%, 0%]	0 [0%, 0%, 0%]	245 [1% of Total]
-100m to -150m	71 [62%, 3%, 0%]	28 [24%, 1%, 0%]	0 [0%, 0%, 0%]	0 [0%, 0%, 0%]	16 [14%, 0%, 0%]	0 [0%, 0%, 0%]	115 [0% of Total]
-150m to -200m	64 [57%, 2%, 0%]	16 [14%, 0%, 0%]	0 [0%, 0%, 0%]	0 [0%, 0%, 0%]	32 [28%, 0%, 0%]	0 [0%, 0%, 0%]	112 [0% of Total]
<-200m	1,081 [3%, 41%, 3%]	3,261 [9%, 91%, 9%]	1,978 [6%, 100%, 5%]	3,825 [11%, 100%, 11%]	24,230 [70%, 100%, 67%]	0 [0%, 0%, 0%]	34,375 [95% of Total]
Total	2,629 [7% of Total]	3,584 [10% of Total]	1,978 [5% of Total]	3,825 [11% of Total]	24,278 [67% of Total]	0.000 [0% of Total]	36,295 Km^2

The designations employed and the presentation of material in the map do not imply the expression of any opinion whatsoever on the part of FAO concerning the legal or constitutional status of any country, territory or sea area, or concerning the delimitation of frontiers.

Background reference map from National Geographic. Content may not reflect National Geographic's current map policy. Sources: National Geographic, Esri, DeLorme, HERE, UNEP-WCMC, USGS, NASA, ESA, METI, NRCAN, GEBCO, NOAA, increment P Corp.

Projection: Azimuthal Equidistant
Datum: WGS 1984
False Easting: 0.0000
False Northing: 0.0000
Central Meridian: -61.8321
Latitude Of Origin: 13.0712

Food and Agriculture Organization of the United Nations

Slovenia: Mediterranean Region

©FAO, 2018

Kilometers
0 10

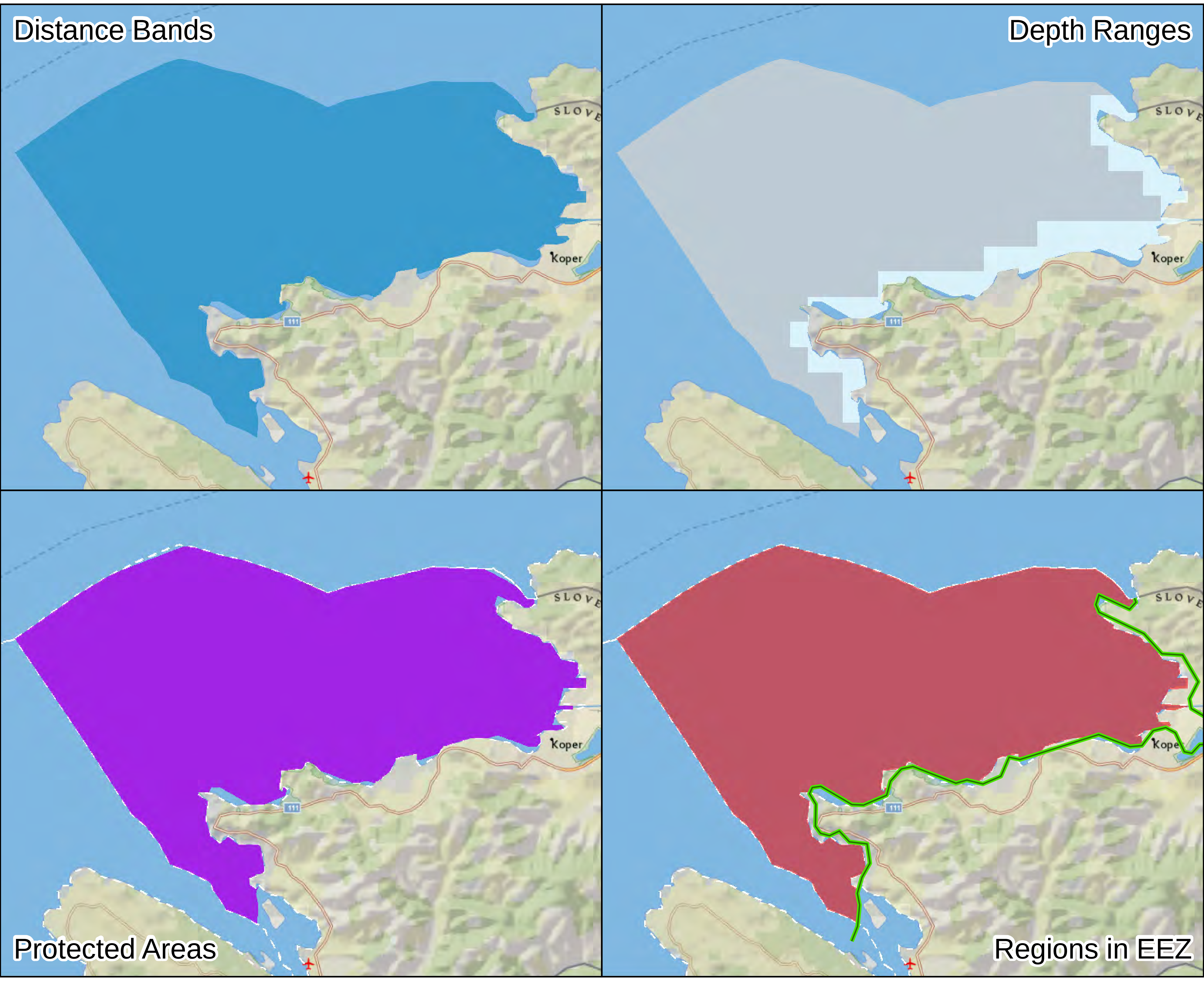

Distance Bands
0 to 5 NM

Depth Bands
> -10m
-10m to -50m

Protected
Protected Areas

Coastline Length
35 Km

Region
Mediterranean

All Areas in Slovenia EEZ: Cell Values = Area in Km^2 [% Depth Band (Row), % Distance Band (Column), % EEZ]

	0 to 5 NM	5 to 12 NM	12 to 16 NM	16 to 24 NM	24 to 200 NM	> 200 NM	Total
> -10m	19 [100%, 13%, 13%]	0 [0%, 0%, 0%]	0 [0%, 0%, 0%]	0 [0%, 0%, 0%]	0 [0%, 0%, 0%]	0 [0%, 0%, 0%]	19 [13% of Total]
-10m to -50m	125 [100%, 87%, 87%]	0 [0%, 0%, 0%]	0 [0%, 0%, 0%]	0 [0%, 0%, 0%]	0 [0%, 0%, 0%]	0 [0%, 0%, 0%]	125 [87% of Total]
-50m to -100m	0 [0%, 0%, 0%]	0 [0%, 0%, 0%]	0 [0%, 0%, 0%]	0 [0%, 0%, 0%]	0 [0%, 0%, 0%]	0 [0%, 0%, 0%]	0.000 [0% of Total]
-100m to -150m	0 [0%, 0%, 0%]	0 [0%, 0%, 0%]	0 [0%, 0%, 0%]	0 [0%, 0%, 0%]	0 [0%, 0%, 0%]	0 [0%, 0%, 0%]	0.000 [0% of Total]
-150m to -200m	0 [0%, 0%, 0%]	0 [0%, 0%, 0%]	0 [0%, 0%, 0%]	0 [0%, 0%, 0%]	0 [0%, 0%, 0%]	0 [0%, 0%, 0%]	0.000 [0% of Total]
<-200m	0 [0%, 0%, 0%]	0 [0%, 0%, 0%]	0 [0%, 0%, 0%]	0 [0%, 0%, 0%]	0 [0%, 0%, 0%]	0 [0%, 0%, 0%]	0.000 [0% of Total]
Total	144 [100% of Total]	0.000 [0% of Total]	0.000 [0% of Total]	0.000 [0% of Total]	0.000 [0% of Total]	0.000 [0% of Total]	144 Km^2

Areas in Slovenia EEZ Excluding Protected Areas: Cell Values = Area in Km^2 [% Depth Band (Row), % Distance Band (Column), % EEZ]

143 [100%] Km^2 Protected	0 to 5 NM	5 to 12 NM	12 to 16 NM	16 to 24 NM	24 to 200 NM	> 200 NM	Total
> -10m	0.298 [100%, 47%, 47%]	0 [0%, 0%, 0%]	0 [0%, 0%, 0%]	0 [0%, 0%, 0%]	0 [0%, 0%, 0%]	0 [0%, 0%, 0%]	0.298 [47% of Total]
-10m to -50m	0.334 [100%, 53%, 53%]	0 [0%, 0%, 0%]	0 [0%, 0%, 0%]	0 [0%, 0%, 0%]	0 [0%, 0%, 0%]	0 [0%, 0%, 0%]	0.334 [53% of Total]
-50m to -100m	0 [0%, 0%, 0%]	0 [0%, 0%, 0%]	0 [0%, 0%, 0%]	0 [0%, 0%, 0%]	0 [0%, 0%, 0%]	0 [0%, 0%, 0%]	0.000 [0% of Total]
-100m to -150m	0 [0%, 0%, 0%]	0 [0%, 0%, 0%]	0 [0%, 0%, 0%]	0 [0%, 0%, 0%]	0 [0%, 0%, 0%]	0 [0%, 0%, 0%]	0.000 [0% of Total]
-150m to -200m	0 [0%, 0%, 0%]	0 [0%, 0%, 0%]	0 [0%, 0%, 0%]	0 [0%, 0%, 0%]	0 [0%, 0%, 0%]	0 [0%, 0%, 0%]	0.000 [0% of Total]
<-200m	0 [0%, 0%, 0%]	0 [0%, 0%, 0%]	0 [0%, 0%, 0%]	0 [0%, 0%, 0%]	0 [0%, 0%, 0%]	0 [0%, 0%, 0%]	0.000 [0% of Total]
Total	0.632 [100% of Total]	0.000 [0% of Total]	0.000 [0% of Total]	0.000 [0% of Total]	0.000 [0% of Total]	0.000 [0% of Total]	0.632 Km^2

The designations employed and the presentation of material in the map do not imply the expression of any opinion whatsoever on the part of FAO concerning the legal or constitutional status of any country, territory or sea area, or concerning the delimitation of frontiers.

Background reference map from National Geographic. Content may not reflect National Geographic's current map policy. Sources: National Geographic, Esri, DeLorme, HERE, UNEP-WCMC, USGS, NASA, ESA, METI, NRCAN, GEBCO, NOAA, increment P Corp.

Projection: Azimuthal Equidistant
Datum: WGS 1984
False Easting: 0.0000
False Northing: 0.0000
Central Meridian: 13.6116
Latitude Of Origin: 45.5496

Food and Agriculture Organization of the United Nations

Spain: Atlantic and Mediterranean Regions

©FAO, 2018

0 125 250 375 500 625 750 875 1,000 1,125 Kilometers

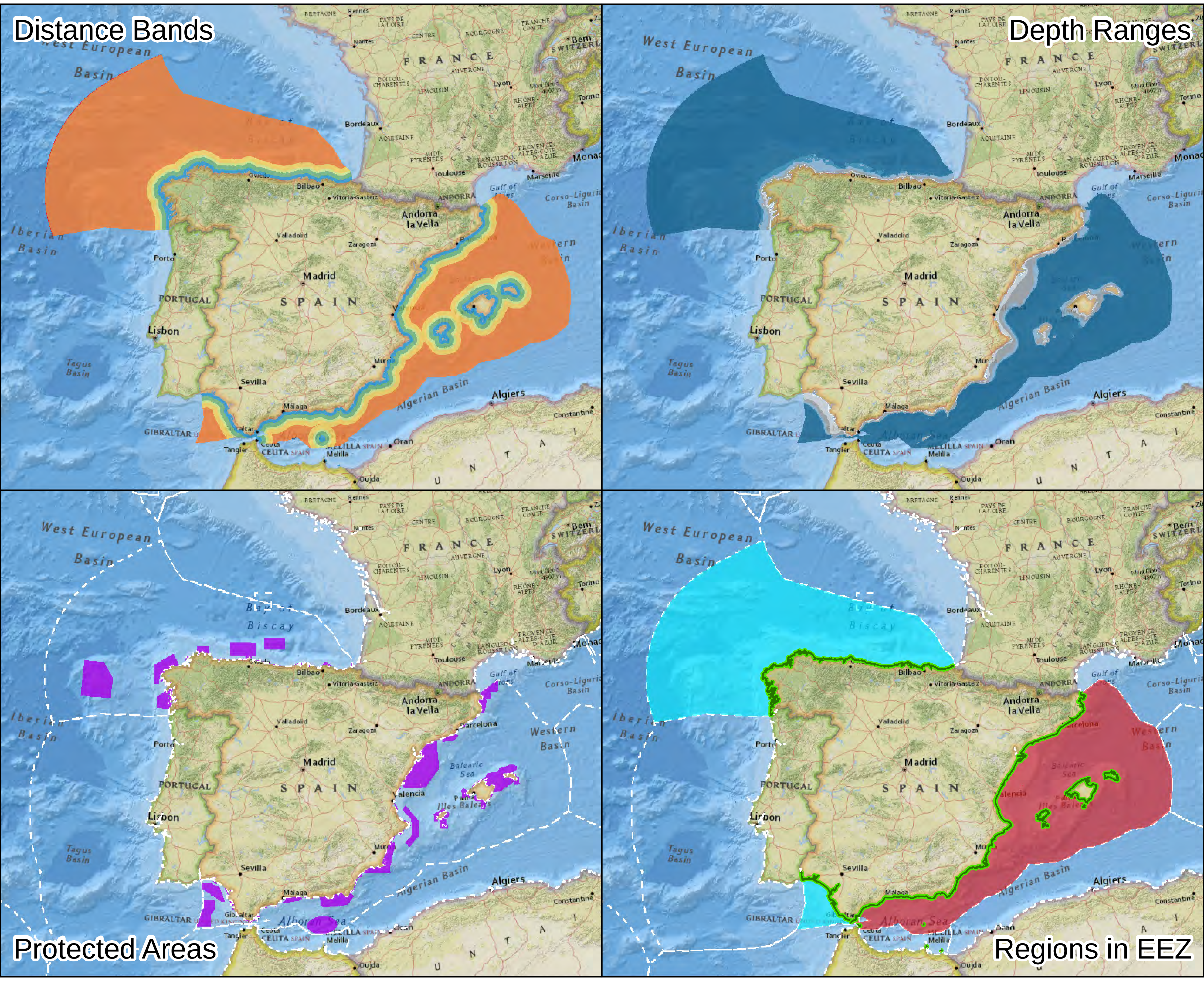

Distance Bands
- 0 to 5 NM
- 5 to 12 NM
- 12 to 16 NM
- 16 to 24 NM
- 24 to 200 NM
- > 200 NM

Depth Bands
- > -10m
- -10m to -50m
- -50m to -100m
- -100m to -150m
- -150m to -200m
- < -200m

Protected
- Protected Areas

Coastline Length
4,843 Km

Regions
- Mediterranean
- Atlantic

All Areas in Spain EEZ: Cell Values = Area in Km^2 [% Depth Band (Row), % Distance Band (Column), % EEZ]

	0 to 5 NM	5 to 12 NM	12 to 16 NM	16 to 24 NM	24 to 200 NM	> 200 NM	Total
> -10m	2,599 [100%, 8%, 0%]	0 [0%, 0%, 0%]	0 [0%, 0%, 0%]	0 [0%, 0%, 0%]	0 [0%, 0%, 0%]	0 [0%, 0%, 0%]	2,599 [0% of Total]
-10m to -50m	10,424 [79%, 32%, 2%]	2,478 [19%, 6%, 0%]	249 [2%, 1%, 0%]	2 [0%, 0%, 0%]	6 [0%, 0%, 0%]	0 [0%, 0%, 0%]	13,158 [2% of Total]
-50m to -100m	11,495 [48%, 36%, 2%]	7,412 [31%, 17%, 1%]	2,277 [9%, 9%, 0%]	1,876 [8%, 4%, 0%]	1,097 [5%, 0%, 0%]	0 [0%, 0%, 0%]	24,157 [4% of Total]
-100m to -150m	4,366 [24%, 14%, 1%]	8,819 [48%, 20%, 2%]	2,204 [12%, 9%, 0%]	2,347 [13%, 4%, 0%]	702 [4%, 0%, 0%]	0 [0%, 0%, 0%]	18,439 [3% of Total]
-150m to -200m	752 [7%, 2%, 0%]	5,910 [55%, 13%, 1%]	2,244 [21%, 9%, 0%]	1,697 [16%, 3%, 0%]	184 [2%, 0%, 0%]	0 [0%, 0%, 0%]	10,786 [2% of Total]
<-200m	2,442 [0%, 8%, 0%]	19,194 [4%, 44%, 3%]	18,758 [4%, 73%, 3%]	46,582 [9%, 89%, 8%]	403,499 [82%, 100%, 72%]	694 [0%, 100%, 0%]	491,168 [88% of Total]
Total	32,078 [6% of Total]	43,812 [8% of Total]	25,732 [5% of Total]	52,503 [9% of Total]	405,488 [72% of Total]	694 [0% of Total]	560,307 Km^2

Areas in Spain EEZ Excluding Protected Areas: Cell Values = Area in Km^2 [% Depth Band (Row), % Distance Band (Column), % EEZ]

63,008 [11%] Km^2 Protected	0 to 5 NM	5 to 12 NM	12 to 16 NM	16 to 24 NM	24 to 200 NM	> 200 NM	Total
> -10m	1,119 [100%, 7%, 0%]	0 [0%, 0%, 0%]	0 [0%, 0%, 0%]	0 [0%, 0%, 0%]	0 [0%, 0%, 0%]	0 [0%, 0%, 0%]	1,119 [0% of Total]
-10m to -50m	4,992 [81%, 30%, 1%]	1,044 [17%, 4%, 0%]	102 [2%, 1%, 0%]	1 [0%, 0%, 0%]	0 [0%, 0%, 0%]	0 [0%, 0%, 0%]	6,140 [1% of Total]
-50m to -100m	6,189 [54%, 38%, 1%]	4,044 [36%, 14%, 1%]	799 [7%, 4%, 0%]	169 [1%, 0%, 0%]	165 [1%, 0%, 0%]	0 [0%, 0%, 0%]	11,366 [2% of Total]
-100m to -150m	2,298 [20%, 14%, 0%]	6,046 [52%, 21%, 1%]	1,603 [14%, 8%, 0%]	1,372 [12%, 3%, 0%]	205 [2%, 0%, 0%]	0 [0%, 0%, 0%]	11,523 [2% of Total]
-150m to -200m	341 [5%, 2%, 0%]	3,199 [49%, 11%, 1%]	1,560 [24%, 8%, 0%]	1,363 [21%, 3%, 0%]	83 [1%, 0%, 0%]	0 [0%, 0%, 0%]	6,546 [1% of Total]
<-200m	1,559 [0%, 9%, 0%]	13,971 [3%, 49%, 3%]	15,222 [3%, 79%, 3%]	42,607 [9%, 94%, 9%]	386,552 [84%, 100%, 78%]	694 [0%, 100%, 0%]	460,605 [93% of Total]
Total	16,498 [3% of Total]	28,305 [6% of Total]	19,285 [4% of Total]	45,512 [9% of Total]	387,005 [78% of Total]	694 [0% of Total]	497,299 Km^2

The designations employed and the presentation of material in the map do not imply the expression of any opinion whatsoever on the part of FAO concerning the legal or constitutional status of any country, territory or sea area, or concerning the delimitation of frontiers.

Background reference map from National Geographic. Content may not reflect National Geographic's current map policy. Sources: National Geographic, Esri, DeLorme, HERE, UNEP-WCMC, USGS, NASA, ESA, METI, NRCAN, GEBCO, NOAA, increment P Corp.

Projection: Azimuthal Equidistant
Datum: WGS 1984
False Easting: 0.0000
False Northing: 0.0000
Central Meridian: -3.7765
Latitude Of Origin: 41.0142

N W E S

Food and Agriculture Organization of the United Nations

Suriname: Caribbean Region

©FAO, 2018

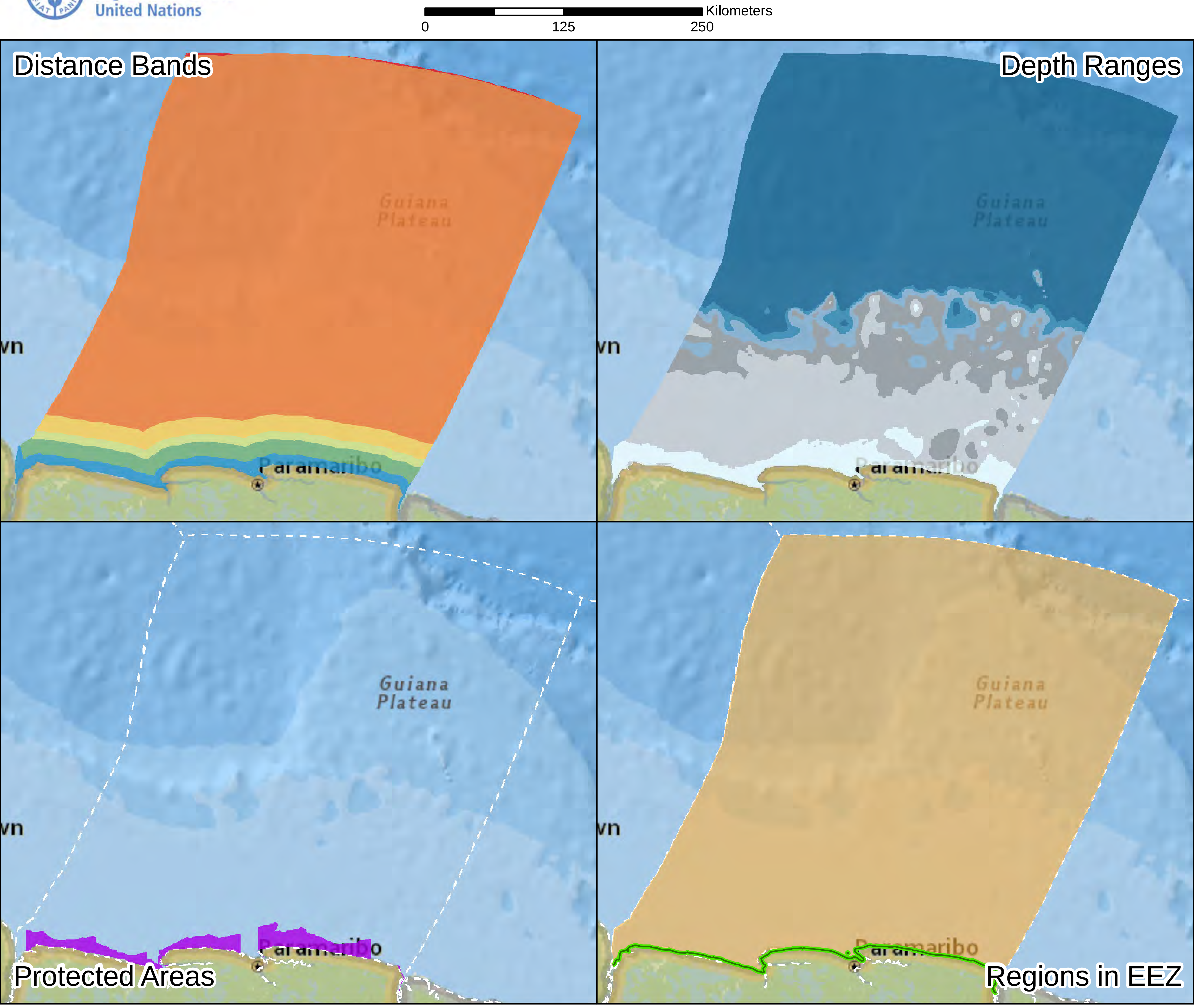

Distance Bands

- 0 to 5 NM
- 5 to 12 NM
- 12 to 16 NM
- 16 to 24 NM
- 24 to 200 NM
- > 200 NM

Depth Bands

- > -10m
- -10m to -50m
- -50m to -100m
- -100m to -150m
- -150m to -200m
- < -200m

Protected

- Protected Areas

Coastline Length

444 Km

Region

- Caribbean

All Areas in Suriname EEZ: Cell Values = Area in Km^2 [% Depth Band (Row), % Distance Band (Column), % EEZ]

	0 to 5 NM	5 to 12 NM	12 to 16 NM	16 to 24 NM	24 to 200 NM	> 200 NM	Total
> -10m	3,097 [48%, 90%, 2%]	2,665 [41%, 55%, 2%]	375 [6%, 14%, 0%]	133 [2%, 2%, 0%]	175 [3%, 0%, 0%]	0 [0%, 0%, 0%]	6,445 [5% of Total]
-10m to -50m	353 [1%, 10%, 0%]	1,937 [7%, 40%, 1%]	2,070 [8%, 77%, 2%]	4,977 [19%, 93%, 4%]	17,107 [65%, 15%, 13%]	0 [0%, 0%, 0%]	26,444 [20% of Total]
-50m to -100m	0 [0%, 0%, 0%]	236 [2%, 5%, 0%]	249 [2%, 9%, 0%]	247 [2%, 5%, 0%]	13,239 [95%, 11%, 10%]	0 [0%, 0%, 0%]	13,971 [10% of Total]
-100m to -150m	0 [0%, 0%, 0%]	0 [0%, 0%, 0%]	0 [0%, 0%, 0%]	0 [0%, 0%, 0%]	5,437 [100%, 5%, 4%]	0 [0%, 0%, 0%]	5,437 [4% of Total]
-150m to -200m	0 [0%, 0%, 0%]	0 [0%, 0%, 0%]	0 [0%, 0%, 0%]	0 [0%, 0%, 0%]	2,729 [100%, 2%, 2%]	0 [0%, 0%, 0%]	2,729 [2% of Total]
<-200m	0 [0%, 0%, 0%]	0 [0%, 0%, 0%]	0 [0%, 0%, 0%]	0 [0%, 0%, 0%]	78,217 [100%, 67%, 59%]	254 [0%, 100%, 0%]	78,471 [59% of Total]
Total	3,450 [3% of Total]	4,838 [4% of Total]	2,693 [2% of Total]	5,356 [4% of Total]	116,904 [88% of Total]	254 [0% of Total]	133,496 Km^2

Areas in Suriname EEZ Excluding Protected Areas: Cell Values = Area in Km^2 [% Depth Band (Row), % Distance Band (Column), % EEZ]

3,386 [3%] Km^2 Protected	0 to 5 NM	5 to 12 NM	12 to 16 NM	16 to 24 NM	24 to 200 NM	> 200 NM	Total
> -10m	955 [26%, 96%, 1%]	2,038 [55%, 52%, 2%]	375 [10%, 14%, 0%]	133 [4%, 2%, 0%]	175 [5%, 0%, 0%]	0 [0%, 0%, 0%]	3,675 [3% of Total]
-10m to -50m	43 [0%, 4%, 0%]	1,630 [6%, 42%, 1%]	2,070 [8%, 77%, 2%]	4,977 [19%, 93%, 4%]	17,107 [66%, 15%, 13%]	0 [0%, 0%, 0%]	25,827 [20% of Total]
-50m to -100m	0 [0%, 0%, 0%]	236 [2%, 6%, 0%]	249 [2%, 9%, 0%]	247 [2%, 5%, 0%]	13,239 [95%, 11%, 10%]	0 [0%, 0%, 0%]	13,971 [11% of Total]
-100m to -150m	0 [0%, 0%, 0%]	0 [0%, 0%, 0%]	0 [0%, 0%, 0%]	0 [0%, 0%, 0%]	5,437 [100%, 5%, 4%]	0 [0%, 0%, 0%]	5,437 [4% of Total]
-150m to -200m	0 [0%, 0%, 0%]	0 [0%, 0%, 0%]	0 [0%, 0%, 0%]	0 [0%, 0%, 0%]	2,729 [100%, 2%, 2%]	0 [0%, 0%, 0%]	2,729 [2% of Total]
<-200m	0 [0%, 0%, 0%]	0 [0%, 0%, 0%]	0 [0%, 0%, 0%]	0 [0%, 0%, 0%]	78,217 [100%, 67%, 60%]	254 [0%, 100%, 0%]	78,471 [60% of Total]
Total	998 [1% of Total]	3,905 [3% of Total]	2,693 [2% of Total]	5,356 [4% of Total]	116,904 [90% of Total]	254 [0% of Total]	130,110 Km^2

The designations employed and the presentation of material in the map do not imply the expression of any opinion whatsoever on the part of FAO concerning the legal or constitutional status of any country, territory or sea area, or concerning the delimitation of frontiers.

Background reference map from National Geographic. Content may not reflect National Geographic's current map policy. Sources: National Geographic, Esri, DeLorme, HERE, UNEP-WCMC, USGS, NASA, ESA, METI, NRCAN, GEBCO, NOAA, increment P Corp.

Projection: Azimuthal Equidistant
Datum: WGS 1984
False Easting: 0.0000
False Northing: 0.0000
Central Meridian: -54.8338
Latitude Of Origin: 7.4874

Sweden: Baltic Region

©FAO, 2018

Food and Agriculture Organization of the United Nations

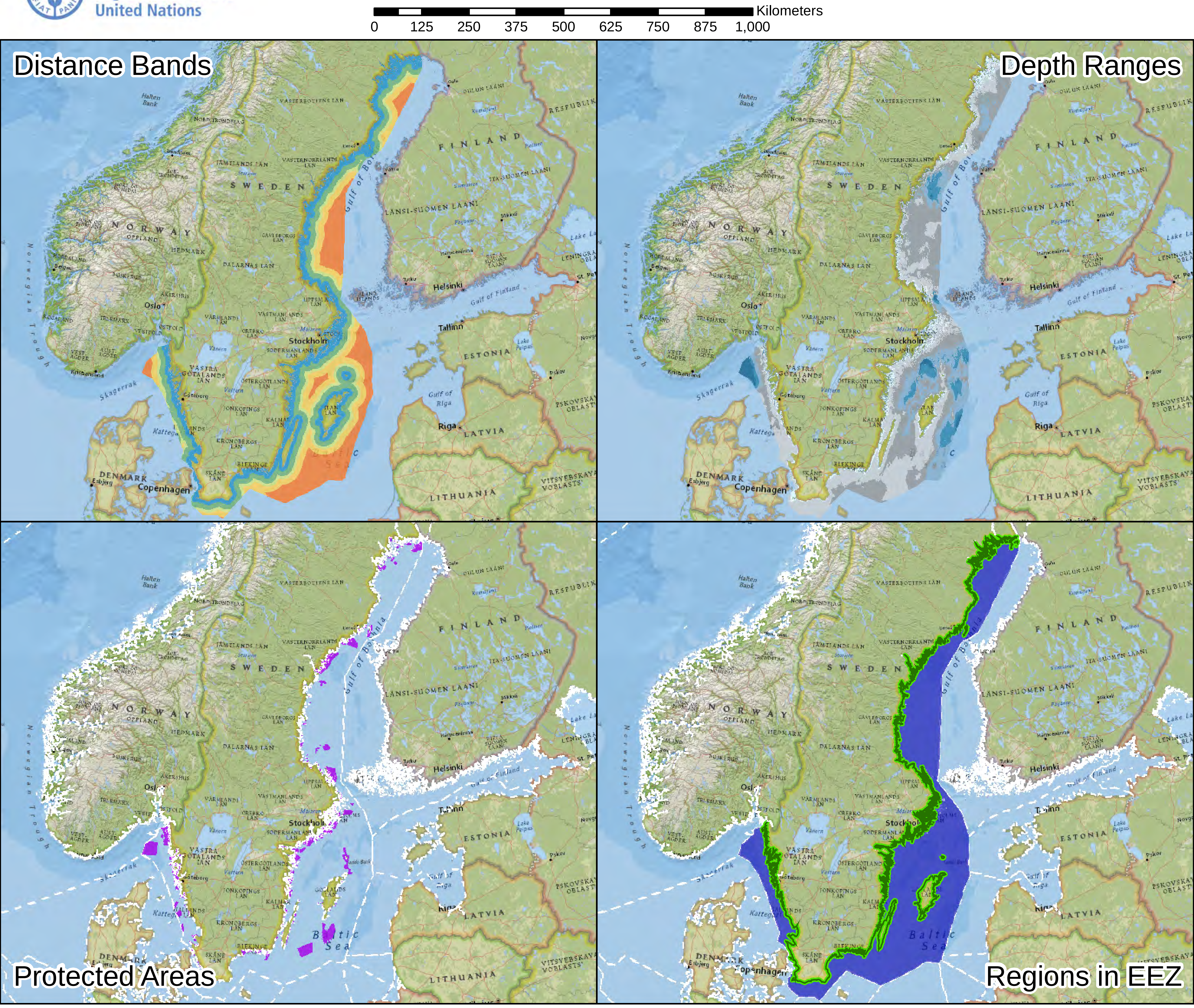

Distance Bands

- 0 to 5 NM
- 5 to 12 NM
- 12 to 16 NM
- 16 to 24 NM
- 24 to 200 NM

Depth Bands

- > -10m
- -10m to -50m
- -50m to -100m
- -100m to -150m
- -150m to -200m
- < -200m

Protected

- Protected Areas

Region

- Baltic

Coastline Length

11,952 Km

All Areas in Sweden EEZ: Cell Values = Area in Km^2 [% Depth Band (Row), % Distance Band (Column), % EEZ]

	0 to 5 NM	5 to 12 NM	12 to 16 NM	16 to 24 NM	24 to 200 NM	> 200 NM	Total
> -10m	10,073 [97%, 27%, 7%]	231 [2%, 1%, 0%]	28 [0%, 0%, 0%]	14 [0%, 0%, 0%]	23 [0%, 0%, 0%]	0 [0%, 0%, 0%]	10,369 [7% of Total]
-10m to -50m	22,444 [42%, 60%, 15%]	14,463 [27%, 41%, 9%]	4,119 [8%, 24%, 3%]	3,998 [7%, 15%, 3%]	8,373 [16%, 23%, 5%]	0 [0%, 0%, 0%]	53,398 [35% of Total]
-50m to -100m	3,948 [7%, 11%, 3%]	15,546 [29%, 45%, 10%]	8,058 [15%, 47%, 5%]	11,610 [21%, 43%, 8%]	15,043 [28%, 41%, 10%]	0 [0%, 0%, 0%]	54,205 [35% of Total]
-100m to -150m	797 [3%, 2%, 1%]	3,586 [15%, 10%, 2%]	3,470 [15%, 20%, 2%]	6,905 [29%, 26%, 5%]	8,830 [37%, 24%, 6%]	0 [0%, 0%, 0%]	23,587 [15% of Total]
-150m to -200m	151 [2%, 0%, 0%]	914 [10%, 3%, 1%]	1,238 [14%, 7%, 1%]	3,322 [38%, 12%, 2%]	3,112 [36%, 9%, 2%]	0 [0%, 0%, 0%]	8,736 [6% of Total]
<-200m	22 [1%, 0%, 0%]	169 [6%, 0%, 0%]	241 [9%, 1%, 0%]	1,078 [40%, 4%, 1%]	1,216 [45%, 3%, 1%]	0 [0%, 0%, 0%]	2,727 [2% of Total]
Total	37,435 [24% of Total]	34,909 [23% of Total]	17,154 [11% of Total]	26,927 [18% of Total]	36,597 [24% of Total]	0.000 [0% of Total]	153,022 Km^2

Areas in Sweden EEZ Excluding Protected Areas: Cell Values = Area in Km^2 [% Depth Band (Row), % Distance Band (Column), % EEZ]

12,531 [8%] Km^2 Protected	0 to 5 NM	5 to 12 NM	12 to 16 NM	16 to 24 NM	24 to 200 NM	> 200 NM	Total
> -10m	7,189 [98%, 23%, 5%]	124 [2%, 0%, 0%]	12 [0%, 0%, 0%]	0.449 [0%, 0%, 0%]	0 [0%, 0%, 0%]	0 [0%, 0%, 0%]	7,327 [5% of Total]
-10m to -50m	19,228 [42%, 63%, 14%]	13,146 [29%, 40%, 9%]	3,554 [8%, 21%, 3%]	3,146 [7%, 12%, 2%]	6,983 [15%, 20%, 5%]	0 [0%, 0%, 0%]	46,057 [33% of Total]
-50m to -100m	3,445 [6%, 11%, 2%]	15,300 [29%, 46%, 11%]	8,046 [15%, 49%, 6%]	11,609 [22%, 45%, 8%]	15,040 [28%, 44%, 11%]	0 [0%, 0%, 0%]	53,439 [38% of Total]
-100m to -150m	675 [3%, 2%, 0%]	3,578 [15%, 11%, 3%]	3,470 [15%, 21%, 2%]	6,869 [29%, 27%, 5%]	8,830 [38%, 26%, 6%]	0 [0%, 0%, 0%]	23,421 [17% of Total]
-150m to -200m	113 [1%, 0%, 0%]	911 [11%, 3%, 1%]	1,238 [14%, 7%, 1%]	3,215 [37%, 13%, 2%]	3,112 [36%, 9%, 2%]	0 [0%, 0%, 0%]	8,589 [6% of Total]
<-200m	11 [1%, 0%, 0%]	163 [10%, 0%, 0%]	241 [15%, 1%, 0%]	725 [44%, 3%, 1%]	518 [31%, 2%, 0%]	0 [0%, 0%, 0%]	1,658 [1% of Total]
Total	30,661 [22% of Total]	33,222 [24% of Total]	16,562 [12% of Total]	25,563 [18% of Total]	34,483 [25% of Total]	0.000 [0% of Total]	140,491 Km^2

The designations employed and the presentation of material in the map do not imply the expression of any opinion whatsoever on the part of FAO concerning the legal or constitutional status of any country, territory or sea area, or concerning the delimitation of frontiers.

Background reference map from National Geographic. Content may not reflect National Geographic's current map policy. Sources: National Geographic, Esri, DeLorme, HERE, UNEP-WCMC, USGS, NASA, ESA, METI, NRCAN, GEBCO, NOAA, increment P Corp.

Projection: Azimuthal Equidistant
Datum: WGS 1984
False Easting: 0.0000
False Northing: 0.0000
Central Meridian: 17.1099
Latitude Of Origin: 60.4346

Syria: Mediterranean Region

©FAO, 2018

Food and Agriculture Organization of the United Nations

Kilometers
0 125

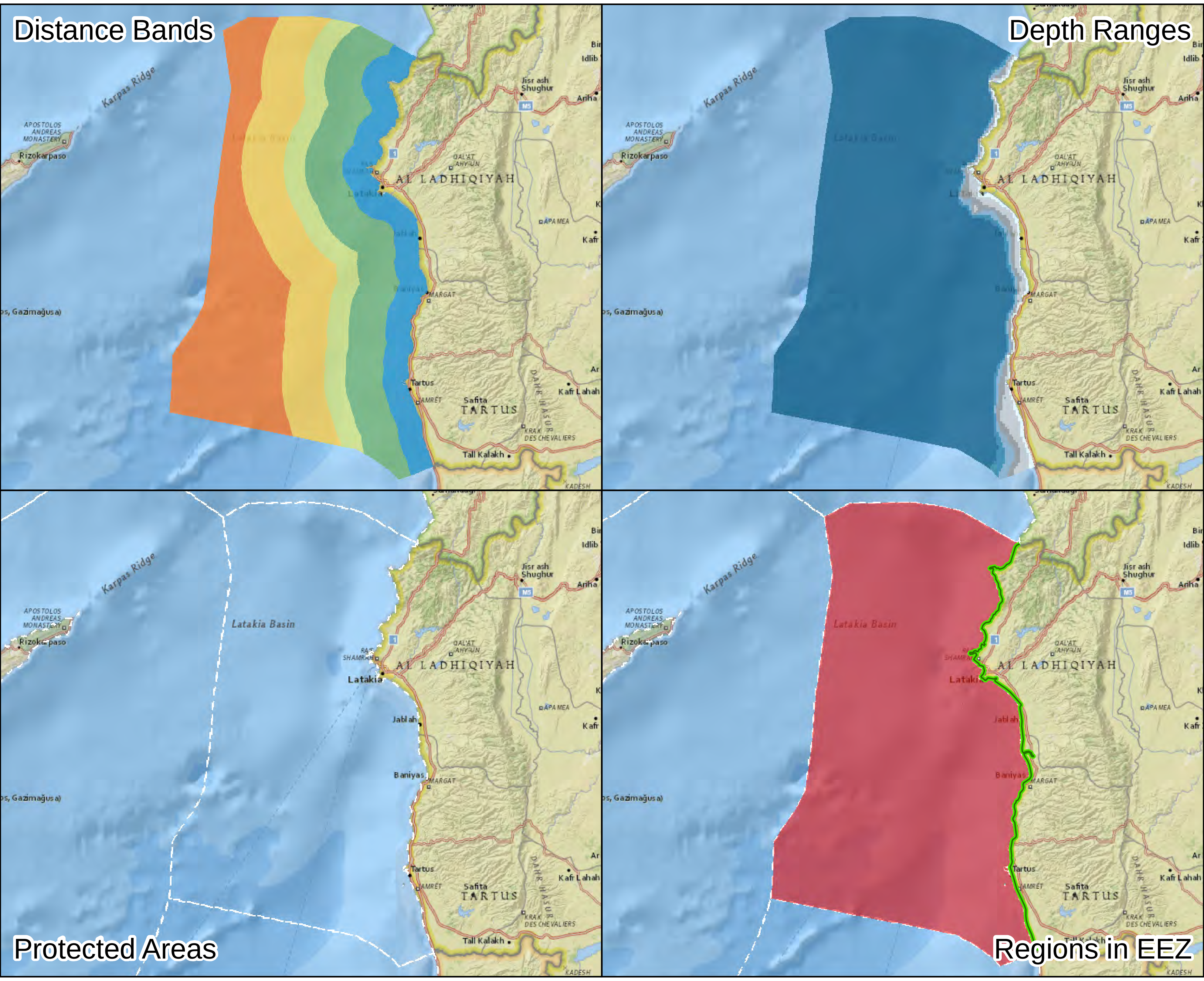

Distance Bands

- 0 to 5 NM
- 5 to 12 NM
- 12 to 16 NM
- 16 to 24 NM
- 24 to 200 NM

Depth Bands

- > -10m
- -10m to -50m
- -50m to -100m
- -100m to -150m
- -150m to -200m
- < -200m

Protected

- Protected Areas

Coastline Length

- 192 Km

Region

- Mediterranean

All Areas in Syria EEZ: Cell Values = Area in Km^2 [% Depth Band (Row), % Distance Band (Column), % EEZ]

	0 to 5 NM	5 to 12 NM	12 to 16 NM	16 to 24 NM	24 to 200 NM	> 200 NM	Total
> -10m	105 [100%, 7%, 1%]	0 [0%, 0%, 0%]	0 [0%, 0%, 0%]	0 [0%, 0%, 0%]	0 [0%, 0%, 0%]	0 [0%, 0%, 0%]	105 [1% of Total]
-10m to -50m	243 [100%, 16%, 2%]	0 [0%, 0%, 0%]	0 [0%, 0%, 0%]	0 [0%, 0%, 0%]	0 [0%, 0%, 0%]	0 [0%, 0%, 0%]	243 [2% of Total]
-50m to -100m	206 [98%, 14%, 2%]	3 [2%, 0%, 0%]	0 [0%, 0%, 0%]	0 [0%, 0%, 0%]	0 [0%, 0%, 0%]	0 [0%, 0%, 0%]	209 [2% of Total]
-100m to -150m	143 [87%, 10%, 1%]	21 [13%, 1%, 0%]	0 [0%, 0%, 0%]	0 [0%, 0%, 0%]	0 [0%, 0%, 0%]	0 [0%, 0%, 0%]	164 [2% of Total]
-150m to -200m	95 [70%, 6%, 1%]	41 [30%, 2%, 0%]	0 [0%, 0%, 0%]	0 [0%, 0%, 0%]	0 [0%, 0%, 0%]	0 [0%, 0%, 0%]	137 [1% of Total]
<-200m	707 [8%, 47%, 7%]	2,152 [23%, 97%, 21%]	1,209 [13%, 100%, 12%]	2,349 [25%, 100%, 23%]	2,992 [32%, 100%, 29%]	0 [0%, 0%, 0%]	9,409 [92% of Total]
Total	1,500 [15% of Total]	2,217 [22% of Total]	1,209 [12% of Total]	2,349 [23% of Total]	2,992 [29% of Total]	0.000 [0% of Total]	10,267 Km^2

Areas in Syria EEZ Excluding Protected Areas: Cell Values = Area in Km^2 [% Depth Band (Row), % Distance Band (Column), % EEZ]

0 [0%] Km^2 Protected	0 to 5 NM	5 to 12 NM	12 to 16 NM	16 to 24 NM	24 to 200 NM	> 200 NM	Total
> -10m	105 [100%, 7%, 1%]	0 [0%, 0%, 0%]	0 [0%, 0%, 0%]	0 [0%, 0%, 0%]	0 [0%, 0%, 0%]	0 [0%, 0%, 0%]	105 [1% of Total]
-10m to -50m	243 [100%, 16%, 2%]	0 [0%, 0%, 0%]	0 [0%, 0%, 0%]	0 [0%, 0%, 0%]	0 [0%, 0%, 0%]	0 [0%, 0%, 0%]	243 [2% of Total]
-50m to -100m	206 [98%, 14%, 2%]	3 [2%, 0%, 0%]	0 [0%, 0%, 0%]	0 [0%, 0%, 0%]	0 [0%, 0%, 0%]	0 [0%, 0%, 0%]	209 [2% of Total]
-100m to -150m	143 [87%, 10%, 1%]	21 [13%, 1%, 0%]	0 [0%, 0%, 0%]	0 [0%, 0%, 0%]	0 [0%, 0%, 0%]	0 [0%, 0%, 0%]	164 [2% of Total]
-150m to -200m	95 [70%, 6%, 1%]	41 [30%, 2%, 0%]	0 [0%, 0%, 0%]	0 [0%, 0%, 0%]	0 [0%, 0%, 0%]	0 [0%, 0%, 0%]	137 [1% of Total]
<-200m	707 [8%, 47%, 7%]	2,152 [23%, 97%, 21%]	1,209 [13%, 100%, 12%]	2,349 [25%, 100%, 23%]	2,992 [32%, 100%, 29%]	0 [0%, 0%, 0%]	9,409 [92% of Total]
Total	1,500 [15% of Total]	2,217 [22% of Total]	1,209 [12% of Total]	2,349 [23% of Total]	2,992 [29% of Total]	0.000 [0% of Total]	10,267 Km^2

The designations employed and the presentation of material in the map do not imply the expression of any opinion whatsoever on the part of FAO concerning the legal or constitutional status of any country, territory or sea area, or concerning the delimitation of frontiers.

Background reference map from National Geographic. Content may not reflect National Geographic's current map policy. Sources: National Geographic, Esri, DeLorme, HERE, UNEP-WCMC, USGS, NASA, ESA, METI, NRCAN, GEBCO, NOAA, increment P Corp.

Projection: Azimuthal Equidistant
Datum: WGS 1984
False Easting: 0.0000
False Northing: 0.0000
Central Meridian: 35.4679
Latitude Of Origin: 35.3309

Trinidad and Tobago: Caribbean Region

Food and Agriculture Organization of the United Nations

©FAO, 2018

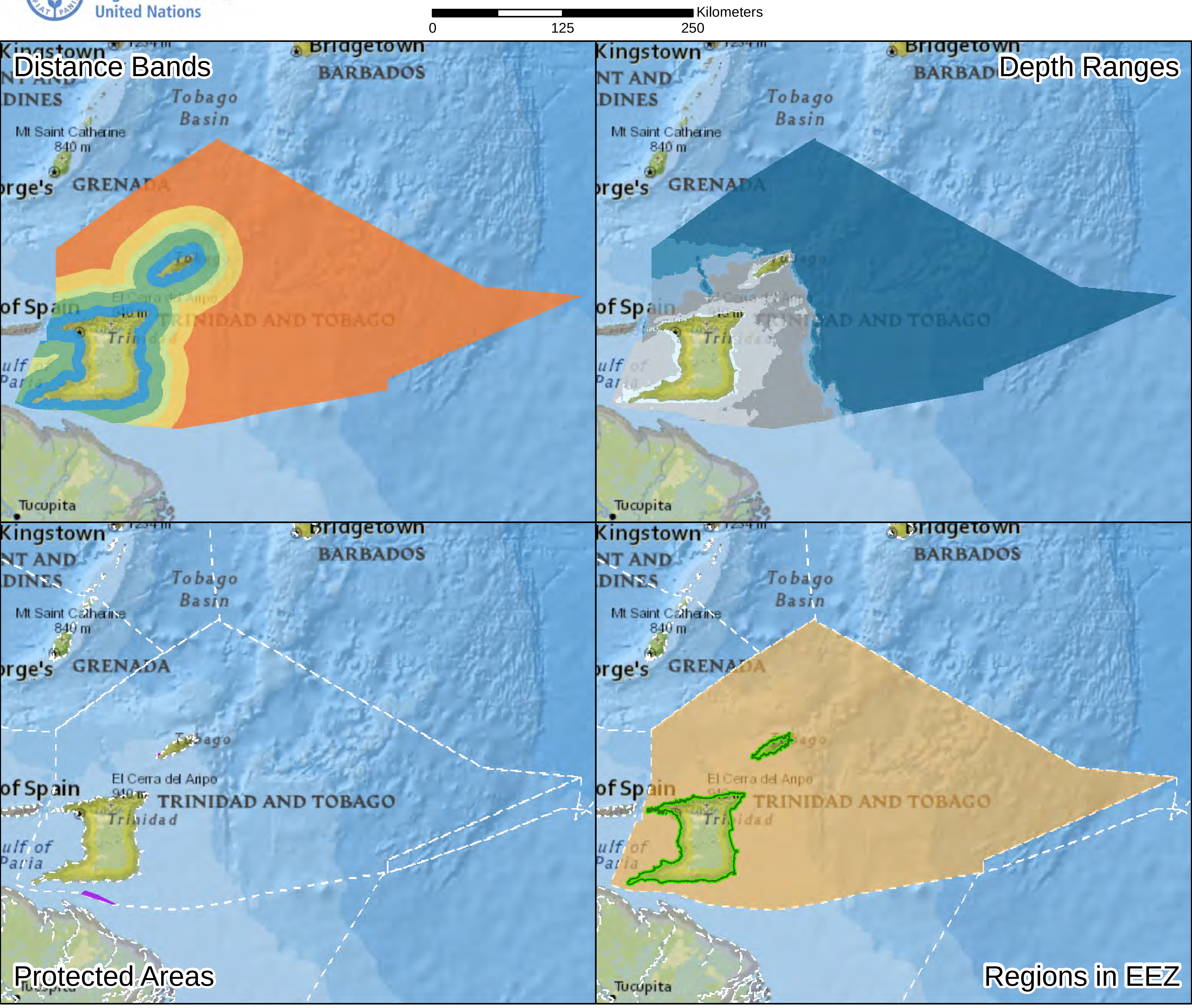

Distance Bands

- 0 to 5 NM
- 5 to 12 NM
- 12 to 16 NM
- 16 to 24 NM
- 24 to 200 NM

Depth Bands

- > -10m
- -10m to -50m
- -50m to -100m
- -100m to -150m
- -150m to -200m
- < -200m

Protected

- Protected Areas

Region

- Caribbean

Coastline Length

797 Km

All Areas in Trinidad and Tobago EEZ: Cell Values = Area in Km^2 [% Depth Band (Row), % Distance Band (Column), % EEZ]

	0 to 5 NM	5 to 12 NM	12 to 16 NM	16 to 24 NM	24 to 200 NM	> 200 NM	Total
> -10m	1,221 [97%, 22%, 2%]	32 [3%, 0%, 0%]	0 [0%, 0%, 0%]	0 [0%, 0%, 0%]	2 [0%, 0%, 0%]	0 [0%, 0%, 0%]	1,254 [2% of Total]
-10m to -50m	2,976 [38%, 53%, 4%]	3,230 [41%, 45%, 4%]	985 [13%, 28%, 1%]	599 [8%, 9%, 1%]	57 [1%, 0%, 0%]	0 [0%, 0%, 0%]	7,846 [10% of Total]
-50m to -100m	871 [10%, 16%, 1%]	2,094 [24%, 29%, 3%]	813 [9%, 24%, 1%]	1,728 [20%, 27%, 2%]	3,106 [36%, 6%, 4%]	0 [0%, 0%, 0%]	8,612 [11% of Total]
-100m to -150m	136 [4%, 2%, 0%]	620 [19%, 9%, 1%]	553 [17%, 16%, 1%]	967 [30%, 15%, 1%]	944 [29%, 2%, 1%]	0 [0%, 0%, 0%]	3,220 [4% of Total]
-150m to -200m	68 [3%, 1%, 0%]	136 [5%, 2%, 0%]	155 [6%, 4%, 0%]	319 [12%, 5%, 0%]	1,913 [74%, 4%, 2%]	0 [0%, 0%, 0%]	2,591 [3% of Total]
<-200m	324 [1%, 6%, 0%]	1,121 [2%, 15%, 1%]	951 [2%, 28%, 1%]	2,720 [5%, 43%, 4%]	48,224 [90%, 89%, 63%]	0 [0%, 0%, 0%]	53,340 [69% of Total]
Total	5,596 [7% of Total]	7,232 [9% of Total]	3,456 [4% of Total]	6,333 [8% of Total]	54,246 [71% of Total]	0.000 [0% of Total]	76,863 Km^2

Areas in Trinidad and Tobago EEZ Excluding Protected Areas: Cell Values = Area in Km^2 [% Depth Band (Row), % Distance Band (Column), % EEZ]

131 [0%] Km^2 Protected	0 to 5 NM	5 to 12 NM	12 to 16 NM	16 to 24 NM	24 to 200 NM	> 200 NM	Total
> -10m	1,205 [97%, 22%, 2%]	32 [3%, 0%, 0%]	0 [0%, 0%, 0%]	0 [0%, 0%, 0%]	2 [0%, 0%, 0%]	0 [0%, 0%, 0%]	1,239 [2% of Total]
-10m to -50m	2,970 [38%, 53%, 4%]	3,129 [40%, 44%, 4%]	985 [13%, 28%, 1%]	599 [8%, 9%, 1%]	57 [1%, 0%, 0%]	0 [0%, 0%, 0%]	7,740 [10% of Total]
-50m to -100m	871 [10%, 16%, 1%]	2,084 [24%, 29%, 3%]	813 [9%, 24%, 1%]	1,728 [20%, 27%, 2%]	3,106 [36%, 6%, 4%]	0 [0%, 0%, 0%]	8,602 [11% of Total]
-100m to -150m	136 [4%, 2%, 0%]	620 [19%, 9%, 1%]	553 [17%, 16%, 1%]	967 [30%, 15%, 1%]	944 [29%, 2%, 1%]	0 [0%, 0%, 0%]	3,220 [4% of Total]
-150m to -200m	68 [3%, 1%, 0%]	136 [5%, 2%, 0%]	155 [6%, 4%, 0%]	319 [12%, 5%, 0%]	1,913 [74%, 4%, 2%]	0 [0%, 0%, 0%]	2,591 [3% of Total]
<-200m	324 [1%, 6%, 0%]	1,121 [2%, 16%, 1%]	951 [2%, 28%, 1%]	2,720 [5%, 43%, 4%]	48,224 [90%, 89%, 63%]	0 [0%, 0%, 0%]	53,340 [70% of Total]
Total	5,575 [7% of Total]	7,122 [9% of Total]	3,456 [5% of Total]	6,333 [8% of Total]	54,246 [71% of Total]	0.000 [0% of Total]	76,732 Km^2

The designations employed and the presentation of material in the map do not imply the expression of any opinion whatsoever on the part of FAO concerning the legal or constitutional status of any country, territory or sea area, or concerning the delimitation of frontiers.

Background reference map from National Geographic. Content may not reflect National Geographic's current map policy. Sources: National Geographic, Esri, DeLorme, HERE, UNEP-WCMC, USGS, NASA, ESA, METI, NRCAN, GEBCO, NOAA, increment P Corp.

Projection: Azimuthal Equidistant
Datum: WGS 1984
False Easting: 0.0000
False Northing: 0.0000
Central Meridian: -59.6003
Latitude Of Origin: 11.0933

Food and Agriculture Organization of the United Nations

Tunisia: Mediterranean Region

©FAO, 2018

Kilometers: 0, 125, 250, 375

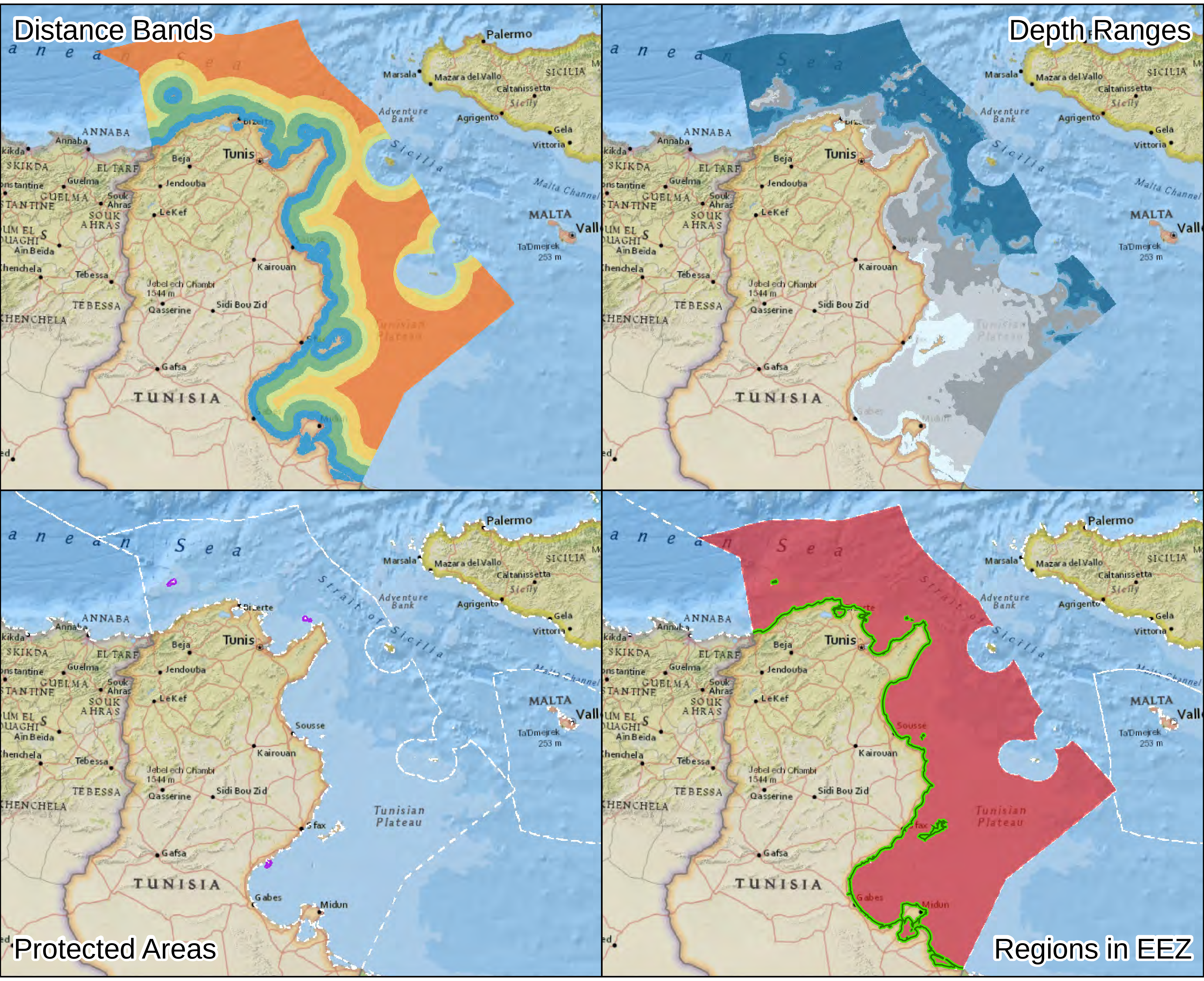

Distance Bands

- 0 to 5 NM
- 5 to 12 NM
- 12 to 16 NM
- 16 to 24 NM
- 24 to 200 NM

Depth Bands

- > -10m
- -10m to -50m
- -50m to -100m
- -100m to -150m
- -150m to -200m
- < -200m

Protected

- Protected Areas

Coastline Length

1,593 Km

Region

- Mediterranean

All Areas in Tunisia EEZ: Cell Values = Area in Km^2 [% Depth Band (Row), % Distance Band (Column), % EEZ]

	0 to 5 NM	5 to 12 NM	12 to 16 NM	16 to 24 NM	24 to 200 NM	> 200 NM	Total
> -10m	4,680 [70%, 37%, 5%]	1,546 [23%, 10%, 2%]	298 [4%, 3%, 0%]	165 [2%, 1%, 0%]	0 [0%, 0%, 0%]	0 [0%, 0%, 0%]	6,688 [7% of Total]
-10m to -50m	5,450 [20%, 43%, 5%]	5,750 [21%, 37%, 6%]	3,270 [12%, 34%, 3%]	5,491 [20%, 29%, 6%]	6,868 [26%, 16%, 7%]	0 [0%, 0%, 0%]	26,828 [27% of Total]
-50m to -100m	1,922 [9%, 15%, 2%]	4,020 [19%, 26%, 4%]	1,836 [9%, 19%, 2%]	3,084 [15%, 16%, 3%]	9,780 [47%, 23%, 10%]	0 [0%, 0%, 0%]	20,642 [21% of Total]
-100m to -150m	464 [6%, 4%, 0%]	1,667 [22%, 11%, 2%]	649 [8%, 7%, 1%]	1,547 [20%, 8%, 2%]	3,401 [44%, 8%, 3%]	0 [0%, 0%, 0%]	7,728 [8% of Total]
-150m to -200m	15 [0%, 0%, 0%]	1,245 [23%, 8%, 1%]	638 [12%, 7%, 1%]	894 [17%, 5%, 1%]	2,580 [48%, 6%, 3%]	0 [0%, 0%, 0%]	5,372 [5% of Total]
<-200m	0 [0%, 0%, 0%]	1,270 [4%, 8%, 1%]	2,972 [9%, 31%, 3%]	7,626 [24%, 41%, 8%]	20,129 [63%, 47%, 20%]	0 [0%, 0%, 0%]	31,998 [32% of Total]
Total	12,530 [13% of Total]	15,499 [16% of Total]	9,662 [10% of Total]	18,807 [19% of Total]	42,757 [43% of Total]	0.000 [0% of Total]	99,255 Km^2

Areas in Tunisia EEZ Excluding Protected Areas: Cell Values = Area in Km^2 [% Depth Band (Row), % Distance Band (Column), % EEZ]

225 [0%] Km^2 Protected	0 to 5 NM	5 to 12 NM	12 to 16 NM	16 to 24 NM	24 to 200 NM	> 200 NM	Total
> -10m	4,600 [70%, 37%, 5%]	1,546 [23%, 10%, 2%]	298 [5%, 3%, 0%]	165 [2%, 1%, 0%]	0 [0%, 0%, 0%]	0 [0%, 0%, 0%]	6,609 [7% of Total]
-10m to -50m	5,347 [20%, 43%, 5%]	5,750 [22%, 37%, 6%]	3,270 [12%, 34%, 3%]	5,491 [21%, 29%, 6%]	6,868 [26%, 16%, 7%]	0 [0%, 0%, 0%]	26,726 [27% of Total]
-50m to -100m	1,883 [9%, 15%, 2%]	4,020 [20%, 26%, 4%]	1,836 [9%, 19%, 2%]	3,084 [15%, 16%, 3%]	9,780 [47%, 23%, 10%]	0 [0%, 0%, 0%]	20,603 [21% of Total]
-100m to -150m	459 [6%, 4%, 0%]	1,667 [22%, 11%, 2%]	649 [8%, 7%, 1%]	1,547 [20%, 8%, 2%]	3,401 [44%, 8%, 3%]	0 [0%, 0%, 0%]	7,722 [8% of Total]
-150m to -200m	15 [0%, 0%, 0%]	1,245 [23%, 8%, 1%]	638 [12%, 7%, 1%]	894 [17%, 5%, 1%]	2,580 [48%, 6%, 3%]	0 [0%, 0%, 0%]	5,372 [5% of Total]
<-200m	0 [0%, 0%, 0%]	1,270 [4%, 8%, 1%]	2,972 [9%, 31%, 3%]	7,626 [24%, 41%, 8%]	20,129 [63%, 47%, 20%]	0 [0%, 0%, 0%]	31,998 [32% of Total]
Total	12,305 [12% of Total]	15,499 [16% of Total]	9,662 [10% of Total]	18,807 [19% of Total]	42,757 [43% of Total]	0.000 [0% of Total]	99,030 Km^2

The designations employed and the presentation of material in the map do not imply the expression of any opinion whatsoever on the part of FAO concerning the legal or constitutional status of any country, territory or sea area, or concerning the delimitation of frontiers.

Background reference map from National Geographic. Content may not reflect National Geographic's current map policy. Sources: National Geographic, Esri, DeLorme, HERE, UNEP-WCMC, USGS, NASA, ESA, METI, NRCAN, GEBCO, NOAA, increment P Corp.

Projection: Azimuthal Equidistant
Datum: WGS 1984
False Easting: 0.0000
False Northing: 0.0000
Central Meridian: 10.7503
Latitude Of Origin: 35.7887

Food and Agriculture Organization of the United Nations

Turkey: Mediterranean and Black Sea Regions

©FAO, 2018

Kilometers: 0, 125, 250, 375, 500, 625, 750, 875, 1,000

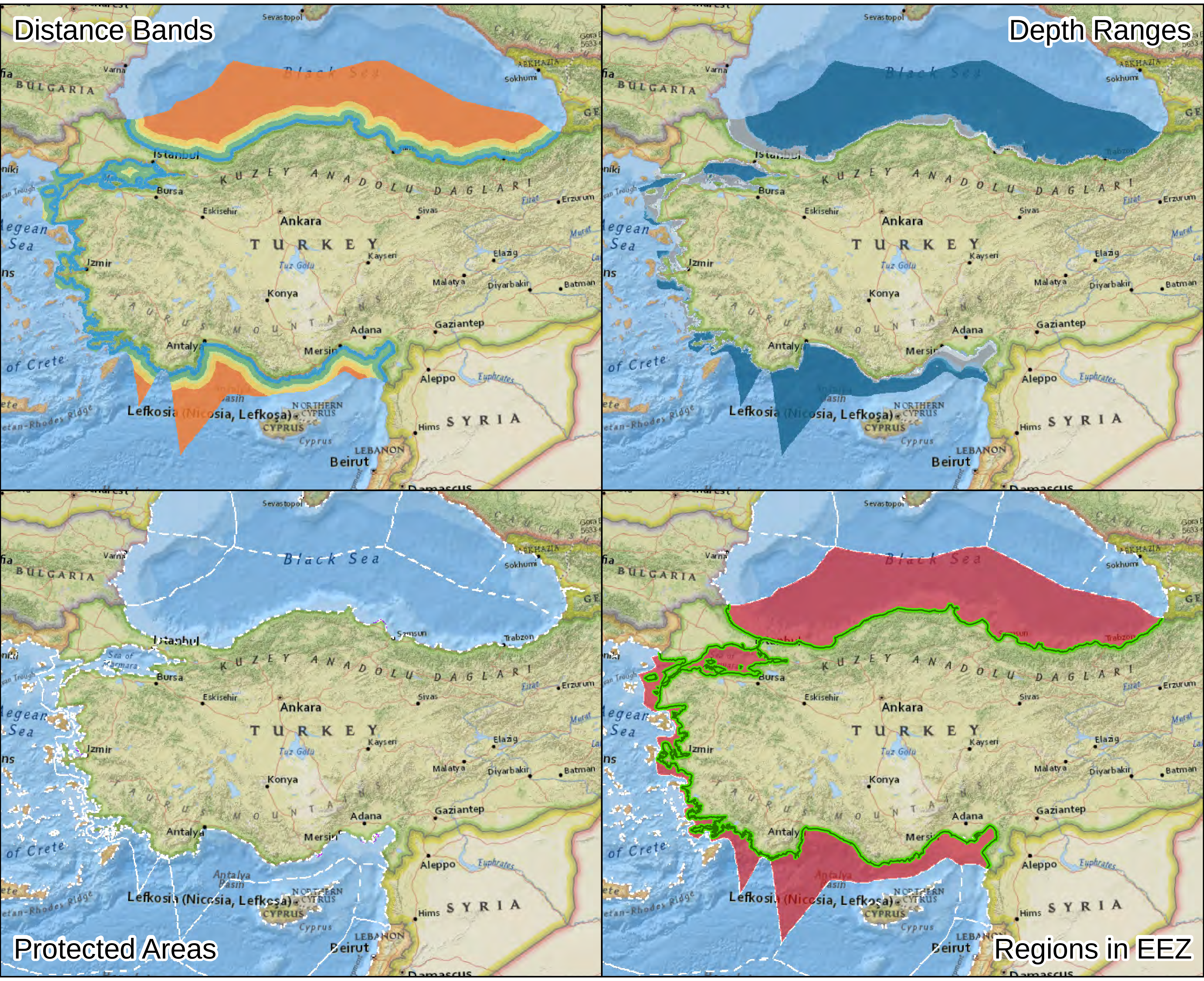

Distance Bands

- 0 to 5 NM
- 5 to 12 NM
- 12 to 16 NM
- 16 to 24 NM
- 24 to 200 NM

Depth Bands

- > -10m
- -10m to -50m
- -50m to -100m
- -100m to -150m
- -150m to -200m
- < -200m

Protected

- Protected Areas

Region

- Mediterranean

Coastline Length

6,640 Km

All Areas in Turkey EEZ: Cell Values = Area in Km^2 [% Depth Band (Row), % Distance Band (Column), % EEZ]

	0 to 5 NM	5 to 12 NM	12 to 16 NM	16 to 24 NM	24 to 200 NM	> 200 NM	Total
> -10m	3,532 [100%, 8%, 1%]	0.409 [0%, 0%, 0%]	0 [0%, 0%, 0%]	0 [0%, 0%, 0%]	0 [0%, 0%, 0%]	0 [0%, 0%, 0%]	3,532 [1% of Total]
-10m to -50m	11,273 [95%, 26%, 4%]	636 [5%, 2%, 0%]	0.123 [0%, 0%, 0%]	1 [0%, 0%, 0%]	0 [0%, 0%, 0%]	0 [0%, 0%, 0%]	11,910 [5% of Total]
-50m to -100m	13,730 [59%, 32%, 5%]	7,780 [33%, 21%, 3%]	1,136 [5%, 7%, 0%]	624 [3%, 2%, 0%]	0 [0%, 0%, 0%]	0 [0%, 0%, 0%]	23,270 [9% of Total]
-100m to -150m	3,003 [42%, 7%, 1%]	2,477 [34%, 7%, 1%]	882 [12%, 5%, 0%]	821 [11%, 3%, 0%]	30 [0%, 0%, 0%]	0 [0%, 0%, 0%]	7,213 [3% of Total]
-150m to -200m	1,882 [47%, 4%, 1%]	1,066 [26%, 3%, 0%]	459 [11%, 3%, 0%]	411 [10%, 1%, 0%]	206 [5%, 0%, 0%]	0 [0%, 0%, 0%]	4,024 [2% of Total]
<-200m	9,738 [5%, 23%, 4%]	24,456 [12%, 67%, 9%]	13,637 [6%, 85%, 5%]	25,815 [12%, 93%, 10%]	136,791 [65%, 100%, 53%]	0 [0%, 0%, 0%]	210,436 [81% of Total]
Total	43,157 [17% of Total]	36,415 [14% of Total]	16,114 [6% of Total]	27,672 [11% of Total]	137,027 [53% of Total]	0.000 [0% of Total]	260,385 Km^2

Areas in Turkey EEZ Excluding Protected Areas: Cell Values = Area in Km^2 [% Depth Band (Row), % Distance Band (Column), % EEZ]

260 [0%] Km^2 Protected	0 to 5 NM	5 to 12 NM	12 to 16 NM	16 to 24 NM	24 to 200 NM	> 200 NM	Total
> -10m	3,326 [100%, 8%, 1%]	0.409 [0%, 0%, 0%]	0 [0%, 0%, 0%]	0 [0%, 0%, 0%]	0 [0%, 0%, 0%]	0 [0%, 0%, 0%]	3,327 [1% of Total]
-10m to -50m	11,221 [95%, 26%, 4%]	636 [5%, 2%, 0%]	0.123 [0%, 0%, 0%]	1 [0%, 0%, 0%]	0 [0%, 0%, 0%]	0 [0%, 0%, 0%]	11,859 [5% of Total]
-50m to -100m	13,727 [59%, 32%, 5%]	7,780 [33%, 21%, 3%]	1,136 [5%, 7%, 0%]	624 [3%, 2%, 0%]	0 [0%, 0%, 0%]	0 [0%, 0%, 0%]	23,267 [9% of Total]
-100m to -150m	3,003 [42%, 7%, 1%]	2,477 [34%, 7%, 1%]	882 [12%, 5%, 0%]	821 [11%, 3%, 0%]	30 [0%, 0%, 0%]	0 [0%, 0%, 0%]	7,213 [3% of Total]
-150m to -200m	1,882 [47%, 4%, 1%]	1,066 [26%, 3%, 0%]	459 [11%, 3%, 0%]	411 [10%, 1%, 0%]	206 [5%, 0%, 0%]	0 [0%, 0%, 0%]	4,024 [2% of Total]
<-200m	9,738 [5%, 23%, 4%]	24,456 [12%, 67%, 9%]	13,637 [6%, 85%, 5%]	25,815 [12%, 93%, 10%]	136,791 [65%, 100%, 53%]	0 [0%, 0%, 0%]	210,436 [81% of Total]
Total	42,896 [16% of Total]	36,415 [14% of Total]	16,114 [6% of Total]	27,672 [11% of Total]	137,027 [53% of Total]	0.000 [0% of Total]	260,125 Km^2

The designations employed and the presentation of material in the map do not imply the expression of any opinion whatsoever on the part of FAO concerning the legal or constitutional status of any country, territory or sea area, or concerning the delimitation of frontiers.

Background reference map from National Geographic. Content may not reflect National Geographic's current map policy. Sources: National Geographic, Esri, DeLorme, HERE, UNEP-WCMC, USGS, NASA, ESA, METI, NRCAN, GEBCO, NOAA, increment P Corp.

Projection: Azimuthal Equidistant
Datum: WGS 1984
False Easting: 0.0000
False Northing: 0.0000
Central Meridian: 33.4937
Latitude Of Origin: 38.8295

N W E S

Ukraine: Black Sea Region

©FAO, 2018

Food and Agriculture Organization of the United Nations

Kilometers: 0 125 250 375

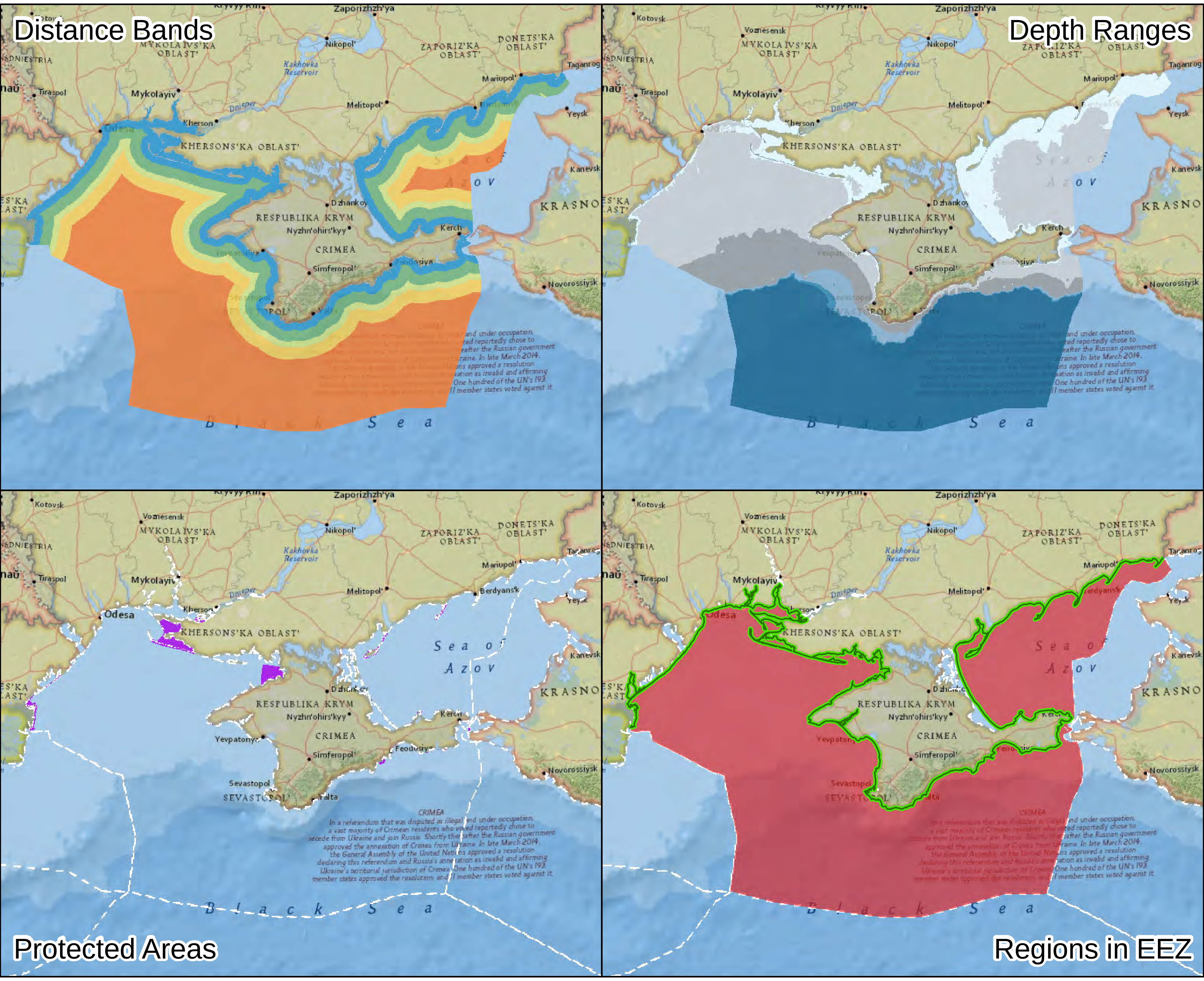

Distance Bands

- 0 to 5 NM
- 5 to 12 NM
- 12 to 16 NM
- 16 to 24 NM
- 24 to 200 NM

Depth Bands

- > -10m
- -10m to -50m
- -50m to -100m
- -100m to -150m
- -150m to -200m
- < -200m

Protected

- Protected Areas

Region

- Mediterranean

Coastline Length

3,083 Km

All Areas in Ukraine EEZ: Cell Values = Area in Km^2 [% Depth Band (Row), % Distance Band (Column), % EEZ]

	0 to 5 NM	5 to 12 NM	12 to 16 NM	16 to 24 NM	24 to 200 NM	> 200 NM	Total
> -10m	10,211 [68%, 54%, 8%]	4,020 [27%, 21%, 3%]	589 [4%, 6%, 0%]	241 [2%, 1%, 0%]	2 [0%, 0%, 0%]	0 [0%, 0%, 0%]	15,062 [11% of Total]
-10m to -50m	7,078 [17%, 37%, 5%]	9,682 [23%, 51%, 7%]	5,043 [12%, 55%, 4%]	8,661 [21%, 53%, 6%]	11,135 [27%, 16%, 8%]	0 [0%, 0%, 0%]	41,599 [31% of Total]
-50m to -100m	1,391 [10%, 7%, 1%]	2,707 [20%, 14%, 2%]	1,448 [11%, 16%, 1%]	1,847 [13%, 11%, 1%]	6,323 [46%, 9%, 5%]	0 [0%, 0%, 0%]	13,717 [10% of Total]
-100m to -150m	126 [4%, 1%, 0%]	1,056 [31%, 6%, 1%]	457 [14%, 5%, 0%]	983 [29%, 6%, 1%]	746 [22%, 1%, 1%]	0 [0%, 0%, 0%]	3,369 [3% of Total]
-150m to -200m	24 [3%, 0%, 0%]	101 [11%, 1%, 0%]	155 [17%, 2%, 0%]	211 [24%, 1%, 0%]	393 [44%, 1%, 0%]	0 [0%, 0%, 0%]	884 [1% of Total]
<-200m	116 [0%, 1%, 0%]	1,512 [3%, 8%, 1%]	1,552 [3%, 17%, 1%]	4,521 [8%, 27%, 3%]	51,000 [87%, 73%, 38%]	0 [0%, 0%, 0%]	58,700 [44% of Total]
Total	18,946 [14% of Total]	19,077 [14% of Total]	9,243 [7% of Total]	16,464 [12% of Total]	69,600 [52% of Total]	0.000 [0% of Total]	133,330 Km^2

Areas in Ukraine EEZ Excluding Protected Areas: Cell Values = Area in Km^2 [% Depth Band (Row), % Distance Band (Column), % EEZ]

1,412 [1%] Km^2 Protected	0 to 5 NM	5 to 12 NM	12 to 16 NM	16 to 24 NM	24 to 200 NM	> 200 NM	Total
> -10m	8,867 [65%, 50%, 7%]	3,985 [29%, 21%, 3%]	589 [4%, 6%, 0%]	241 [2%, 1%, 0%]	2 [0%, 0%, 0%]	0 [0%, 0%, 0%]	13,682 [10% of Total]
-10m to -50m	7,046 [17%, 40%, 5%]	9,682 [23%, 51%, 7%]	5,043 [12%, 55%, 4%]	8,661 [21%, 53%, 7%]	11,135 [27%, 16%, 8%]	0 [0%, 0%, 0%]	41,567 [32% of Total]
-50m to -100m	1,391 [10%, 8%, 1%]	2,707 [20%, 14%, 2%]	1,448 [11%, 16%, 1%]	1,847 [13%, 11%, 1%]	6,323 [46%, 9%, 5%]	0 [0%, 0%, 0%]	13,716 [10% of Total]
-100m to -150m	126 [4%, 1%, 0%]	1,056 [31%, 6%, 1%]	457 [14%, 5%, 0%]	983 [29%, 6%, 1%]	746 [22%, 1%, 1%]	0 [0%, 0%, 0%]	3,369 [3% of Total]
-150m to -200m	24 [3%, 0%, 0%]	101 [11%, 1%, 0%]	155 [17%, 2%, 0%]	211 [24%, 1%, 0%]	393 [44%, 1%, 0%]	0 [0%, 0%, 0%]	884 [1% of Total]
<-200m	116 [0%, 1%, 0%]	1,512 [3%, 8%, 1%]	1,552 [3%, 17%, 1%]	4,521 [8%, 27%, 3%]	51,000 [87%, 73%, 39%]	0 [0%, 0%, 0%]	58,700 [44% of Total]
Total	17,569 [13% of Total]	19,042 [14% of Total]	9,243 [7% of Total]	16,464 [12% of Total]	69,600 [53% of Total]	0.000 [0% of Total]	131,919 Km^2

The designations employed and the presentation of material in the map do not imply the expression of any opinion whatsoever on the part of FAO concerning the legal or constitutional status of any country, territory or sea area, or concerning the delimitation of frontiers.

Background reference map from National Geographic. Content may not reflect National Geographic's current map policy. Sources: National Geographic, Esri, DeLorme, HERE, UNEP-WCMC, USGS, NASA, ESA, METI, NRCAN, GEBCO, NOAA, increment P Corp.

Projection: Azimuthal Equidistant
Datum: WGS 1984
False Easting: 0.0000
False Northing: 0.0000
Central Meridian: 33.9756
Latitude Of Origin: 45.1516

Food and Agriculture Organization of the United Nations

United Kingdom: Atlantic, North Sea and Mediterranean Regions

©FAO, 2018

Kilometers
0 250 500 750 1,000 1,250 1,500 1,750 2,000 2,250

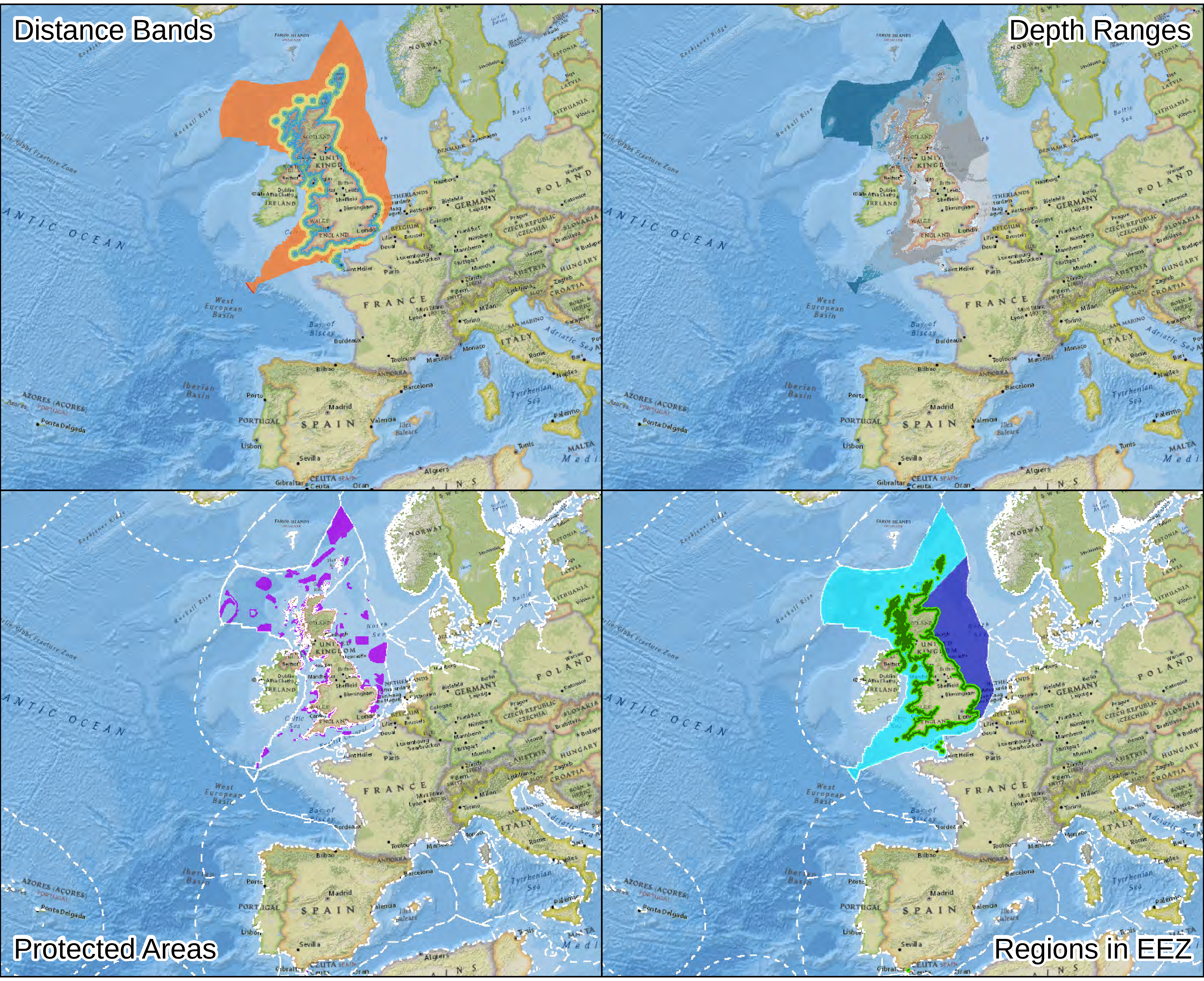

Distance Bands
- 0 to 5 NM
- 5 to 12 NM
- 12 to 16 NM
- 16 to 24 NM
- 24 to 200 NM
- > 200 NM

Depth Bands
- > -10m
- -10m to -50m
- -50m to -100m
- -100m to -150m
- -150m to -200m
- < -200m

Protected
- Protected Areas

Coastline Length
- 30,251 Km

Regions
- Mediterranean
- Baltic
- Atlantic

All Areas in United Kingdom EEZ: Cell Values = Area in Km² [% Depth Band (Row), % Distance Band (Column), % EEZ]

	0 to 5 NM	5 to 12 NM	12 to 16 NM	16 to 24 NM	24 to 200 NM	> 200 NM	Total
> -10m	12,311 [91%, 16%, 2%]	964 [7%, 1%, 0%]	172 [1%, 0%, 0%]	33 [0%, 0%, 0%]	29 [0%, 0%, 0%]	0 [0%, 0%, 0%]	13,510 [2% of Total]
-10m to -50m	37,534 [31%, 50%, 5%]	24,403 [20%, 31%, 3%]	7,875 [7%, 20%, 1%]	10,060 [8%, 16%, 1%]	39,971 [33%, 8%, 5%]	0 [0%, 0%, 0%]	119,843 [16% of Total]
-50m to -100m	19,775 [9%, 26%, 3%]	38,986 [18%, 49%, 5%]	19,867 [9%, 51%, 3%]	32,434 [15%, 50%, 4%]	103,828 [48%, 22%, 14%]	0 [0%, 0%, 0%]	214,891 [29% of Total]
-100m to -150m	4,993 [3%, 7%, 1%]	13,562 [9%, 17%, 2%]	9,360 [6%, 24%, 1%]	17,072 [11%, 27%, 2%]	103,528 [70%, 22%, 14%]	0 [0%, 0%, 0%]	148,515 [20% of Total]
-150m to -200m	766 [2%, 1%, 0%]	1,325 [4%, 2%, 0%]	1,298 [4%, 3%, 0%]	3,937 [11%, 6%, 1%]	29,081 [80%, 6%, 4%]	0.521 [0%, 0%, 0%]	36,407 [5% of Total]
<-200m	211 [0%, 0%, 0%]	346 [0%, 0%, 0%]	53 [0%, 0%, 0%]	842 [0%, 1%, 0%]	201,708 [99%, 42%, 27%]	612 [0%, 100%, 0%]	203,772 [28% of Total]
Total	75,590 [10% of Total]	79,586 [11% of Total]	38,627 [5% of Total]	64,378 [9% of Total]	478,145 [65% of Total]	613 [0% of Total]	736,939 Km²

Areas in United Kingdom EEZ Excluding Protected Areas: Cell Values = Area in Km² [% Depth Band (Row), % Distance Band (Column), % EEZ]

128,480 [17%] Km² Protected	0 to 5 NM	5 to 12 NM	12 to 16 NM	16 to 24 NM	24 to 200 NM	> 200 NM	Total
> -10m	4,431 [91%, 8%, 1%]	403 [8%, 1%, 0%]	48 [1%, 0%, 0%]	2 [0%, 0%, 0%]	0.822 [0%, 0%, 0%]	0 [0%, 0%, 0%]	4,886 [1% of Total]
-10m to -50m	26,539 [31%, 51%, 4%]	20,646 [24%, 29%, 3%]	6,589 [8%, 19%, 1%]	8,264 [10%, 14%, 1%]	24,213 [28%, 6%, 4%]	0 [0%, 0%, 0%]	86,252 [14% of Total]
-50m to -100m	16,228 [8%, 31%, 3%]	36,491 [19%, 50%, 6%]	18,566 [10%, 53%, 3%]	29,025 [15%, 51%, 5%]	93,001 [48%, 24%, 15%]	0 [0%, 0%, 0%]	193,311 [32% of Total]
-100m to -150m	4,334 [3%, 8%, 1%]	13,175 [9%, 18%, 2%]	8,618 [6%, 25%, 1%]	15,547 [11%, 27%, 3%]	98,868 [70%, 25%, 16%]	0 [0%, 0%, 0%]	140,543 [23% of Total]
-150m to -200m	660 [2%, 1%, 0%]	1,271 [4%, 2%, 0%]	1,196 [3%, 3%, 0%]	3,536 [10%, 6%, 1%]	27,699 [81%, 7%, 5%]	0.362 [0%, 0%, 0%]	34,363 [6% of Total]
<-200m	91 [0%, 0%, 0%]	274 [0%, 0%, 0%]	47 [0%, 0%, 0%]	813 [1%, 1%, 0%]	147,268 [99%, 38%, 24%]	612 [0%, 100%, 0%]	149,105 [25% of Total]
Total	52,283 [9% of Total]	72,260 [12% of Total]	35,065 [6% of Total]	57,187 [9% of Total]	391,051 [64% of Total]	612 [0% of Total]	608,459 Km²

The designations employed and the presentation of material in the map do not imply the expression of any opinion whatsoever on the part of FAO concerning the legal or constitutional status of any country, territory or sea area, or concerning the delimitation of frontiers.

Background reference map from National Geographic. Content may not reflect National Geographic's current map policy. Sources: National Geographic, Esri, DeLorme, HERE, UNEP-WCMC, USGS, NASA, ESA, METI, NRCAN, GEBCO, NOAA, increment P Corp.

Projection: Azimuthal Equidistant
Datum: WGS 1984
False Easting: 0.0000
False Northing: 0.0000
Central Meridian: -5.7487
Latitude Of Origin: 49.9490

Food and Agriculture Organization of the United Nations

United Kingdom: Caribbean Region

©FAO, 2018

0 125 250 375 500 625 750 875 1,000 1,125 Kilometers

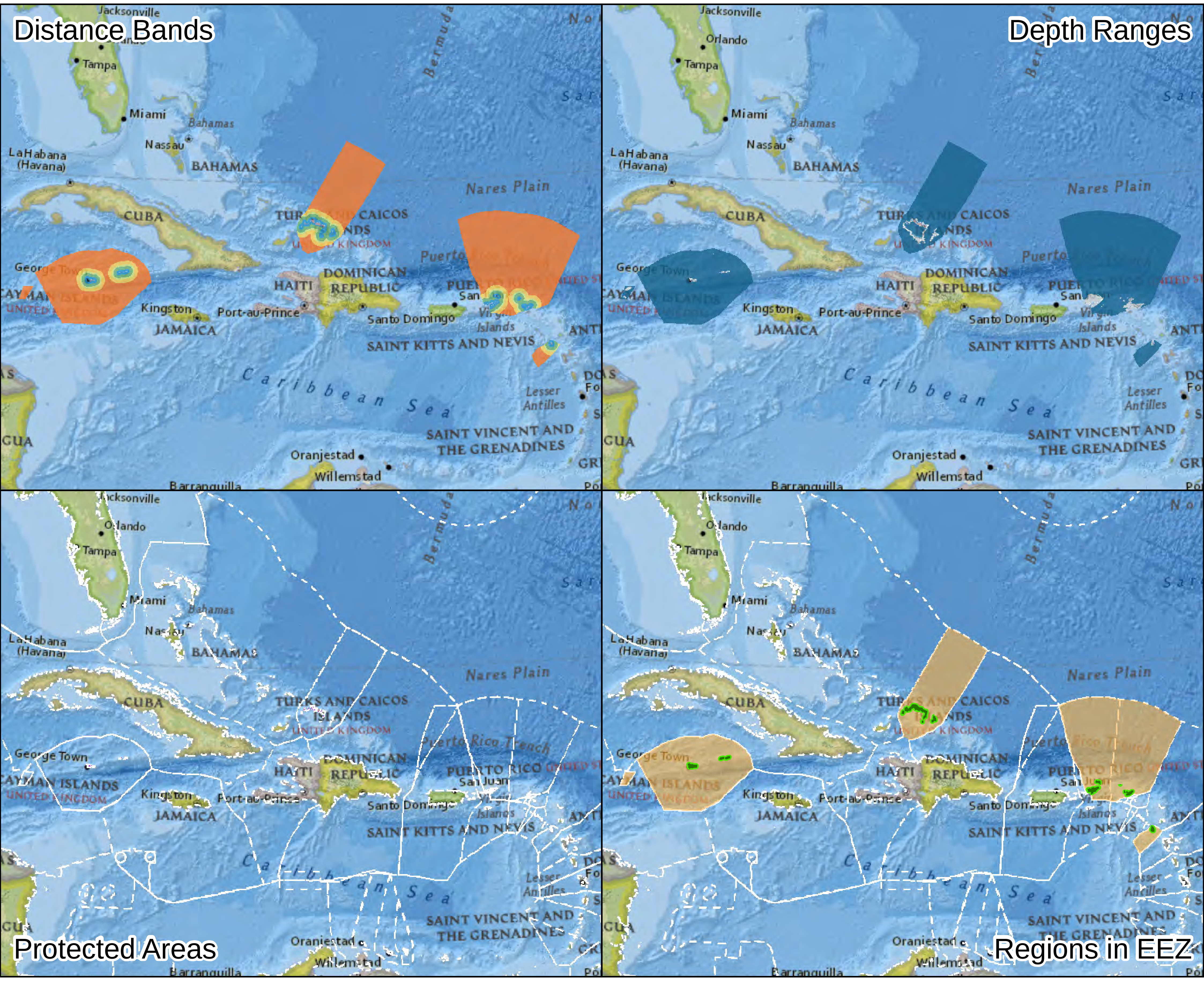

Distance Bands

- 0 to 5 NM
- 5 to 12 NM
- 12 to 16 NM
- 16 to 24 NM
- 24 to 200 NM
- > 200 NM

Depth Bands

- > -10m
- -10m to -50m
- -50m to -100m
- -100m to -150m
- -150m to -200m
- < -200m

Protected

- Protected Areas

Coastline Length

2,892 Km

Region

- Caribbean

All Areas in United Kingdom EEZ: Cell Values = Area in Km^2 [% Depth Band (Row), % Distance Band (Column), % EEZ]

	0 to 5 NM	5 to 12 NM	12 to 16 NM	16 to 24 NM	24 to 200 NM	> 200 NM	Total
> -10m	1,200 [72%, 11%, 0%]	162 [10%, 1%, 0%]	71 [4%, 1%, 0%]	93 [6%, 0%, 0%]	151 [9%, 0%, 0%]	0 [0%, 0%, 0%]	1,677 [0% of Total]
-10m to -50m	2,665 [54%, 24%, 1%]	1,386 [28%, 7%, 0%]	409 [8%, 4%, 0%]	244 [5%, 1%, 0%]	207 [4%, 0%, 0%]	0 [0%, 0%, 0%]	4,911 [1% of Total]
-50m to -100m	687 [36%, 6%, 0%]	450 [23%, 2%, 0%]	352 [18%, 3%, 0%]	317 [16%, 1%, 0%]	120 [6%, 0%, 0%]	0 [0%, 0%, 0%]	1,925 [0% of Total]
-100m to -150m	378 [39%, 3%, 0%]	190 [19%, 1%, 0%]	130 [13%, 1%, 0%]	201 [21%, 1%, 0%]	79 [8%, 0%, 0%]	0 [0%, 0%, 0%]	979 [0% of Total]
-150m to -200m	356 [38%, 3%, 0%]	208 [22%, 1%, 0%]	106 [11%, 1%, 0%]	200 [21%, 1%, 0%]	76 [8%, 0%, 0%]	0 [0%, 0%, 0%]	946 [0% of Total]
<-200m	5,724 [2%, 52%, 1%]	16,933 [4%, 88%, 4%]	10,593 [3%, 91%, 3%]	23,337 [6%, 96%, 6%]	324,391 [85%, 100%, 83%]	37 [0%, 100%, 0%]	381,015 [97% of Total]
Total	11,011 [3% of Total]	19,329 [5% of Total]	11,661 [3% of Total]	24,392 [6% of Total]	325,025 [83% of Total]	37 [0% of Total]	391,454 Km^2

Areas in United Kingdom EEZ Excluding Protected Areas: Cell Values = Area in Km^2 [% Depth Band (Row), % Distance Band (Column), % EEZ]

256 [0%] Km^2 Protected	0 to 5 NM	5 to 12 NM	12 to 16 NM	16 to 24 NM	24 to 200 NM	> 200 NM	Total
> -10m	1,090 [70%, 10%, 0%]	160 [10%, 1%, 0%]	71 [5%, 1%, 0%]	93 [6%, 0%, 0%]	151 [10%, 0%, 0%]	0 [0%, 0%, 0%]	1,565 [0% of Total]
-10m to -50m	2,620 [54%, 24%, 1%]	1,386 [28%, 7%, 0%]	409 [8%, 4%, 0%]	244 [5%, 1%, 0%]	207 [4%, 0%, 0%]	0 [0%, 0%, 0%]	4,866 [1% of Total]
-50m to -100m	663 [35%, 6%, 0%]	450 [24%, 2%, 0%]	352 [19%, 3%, 0%]	317 [17%, 1%, 0%]	120 [6%, 0%, 0%]	0 [0%, 0%, 0%]	1,901 [0% of Total]
-100m to -150m	369 [38%, 3%, 0%]	190 [20%, 1%, 0%]	130 [13%, 1%, 0%]	201 [21%, 1%, 0%]	79 [8%, 0%, 0%]	0 [0%, 0%, 0%]	969 [0% of Total]
-150m to -200m	348 [37%, 3%, 0%]	207 [22%, 1%, 0%]	106 [11%, 1%, 0%]	200 [21%, 1%, 0%]	76 [8%, 0%, 0%]	0 [0%, 0%, 0%]	937 [0% of Total]
<-200m	5,673 [1%, 53%, 1%]	16,929 [4%, 88%, 4%]	10,593 [3%, 91%, 3%]	23,337 [6%, 96%, 6%]	324,391 [85%, 100%, 83%]	37 [0%, 100%, 0%]	380,959 [97% of Total]
Total	10,762 [3% of Total]	19,321 [5% of Total]	11,661 [3% of Total]	24,392 [6% of Total]	325,025 [83% of Total]	37 [0% of Total]	391,198 Km^2

The designations employed and the presentation of material in the map do not imply the expression of any opinion whatsoever on the part of FAO concerning the legal or constitutional status of any country, territory or sea area, or concerning the delimitation of frontiers.

Background reference map from National Geographic. Content may not reflect National Geographic's current map policy. Sources: National Geographic, Esri, DeLorme, HERE, UNEP-WCMC, USGS, NASA, ESA, METI, NRCAN, GEBCO, NOAA, increment P Corp.

Projection: Azimuthal Equidistant
Datum: WGS 1984
False Easting: 0.0000
False Northing: 0.0000
Central Meridian: -72.4683
Latitude Of Origin: 20.4370

Food and Agriculture Organization of the United Nations

United States: Gulf of Mexico and Caribbean Regions

©FAO, 2018

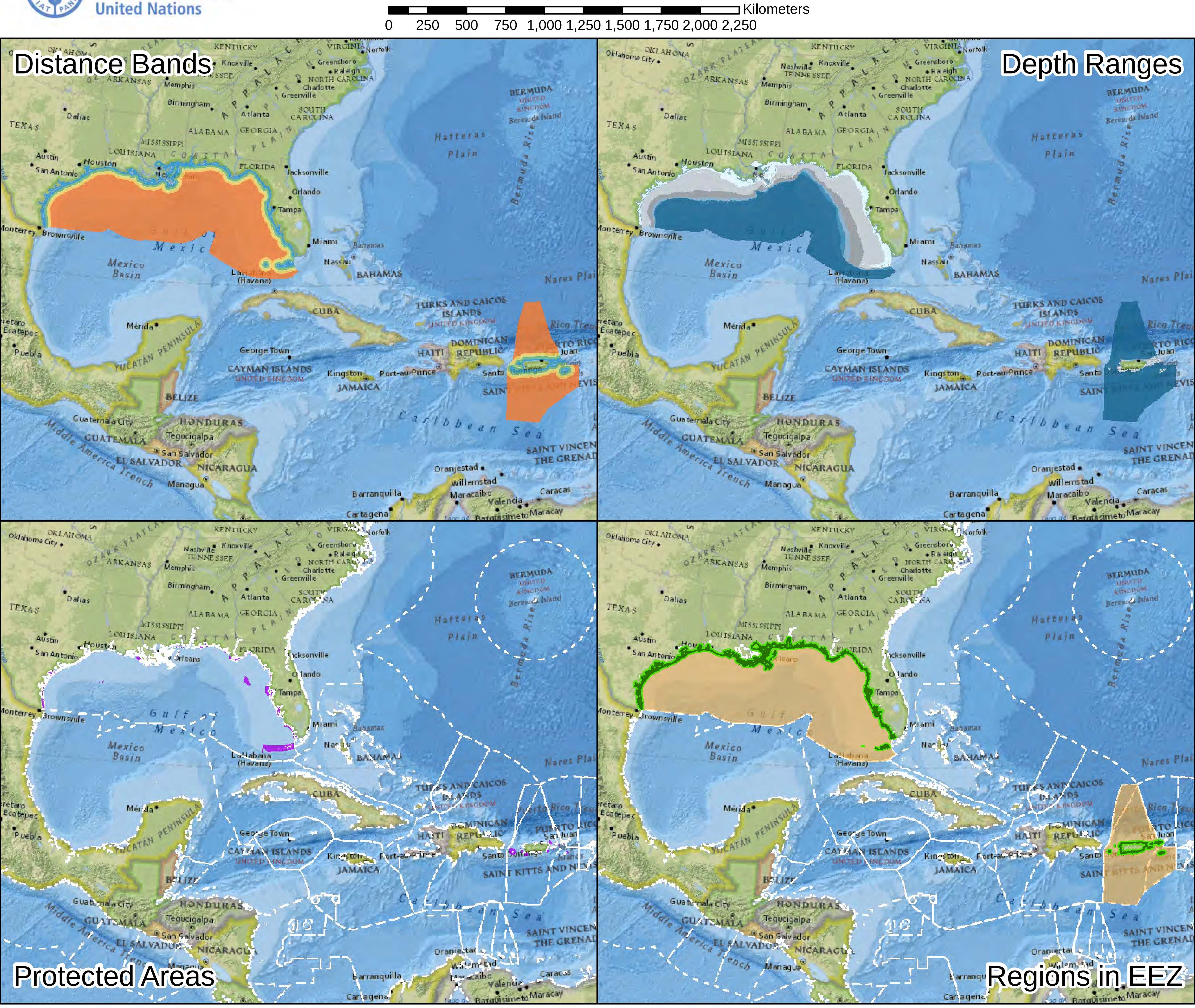

Distance Bands: 0 to 5 NM; 5 to 12 NM; 12 to 16 NM; 16 to 24 NM; 24 to 200 NM; > 200 NM

Depth Bands: > -10m; -10m to -50m; -50m to -100m; -100m to -150m; -150m to -200m; < -200m

Protected: Protected Areas

Region: Caribbean

Coastline Length: 31,454 Km

All Areas in United States EEZ: Cell Values = Area in Km² [% Depth Band (Row), % Distance Band (Column), % EEZ]

	0 to 5 NM	5 to 12 NM	12 to 16 NM	16 to 24 NM	24 to 200 NM	> 200 NM	Total
> -10m	40,971 [70%, 69%, 4%]	11,500 [20%, 21%, 1%]	3,527 [6%, 12%, 0%]	2,808 [5%, 5%, 0%]	38 [0%, 0%, 0%]	0 [0%, 0%, 0%]	58,844 [6% of Total]
-10m to -50m	12,923 [8%, 22%, 1%]	25,325 [15%, 47%, 3%]	15,014 [9%, 51%, 2%]	31,273 [18%, 56%, 3%]	86,822 [51%, 12%, 9%]	0 [0%, 0%, 0%]	171,356 [19% of Total]
-50m to -100m	960 [1%, 2%, 0%]	1,868 [3%, 3%, 0%]	1,099 [2%, 4%, 0%]	2,835 [4%, 5%, 0%]	65,092 [91%, 9%, 7%]	0 [0%, 0%, 0%]	71,855 [8% of Total]
-100m to -150m	194 [1%, 0%, 0%]	794 [3%, 1%, 0%]	315 [1%, 1%, 0%]	738 [3%, 1%, 0%]	22,197 [92%, 3%, 2%]	0 [0%, 0%, 0%]	24,237 [3% of Total]
-150m to -200m	192 [1%, 0%, 0%]	770 [4%, 1%, 0%]	548 [3%, 2%, 0%]	518 [3%, 1%, 0%]	16,187 [89%, 2%, 2%]	0 [0%, 0%, 0%]	18,215 [2% of Total]
<-200m	4,558 [1%, 8%, 0%]	13,348 [2%, 25%, 1%]	8,787 [2%, 30%, 1%]	17,556 [3%, 32%, 2%]	527,869 [92%, 73%, 58%]	269 [0%, 100%, 0%]	572,387 [62% of Total]
Total	59,798 [7% of Total]	53,605 [6% of Total]	29,289 [3% of Total]	55,728 [6% of Total]	718,205 [78% of Total]	269 [0% of Total]	916,893 Km²

Areas in United States EEZ Excluding Protected Areas: Cell Values = Area in Km² [% Depth Band (Row), % Distance Band (Column), % EEZ]

18,646 [2%] Km² Protected	0 to 5 NM	5 to 12 NM	12 to 16 NM	16 to 24 NM	24 to 200 NM	> 200 NM	Total
> -10m	32,657 [66%, 67%, 4%]	10,711 [22%, 22%, 1%]	3,448 [7%, 12%, 0%]	2,768 [6%, 5%, 0%]	38 [0%, 0%, 0%]	0 [0%, 0%, 0%]	49,623 [6% of Total]
-10m to -50m	11,485 [7%, 23%, 1%]	23,358 [14%, 48%, 3%]	14,448 [9%, 51%, 2%]	30,320 [18%, 56%, 3%]	85,640 [52%, 12%, 10%]	0 [0%, 0%, 0%]	165,251 [18% of Total]
-50m to -100m	833 [1%, 2%, 0%]	1,459 [2%, 3%, 0%]	987 [1%, 3%, 0%]	2,777 [4%, 5%, 0%]	65,022 [91%, 9%, 7%]	0 [0%, 0%, 0%]	71,077 [8% of Total]
-100m to -150m	147 [1%, 0%, 0%]	746 [3%, 2%, 0%]	302 [1%, 1%, 0%]	722 [3%, 1%, 0%]	22,127 [92%, 3%, 2%]	0 [0%, 0%, 0%]	24,044 [3% of Total]
-150m to -200m	149 [1%, 0%, 0%]	761 [4%, 2%, 0%]	548 [3%, 2%, 0%]	500 [3%, 1%, 0%]	16,187 [89%, 2%, 2%]	0 [0%, 0%, 0%]	18,144 [2% of Total]
<-200m	3,618 [1%, 7%, 0%]	12,063 [2%, 25%, 1%]	8,787 [2%, 31%, 1%]	17,500 [3%, 32%, 2%]	527,869 [93%, 74%, 59%]	269 [0%, 100%, 0%]	570,107 [63% of Total]
Total	48,888 [5% of Total]	49,099 [5% of Total]	28,521 [3% of Total]	54,588 [6% of Total]	716,883 [80% of Total]	269 [0% of Total]	898,247 Km²

The designations employed and the presentation of material in the map do not imply the expression of any opinion whatsoever on the part of FAO concerning the legal or constitutional status of any country, territory or sea area, or concerning the delimitation of frontiers.

Background reference map from National Geographic. Content may not reflect National Geographic's current map policy. Sources: National Geographic, Esri, DeLorme, HERE, UNEP-WCMC, USGS, NASA, ESA, METI, NRCAN, GEBCO, NOAA, increment P Corp.

Projection: Azimuthal Equidistant
Datum: WGS 1984
False Easting: 0.0000
False Northing: 0.0000
Central Meridian: -80.8265
Latitude Of Origin: 22.8693

Food and Agriculture Organization of the United Nations

Venezuela: Caribbean Region

©FAO, 2018

Kilometers: 0 125 250 375 500 625 750 875

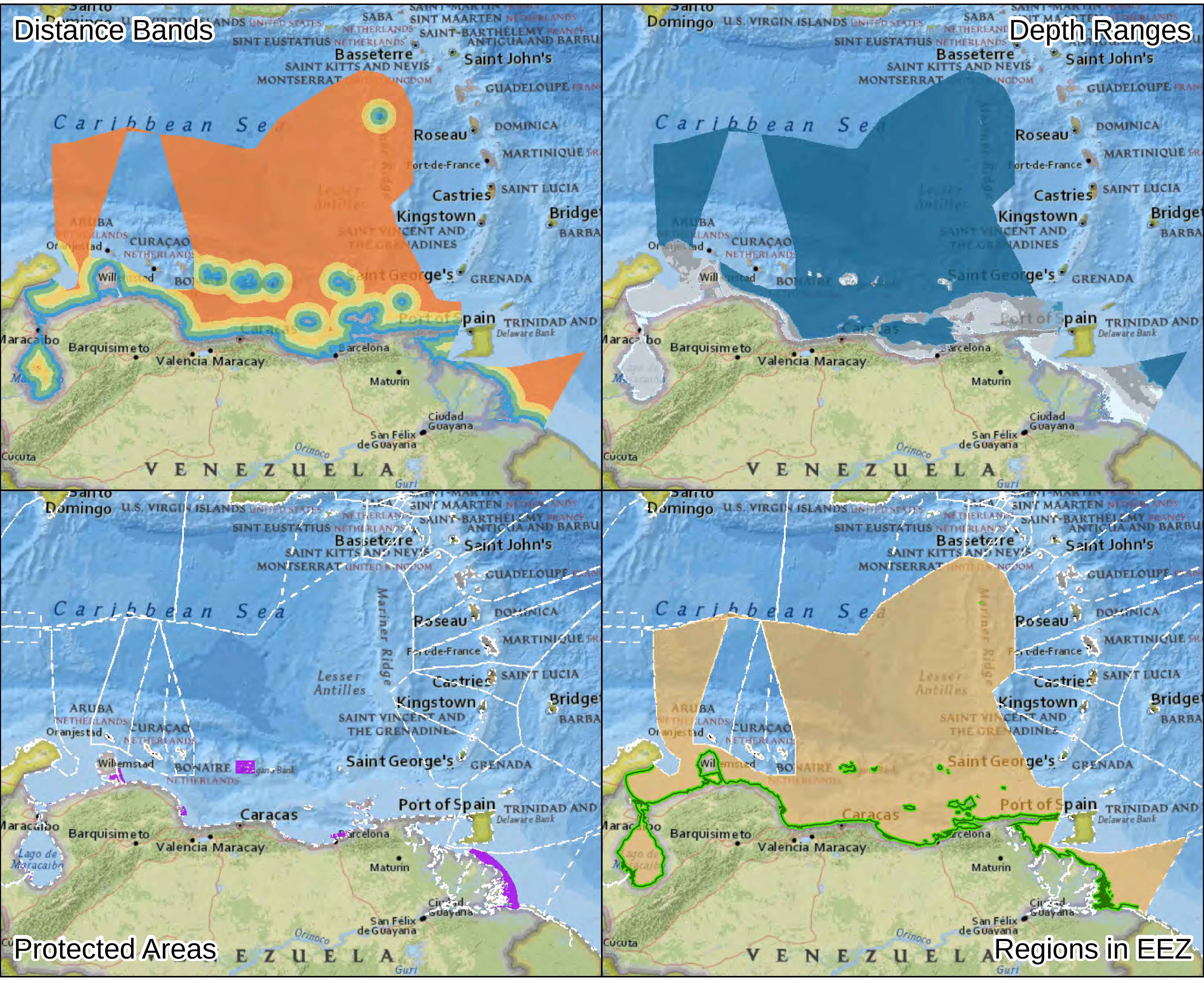

Distance Bands: 0 to 5 NM; 5 to 12 NM; 12 to 16 NM; 16 to 24 NM; 24 to 200 NM

Depth Bands: > -10m; -10m to -50m; -50m to -100m; -100m to -150m; -150m to -200m; < -200m

Protected: Protected Areas

Region: Caribbean

Coastline Length: 5,882 Km

All Areas in Venezuela EEZ: Cell Values = Area in Km^2 [% Depth Band (Row), % Distance Band (Column), % EEZ]

	0 to 5 NM	5 to 12 NM	12 to 16 NM	16 to 24 NM	24 to 200 NM	> 200 NM	Total
> -10m	11,840 [75%, 32%, 3%]	2,751 [18%, 6%, 1%]	485 [3%, 2%, 0%]	161 [1%, 0%, 0%]	450 [3%, 0%, 0%]	0 [0%, 0%, 0%]	15,687 [3% of Total]
-10m to -50m	15,164 [30%, 41%, 3%]	16,734 [33%, 37%, 4%]	7,759 [15%, 29%, 2%]	9,183 [18%, 19%, 2%]	2,245 [4%, 1%, 0%]	0 [0%, 0%, 0%]	51,085 [11% of Total]
-50m to -100m	3,912 [13%, 11%, 1%]	6,313 [21%, 14%, 1%]	3,311 [11%, 12%, 1%]	6,057 [21%, 13%, 1%]	9,904 [34%, 3%, 2%]	0 [0%, 0%, 0%]	29,497 [6% of Total]
-100m to -150m	1,279 [17%, 3%, 0%]	1,673 [22%, 4%, 0%]	738 [10%, 3%, 0%]	1,672 [22%, 3%, 0%]	2,111 [28%, 1%, 0%]	0 [0%, 0%, 0%]	7,472 [2% of Total]
-150m to -200m	594 [14%, 2%, 0%]	1,241 [29%, 3%, 0%]	413 [10%, 2%, 0%]	687 [16%, 1%, 0%]	1,279 [30%, 0%, 0%]	0 [0%, 0%, 0%]	4,214 [1% of Total]
<-200m	3,934 [1%, 11%, 1%]	16,688 [5%, 37%, 4%]	13,866 [4%, 52%, 3%]	30,080 [8%, 63%, 6%]	300,396 [82%, 95%, 64%]	0 [0%, 0%, 0%]	364,964 [77% of Total]
Total	36,723 [8% of Total]	45,399 [10% of Total]	26,573 [6% of Total]	47,840 [10% of Total]	316,385 [67% of Total]	0.000 [0% of Total]	472,921 Km^2

Areas in Venezuela EEZ Excluding Protected Areas: Cell Values = Area in Km^2 [% Depth Band (Row), % Distance Band (Column), % EEZ]

7,450 [2%] Km^2 Protected	0 to 5 NM	5 to 12 NM	12 to 16 NM	16 to 24 NM	24 to 200 NM	> 200 NM	Total
> -10m	9,131 [75%, 29%, 2%]	1,975 [16%, 5%, 0%]	485 [4%, 2%, 0%]	161 [1%, 0%, 0%]	450 [4%, 0%, 0%]	0 [0%, 0%, 0%]	12,203 [3% of Total]
-10m to -50m	13,260 [27%, 43%, 3%]	15,950 [33%, 37%, 3%]	7,709 [16%, 29%, 2%]	9,183 [19%, 19%, 2%]	2,245 [5%, 1%, 0%]	0 [0%, 0%, 0%]	48,349 [10% of Total]
-50m to -100m	3,462 [12%, 11%, 1%]	6,208 [21%, 14%, 1%]	3,311 [11%, 12%, 1%]	6,057 [21%, 13%, 1%]	9,904 [34%, 3%, 2%]	0 [0%, 0%, 0%]	28,942 [6% of Total]
-100m to -150m	1,103 [15%, 4%, 0%]	1,655 [23%, 4%, 0%]	738 [10%, 3%, 0%]	1,672 [23%, 3%, 0%]	2,111 [29%, 1%, 0%]	0 [0%, 0%, 0%]	7,279 [2% of Total]
-150m to -200m	552 [13%, 2%, 0%]	1,232 [30%, 3%, 0%]	413 [10%, 2%, 0%]	687 [16%, 1%, 0%]	1,279 [31%, 0%, 0%]	0 [0%, 0%, 0%]	4,162 [1% of Total]
<-200m	3,628 [1%, 12%, 1%]	16,565 [5%, 38%, 4%]	13,866 [4%, 52%, 3%]	30,080 [8%, 63%, 6%]	300,396 [82%, 95%, 65%]	0 [0%, 0%, 0%]	364,536 [78% of Total]
Total	31,136 [7% of Total]	43,587 [9% of Total]	26,522 [6% of Total]	47,840 [10% of Total]	316,385 [68% of Total]	0.000 [0% of Total]	465,470 Km^2

The designations employed and the presentation of material in the map do not imply the expression of any opinion whatsoever on the part of FAO concerning the legal or constitutional status of any country, territory or sea area, or concerning the delimitation of frontiers.

Background reference map from National Geographic. Content may not reflect National Geographic's current map policy. Sources: National Geographic, Esri, DeLorme, HERE, UNEP-WCMC, USGS, NASA, ESA, METI, NRCAN, GEBCO, NOAA, increment P Corp.

Projection: Azimuthal Equidistant
Datum: WGS 1984
False Easting: 0.0000
False Northing: 0.0000
Central Meridian: -65.4734
Latitude Of Origin: 12.5558
